# Genome‐Wide Association Analysis of Grain Yield‐Associated Traits in a Pan‐European Barley Cultivar Collection

**DOI:** 10.3835/plantgenome2017.08.0073

**Published:** 2018-03-01

**Authors:** Xin Xu, Rajiv Sharma, Alessandro Tondelli, Joanne Russell, Jordi Comadran, Florian Schnaithmann, Klaus Pillen, Benjamin Kilian, Luigi Cattivelli, William T. B. Thomas, Andrew J. Flavell

**Affiliations:** ^1^ Univ. of Dundee at JHI Invergowrie Dundee DD2 5DA UK; ^2^ Present address: Hubei Provincial Key Lab. for Protection and Application of Special Plants in Wuling Area of China, College of Life Sciences, South‐Central Univ. for Nationalities Wuhan China 430074; ^3^ Univ. of Dundee at JHI Invergowrie Dundee DD2 5DA UK; ^4^ Present address: Leibniz Institute of Plant Genetics and Crop Plant Research (IPK) Corrensstrasse 3 06466 Gatersleben Germany; ^5^ Univ. of Dundee at JHI Invergowrie Dundee DD2 5DA UK; ^6^ Leibniz Institute of Plant Genetics and Crop Plant Research (IPK) Corrensstrasse 3 06466 Gatersleben Germany; ^7^ Present address: Global Crop Diversity Trust Platz der Vereinten Nationen 7 53113 Bonn Germany; ^8^ Council for Agricultural Research and Economics (CREA), Research Centre for Genomics and Bioinformatics Via S. Protaso, 302. I ‐29017 Fiorenzuola d'Arda PC Italy; ^9^ James Hutton Institute Invergowrie Dundee DD2 5DA UK; ^10^ Martin‐Luther‐Univ. Halle‐Wittenberg Betty‐Heimann‐Str. 3 06120 Halle/Saale Germany

## Abstract

A collection of 379 Hordeum vulgare cultivars, comprising all combinations of spring and winter growth habits with two and six row ear type, was screened by genome wide association analysis to discover alleles controlling traits related to grain yield. Genotypes were obtained at 6,810 segregating gene‐based single nucleotide polymorphism (SNP) loci and corresponding field trial data were obtained for eight traits related to grain yield at four European sites in three countries over two growth years. The combined data were analyzed and statistically significant associations between the traits and regions of the barley genomes were obtained. Combining this information with the high resolution gene map for barley allowed the identification of candidate genes underlying all scored traits and superposition of this information with the known genomics of grain trait genes in rice resulted in the assignation of 13 putative barley genes controlling grain traits in European cultivated barley. Several of these genes are associated with grain traits in both winter and spring barley.

**Abbreviations**
BLUE: best linear unbiased estimate
CRA: Genomics Research Centre; Fiorenzuola d'Arda; Italy
EL: ear length
GA: grain area
G/E: grains per ear
GL: grain length
GR: grain roundness
GW: grain width
GWAS: genome‐wide association scan/scanning
GY: Grain yield
IPK: Leibniz Institute; Gatersleben; Germany
JHI: James Hutton Institute; Dundee; UK
LR‐PC: Low‐recombining Peri‐centromeric
QTL: quantitative trait locus
SNP: single nucleotide polymorphism
TGW: thousand grain weight
UHA: Martin‐Luther‐University of Halle‐Wittenberg; Halle; Germany

## Core Ideas

- The GWAS revealed associations for 8 yield‐associated traits in 379 barley cultivars.
- Many barley grain and ear dimension traits are highly correlated.
- In 29 corresponding hotspots, 45% of the grain trait component QTLs overlap.
- Nine hotspots map to barley yield loci or grain trait orthologues of other cereals.

The challenge of feeding an increasing world population using a finite amount of growing space under increasingly uncertain climatic conditions has intensified the search for improved crop yield. Cereals comprise more than 50% of the world's total calorific intake (FAO, 2003) and the Triticeae family is a major contributor to this, with wheat, barley, and rye being the most important Triticeae crops. Improving the genetic component of crop yield requires selection of superior gene alleles and/or allele combinations, which are taken almost always from the existing cultivated crop germplasm pool. Cultivar germplasm already contains optimized alleles that are a narrow subset of the total genetic diversity originally available in the wild progenitor species (Tanksley and McCouch, 1997). This residual allelic diversity is sampled and resorted into new combinations by the breeder in search of improved lines for agriculture.

The most important agronomic trait by far in crop breeding is yield. Grain yield is a complex trait with quite low heritability and is the compound of multiple interacting component traits (Jiang et al., 2004, Zhao et al., 2016). The biggest improvements in yield in cereal breeding are associated with optimization of flowering time and plant height (Snape et al., 2001, Cockram et al., 2007, Hedden, 2003, Nadolska‐Orczyk et al., 2017). Optimal flowering time allows optimal grain development with regard to the availability of heat, light and water, while semi‐dwarf cereals allocate more resources into grain production than taller plants and show reduced losses through lodging. Major genes controlling flowering time and semi‐dwarfism have been identified and the best alleles are tending to become fixed in modern breeding germplasm (Jung and Müller, 2009; Jia et al., 2011). The scope for further improvement via this route may therefore be limited and this may in part be the reason that yield improvements of barley and wheat due to breeding over the past 20 yr are stagnating (Ray et al., 2012).

One promising avenue for future barley yield improvement is optimization of the component traits that contribute to grain yield using marker‐assisted selection. These include grain weight, the number of grains per ear, the number of ears per plant, and the number of plants per unit area. The first two components are more strongly inherited and thus are potential targets for indirect selection of yield but the last two, which determine the number of ears per unit area, are affected greatly by environmental variation and agronomic management. Growers tend, however, to sow at a constant seed density to establish a target number of ears per unit area so selection for the first two components is likely to have a greater effect on increasing grain yield. Grain shape and uniformity also feed into grain quality, which is another desirable trait because of its beneficial effect on malting quality. Quantitative trait loci (QTL) affecting these grain parameters have been defined and mapped in barley (Walker et al., 2013; Cu et al., 2016; Mikołajczak et al., 2016; Zhou et al., 2016; Maurer et al. 2016). These traits tend to interact with each other and increase in one of them (*e.g*. thousand grain weight) can be associated with reduction in another, (*e.g*. grains per ear). Nevertheless, independent improvement is achievable (Zhou et al., 2016).

Molecular markers are a key tool in the modern breeding process and the availability of genome‐wide, high throughput SNP markers for barley has enabled the genotyping of germplasm collections comprising hundreds of samples at thousands of gene loci (*e.g*. Tondelli et al., 2013). This has facilitated the detailed description of the genetic diversity of cultivated barley at both the genome‐wide and gene levels. The marker data can be combined with corresponding trait data using a genome‐wide association scan (GWAS) to reveal associations between genomic loci and advantageous traits for the breeder, (Russell et al., 2011; Kilian and Graner, 2012; Tondelli et al., 2013). These loci then become targets for crop improvement by the breeder. The GWAS is very effective at identifying major genes regulating mono‐ or oligogenic agricultural traits (Cockram et al., 2010, Ramsay et al., 2011, Comadran et al., 2012) but has been less successful for yield‐related traits, because these follow polygenic quantitative inheritance and, in addition, depend on genotype and environment interaction. Yield component QTL each contribute a small part of the total value for a trait and multiple QTL for a given trait segregate independently in populations. Therefore, the exact genomic locations and the causal DNA polymorphisms encoding QTL are inherently difficult to determine. Nevertheless, GWAS has successfully yielded genomic locations for QTL in cereals (Visioni et al., 2013; Tondelli et al., 2013, Rhodes et al., 2014).

The identification of genomic locations for QTL using linked segregating markers is highly useful for marker‐assisted breeding. Nevertheless, the ultimate goal of GWAS is to identify causal polymorphisms in specific genes that are responsible for variation in trait(s) of interest, because this allows gene‐targeted searches for germplasm improvement. The rate‐limiting step in reaching this goal has moved on from the GWAS process and is currently the candidate gene validation step. It is relatively easy to delineate genomic regions containing genes controlling interesting QTL but very difficult to prove that a candidate gene in such a region is responsible for the trait variation observed, especially when the trait is quantitative. It is therefore important to optimize methods for selecting strong gene candidates among the multiple genes residing in a genomic region containing a useful QTL.

Selection of gene candidates for barley QTL can in principle be accelerated if the same genes (i.e., gene orthologs) operate to specify these loci in related crops. Multiple genes affecting grain traits have been identified in cereals other than barley, particularly rice (Huang et al., 2013), where the OsGW2 gene acts as a negative regulator of grain width and weight and mutation to its wheat ortholog TaGW2‐A1 confers significant increases in thousand grain weight (Simmonds et al., 2016). Another way to approach this is to compare genomic positions of QTLs specifying a given trait between related species and thus search for potentially orthologous QTL shared between species. One such study failed to find any orthologous genomic correspondence between grain trait QTL identified by GWAS in sorghum and known causative genes for grain traits in rice or maize (Zhang et al., 2015).

In this project we apply GWAS for yield‐associated component traits to a population of predominantly European barley cultivars which encompasses all possible combinations of spring and winter growth habit, together with two‐row and six‐row spike morphology. This allows us to both explore this germplasm set for useful yield‐related QTL and to investigate the extent to which genes affecting such traits overlap among these different germplasm sets that show strong population differentiation (Comadran et al., 2012). We also explore the overlap between the QTLs identified here and the locations of known loci affecting grain component traits in other cereals, to investigate the efficacy of candidate gene identification for grain traits in barley.

## MATERIALS AND METHODS

### Plant Materials and Phenotypic Evaluation

The association mapping panel consists of 379 barley accessions (Supplemental Table S1) selected from the Exbardiv Collection (Genomics‐Assisted Analysis and Exploitation of Barley Diversity; http://www.erapg.org). Each accession was derived from a single seed chosen at random from a genebank accession, selfed for two generations under greenhouse conditions and subsequently genotyped (see below) and propagated in the field. The panel was segregated by growth habit into 268 spring and 111 winter pools, which were grown correspondingly in spring‐ and autumn‐sown field trials (*i.e*. spring lines sown in the spring and winter lines sown in the autumn) at four different locations across Europe, namely Genomics Research Centre, Fiorenzuola d'Arda, Italy (CRA), James Hutton Institute, Dundee, UK (JHI), Leibniz Institute, Gatersleben, Germany (IPK) and the Martin‐Luther‐University of Halle‐Wittenberg, Germany (UHA) during the harvest seasons 2009 and 2010 as described by Tondelli et al. (2013). Plots between 2 and 3 m^2^ were grown using two‐replicate row and column design, with filler plots to complete a rectangular grid where necessary. Plots were grown according to local management practices for sowing rate and chemical inputs. When the majority of plots in each trial were ripe, a fixed number of ears (typically five) were manually collected from each plot, ear length (EL) was scored, the seed were cleaned and the following yield‐related traits were scored on the sample (typically between 200 and 250 grains) using a MARVIN grain analyzer (GTA Sensorik GmbH, Germany): grain length (GL), grain width (GW), grain area (GA = GL x GW), grain roundness (GR = GW/GL), thousand grain weight (TGW). Finally, the whole trial was harvested with a small plot combine, grain from each plot was weighed and the data were combined with the harvested plot area to derive grain yield (GY) in t/ha.

### Genotypic Analysis of Germplasm

DNA isolation and genotyping were as described by Tondelli et al. (2013). A set of 7864 high‐confidence, gene‐based SNPs incorporated into a single Illumina iSelect assay (Comadran et al., 2012) was used, yielding 6810 markers segregating in this germplasm set. Genotype data for spring two‐row germplasm and winter two‐row and six‐row germplasm were used by Tondelli et al. (2013) and Digel et al. (2016), respectively. The six‐row spring genotype data have not been reported previously. The complete SNP data set (379 lines by 6810 markers) is available on request. Within each crop type (winter or spring), SNPs were filtered to exclude markers with a minimum allele frequency of < 10 and > 20% missing values to give 5731 and 4343 useable SNPs for spring and winter growth habits, respectively.

### Genome‐Wide Association Analysis

Best linear unbiased estimators (BLUEs) for each trait and genotype from each trial were calculated separately, as described in Tondelli et al. (2013). Within each growth habit, the eight BLUEs for each genotype/trait combination were combined with the polymorphic genotypic data in a QTL × Environment GWAS using the ‘Single Trait Association Analysis (Multiple Environments)’ option of the QTL mapping procedures implemented in Genstat v14 (VSN International). In this context the term environment refers to each site‐year combination. The Eigen analysis option was used to control population sub‐structure. The setting ‘uniform covariance and unequal variance’ was considered to best model the variance/covariance structure of our data. A threshold of –log10(P) = 4 was used to identify significant associations (Tondelli et al., 2013, Long et al., 2013, Matthies et al., 2014). Estimated allelic effects were expressed relative to cv. Optic, (i.e., Optic alleles at all marker loci were coded ‘1’ and the alternative allele coded ‘0’) to obtain allelic effects and directionality of the trait value for SNPs. Cv. Optic was chosen as a reference because it was the most popular cultivar in UK at the time of our trials. Confidence intervals for the GWAS peaks were defined by the SNP with the highest –log10(P) value within a ± 10cM window and extended to the most distant marker within that window with whose –log10(P) was within a value of 1 of the peak value.

### *In Silico* Genomic Positioning Barley Orthologs of Cereal Genes Encoding Yield‐Associated QTLs

Barley orthologs of cereal genes encoding yield‐associated QTLs were positioned on the barley genome physical map (Mascher et al., 2017) by BLAST querying the barley high confidence gene coding sequences database (http://webblast.ipk‐gatersleben.de/barley_ibsc/index.php). To allow comparison between these barley orthologs and the QTL loci found here, the physical map positions were converted to genetic map positions, using a map containing 7480 Illumina iSelect SNP markers (Comadran et al., 2012) with both genetic and physical map locations derived by merging four preexisting maps (Mayer et al., 2012; Mascher et al., 2013; Tondelli et al., 2013; Muñoz‐Amatriaín et al., 2015) with the R program LPmerge (Endelman and Plomion, 2014) (Supplemental Table S2). Results from each growth habit were merged into a Flapjack (Milne et al., 2010) display for easy visualization and interpretation of QTL discovered.

## RESULTS

### Genotyping of a European Barley Cultivar Collection

A total of 379 *H. vulgare* cultivar accessions, containing all combinations of spring/winter growth habit and 2/6 row ear morphology, were genotyped using an Illumina iSelect marker set of 7864 SNPs (Comadran et al., 2012), 6810 of which are polymorphic within this germplasm set. After data filtering to remove markers with poor quality and/or minor allele frequency below 10%, 5638 segregating mapped markers and 93 segregating unmapped markers were selected for this study. Numbers of SNP markers mapping to each barley chromosome ranged from 532 (chromosome 1H) to 1161 (5H) (not shown), with corresponding marker densities (SNPs/cM) ranging between 5.57 (chromosome 1H) and 8.42 (2H).

### Heritability and Correlations of European Barley Yield Traits

The selected germplasm was grown in spring and autumn sown field trials at four different locations across Europe in two separate years (Tondelli et al., 2013) and 10 yield‐related traits were scored. Variance component analysis (Supplemental Fig. S1) shows that heritabilities were generally high (between 45 and 85%) for ear and grain parameters [GL, GW, GA, GR, TGW, grains per ear (G/E), EL] in both spring and winter germplasm sets, and moderate for GY (between 23 and 26%). Environmental effects of Site and Year were generally low to moderate for grain parameters (8–18%), low for ear parameters (3–6%, apart from winter EL = 15%) and moderate for grain yield (23–26%).

**Fig. 1:**
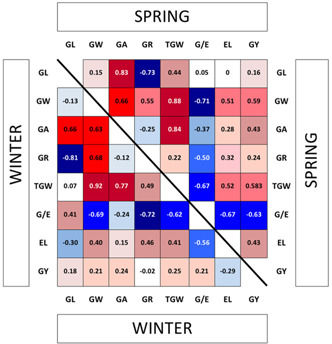
Correlations between traits in this study. Correlations within spring and winter germplasms are shown numerically and by heat mapping. Red = positive correlation, blue = negative correlation.

Correlations between the traits were also analyzed (Fig. 1). Many of the grain and ear dimensions are highly correlated: For example, in spring germplasm TGW correlates closely with GW and GA (*r* > 0.8) and both GY and EL correlate moderately (*r* > 0.5) with GW and TGW. In winter germplasm these correlations hold for TGW but are less pronounced (r between 0.21 and 0.41) for GY and EL. Interestingly, in spring germplasm GY correlates positively with every trait measured except G/E (significant negative correlation), whereas for winter germplasm almost all the correlations are lower and the negative correlation with G/E is not apparent. In fact, for spring germplasm G/E shows negative correlations with every other trait measured except GL. For our spring germplasm these data are consistent with the model that yield is dependent on grain size and ear length but not grains per ear. In addition, grain size and ear length can vary independently and the strongest determinant of grain weight is its width. For winter germplasm the correlations are weaker, presumably because of the lower numbers of lines involved.

### Genome‐Wide Association Analysis of Grain Yield Traits in European Barley Cultivars

The trait data were analyzed together with the marker data by genome‐wide association analysis (Supplemental Fig. S2). A complex set of marker‐trait associations was observed, with many –log_10_(P) values greater than a standard threshold of 3 dispersed across all barley linkage groups. To simplify these data to concentrate on the more highly significant QTL we applied a more stringent filter of –log_10_ *P* > 4 (Tondelli et al., 2013, Long et al., 2013, Matthies et al., 2014), resulting in 360 QTL Supplemental Table S3). A total of 217 and 143 QTL were found in spring and winter germplasm, respectively. Moreover, 114 QTL presented main effects, 246 presented QTLxE interaction. QTLs for each trait, EL, G/E, GA, GL, GR, GW, GY, and TGW, were 25, 49, 62, 66, 52, 38, 23, and 45, respectively.

### QTL Hotspots for Barley Grain Traits

**Fig. 2:**
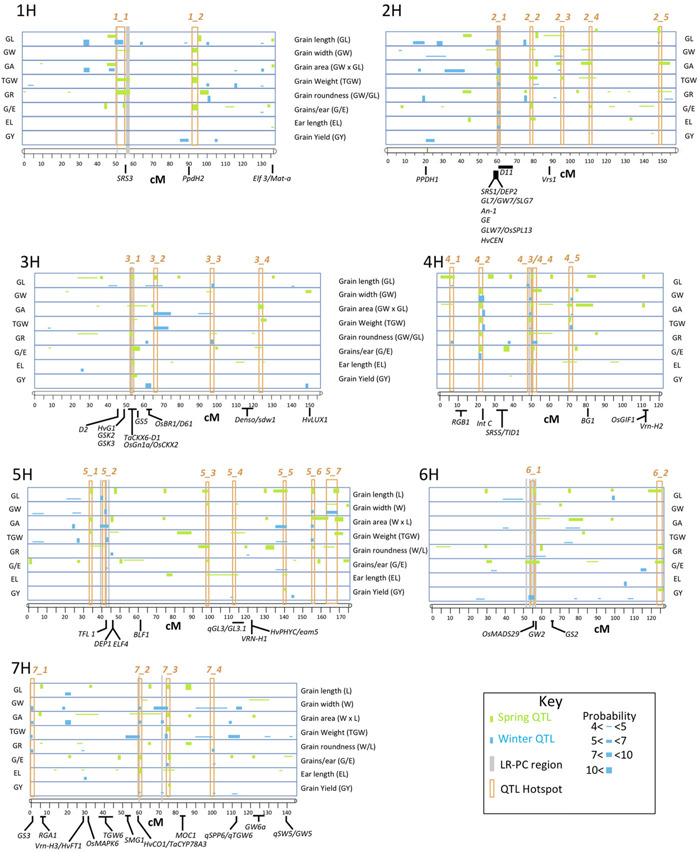
Barley grain trait QTLs– genetic distributions, probabilities and overlaps. The genetic positions of 342 barley grain trait QTLs identified here are plotted on a consensus linkage map (see Materials & Methods). The QTLs are categorized by their effects in spring (green) or winter (blue) growth habit germplasm, their Log_10_ probabilities and their grouping into QTL hotspots (see Key). Also plotted are genetic map locations for barley orthologs of known cereal grain trait genes (Supplemental Table S4), other developmental genes known to affect yield in barley and the low‐recombining peri‐centromeric (LR‐PC) genomic regions (Baker et al. 2014).

Not all the polymorphic SNPs used in the QTLxE analysis have known map locations and 18 of our QTL could not be plotted on the barley linkage map. When the 342 QTLs with genetic map positions were plotted on the barley linkage map, multiple overlaps between QTLs for different traits became apparent (Fig. 2). This is unsurprising, considering the large number of QTL revealed, their map resolution (averaging 2.9 cM, data not shown) and the correlations between many of these traits (Fig. 1). The QTL locations are shared both between different traits and/or different growth habits (spring/winter). There are 29 ‘hotspots’, where four or more QTLs coincide (Fig. 2; Table 1; Supplemental Table S3) and these together comprise 45% of all mapped QTL found. We suggest that important pleiotropic grain trait genes reside in these regions (see Discussion). Between 2 and 7 hotspots are found per chromosome and all seven centromere‐proximal low‐recombining peri‐centromeric (LR‐PC) regions contain a hotspot. Seventeen hotspots contain QTL specific to both winter and spring germplasm, 10 are spring germplasm‐specific and two are winter‐specific.

**Table 1:** QTL Hotspots for yield‐related traits in the barley genome.

Hotspot	QTL per region	Chromosome	Hotspot overlap region		Trait																Average Probability
			Start†	End†	GL		GW		GA		TGW		GR		G/E		EL		GY		
					S‡	W‡	S‡	W‡	S‡	W‡	S‡	W‡	S‡	W‡	S‡	W‡	S‡	W‡	S‡	W‡	
1_1	4	1H	**50.28**	**54.91**			4.85				5.69		9.03		9.05						7.15
1_2	4		92.32	95.36	6.28		8.14				10.57				32.73						14.43
2_1	8	2H	**60.91**	**61.04**	7.03		4.08		5.85	10.01	5.20	5.18				5.02		6.07			6.06
2_2	7		79.11	79.97			4.93				5.12	4.37			12.03		6.24				7.08
2_3	5		95.88	96.02	4.82		4.57	4.56	6.51		5.07										5.03
2_4	4		111.49	113.69			4.40		5.28		4.49				5.35						4.88
2_5	4		148.22	149.07	11.00	4.19			5.82				4.92								6.48
3_1	7	3H	**53.48**	**53.54**	9.87				4.17		4.48		7.44		7.71		9.30		6.13		7.01
3_2	5		66.57	66.91	7.99	4.78			5.41	5.92		5.49									5.92
3_3	5		97.42	97.42		9.27	4.97			4.21			4.62	7.66							6.25
3_4	4		124.58	124.62					7.89		5.51				4.81		4.19				5.60
4_1	4	4H	6.41	7.71	5.43	4.37			4.55					5.44							4.95
4_2	9		23.61	23.61			16.13	14.83	11.40	18.13	15.89	17.16	7.15		51.27	15.06					18.56
4_3	6		**49.57**	**49.57**		5.57		6.46		5.54	8.74	4.73	7.00								6.76
4_4	4		**50.82**	**50.97**	9.28				8.38					5.16	8.08						7.73
4_5	6		72.17	72.82				5.77	5.65	4.13	8.08	7.91					4.21				5.96
5_1	4	5H	33.45	35.51	13.25				8.55		5.03		4.23								7.76
5_2	5		**42.44**	**42.44**				8.11		5.12		9.53	4.23			4.52					6.46
5_3	5		97.69	99.01	5.83		6.16						5.23		6.63		4.25				5.62
5_4	4		113.36	114.09			4.97		4.73		4.29						4.25				4.59
5_5	6		139.54	140.93	5.09					6.01		4.48				4.42	4.94		5.45		5.06
5_6	6		155.14	155.52	10.54			6.97	5.23	5.92		5.64	6.33								6.77
5_7	5		162.41	169.62	7.04		4.46	5.89	8.38		5.79										5.89
6_1	7	6H	**55.18**	**55.46**	10.03		4.22		4.88		4.78			4.31	5.31					7.43	5.85
6_2	4		123.55	126.6	5.99								6.21		6.51				5.57		6.07
7_1	4	7H	0.001	0.26				7.39		5.32		6.02		5.13							5.97
7_2	7		**59.1**	**59.1**				6.31	4.81	6.32		6.33			7.31		10.31			4.31	6.53
7_3	9		74.79	74.99	8.34			5.34	9.76		7.71	8.90			12.66	6.48	4.63		6.46		7.35
7_4	5		99.13	100.33				4.87				4.00		6.57		4.18				4.29	4.78

† PC regions are in bold typeface; Hotspot overlap region is the maximum genetic distance containing all QTLs in a hotspot.

‡ S = Spring; W = Winter.

### Overlaps of Barley Grain Trait QTLs and QTL Hotspots with Characterized Cereal Genes affecting Grain Traits

To explore the possibility that the QTLs found here correspond to previously described genes affecting yield in barley and/or grain traits in other cereal species, we placed 15 mapped barley loci that affect grain yield (Nadolska‐Orczyk et al., 2017) and 30 barley orthologs of genes from other cereal species encoding yield‐associated QTLs (Supplemental Table S4) on our barley genome map and aligned these against our QTLs (Fig. 2, Supplemental Table S3). Twelve of the barley grain yield loci and 22 grain trait orthologs overlap with our QTLs and 2 and 12 map respectively to 9 QTL hotspots (Table 1; Supplemental Table S3). Eight of the 45 cereal loci show no overlap with QTLs found here.

If these cereal genes are causative for the QTLs that they overlap with then we would expect that their mutant phenotypic effects should correlate also, with the caution that many of the traits studied here are inter‐correlated and in most cases we are comparing barley QTLs with rice mutant phenotypes. Table 2 compares these effects. Five of the nine QTL hotspots show phenotypes consistent with the overlapping candidate genes being responsible for the traits, two hotspots show partially consistent phenotypes and two have phenotypes that are inconsistent with the candidate genes being responsible for the QTLs. The remaining 20 candidate gene‐containing regions showing overlap with one to three QTLs yield six consistent phenotypes, seven partially consistent phenotypes and seven inconsistent phenotypes. It is noteworthy that 10 candidate gene‐containing regions showing overlap with two to three QTLs showed only one inconsistent phenotype.

**Table 2:** Barley grain trait QTL overlaps with known cereal grain trait genes.

QTL hotspot	QTL nos†	Gene(s)	Mutant grain trait phenotype(s)	Mutant species	Overlapping barley grain trait QTLs in this study								Correlated phenotypic effect?
					GL	GW	GA	TGW	GR	G/E	EL	GY	
1_1	n/a	SRS3	Small round grain	Rice		GW		TGW	GR	G/E			Yes
2_1	n/a	SRS1/DEP2; An‐1; GE; D11; GLW7/OsSPL13	Small round grain, Grain number	Rice	GL	GW	GA	TGW		G/E	EL		Yes
2_2	n/a	Vrs1	2‐row/6‐row spike (lateral spikelet fertility)	Barley		GW		TGW		G/E	EL		Yes
3_1	n/a	OsGn1a/OsCKX2	Tiller number, grain yield	Rice	GL		GA	TGW	GR	G/E	EL	GY	Partial
4_1	n/a	RGB1	Small grain	Rice	GL		GA		GR				Yes
4_2	n/a	int‐C	2‐row/6‐row spike (lateral spikelet fertility)	Barley		GW	GA	TGW	GR	G/E			Yes
5_4	n/a	qGL3/GL3.1	Long grain	Rice		GW	GA	TGW			EL		No
6_1	n/a	OsMADS29; GW2	Wide grain, shrivelled grain	Rice	GL	GW	GA	TGW	GR	G/E		GY	Partial
7_1	n/a	GS3	Long grain	Rice		GW	GA	TGW	GR				No
–	177	BG1	Small grain	Rice			GA						Partial
–	205	Broad leaf1	Round grain	Barley						G/E			No
–	200–202	DEP1	Small grain (gain of function)	Barley, rice			GA	TGW	GR				Yes
–	202–203	ELF4	Grain yield	Barley	GL			TGW					No
–	56	GL7/GW7/SLG7	Long grain (gain of function)	Rice		GW							No
–	259	GS2	Large grain (gain of function)	Rice			GA						No
–	336–338	GW6a	Small grain	Rice		GW	GA			G/E			Partial
–	56	HvCEN	Grain yield	Barley		GW							No
–	302	HvCO1	Grain yield	Barley				TGW					Partial
–	134–135	HvLUX1	Grain yield	Barley		GW						GY	Yes
–	111–113	OsBRI/D61	Small grain	Rice	GL				GR			GY	Partial
–	298–299	OsMAPK6	Small grain	Rice	GL		GA						Yes
–	45–46	Ppd‐H1	Grain yield	Barley		GW						GY	Yes
–	333–335	qSPP6 qTGW6	Unknown (TGW QTL)	Rice		GW		TGW				GY	Yes
–	341	qSW5/GW5	Wide grain	Rice						G/E			No
–	286–288	RGA1	Small grain	Rice	GL		GA		GR				Partial
–	302	SMG1	Small grain	Rice				TGW					Yes
–	150–151	SRS5/TID1	Small round grain	Rice					GR	G/E			Partial
–	299	TGW	Large grain	Rice			GA						Partial
–	299	Vrn‐H3/HvFT1	Grain yield	Barley			GA						No
–	–	D2	Grain yield	Rice									n/a
–	–	denso/sdw1	Grain yield	Barley									n/a
–	–	Elf 3/Mat‐a	Grain yield	Barley									n/a
–	–	GSK2/GSK3	Large grain	Rice									n/a
–	–	MOC1	Grain yield	Rice									n/a
–	–	OsGIF1	Large grain (gain of function)	Barley									n/a
–	–	VRN‐H1; HvPHYC/eam5	Grain yield	Barley									n/a
–	–	Vrn‐H2	Grain yield	Barley									

† For QTL numbers, see Supplemental Table S3.

## DISCUSSION

In this work we have applied GWAS to a broad spectrum of yield component traits in a large European barley cultivars, encompassing all four combinations of row type (2/6) and growth habit (winter/spring). Yield is the most important trait for agriculture and is the product of multiple growth and development processes that occur throughout the barley life cycle. Therefore, many genes are expected to show direct (e.g. grain size) and indirect (e.g. developmental parameters) effects on barley yield. The most important barley genes controlling indirect effects regulate flowering time, including the responses to photoperiod and vernalization, and plant height (e.g., semi‐dwarf), (Comadran et al., 2011, Matthies et al., 2014, Nadolska‐Orczyk et al., 2017). The genomic locations of many such genes are well understood for barley (Von Zitzewitz et al., 2005; Yan et al., 2006; Jia et al., 2009; Digel et al., 2016) but the genes directly controlling yield parameters are more elusive and at present genes for these yield component traits have not been identified at the molecular level yet for barley.

### Genomic Hotspots for Barley Grain Trait QTLs

The GWAS has proven to be a powerful tool in detecting yield associated QTLs, in rice (Huang et al., 2012), wheat (Zanke et al., 2015) and maize (Liu et al., 2011). In this study, we have detected 360 QTLs [–log10(P) > 4] for eight yield‐associated traits in a panel of 379 barley accessions, using *ca* 7000 SNPs. This might seem to be an excessively large number but many of the traits studied here are highly correlated in the marker‐germplasm set and these likely represent pleiotropic phenotypic effects from a lower number of underlying loci. Our main reason supporting this hypothesis is the highly non‐random positioning of many of our QTLs in hotspots. (Fig. 2, Table 1).

The QTL hotspots for yield‐related component traits have been seen by others in cereals, e.g., barley (Mikołajczak et al., 2016; Wang et al., 2015), rice (Marathi et al., 2012) and wheat (Rustgi et al., 2013). Major QTLs in a hotspot region may obscure the effect of minor QTLs, making the latter more difficult to detect. For example, nine QTLs encompassing five traits are clustered on chromosome 4H at 23 cM (Table 1). This region contains the *int‐c* gene, which affects spike morphology (and therefore G/E) and interacts with other row‐type loci in barley (Ramsay et al., 2011). Several important yield‐related QTL, including TGW, have been reported to be tightly linked to *int*‐*c* (Comadran et al., 2011). In our study, the most significant QTLs for G/E, GW, and TGW are all in this region. The QTLs for GA and TGW showed positive effects in both spring barley and winter barley. Nevertheless QTLs of GW showed positive effects in spring barley and negative effects in winter barley, respectively and QTLs of G/E showed opposite effects in those two germplasms.

Another QTL hotspot for four traits (G/E, GW, EL, and TGW) maps quite close to *Vrs1* on chromosome 2H at 79 cM. *Vrs1* has previously been found to be a major locus affecting row type, TGW and grain size and shape parameters (Ayoub et al., 2002, Komatsuda et al., 2007). In our study, QTLs of EL, GW, and TGW (spring) showed positive effects and QTLs of G/E (spring) and TGW (winter) showed negative effects. The difference in between winter and spring barley effects on TGW in this region suggests that either two different genes are acting within this interval or different major haplotypes are segregating between winter and spring germplasms. Liller et al. (2015) suggested that pleiotropic effects between yield parameters for these barley row type genes are not only caused by competition of different plant organs for limited resources but also that row type genes may control development of different meristematic tissues.

### Correspondence between Barley Grain Trait QTLs and Other Cereal Loci affecting Yield‐Related Traits

Several of the QTLs found here map to the genomic locations of known barley major yield QTLs. For example, the most significant yield QTL in our study lies on chromosome 3H at 149.93 cM (Fig. 2, Supplemental Table S3), with a positive effect from the Optic cultivar allele. This locus may correspond to the *eam10* (early maturity 10) locus of barley and *HvLUX1*, an ortholog of the *Arabidopsis* circadian gene LUX. ARRHYTHMO, is a candidate gene for explaining its function. *Hvlux1* mutants cause circadian defects and interacts with the major barley photoperiod response gene *Ppd‐H1* to accelerate flowering under long‐day and short‐day conditions (Campoli et al., 2013).

Comparative mapping has provided a strategy to clone genes for important traits from cereal genomes. Although some research has suggested that *Brachypodium* is a better model for analysis of the genomes of temperate cereals like wheat and barley (Kumar et al., 2009; Girin et al., 2014), rice has been more successful as a model crop because of the huge amount of genetic, genomic and genotype‐trait data available in this species. Multiple genes, including those affecting yield and grain traits, have been identified in rice, providing a useful resource for isolating orthologs in another cereal species (see references in Supplemental Table S4). In wheat, homology‐based cloning has also become an efficient way to isolate grain size/weight genes. For instance, *TaGW2* was successfully mapped and isolated by this approach (Su et al., 2011; Ma et al., 2016).

The GWAS allows the mapping of markers linked to traits within relative narrow regions. Functional annotation of the genes located in these genomic regions can then be used to select candidate genes responsible for the linked traits (Mikołajczak et al., 2016; Zhou et al., 2016). However, this approach is not guaranteed to pinpoint the causative loci. For example, barley chromosome 2H is collinear with rice chromosome 4 for all markers surrounding *Vrs1*, but the rice *Vrs1* ortholog is on chromosome 7, indicating that the position of the gene has changed in evolutionary time. Zhang et al. (2015) failed to find convincing correspondence between grain trait QTL identified by GWAS in sorghum and known causative genes for grain traits in rice or maize. In contrast, our study has found many correspondences, particularly between rice grain trait genes and barley grain trait QTLs (Fig. 2, Supplemental Table S3). For example, the barley ortholog of the rice *GW2* gene is on Chromosome 6H at 54.82 cM, which contains a yield‐associated QTL hotspot. Orthologs of GRAIN WIDTH 2 (GW2) were proven to have similar effects on grain width and weight in rice, maize, and wheat (Li et al., 2010; Song et al., 2007; Su et al., 2011). This hotspot is peri‐centromeric (Fig. 1, Table 1) and therefore a region of low recombination and very high gene density relative to the genetic map. This reduces our confidence that the barley GW2 ortholog is responsible for these QTLs and a genome editing or TILLING approach would be required to provide stronger evidence that it was the candidate gene. However, correspondence to orthologs provides a potential solution to the widespread and very difficult problem of discovering causative genes in low‐recombining genomic regions. Similar examples include the barley orthologs of rice *SRS3* and *OsCKX2*, which map to hotspots *1_1* and *3_1* respectively and a complex of seven orthologs which map nearby or within hotspot *2_1* (Fig. 2, Supplemental Tables S3 and S4). *Hv‐SRS3* maps just outside the LR‐PC of chromosome 1H and *Hv‐CKX6* maps to a region containing the LR‐PCH of chromosome 3H. *CKX6‐D1* affects tiller number and yield in rice and grain weight in bread wheat (Yeh et al., 2015; Zhang et al., 2012).

We also have promising euchromatic barley gene candidates for the QTLs that they map to. The barley orthologs of *RGB1* (Hotspot *4_1*), *GL3* (Hotspot *5_4*) and *GS3* (Hotspot *7_1*) are strong candidate genes for further research and multiple orthologs mapping to one or more of the QTLs identified here, including *OsBRI/D6* (3H, 62–26cM), *SRS5/TID1* (4H, 31–37cM), *BG1* (4H, 81cM), *RGA* (7H, 3–6cM), *TGW6* (7H, 37–44cM), and *GW6a* (7H, 122cM) are worthy of further investigation (Fig. 2, Supplemental Table S3).

### Supplemental Materials

Supplemental Figure S1: Variance component analysis of traits studied here. Data from spring and winter trials are shown.

Supplemental Figure S2: GWAS plots for 8 yield‐related phenotypic traits in barley germplasm. Scans are color‐coded for trait as shown. X axis, Log_10_ probabilities. Y axes, marker numbers. The approximate locations of LR‐PCH regions (Baker et al. 2014) are indicated by gray shading.

Supplemental Table S1: Germplasm used in this study

Supplemental Table S2: Genetic linkage map for SNP markers used in this study

Supplemental Table S3: Associated data for grain trait QTLs in this study

Supplemental Table S4: Cereal genes involved in seed size control and their barley orthologs.

### Conflict of Interest Disclosure

The authors declare that there is no conflict of interest.

## Supporting information

Supplemental Information
Supplemental File 1

Supplemental Information
Supplemental File 2

Supplemental Information
Supplemental File 3

Supplemental Information
Supplemental File 4

Supplemental Information
Supplemental File 5

Supplemental Information
Supplemental File 6
