# Supplementary material for: Genome‐Wide Association Analysis of Grain Yield‐Associated Traits in a Pan‐European Barley Cultivar Collection
Source: Plant Genome. 2018 Mar 1;11(1):170073. doi: 10.3835/plantgenome2017.08.0073 (PMC12962562; doi:10.3835/plantgenome2017.08.0073)
Supplement: Supplementary file 1 [file TPG2-11-170073-s001.docx]

| **Supplemental Tables**  **Supplemental Table S1: Germplasm used in this study** | | | | |  | |  | |  |  | |  | |  | |
| --- | --- | --- | --- | --- | --- | --- | --- | --- | --- | --- | --- | --- | --- | --- | --- |
| **Reference Number** | **Name** | **Germplasm source** | **Accession** | **Habit** | | **Row type** | **Year** | **Country** | | | **Pedigree** | | **Breeder** | |  |
| EXB_0126 | Aapo | JIC | JIC 6588 | Spring | | 2-row | 1975 | Finland | | | Selection from X-ray treated TA B7790 [(Louhi x Opal) x Stallar II] | | Hankija | |  |
| EXB_0127 | Abacus | IPK | HOR 7488 | Spring | | 2-row | 1974 | United Kingdom | | | Vada x Zephyr | | Nickerson | |  |
| EXB_0128 | Abava |  |  | Spring | | 2-row | 1978 | Latvia | | | (Mari x Elsa) x Domen | | Stende Plant Breeding Station | |  |
| EXB_0131 | Akcent |  |  | Spring | | 2-row | 1992 | Czech Republic | | | Salome (HVS 827) x EP 79 | | Selgen Stupice | |  |
| EXB_0132 | Akka | IPK | HOR 19368 | Spring | | 2-row | 1970 | Sweden | | | (Arla)6 x Monte Cristo | | Weibull | |  |
| EXB_0134 | Alexis | IPK |  | Spring | | 2-row | 1986 | Germany | | | Breun 1622D x Triumph | | Breun | |  |
| EXB_0135 | Alis |  |  | Spring | | 2-row | 1985 | Denmark | | | Triumph x Rosie Abed | | Abed | |  |
| EXB_0136 | Alliot |  |  | Spring | | 2-row | 1999 | Denmark | | | Chariot x Alexis | | Pajbjergfonden | |  |
| EXB_0137 | Alva |  |  | Spring | | 2-row | 1979 | Sweden | | | Balder x (Binder x Seger x Vada) | | Svalof | |  |
| EXB_0138 | Ametyst |  |  | Spring | | 2-row | 1972 | Czech Republic | | | Domen x (Valticky x Hanacky jubilejni) x (Voldagsen x Diamant) | | Hrubcice | |  |
| EXB_0140 | Annabell |  |  | Spring | | 2-row | 1999 | Germany | | | Henni x Krona | | Nordsaat/Ackermann | |  |
| EXB_0141 | Anni |  |  | **Spring** | | 2-row | 1980 | Estonia | | | Lola x Liisa | | Jogeva Plant Breeding Institute | |  |
| EXB_0142 | Ansis |  |  | **Spring** | | 2-row | 1996 | Latvia | | | Jarek x Taifun | | Stende Plant Breeding Station | |  |
| EXB_0143 | Apex | SCRI |  | **Spring** | | 2-row | 1984 | Netherlands | | | Aramir x F1[CEB 6721 x (Julia3 x Volla x L100)] | | Cebeco | |  |
| EXB_0144 | Aramir | SCRI |  | **Spring** | | 2-row | 1974 | Netherlands | | | Volla x Emir | | Cebeco | |  |
| EXB_0146 | Armelle | SCRI |  | **Spring** | | 2-row | 1974 | France | | | Ceres x Clermont | | INRA | |  |
| EXB_0149 | Arvo | IPK | HOR 4736 | Spring | | 2-row | 1966 | Finland | | | Balder x Helmi | | Jokioinen | |  |
| EXB_0152 | Atem | SCRI |  | Spring | | 2-row | 1980 | Netherlands | | | ((L 92 x Minerva) x Emir) x Zephyr | | Wiersum | |  |
| EXB_0153 | Athos | SCRI |  | Spring | | 2-row | 1975 | France | | | Line 207 x Emir | | Desprez | |  |
| EXB_0154 | Atlas |  |  | Spring | | 2-row | 1976 | Czech Republic | | | Mutant SS 55 x Diamant | | Selgen Stupice | |  |
| EXB_0155 | Atribut |  |  | Spring | | 2-row | 1996 | Czech Republic | | | KM V 3/83 x BR 2174 | | Selgen Stupice | |  |
| EXB_0156 | Auriga |  |  | Spring | | 2-row | 2002 | Germany | | | (Viskosa x Krona) x Annabell | | Ackermann | |  |
| EXB_0157 | Balder | IPK | HOR 2490 | Spring | | 2-row | 1942 | Sweden | | | (Gull x Scanian Barley) x Maja | | Weibull | |  |
| EXB_0158 | Balder J | IPK | HOR 3872 | Spring | | 2-row | 1964 | Sweden | | | Balder X-Ray Mutant | | Weibull | |  |
| EXB_0159 | Balga |  |  | Spring | | 2-row | 1990 | Latvia | | | Gunilla x KM1192 | | APP Valsts Priekulu laukaugu selekcijas instituts | |  |
| EXB_0160 | Barabas |  |  | Spring | | 2-row | 2005 | Denmark | | | SJ 970621 x (Lux x Annabell) | | Sejet | |  |
| EXB_0162 | Barke | SCRI |  | Spring | | 2-row | 1996 | Germany | | | Libelle x Alexis | | Breun | |  |
| EXB_0163 | Baronesse |  |  | Spring | | 2-row | 1989 | Germany | | | ((343/6 x V34/6) x J-427) x (Oriol x LBW6153 P40) | | Nordsaat | |  |
| EXB_0164 | Beatrix |  |  | Spring | | 2-row | 2004 | Germany | | | Viskosa x Pasadena | | Nordsaat | |  |
| EXB_0165 | Berenice | IPK |  | Spring | | 2-row | 1972 | France | | | ((Union x (Bordia x Kenia))x Frisia | | INRA | |  |
| EXB_0166 | Binder | JIC |  | Spring | | 2-row | 1916 | Denmark | | | Selection from Hanna | | Abed | |  |
| EXB_0167 | Birgitta | JIC | Svalof 02338 | Spring | | 2-row | 1963 | Sweden | | | (Opal x Vega) x Maja | | Svalof | |  |
| EXB_0168 | Birka | IPK | HOR 13737 | Spring | | 2-row | 1981 | Sweden | | | Baladi16 x (Rika x ((Tellus x (Monte Christo x Tellus MD)) | | Weibull | |  |
| EXB_0169 | Blenheim | SCRI |  | Spring | | 2-row | 1987 | United Kingdom | | | Triumph x Egmont | | PBI | |  |
| EXB_0171 | Bonus | IPK | HOR 20829 | Spring | | 2-row | 1952 | Sweden | | | Maja X (Victory=Seger X Opal) | | Svalof | |  |
| EXB_0173 | Braemar |  |  | Spring | | 2-row | 2000 | United Kingdom | | | NFC 5563 x NFC 94.20 | | New Farm Crops (NFC) | |  |
| EXB_0174 | Brazil |  |  | Spring | | 2-row | 2001 | France | | | Trebon x Cooper | | Momont | |  |
| EXB_0176 | Britta | IPK | HOR 4485 | Spring | | 2-row | 1964 | Sweden | | | (Binder x Opal) x (Balder x Kenia) | | Lantmännen SW Seed | |  |
| EXB_0179 | Cabaret |  |  | Spring | | 2-row | 2004 | Denmark | | | Tumbler x Dialog | | Sejet | |  |
| EXB_0180 | Caja |  |  | Spring | | 2-row | 1980 | Denmark | | | PF-M-13 [(((Herta/KVL-191) x Ymer) x Rika)] x PF-62-6-6-4 | | Pajbjerg | |  |
| EXB_0181 | Calgary |  |  | Spring | | 2-row | 2002 | France | | | (Dominique x Blenheim) x (Barleta x Chapka) | | Serasem | |  |
| EXB_0182 | Cameo | JIC | RPB 80-1116 | Spring | | 2-row | 1985 | United Kingdom | | | RPB 713/77 x Kym | | Nickerson | |  |
| EXB_0183 | Caminant |  |  | Spring | | 2-row | 1994 | Denmark | | | Ant 484 (Grit) x Blenheim | | Sejet | |  |
| EXB_0184 | Camir |  |  | Spring | | 2-row | 1985 | Denmark | | | Triumph x Emir | | Carlsberg | |  |
| EXB_0187 | Carlsberg |  |  | Spring | | 2-row | 1947 | Denmark | | | Prentice x Maja | | Carlsberg | |  |
| EXB_0188 | Carvilla |  |  | Spring | | 2-row | 2004 | United Kingdom | | | (Cork x Newgrange) x Vortex | | New Farm Crops (NFC) | |  |
| EXB_0189 | Cellar | JIC | NFC 497-16 | Spring | | 2-row | 2001 | United Kingdom | | | (NFC 94.20 x Cork) NFC 94-11 | | New Farm Crops (NFC) | |  |
| EXB_0190 | Ceylon |  |  | Spring | | 2-row | 2003 | Netherlands | | | Portia x Amber | | Cebeco | |  |
| EXB_0191 | Chalice | JIC | NFC SB 94-7 | Spring | | 2-row | 1997 | United Kingdom | | | (Cooper x NFC 514-5) x Chariot | | New Farm Crops (NFC) | |  |
| EXB_0192 | Chamant |  |  | Spring | | 2-row | 1999 | Denmark | | | Ca 794891 x Krona | | Sejet | |  |
| EXB_0193 | Chanell |  |  | Spring | | 2-row | 2006 | Denmark | | | Barke x Ca 500201 | | Carlsberg | |  |
| EXB_0194 | Chariot | SCRI |  | Spring | | 2-row | 1992 | United Kingdom | | | Dera x (Carnival x Atem) | | PBI | |  |
| EXB_0195 | Cheri | IPK | HOR 18426 | Spring | | 2-row | 1987 | Germany | | | Triumph x (Medusa x Diamant) | | Bayer | |  |
| EXB_0026 | Chess | JIC | ICI 85-39-3 | Spring | | 2-row | 1995 | United Kingdom | | | Casino x Sherpa | | Limagrain (ICI Seeds) | |  |
| EXB_0197 | Cicero |  |  | Spring | | 2-row | 1999 | Denmark | | | Chalice x SJ 933275 | | Sejet | |  |
| EXB_0198 | Cilla |  |  | Spring | | 2-row | 1968 | Sweden | | | Ingrid x Hado Streng | | Weibull | |  |
| EXB_0199 | Clara (W5690) | JIC | JIC 7682 | Spring | | 2-row | 1964 | Sweden | | | Rika x (Balder x (Binder x Opal)) | | Weibull | |  |
| EXB_0200 | Claret | JIC | RPB 199-74 | Spring | | 2-row | 1980 | United Kingdom | | | ((Proctor x HP 5466) x Armelle) x Abacus | | Nickerson | |  |
| EXB_0201 | Class |  |  | Spring | | 2-row | 2003 | United Kingdom | | | Prestige x Optic | | PBI | |  |
| EXB_0202 | Claude |  |  | Spring | | 2-row | 2006 | Denmark | | | Annabell x Ca 800511 | | Carlsberg | |  |
| EXB_0203 | Cleopatra | IPK | HOR 20396 | Spring | | 2-row | 1991 | Austria | | | Beate x Kristall | | Saatbau | |  |
| EXB_0205 | Cooper | JIC | NFC 321/2 | Spring | | 2-row | 1994 | United Kingdom | | | (Corniche x Force) x Troop | | New Farm Crops (NFC) | |  |
| EXB_0206 | Corgi | JIC | 17693 Co 5 | Spring | | 2-row | 1983 | United Kingdom | | | Triumph x 15533 Co [((C.18-256 x Proctor) x Zephyr) x Julia)] | | Welsh Pl Breed Stn | |  |
| EXB_0207 | Corniche | SCRI |  | Spring | | 2-row | 1986 | Germany | | | (Diamant x 14029/64) x F2(Emir x (HOR 3270 x 46132/68)) | | Hadmersleben | |  |
| EXB_0208 | Cristalia |  |  | Spring | | 2-row | 2004 | United Kingdom | | | Ortoli x Brise | | Syngenta | |  |
| EXB_0209 | Croydon | JIC | Weibull 6855 | Spring | | 2-row | 1982 | Sweden | | | Welam x Tellus mutant | | Weibull | |  |
| EXB_0210 | Dandy | JIC | 18734 Co 95 | Spring | | 2-row | 1988 | United Kingdom | | | Egmont x Atem | | Welsh Pl Breed Stn | |  |
| EXB_0211 | Danuta |  |  | Spring | | 2-row | 2000 | Germany | | | 90014DH [Krona] x (Salome x Maresi) | | Nordsaat | |  |
| EXB_0212 | Deba Abed |  |  | Spring | | 2-row | 1965 | Denmark | | | Abed Denso x Weihenstephaner MR 2 | | Abed | |  |
| EXB_0214 | Derkado | SCRI |  | Spring | | 2-row | 1987 | Germany | | | Lada x Salome | | Hadmersleben | |  |
| EXB_0215 | Dialog |  |  | Spring | | 2-row | 2000 | Denmark | | | Otira x (Ferment x Mentor) | | Sejet | |  |
| EXB_0216 | Diamant | SCRI |  | Spring | | 2-row | 1965 | Czech Republic | | | Valticky mutant | | OGZ | |  |
| EXB_0217 | Digersano |  |  | Spring | | 2-row | 1991 | Italy | | | Mari-Coho x Sul-Mackta | | Cermis/ENEA | |  |
| EXB_0218 | Dina |  |  | Spring | | 2-row | 1975 | Denmark | | | Amsel x Deba | | Abed | |  |
| EXB_0220 | Domen | IPK | HOR 2494 | Spring | | 2-row | 1952 | Norway | | | Opal B x Maskin | | Moystadt | |  |
| EXB_0221 | Doublet | JIC | 17920 Co H26 | Spring | | 2-row | 1985 | United Kingdom | | | Triumph x Goldspear | | Welsh Pl Breed Stn | |  |
| EXB_0222 | Drake | JIC | JIC 8125 | Spring | | 2-row | 1968 | Netherlands | | | Volla x Abed 3371 | | MGH | |  |
| EXB_0223 | Drost |  |  | Spring | | 2-row | 1954 | Denmark | | | Maja x Kenia | | Pajbjerg | |  |
| EXB_0228 | Egmont | IPK | HOR 8890 | Spring | | 2-row | 1980 | United Kingdom | | | (Maris Yak x Weib. 1001 2) x Vada | | PBI | |  |
| EXB_0229 | Elantra |  |  | Spring | | 2-row | 1998 | Denmark | | | Caminant x Heron | | Sejet | |  |
| EXB_0230 | Elo |  |  | Spring | | 2-row | 1989 | Estonia | | | Triumph x Lofa | | Jogeva Plant Breeding Institute | |  |
| EXB_0231 | Emir | JIC |  | Spring | | 2-row | 1962 | Netherlands | | | Delta x (Agio x (Kenia*3 x Arabische)) | | Cebeco | |  |
| EXB_0235 | Eunova |  |  | Spring | | 2-row | 1998 | Austria | | | (Serva x ML502) x CF 79 | | Probstdorf | |  |
| EXB_0237 | Famin |  |  | Spring | | 2-row | 1996 | Czech Republic | | | ST 145 (Akcent) x CE 597 | | Hrubcice | |  |
| EXB_0238 | Favorit |  |  | Spring | | 2-row | 1973 | Czech Republic | | | Diamant x Firlb. Union | | Hrubcice | |  |
| EXB_0239 | Felicitas |  |  | Spring | | 2-row | 2002 | Germany | | | (Baronesse x Meltan) x Krona) | | Breun | |  |
| EXB_0240 | Formula |  |  | Spring | | 2-row | 1987 | Sweden | | | Triumph x A 11 3109 | | Weibull | |  |
| EXB_0241 | Forum |  |  | Spring | | 2-row | 1993 | Czech Republic | | | (H 387/75 x Horpadsi Ketsoras) x KM0 44/78 | | HYBRITECH | |  |
| EXB_0242 | Freja | IPK | HOR 20460 | Spring | | 2-row | 1941 | Sweden | | | Seger x Opal | | Svalof | |  |
| EXB_0245 | Galan |  |  | Spring | | 2-row | 1990 | Slovak Republic | | | SK 2567 x HE 1428 | | Sladkovicovo | |  |
| EXB_0246 | Gant |  |  | Spring | | 2-row | 1997 | Denmark | | | Ant 499 x Alexis | | Sejet | |  |
| EXB_0247 | Gate |  |  | Spring | | 2-row | 1995 | Latvia | | | (H497 x Hadmersleben) x (Nadja*2 x Emir) | | Priekuli | |  |
| EXB_0248 | Gerkra | JIC | Kraai 612 | Spring | | 2-row | 1970 | Netherlands | | | Volla x Proctor | | Kraai | |  |
| EXB_0249 | Gitane |  |  | Spring | | 2-row | 1976 | Netherlands | | | Zephyr x (C I 1237 x Cambrinus) | | Wiersum | |  |
| EXB_0250 | Gizmo |  |  | Spring | | 2-row | 2006 | Denmark | | | Prestige x Ca 800602 | | Carlsberg | |  |
| EXB_0251 | Golden Promise | SCRI |  | Spring | | 2-row | 1968 | United Kingdom | | | Maythorpe Gamma-Ray Mutant | | Miln Marsters | |  |
| EXB_0253 | Golf | IPK |  | Spring | | 2-row | 1983 | United Kingdom | | | (Armelle x Lud) x Luke | | Nickerson | |  |
| EXB_0254 | Gorm |  |  | Spring | | 2-row | 1981 | Denmark | | | ((Herta*8 x Byg 191) x Ingrid) x Minerva) x Kristina | | Sejet | |  |
| EXB_0255 | Gull |  |  | Spring | | 2-row | 1915 | Sweden | | | Landrace selection from Gotland | | Svalof | |  |
| EXB_0257 | Hana |  |  | Spring | | 2-row | 1973 | Czech Republic | | | Diamant x Alsa | | OGZ | |  |
| EXB_0258 | Hanka |  |  | Spring | | 2-row | 1997 | Germany | | | Hadm.59473-85 x Hadm.96677-87 (from Krona) | | Semundo | |  |
| EXB_0260 | Hanna | JIC | Weibull 2733 68 | Spring | | 2-row | 1977 | Sweden | | | (Monte Christo x Clara) x (W 5793 x W 5853) | | Weibull | |  |
| EXB_0051 | Harry | IPK |  | Spring | | 2-row | 1978 | Sweden | | | Arla M1 x Tellus | | Weibull | |  |
| EXB_0263 | Hassan | IPK | HOR 12141 | Spring | | 2-row | 1971 | Netherlands | | | Delta x (Agio x (Kenia x Arabische)) | | Cebeco | |  |
| EXB_0266 | Hellas | IPK |  | Spring | | 2-row | 1967 | Sweden | | | Herta x Pallas | | Svalof | |  |
| EXB_0267 | Helmi | IPK |  | Spring | | 2-row | 1942 | Finland | | | Binder x Pikkio | | na | |  |
| EXB_0268 | Heris |  |  | Spring | | 2-row | 1998 | Czech Republic | | | HE 4436 x CE 431 | | Hrubcice | |  |
| EXB_0271 | Hydrogen |  |  | Spring | | 2-row | 1999 | Denmark | | | (Alis x Digger) x Derkado | | Nordic seed | |  |
| EXB_0272 | Ida | NGB |  | Spring | | 2-row | 1980 | Sweden | | | Arla M1 x Tellus | | Weibull | |  |
| EXB_0273 | Idumeja |  |  | Spring | | 2-row | 1998 | Latvia | | | Imula x Ida | | Priekuli | |  |
| EXB_0274 | Ilga |  |  | Spring | | 2-row | 1983 | Latvia | | | KM-1192 x Hadmersleben 70197/70 | | na | |  |
| EXB_0275 | Imber | IPK | HOR 16648 | Spring | | 2-row | 1970 | United Kingdom | | | Elsa x Proctor | | Guinness | |  |
| EXB_0276 | Imidis |  |  | Spring | | 2-row | 2006 | Denmark | | | Fabel x (Vortex x SJ 203256) | | Sejet | |  |
| EXB_0277 | Impala | IPK |  | Spring | | 2-row | 1964 | Netherlands | | | (Wisa x Balder) x (Haisa x Imperial x Hord.sp.204) | | MGH | |  |
| EXB_0278 | Imula |  |  | Spring | | 2-row | 1985 | Latvia | | | Abavax Akka*2 | | Stende Plant Breeding Station | |  |
| EXB_0279 | Inari |  |  | Spring | | 2-row | 1994 | Finland | | | (Herta x Jo 1490) xTriumph | | Boreal Plant Breeding Ltd | |  |
| EXB_0281 | Ingrid | SCRI |  | Spring | | 2-row | 1956 | Sweden | | | Balder x (Binder x Opal) | | Weibull | |  |
| EXB_0282 | Isabella |  |  | Spring | | 2-row | 2005 | Denmark | | | Power x Recept | | Sejet | |  |
| EXB_0283 | Isaria | SCRI |  | Spring | | 2-row | 1924 | Germany | | | Bavaria x Danubia | | Ackermann | |  |
| EXB_0285 | Jarek |  |  | Spring | | 2-row | 1987 | Czech Republic | | | (Km 1192 x Sladar) x Opal | | Kromeriz | |  |
| EXB_0293 | Karat |  |  | Spring | | 2-row | 1981 | Czech Republic | | | (Valticky x B2145) x (Carlsberg x F.Union x Diamant x 1293/70) | | Kromeriz | |  |
| EXB_0294 | Karri | IPK | HOR 4734 | Spring | | 2-row | 1967 | Finland | | | Carlsberg x Rigel | | TAB | |  |
| EXB_0295 | Kenia | SCRI |  | Spring | | 2-row | 1931 | Denmark | | | Binder x Gull | | Abed | |  |
| EXB_0296 | Keops |  |  | Spring | | 2-row | 2006 | Denmark | | | Simba x Power | | Sejet | |  |
| EXB_0300 | Koral |  |  | Spring | | 2-row | 1980 | Czech Republic | | | Hana x (((Carlsb.II x Union) x Alsa) x (Hanacky x Imune 25)) | | Hrubcice | |  |
| EXB_0301 | Kristaps |  |  | Spring | | 2-row | 2002 | Latvia | | | CF 79502 x STN9023 | | Stende Plant Breeding Station | |  |
| EXB_0304 | Krystal |  |  | Spring | | 2-row | 1981 | Czech Republic | | | Koral x Rapid | | Hrubcice | |  |
| EXB_0305 | Ladik |  |  | Spring | | 2-row | 1992 | Czech Republic | | | KM 499-293-80 x Dera (HVS 1461) | | Kromeriz | |  |
| EXB_0306 | Latvijas Vietejie |  |  | Spring | | 2-row | na | Latvia | | | Latvian local landrace | | na | |  |
| EXB_0307 | Leeni |  |  | Spring | | 2-row | 2006 | Estonia | | | (Ingrid x Golden Promise) x Decor | | Jogeva Plant Breeding Institute | |  |
| EXB_0308 | Lenta | JIC |  | Spring | | 2-row | 1947 | Denmark | | | Maja x Kenia | | Carlsberg | |  |
| EXB_0309 | Linga |  |  | Spring | | 2-row | 1985 | Latvia | | | Gunilla x KM 1192 | | Priekulu | |  |
| EXB_0311 | Lofa Abed | IPK | HOR 12142 | Spring | | 2-row | 1970 | Denmark | | | Proctor x Minerva | | Abed | |  |
| EXB_0313 | Lud | IPK | HOR 7292 | Spring | | 2-row | 1976 | United Kingdom | | | (Vada x Zephyr) x RMGH 59114 | | Nickerson | |  |
| EXB_0315 | Lux |  |  | Spring | | 2-row | 1997 | Denmark | | | Goldie x Cork | | Sejet | |  |
| EXB_0316 | Lysiba |  |  | Spring | | 2-row | 1998 | Denmark | | | Lamba x SJ 900691 | | Sejet | |  |
| EXB_0317 | Lysimax |  |  | Spring | | 2-row | 1994 | Denmark | | | Ca 040223 x Carula | | Sejet | |  |
| EXB_0319 | Maja | IPK |  | Spring | | 2-row | 1927 | Denmark | | | Binder x Gull | | Abed | |  |
| EXB_0320 | Mala | IPK | HOR 15000 | Spring | | 2-row | 1972 | Denmark | | | Proctor x Minerva | | Abed | |  |
| EXB_0321 | Malva |  |  | Spring | | 2-row | 2001 | Latvia | | | Stende 8142 x Stende 7542 | | Latvian University of Agriculture | |  |
| EXB_0322 | Maresi |  |  | Spring | | 2-row | 1986 | Germany | | | (Cebeco 6801 x GB 1605) x 46459/68 | | Bernburg/Sacon | |  |
| EXB_0325 | Maris Mink | IPK |  | Spring | | 2-row | 1973 | United Kingdom | | | (Emir x Swallow) x Deba Abed | | PBI | |  |
| EXB_0326 | Mars |  |  | Spring | | 2-row | 1983 | Czech Republic | | | (St 13259 x Hor 2957) x (Abed Lofa) | | Stupice | |  |
| EXB_0327 | Marthe |  |  | Spring | | 2-row | 2005 | Germany | | | Neruda x Recept | | Nordsaat | |  |
| EXB_0329 | Mauritia |  |  | Spring | | 2-row | 2004 | Germany | | | Madras x LP 217.96 | | Lochow-Petkus | |  |
| EXB_0330 | Meltan | SCRI |  | Spring | | 2-row | 1991 | Sweden | | | (Triumph x Georgie) x Tellus MMMDDN | | Svalof | |  |
| EXB_0331 | Mentor |  |  | Spring | | 2-row | 1985 | Netherlands | | | (Trumpf x Cambrinus) x Piccolo | | Wiersum | |  |
| EXB_0332 | Midas | IPK |  | Spring | | 2-row | 1970 | United Kingdom | | | ((Proctor x Wong) x Mildew Resistant A) x Maythorpe Gamma-Ray Mutant | | Miln Marsters | |  |
| EXB_0334 | Mona |  |  | Spring | | 2-row | 1971 | Sweden | | | Mari x Monte Cristo | | Svalof | |  |
| EXB_0337 | Nathalie |  |  | Spring | | 2-row | 2006 | Denmark | | | Alexandra x (Power x Recept) | | Sejet | |  |
| EXB_0338 | Nemex | IPK | HOR 18266 | Spring | | 2-row | 1985 | Sweden | | | (Ingrid M x Ansgar) x (Tellus x Stamm) | | Weibull | |  |
| EXB_0341 | Nordal |  |  | Spring | | 2-row | 1971 | Denmark | | | Heine 4808 x Dana | | Carlsberg | |  |
| EXB_0342 | Novum |  |  | Spring | | 2-row | 1988 | Slovak Republic | | | Sk 1429 x Krystal | | Sladkovicovo | |  |
| EXB_0344 | Odessa |  |  | Spring | | 2-row | 2003 | Germany | | | 4908d x (Volga x Penelope) | | Breun | |  |
| EXB_0345 | Odin |  |  | Spring | | 2-row | 1981 | Denmark | | | SV-665433 x (Ingrid x Proctor) | | Carlsberg | |  |
| EXB_0346 | Okos |  |  | Spring | | 2-row | 1975 | Italy | | | (Otto x Stanka) x Otto | | na | |  |
| EXB_0348 | Optic | SCRI |  | Spring | | 2-row | 1995 | United Kingdom | | | Chad x (Corniche x Force) | | New Farm Crops (NFC) | |  |
| EXB_0349 | Orbit |  |  | Spring | | 2-row | 1986 | Slovak Republic | | | (Rtg.mutant Slovensky 802 x Sladar) x ((Diamant x Monte Christo) x (Valticky x Ekonom)) [SK 783 x CE CD/74] | | Sladkovicovo | |  |
| EXB_0350 | Orza |  |  | Spring | | 2-row | 1994 | Turkey | | | Tokak x Landrace 4857 | | Tarla Bitkileri Merkez Ars.Ens. Müd. | |  |
| EXB_0354 | Otto |  |  | Spring | | 2-row | 1972 | Italy | | | Genealogical selection from African population | | na | |  |
| EXB_0356 | Pallas | IPK |  | Spring | | 2-row | 1961 | Sweden | | | Bonus mutant | | Svalof | |  |
| EXB_0357 | Paloma |  |  | Spring | | 2-row | 1994 | Denmark | | | (Corgi x Alexis) x (Corgi x Alis) | | Abed | |  |
| EXB_0358 | Pasadena |  |  | Spring | | 2-row | 1998 | Germany | | | Marina x Krona | | Lochow-Petkus | |  |
| EXB_0359 | Perun |  |  | Spring | | 2-row | 1988 | Czech Republic | | | HE 1728 x Karat | | Hrubcice | |  |
| EXB_0364 | Power |  |  | Spring | | 2-row | 2003 | Denmark | | | Saloon x (Colada x (Lux x Annabell)) | | Sejet | |  |
| EXB_0368 | Priora | IPK | HOR 18795 | Spring | | 2-row | 2000 | Italy | | | Arda x Mondo | | Cermis/ENEA | |  |
| EXB_0371 | Proctor | JIC |  | Spring | | 2-row | 1953 | United Kingdom | | | Plumage Archer x Kenia | | PBI | |  |
| EXB_0374 | Prosa |  |  | Spring | | 2-row | 1996 | Austria | | | WW 7748 x Br. 3818 d 17 | | Probstdorf | |  |
| EXB_0375 | Publican |  |  | Spring | | 2-row | 2006 | United Kingdom | | | Drum x Sebastian | | Syngenta | |  |
| EXB_0377 | Quartz |  |  | Spring | | 2-row | 1992 | United Kingdom | | | NRPB 82-999 x Apex | | Nickerson | |  |
| EXB_0378 | Quench |  |  | Spring | | 2-row | 2006 | United Kingdom | | | Sebastian x Drum | | Syngenta | |  |
| EXB_0379 | Rapid |  |  | Spring | | 2-row | 1976 | Czech Republic | | | (Voldagsen x Kneifel) x (Diamant x Denar) | | Hrubcice | |  |
| EXB_0380 | Rasa |  |  | Spring | | 2-row | 1991 | Latvia | | | Frankengold x KM-HE-R-54/72 | | Stende Plant Breeding Station | |  |
| EXB_0381 | Rika | IPK |  | Spring | | 2-row | 1951 | Sweden | | | Kenia x Isaria | | Weibull | |  |
| EXB_0382 | Riviera | JIC | CSBA 1433-3717 | Spring | | 2-row | 1995 | United Kingdom | | | Stanza x Cebeco 8331 | | PBI | |  |
| EXB_0383 | Roland | IPK |  | Spring | | 2-row | 1982 | Sweden | | | Lud x Tellus M1D | | Weibull | |  |
| EXB_0385 | Romi |  |  | Spring | | 2-row | 1983 | Denmark | | | Abed 079 x Rupal | | Abed | |  |
| EXB_0388 | Roxana |  |  | Spring | | 2-row | 2000 | Germany | | | (Br st. 3556 e x Korinna) x Alexis | | Breun | |  |
| EXB_0389 | Rubin | IPK | BCC 1530 | Spring | | 2-row | 1982 | Czech Republic | | | (Valticky 2 x Algerien xValticky x Union) x (Diamant x Nadja) | | Hrubcice | |  |
| EXB_0390 | Ruja |  |  | Spring | | 2-row | 1992 | Latvia | | | Abava x (Kombainieris x Triumph) | | Priekuli | |  |
| EXB_0391 | Rupal | NGB |  | Spring | | 2-row | 1972 | Sweden | | | Pallas*3 x Rupee | | Svalof | |  |
| EXB_0392 | Saana |  |  | Spring | | 2-row | 1996 | Finland | | | Kustaa x (Olli x Eero) | | Boreal Plant Breeding Ltd | |  |
| EXB_0393 | Safir |  |  | Spring | | 2-row | 1978 | Czech Republic | | | (Valticky x Kneifel) x (Diamant x arabische Zweizeilige) | | Hrubcice | |  |
| EXB_0394 | Salka |  |  | Spring | | 2-row | 1973 | Denmark | | | Elbo x Vada | | Pajbjerg | |  |
| EXB_0395 | Salve | IPK | HOR 7310 | Spring | | 2-row | 1975 | Sweden | | | Birgitta x Mari | | Svalof | |  |
| EXB_0107 | Sarah | IPK |  | Spring | | 2-row | 1974 | France | | | Selection from Champagne | | na | |  |
| EXB_0396 | Scandium |  |  | Spring | | 2-row | 2005 | Denmark | | | Roxana x Delibes | | Nordic seed | |  |
| EXB_0397 | Scarlett | SCRI |  | Spring | | 2-row | 1996 | Germany | | | (Amazone x Lignee 2730E) x Kym | | Breun | |  |
| EXB_0399 | Senat | IPK | HOR 7311 | Spring | | 2-row | 1974 | Sweden | | | (Hellas x Pallas*3) x Triple awn Lemma | | Svalof | |  |
| EXB_0400 | Sencis |  |  | Spring | | 2-row | 1995 | Latvia | | | (Rupal x Ofir) x Torkel | | Stende Plant Breeding Station | |  |
| EXB_0403 | Simba |  |  | Spring | | 2-row | 2003 | Denmark | | | Otira x Prolog | | Sejet | |  |
| EXB_0404 | Simon | JIC | WR 73294 | Spring | | 2-row | 1978 | Sweden | | | Tellus M1D | | Weibull | |  |
| EXB_0406 | Smilla |  |  | Spring | | 2-row | 2004 | Denmark | | | Tumbler x Dialog | | Sejet | |  |
| EXB_0407 | Spartan |  |  | Spring | | 2-row | 1977 | Czech Republic | | | Diamant x ((Monte-Cristo x Valticky) x Ekonom) | | Hrubcice | |  |
| EXB_0408 | Static | JIC | NFC SB 495-18 | Spring | | 2-row | 1999 | United Kingdom | | | Landlord x Cork | | New Farm Crops (NFC) | |  |
| EXB_0409 | Steffi |  |  | Spring | | 2-row | 1989 | Germany | | | (Stamm 101 x Aramir) x Stamm 210 | | Ackermann | |  |
| EXB_0410 | Steina | JIC |  | Spring | | 2-row | 1980 | Germany | | | (Sultan x St.434/62) x (Voldagsen x Carlsberg x Volla) | | Breun | |  |
| EXB_0412 | Stendes |  |  | Spring | | 2-row | 1972 | Latvia | | | Drost x Maja | | Stende Plant Breeding Station | |  |
| EXB_0413 | Sultan | IPK |  | Spring | | 2-row | 1968 | Netherlands | | | Delta x (Agio x (Kenia*3 x Arabische)) | | Cebeco | |  |
| EXB_0414 | Sundance |  |  | Spring | | 2-row | 1976 | United Kingdom | | | Vada x Zephyr | | Nickerson | |  |
| EXB_0417 | Tarm92 |  |  | Spring | | 2-row | 1993 | Turkey | | | Tokak x Landrace 4875 | | FCCRI-A | |  |
| EXB_0114 | Tea |  |  | Spring | | 2-row | 1988 | Italy | | | IS MV-41 x IS Q 10 | | #N/A | |  |
| EXB_0420 | Tellus | IPK |  | Spring | | 2-row | 1970 | Sweden | | | Strengs Franken III x (Balder x (Binder x Opal)) | | Weibull | |  |
| EXB_0421 | Terno |  |  | Spring | | 2-row | 1991 | Czech Republic | | | KM-S-170-74/Opal | | Branisovice | |  |
| EXB_0422 | Thuringia |  |  | Spring | | 2-row | 1995 | Germany | | | (Steffi x Gerlinde) x (243/4 x Salome) | | Ackermann | |  |
| EXB_0339 | Tipple | SCRI |  | Spring | | 2-row | 2005 | United Kingdom | | | (NFC 497-12 x Cork) x Vortex | | New Farm Crops (NFC) | |  |
| EXB_0424 | Tocada |  |  | Spring | | 2-row | 2003 | Germany | | | Pasadena x Henni | | Lochow-Petkus | |  |
| EXB_0425 | Tremois | SCRI |  | Spring | | 2-row | 1989 | France | | | (Dram x Aramir) x Berac | | Verneuil | |  |
| EXB_0426 | Triumph | SCRI |  | Spring | | 2-row | 1973 | Germany | | | Diamant x St. 14024-64/6 [((Alsa x S3170/Abyss) x 11719/59) x Union] | | Hadmersleben | |  |
| EXB_0428 | Tyra | IPK | BCC 1418 | Spring | | 2-row | 1975 | Denmark | | | (Algerian x Herta*8) x (Rika x Drost) | | Pajbjerg | |  |
| EXB_0429 | Union | IPK |  | Spring | | 2-row | 1955 | Germany | | | (Weihenstephaner 6831 x Donaria) x Firlbecks 621 | | Firlbeck | |  |
| EXB_0256 | Unknown 3 | JIC | JIC 3643 | Spring | | 2-row |  |  | | |  | |  | |  |
| EXB_0430 | Ursel | JIC |  | Spring | | 2-row | 1985 | Germany | | | Aramir x Trumpf | | Firlbeck | |  |
| EXB_0431 | Vada | JIC |  | Spring | | 2-row | 1958 | Netherlands | | | H.laevigatum x Gull | | SVP Wageningen | |  |
| EXB_0433 | Valticky | JIC |  | Spring | | 2-row | 1950 | Czech Republic | | | Valticky B x Starnovsky Kneifel | | Valtice | |  |
| EXB_0434 | Vankkuri | IPK |  | Spring | | 2-row |  | Finland | | | Selection from Landrace Vancouver | | na | |  |
| EXB_0436 | Vega |  |  | Spring | | 2-row | 1977 | Denmark | | | Kristina x Lofa | | Abed | |  |
| EXB_0438 | Viivi |  |  | Spring | | 2-row | 1994 | Finland | | | (Aapo x Berenice) x Ida | | Boreal Plant Breeding Ltd | |  |
| EXB_0439 | Volla | JIC |  | Spring | | 2-row | 1957 | Germany | | | Wisa x Haisa | | Breun | |  |
| EXB_0440 | Welam | IPK |  | Spring | | 2-row | 1976 | Sweden | | | ((Monte-Christo x Clara) x WW-5793*2) x WW-5853*3 | | Weibull | |  |
| EXB_0441 | Wing | IPK |  | Spring | | 2-row | 1968 | Sweden | | | Lyallpur x Ingrid*6 | | Weibull | |  |
| EXB_0442 | Wisa | JIC |  | Spring | | 2-row | 1951 | Germany | | | Weihenstephaner Mehltauresistente 1 x Isaria | | Breun | |  |
| EXB_0443 | Zenit |  |  | Spring | | 2-row | 1985 | Czech Republic | | | (Valticky x KM-1402) x Karat | | Kromeriz | |  |
| EXB_0444 | Zephyr | SCRI |  | Spring | | 2-row | 1966 | Netherlands | | | Heine 2149 x Carlsberg | | MGH | |  |
| EXB_0129 | Agneta | IPK |  | Spring | | 6-row | 1981 | Sweden | | | (Asa x Frisia) x (Edda II*4 x Monte Christo) | | Svalof | |  |
| EXB_0147 | Arra |  |  | Spring | | 6-row | 1982 | Finland | | | Varde x Otra | | Boreal Plant Breeding Ltd | |  |
| EXB_0148 | Artturi |  |  | Spring | | 6-row | 1992 | Finland | | | Arra x Nord | | Boreal Plant Breeding Ltd | |  |
| EXB_0150 | Asplund |  |  | Spring | | 6-row | 1910 | Sweden | | | Swedish landrace | | na | |  |
| EXB_0172 | Botnia |  |  | Spring | | 6-row | 1996 | Finland | | | Silja x Jo 1182 | | Boreal Plant Breeding Ltd | |  |
| EXB_0224 | Druvis |  |  | Spring | | 6-row | 1999 | Latvia | | | Dobrij x HVS 115440 | | Stende Plant Breeding Station | |  |
| EXB_0226 | Edda | IPK |  | Spring | | 6-row | 1949 | Sweden | | | Vega x Asplund | | Svalof | |  |
| EXB_0227 | Eero |  |  | Spring | | 6-row | 1975 | Finland | | | Mari x Otra | | Hankija | |  |
| EXB_0232 | Erkki |  |  | Spring | | 6-row | 1998 | Finland | | | Pohto x Potra | | Boreal Plant Breeding Ltd | |  |
| EXB_0234 | Etu | IPK |  | Spring | | 6-row | 1970 | Finland | | | Bonus M x Varde | | Boreal Plant Breeding Ltd | |  |
| EXB_0243 | Frisia | IPK | HOR 15140 | Spring | | 6-row | 1955 | Germany | | | (Granat-BreuStedt x Pyrthyjärven) x (Eckendorfer (WG) x Kalkreuther (WG)) | | Breustedt | |  |
| EXB_0259 | Hankkija 673 |  |  | Spring | | 6-row | 1973 | Finland | | | Otra x Paavo | | Hankija | |  |
| EXB_0269 | Herse | IPK |  | Spring | | 6-row | 1939 | Norway | | | Asplund x Maskin | | Vollebekk | |  |
| EXB_0284 | Jadar | IPK | HOR 20843 | Spring | | 6-row | 1933 | Norway | | | selection from landvariety originating in Jaerbygg | | na | |  |
| EXB_0286 | Jarle | NGB |  | Spring | | 6-row | 1960 | Norway | | | Jadar x (Asplund x Maskin) | | Vollebekk | |  |
| EXB_0291 | Jyvä |  |  | Spring | | 6-row | 2000 | Finland | | | Arve/Verner | | Boreal Plant Breeding Ltd | |  |
| EXB_0292 | Kajsa |  |  | Spring | | 6-row | 1977 | Finland | | | ((Asa x Frisia) x Edda II*4) x Monte Cristo | | Svalof | |  |
| EXB_0297 | Kilta |  |  | Spring | | 6-row | 1981 | Finland | | | (Seger x Vega) x Bonus | | Boreal Plant Breeding Ltd | |  |
| EXB_0310 | Lise | IPK | BCC 1527 | Spring | | 6-row | 1964 | Norway | | | (DS 295 X Asplund) x Varde | | Vollebekk | |  |
| EXB_0312 | Loviisa |  |  | Spring | | 6-row | 1989 | Finland | | | Potra x (Eero x Pomo) | | Boreal Plant Breeding Ltd | |  |
| EXB_0328 | Maskin | IPK | BCC 1528 | Spring | | 6-row | 1918 | Norway | | | Selection from Bjoernebybygg (east Norway) Landrace | | na | |  |
| EXB_0451 | Morex |  |  | Spring | | 6-row | 1978 | USA | | | Cree x Bonanza | | University of Minnesota | |  |
| EXB_0340 | Niina | NGB |  | Spring | | 6-row | 1986 | Finland | | | Varde x Otra | | Boreal Plant Breeding Ltd | |  |
| EXB_0347 | Olli | IPK |  | Spring | | 6-row | 1927 | Finland | | | Finnish landrace selection | | na | |  |
| EXB_0353 | Otra | IPK3 |  | Spring | | 6-row | 1959 | Finland | | | Edda x Tammi | | Hankija | |  |
| EXB_0355 | Paavo | IPK | HOR 3873 | Spring | | 6-row | 1960 | Finland | | | (OAC 21 x Gull) x Tammi | | Jokioinen | |  |
| EXB_0099 | Pirkka | IPK |  | Spring | | 6-row | 1952 | Finland | | | (Maskin x Finnish Variety) x (Olli x Manchurian Barley) | | Tammisto | |  |
| EXB_0360 | Pohto |  |  | Spring | | 6-row | 1987 | Finland | | | Hankkija 673 x (Hankkija 673 x Hiproly) | | Hankija | |  |
| EXB_0361 | Pokko | NGB |  | Spring | | 6-row | 1980 | Finland | | | Pirka x Pomo*2 | | Hankija | |  |
| EXB_0362 | Pomo | IPK |  | Spring | | 6-row | 1968 | Finland | | | (Seger x Vega) x 6zlg.Linie x Bonus | | Hankija | |  |
| EXB_0363 | Potra |  |  | Spring | | 6-row | 1983 | Finland | | | Otra x ( Andie x Otra) | | Hankija | |  |
| EXB_0386 | Rondo | IPK | BCC 1343 | Spring | | 6-row | 1976 | Italy | | | Tanaroo x Sisfor L. 90 | | SIS | |  |
| EXB_0402 | Silja |  |  | Spring | | 6-row | 1979 | Finland | | | (Seger x Wega) x Suvi | | Jokioinen | |  |
| EXB_0411 | Stella | IPK |  | Spring | | 6-row |  | Sweden | | | Selection from Halsingland landrace | | Svalof? | |  |
| EXB_0450 | Steptoe |  |  | Spring | | 6-row | 1973 | USA | | | Washington Sel. 3564 x Unitan | | Washington State University | |  |
| EXB_0415 | Suvi | IPK | HOR 21240 | Spring | | 6-row | 1973 | Finland | | | Tammi x (Gull x OAC 21) | | Jokioinen | |  |
| EXB_0416 | Tammi | IPK |  | Spring | | 6-row | 1937 | Finland | | | Olli X Asplund | | Tammisto | |  |
| EXB_0418 | Teele |  |  | Spring | | 6-row | 1990 | Estonia | | | Otra DMSO mutant | | Jogeva Plant Breeding Institute | |  |
| EXB_0419 | Teemu |  |  | Spring | | 6-row | 1976 | Finland | | | (Vankkuri x Tammi) x Suvi | | Boreal Plant Breeding Ltd | |  |
| EXB_0145 | Unknown 1 | IPK | HOR 6884 | Spring | | 6-row |  |  | | |  | |  | |  |
| EXB_0170 | Unknown 2 |  |  | Spring | | 6-row |  |  | | |  | |  | |  |
| EXB_0303 | Unknown 4 | IPK | HOR 7301 | Spring | | 6-row |  |  | | |  | |  | |  |
| EXB_0432 | Vairogs |  |  | Spring | | 6-row | 1930 | Latvia | | | Selection from Priekulu local landrace | | na | |  |
| EXB_0435 | Varde | IPK |  | Spring | | 6-row | 1941 | Norway | | | Maskin x Asplund | | Vidarshov | |  |
| EXB_0001 | Aci |  |  | Winter | | 2-row | 1994 | Italy | | | [Alpha x (Alpha x Sonja)]x[Capri x (Okos x 273cat.)] | | CRA-Acireale | |  |
| EXB_0002 | Aiace |  |  | Winter | | 2-row | 2002 | Italy | | | (Opale x HJ54111 x Alpha x Tipper x Alpha) x Katy x HJ54-30 x Igri x Arda x Baraka | | CRA-Fiorenzuola | |  |
| EXB_0003 | Airone |  |  | Winter | | 2-row | 1999 | Italy | | | (Gitane x Tipper) x Arda | | CRA-Fiorenzuola | |  |
| EXB_0004 | Alce |  |  | Winter | | 2-row | 2004 | Italy | | | (Tipper x Igri x Igri x Igri) x Tipper x Alpha x Sonia x W117118 | | CRA-Fiorenzuola | |  |
| EXB_0006 | Alfeo |  |  | Winter | | 2-row | 1993 | Italy | | | Tipper x Igri | | CRA-Fiorenzuola | |  |
| EXB_0008 | Amillis |  |  | Winter | | 2-row | 1995 | France | | | Baraka x Mosar | | Verneuil | |  |
| EXB_0010 | Aquarelle |  |  | Winter | | 2-row | 2003 | Germany | | | Regina x BAU 623/94 | | Lochow-Petkus | |  |
| EXB_0011 | Arda | IPK | BCC 1303 | Winter | | 2-row | 1985 | Italy | | | Igri x HJ 51-15-3 | | CRA-Fiorenzuola | |  |
| EXB_0014 | Asso |  |  | Winter | | 2-row | 1993 | Italy | | | Barberousse x Tipper | | BORASIO ERCOLE | |  |
| EXB_0017 | Aydanhanim |  |  | Winter | | 2-row | 2002 | Turkey | | | GK Omega x Tarm | | FCCRI-A | |  |
| EXB_0020 | Baraka |  |  | Winter | | 2-row | 1986 | France | | | [(Midas x Ribari 33) x Alpha] x Barberousse | | Serasem | |  |
| EXB_0023 | Carat | JIC |  | Winter | | 2-row | 2002 | United Kingdom | | | Volley x Cabrio | | Nickerson | |  |
| EXB_0027 | Clara | IPK | HOR 4567 | Winter | | 2-row | 2001 | Germany | | | Babylone x Anthere | | Nordsaat | |  |
| EXB_0028 | Clarine | IPK | HOR 10864 | Winter | | 2-row | 1988 | France | | | Igri x Mogador | | Secobra | |  |
| EXB_0029 | Dasio |  |  | Winter | | 2-row | 1999 | Italy | | | Tipper x Arupos | | CRA-Foggia | |  |
| EXB_0031 | Diadem | IPK | HOR 20041 | Winter | | 2-row | 1999 | France | | | 29194 x Marianne | | Serasem | |  |
| EXB_0032 | Dolmen |  |  | Winter | | 2-row | 2001 | France | | | Labea x MH 387 | | Momont | |  |
| EXB_0033 | Duchess |  |  | Winter | | 2-row | 1996 | France | | | Sonja x Barberousse | | Lemaire | |  |
| EXB_0034 | Duet | JIC | NRPB 88-5358 | Winter | | 2-row | 1995 | United Kingdom | | | Marinka x Torrent | | Nickerson | |  |
| EXB_0039 | Fanfare | SCRI |  | Winter | | 2-row | 1995 | United Kingdom | | | Torrent x Finesse | | ICI seeds | |  |
| EXB_0041 | Fighter | SCRI |  | Winter | | 2-row | 1991 | United Kingdom | | | RPB 77-5155 x Marinka | | Nickerson | |  |
| EXB_0042 | Finesse | IPK | HOR 18725 | Winter | | 2-row | 1988 | United Kingdom | | | Igri x Maris Otter | | ICI seeds | |  |
| EXB_0047 | Gleam | JIC |  | Winter | | 2-row | 1996 | United Kingdom | | | Torrent x Puffin | | New Farm Crops (NFC) | |  |
| EXB_0050 | Halcyon | SCRI |  | Winter | | 2-row | 1968 | United Kingdom | | | Warboys x Maris Otter | | PBI | |  |
| EXB_0053 | Heligan | JIC | CPBT B16 | Winter | | 2-row | 1998 | United Kingdom | | | Tosca x Intro | | CPB Twyford | |  |
| EXB_0058 | Jewel | JIC | NSL 92-6915 | Winter | | 2-row | 1997 | United Kingdom | | | Clarine x Firefly | | Nickerson | |  |
| EXB_0059 | Kaskade | JIC |  | Winter | | 2-row | 1981 | Germany | | | Carsten x Union x Emir x Malta | | BPZ | |  |
| EXB_0060 | Kelibia |  |  | Winter | | 2-row | 1990 | France | | | Nika x Igri | | Verneuil | |  |
| EXB_0061 | Kestrel |  |  | Winter | | 2-row | 2003 | United Kingdom | | | Intro x Sunrise | | Advanta | |  |
| EXB_0063 | Labea | SCRI |  | Winter | | 2-row | 1992 | Germany | | | Br.269 c x LBP 5906 | | Breun | |  |
| EXB_0065 | Leonie |  |  | Winter | | 2-row | 2000 | Germany | | | Labea x 87585/6 | | Nordsaat | |  |
| EXB_0070 | Magie | JIC |  | Winter | | 2-row | 1983 | France | | | LBP 1911 x Alpha | | Serasem | |  |
| EXB_0071 | Malta | JIC |  | Winter | | 2-row | 1968 | Germany | | | ((Carstens 2-Row x Aurea) x Dea) x Herfordia | | Ackermann | |  |
| EXB_0072 | Malwinta |  |  | Winter | | 2-row | 2004 | Denmark | | | Opal x Labea | | Pajbjergfonden | |  |
| EXB_0074 | Manolia |  |  | Winter | | 2-row | 2001 | France | | | Labea x Astrid | | Secobra | |  |
| EXB_0076 | Marinka | SCRI |  | Winter | | 2-row | 1985 | Netherlands | | | (Alpha x SvP 674) x Malta | | Cebeco | |  |
| EXB_0077 | Maris Otter | SCRI |  | Winter | | 2-row | 1965 | United Kingdom | | | Proctor x Pioneer | | PBI | |  |
| EXB_0078 | Maris Trojan | IPK | HOR 8250 | Winter | | 2-row | 1975 | United Kingdom | | | (Emir x Maris Concord) x [(C205 x Maris Otter) x (Carstens Wintergerste x (France 7 x (Maris Concord x Maris Puma)))] | | PBI | |  |
| EXB_0080 | Melanie | JIC | BR 789A | Winter | | 2-row | 1993 | Germany | | | BR301a x LBP5907 | | Breun | |  |
| EXB_0084 | Murcie |  |  | Winter | | 2-row | 2002 | France | | | Sunrise x Labea | | Desprez | |  |
| EXB_0088 | Nure | SCRI |  | Winter | | 2-row | 1998 | Italy | | | (Fior 40 x Alpha) x Baraka | | CRA-Fiorenzuola | |  |
| EXB_0090 | Opal |  |  | Winter | | 2-row | 1998 | United Kingdom | | | Puffin x Angora | | Nickerson | |  |
| EXB_0092 | Panda | SCRI |  | Winter | | 2-row | 1983 | France | | | Katy x Gerbel | | Desprez | |  |
| EXB_0094 | Pastoral | SCRI |  | Winter | | 2-row | 1987 | France | | | Igri x Mogador | | Secobra | |  |
| EXB_0096 | Pearl | SCRI |  | Winter | | 2-row | 1997 | United Kingdom | | | Puffin x Angora | | Nickerson | |  |
| EXB_0101 | Platine |  |  | Winter | | 2-row | 1996 | France | | | Intro x Mariane | | Serasem | |  |
| EXB_0104 | Puffin | SCRI |  | Winter | | 2-row | 1989 | United Kingdom | | | (Athos x Maris Otter) x Igri | | ICI seeds | |  |
| EXB_0106 | Saigon |  |  | Winter | | 2-row | 2002 | United Kingdom | | | Opal x 93-5758 | | Nickerson | |  |
| EXB_0109 | Sonja | JIC |  | Winter | | 2-row | 1974 | Germany | | | Tria x Malta | | Engelen | |  |
| EXB_0111 | Sprite | JIC | CWB 699-47 | Winter | | 2-row | 1992 | United Kingdom | | | CWB 11/8 x Halcyon | | PBI | |  |
| EXB_0113 | Target | IPK | HOR 18270 | Winter | | 2-row | 1992 | United Kingdom | | | Igri x (Ramage 2-row x Gerbel) | | New Farm Crops (NFC) | |  |
| EXB_0423 | Tidone |  |  | Winter | | 2-row | 1994 | Italy | | | (Okos x 273 cat.) x Igri | | CRA-Fiorenzuola | |  |
| EXB_0117 | Trasimeno |  |  | Winter | | 2-row | 1992 | Italy? | | | Osk4.41/2 x Igri | | POLJOPRIVREDNI INSTITUT | |  |
| EXB_0119 | Tria | SCRI |  | Winter | | 2-row | 1963 | Germany | | | (LBP 348/342 x Firlbecks 4zlg.) x Carsten 1565 | | Ackermann | |  |
| EXB_0120 | Vanessa | SCRI |  | Winter | | 2-row | 2000 | Germany | | | (Br.652h x Br.1201a) x Astrid | | Breun | |  |
| EXB_0121 | Verticale |  |  | Winter | | 2-row | 2000 | France | | | Target x Intro | | Serasem | |  |
| EXB_0124 | Zacinto |  |  | Winter | | 2-row | 2000 | Italy | | | (IABO329 x Arda) x Arda | | CRA-Fiorenzuola | |  |
| EXB_0116 | Torrent | SCRI |  | Winter | | 2-rpw | 1987 | United Kingdom | | | Fenella x TRI 685 | | Nickerson | |  |
| EXB_0005 | Aldebaran |  |  | Winter | | 6-row | 2003 | Italy | | | Rebelle x Jaidor | | CRA-Fiorenzuola | |  |
| EXB_0013 | Arma | IPK | HOR 8903 | Winter | | 6-row | 1976 | France | | | Manon x Ager x Hauter x Ares | | Desprez | |  |
| EXB_0016 | Athene | IPK | HOR 10542 | Winter | | 6-row | 1977 | Germany | | | (Herfordia x Hord.sp.nigrum H204) x (Mädru x Weissenhaus-Stamm) | | Eckendorf | |  |
| EXB_0018 | Balaki |  |  | Winter | | 6-row | 1996 | France | | | Express x 3169 LH2 | | Secobra | |  |
| EXB_0019 | Balda |  |  | Winter | | 6-row | 1998 | Italy | | | (Borwina x Express) x 75 | | Apsov | |  |
| EXB_0022 | Canoro |  |  | Winter | | 6-row | 1992 | Italy | | | Katja x Barberousse | | Secobra | |  |
| EXB_0024 | Carola |  |  | Winter | | 6-row | 1998 | Germany | | | (SG 402085 x Franka) x GW 1307 | | Nordsaat | |  |
| EXB_0030 | Dea | IPK |  | Winter | | 6-row | 1953 | Germany | | | ((Ragusa x Peragis12) x (Heils Franken x Frw.Berg)) x ((Mahnd.Viktoria) x (Ragusa x Bolivia)) | | Engelen | |  |
| EXB_0035 | Dura | JIC |  | Winter | | 6-row | 1961 | Germany | | | (Friedrichswerther Berg x Ragusa) x Doria | | Streng | |  |
| EXB_0036 | Esterel |  |  | Winter | | 6-row | 1996 | France | | | 7761 x Plaisant | | Secobra | |  |
| EXB_0038 | Express | IPK | HOR 18405 | Winter | | 6-row | 1990 | France | | | Robur x Athene | | Serasem | |  |
| EXB_0040 | Federal |  |  | Winter | | 6-row | 1994 | France | | | (Capri x Bison) x Melusine | | Secobra | |  |
| EXB_0043 | Franka | IPK | HOR 20536 | Winter | | 6-row | 1980 | Germany | | | ((Vogels.Gold x Senta) x (Dura x Dea)) x Vogels.Gold | | Streng | |  |
| EXB_0044 | Fridericus |  |  | Winter | | 6-row | 2006 | Germany | | | Carola x LP 6-564 | | KWS | |  |
| EXB_0045 | Frost | IPK |  | Winter | | 6-row | 1988 | Sweden | | | Pella x Astrix | | Weibull | |  |
| EXB_0046 | Gerbel |  |  | Winter | | 6-row | 1977 | France | | | (Ager x Jumbo) x FDE 244/95(Ager x Asterix x Mana) | | Desprez | |  |
| EXB_0048 | Glenan |  |  | Winter | | 6-row | 1988 | France | | | Thibaut x Robur | | Verneuil | |  |
| EXB_0049 | Grete | IPK | HOR 10862 | Winter | | 6-row | 1989 | Germany | | | (641003 x Tocka) x Vogels.Gold x (Pella x Dura) | | Eckendorf | |  |
| EXB_0052 | Hasso | IPK |  | Winter | | 6-row | 1980 | Germany | | | Dura x 12563 (Weissenhaus 6448 x Hauter x D5) | | Lochow-Petkus | |  |
| EXB_0264 | Hatif de Grignon | SCRI |  | Winter | | 6-row | 1937 | France | | | Selection from France landraces | | Institut National Agronomique Paris-Grignon (FR) | |  |
| EXB_0054 | Herfordia | SCRI |  | Winter | | 6-row | 1950 | Germany | | | Peragis-Stamm x Schladener I | | Dippe | |  |
| EXB_0055 | Hoppel | JIC | JIC 8152 | Winter | | 6-row | 1970 | France | | | (Hybrid 456 x Feebar) x Hatif de Grignon | | Blondeau | |  |
| EXB_0056 | Isa |  |  | Winter | | 6-row | 1985 | Belgium | | | Bosquet x Hoppel | | na | |  |
| EXB_0057 | Jaidor | IPK | HOR 10188 | Winter | | 6-row | 1981 | France | | | ((Rika x Balaldi 16) x (33 x Emir) x (Ema 1038 x Robur) | | INRA | |  |
| EXB_0062 | Ketos |  |  | Winter | | 6-row | 2002 | France | | | (Gotic x Orblonde) x (12813 x 91H595) | | Limagrain Verneuil | |  |
| EXB_0064 | Laverda |  |  | Winter | | 6-row | 2006 | Germany | | | (Ludmilla x Nord 1836) x Merlot | | Saaten Union | |  |
| EXB_0066 | Lonni |  |  | Winter | | 6-row | 2005 | Denmark | | | Aviron x Carola | | Pajbjergfonden | |  |
| EXB_0067 | Lorena |  |  | Winter | | 6-row | 1993 | Germany | | | Mr Kanada x Eng.50-8 | | Saaten Union | |  |
| EXB_0068 | Ludmilla |  |  | Winter | | 6-row | 1999 | Germany | | | Hasso x (Banteng x Venus) | | Firlbeck | |  |
| EXB_0069 | Lutece |  |  | Winter | | 6-row | 2003 | France | | | Nikel x Rebelle | | Serasem | |  |
| EXB_0073 | Manitou | IPK | HOR 10920 | Winter | | 6-row | 1988 | France | | | 1055 x Gerbel | | Secobra | |  |
| EXB_0075 | Marado |  |  | Winter | | 6-row | 2003 | France | | | Nikel x 1523 | | Serasem | |  |
| EXB_0079 | Mattina |  |  | Winter | | 6-row | 1998 | France | | | (Friberga x Express) x (L40km x 43) | | INRA | |  |
| EXB_0081 | Mirco |  |  | Winter | | 6-row | 1981 | Italy | | | Selection from local population not identified | | na | |  |
| EXB_0082 | Mirra | IPK |  | Winter | | 6-row | 1972 | Germany | | | 6109 x Herfordia | | Eckendorf | |  |
| EXB_0085 | Muscat | JIC | CPB B8 | Winter | | 6-row | 1996 | United Kingdom | | | Plaisant x Gaulois | | CPB Twyford | |  |
| EXB_0086 | Naomie |  |  | Winter | | 6-row | 2003 | Germany | | | (Julia x NS.90517/16) x Carola | | Ackermann | |  |
| EXB_0089 | Onice |  |  | Winter | | 6-row | 1980 | Italy | | | Perga x Sam-Chio 36 | | SIS Bologna | |  |
| EXB_0091 | Osiris | IPK |  | Winter | | 6-row | 1921 | Algeria | | | Selection from PI39590 | | na | |  |
| EXB_0093 | Passport |  |  | Winter | | 6-row | 1997 | France | | | (Barberousse x 5.4) x Barberousse | | Lemaire | |  |
| EXB_0095 | Patricia |  |  | Winter | | 6-row | 1995 | Austria | | | Romy x (Rachel x MR 13) | | Probstdorf | |  |
| EXB_0097 | Pilastro |  |  | Winter | | 6-row | 1989 | Italy | | | Arma x Sisfor L 38 | | SIS | |  |
| EXB_0098 | Pirate | IPK | HOR 10196 | Winter | | 6-row | 1978 | France | | | (LBP 259/711 x Ares 176) x Pella | | Ringot | |  |
| EXB_0100 | Plaisant | SCRI |  | Winter | | 6-row | 1979 | France | | | Ager x Nymphe | | GAE | |  |
| EXB_0102 | Ponente |  |  | Winter | | 6-row | 2001 | Italy | | | (Vetulkio x Arma) x Express | | CRA | |  |
| EXB_0103 | Princess | IPK |  | Winter | | 6-row | 1989 | France | | | (Gerbel x Barteng) x Barberousse | | Lemaire | |  |
| EXB_0105 | Robur | IPK | HOR 10200 | Winter | | 6-row | 1973 | France | | | Ager x (Hatif de Grignon x Ares) | | INRA | |  |
| EXB_0108 | Senta | IPK |  | Winter | | 6-row | 1963 | Germany | | | (Firlbecks 4zlg. x Ungarische) x Dea | | Engelen | |  |
| EXB_0110 | Sonora |  |  | Winter | | 6-row | 1992 | France | | | (Plaisant x Gerbel) x Jaidor | | Verneuil | |  |
| EXB_0112 | Tapir | IPK |  | Winter | | 6-row | 1980 | Netherlands | | | DSGW 167 x Pella | | Mansholt | |  |
| EXB_0118 | Trebbia |  |  | Winter | | 6-row | 1990 | Italy | | | Selection from Fior Synt 3 | | CRA | |  |
| EXB_0123 | Vogelsanger Gold | JIC |  | Winter | | 6-row | 1965 | Germany | | | (Isaria x H.spontaneum nigrum) x Wintergerste-5 | | Hauptsaaten | |  |
| EXB_0125 | Zoe |  |  | Winter | | 6-row | 1998 | Germany | | | 0 | | Nickerson | |  |
