# Supplementary material for: Genome‐Wide Association Analysis of Grain Yield‐Associated Traits in a Pan‐European Barley Cultivar Collection
Source: Plant Genome. 2018 Mar 1;11(1):170073. doi: 10.3835/plantgenome2017.08.0073 (PMC12962562; doi:10.3835/plantgenome2017.08.0073)
Supplement: Supplementary file 2 [file TPG2-11-170073-s002.docx]

**Supplemental Table S2: Genetic linkage map for SNP markers used in this study**

```
CHR	MARKER	POSITION
1H	12_30968	0
1H	12_30969	0
1H	11_20373	0
1H	11_20479	0
1H	12_10420	0
1H	SCRI_RS_180989	0
1H	SCRI_RS_35790	0
1H	SCRI_RS_142714	0
1H	11_10895	0.65
1H	SCRI_RS_138128	1.36
1H	SCRI_RS_138156	1.36
1H	11_21354	1.37
1H	11_21067	2.13
1H	SCRI_RS_113745	2.13
1H	SCRI_RS_120053	2.13
1H	SCRI_RS_120059	2.13
1H	SCRI_RS_124234	2.51
1H	SCRI_RS_194326	2.51
1H	12_30715	2.97
1H	11_20502	2.99
1H	12_11311	3.25
1H	SCRI_RS_107687	3.29
1H	12_30944	3.44
1H	12_30945	3.44
1H	SCRI_RS_165910	3.47
1H	11_10654	3.9
1H	12_31303	3.9
1H	SCRI_RS_161137	3.9
1H	SCRI_RS_193700	3.9
1H	SCRI_RS_194514	3.9
1H	SCRI_RS_206684	3.9
1H	SCRI_RS_207257	3.9
1H	12_10410	4.47
1H	12_10636	4.75
1H	12_31144	4.75
1H	SCRI_RS_60145	4.75
1H	SCRI_RS_66630	4.75
1H	SCRI_RS_82277	4.75
1H	11_10419	4.77
1H	SCRI_RS_149683	4.77
1H	SCRI_RS_232577	4.77
1H	SCRI_RS_60293	4.77
1H	SCRI_RS_204348	4.79
1H	SCRI_RS_139538	5.22
1H	SCRI_RS_132175	5.99
1H	SCRI_RS_234759	5.99
1H	SCRI_RS_200110	6.27
1H	12_11011	6.28
1H	12_30933	6.28
1H	SCRI_RS_198544	6.28
1H	11_21174	8.69
1H	11_21226	8.78
1H	SCRI_RS_162524	8.78
1H	SCRI_RS_184274	10.08
1H	SCRI_RS_1929	10.08
1H	SCRI_RS_224681	10.18
1H	SCRI_RS_224686	10.18
1H	12_31149	11.17
1H	12_30951	11.17
1H	12_30952	11.17
1H	12_30919	11.97
1H	12_30950	11.97
1H	SCRI_RS_129789	11.97
1H	SCRI_RS_134663	11.97
1H	SCRI_RS_165792	11.97
1H	SCRI_RS_169369	11.97
1H	SCRI_RS_222250	11.97
1H	SCRI_RS_130600	12.07
1H	SCRI_RS_148733	12.07
1H	SCRI_RS_169942	12.07
1H	SCRI_RS_3336	12.07
1H	12_30918	12.07
1H	SCRI_RS_119312	12.72
1H	SCRI_RS_169756	13.05
1H	SCRI_RS_148560	13.97
1H	12_30955	14.41
1H	SCRI_RS_137023	15.67
1H	SCRI_RS_164150	15.67
1H	12_30588	15.67
1H	11_10775	15.73
1H	11_20749	15.73
1H	12_30118	17.87
1H	SCRI_RS_130592	17.87
1H	SCRI_RS_182656	17.87
1H	12_30876	18.04
1H	SCRI_RS_157757	18.77
1H	12_30948	19.56
1H	SCRI_RS_222236	19.56
1H	SCRI_RS_155295	19.63
1H	SCRI_RS_13972	20.05
1H	SCRI_RS_169668	20.05
1H	11_10873	20.15
1H	SCRI_RS_141877	20.26
1H	SCRI_RS_189465	20.6
1H	11_10186	21.86
1H	11_20371	22.96
1H	11_10030	23.06
1H	11_10744	23.44
1H	11_21048	23.85
1H	12_30877	24.1
1H	SCRI_RS_128285	25.26
1H	SCRI_RS_139220	25.26
1H	SCRI_RS_152464	25.26
1H	SCRI_RS_171186	25.26
1H	SCRI_RS_189506	25.26
1H	SCRI_RS_205669	25.26
1H	SCRI_RS_232660	25.26
1H	11_20617	26.94
1H	SCRI_RS_235243	27.69
1H	SCRI_RS_176084	27.69
1H	SCRI_RS_10778	29.05
1H	SCRI_RS_122490	29.05
1H	SCRI_RS_151047	29.05
1H	SCRI_RS_179683	29.05
1H	SCRI_RS_217160	29.05
1H	SCRI_RS_4891	29.26
1H	SCRI_RS_14227	29.26
1H	11_20440	29.26
1H	SCRI_RS_169091	29.26
1H	SCRI_RS_153360	29.33
1H	SCRI_RS_184530	29.33
1H	12_30241	29.56
1H	SCRI_RS_154811	29.7
1H	11_10238	29.97
1H	12_11357	29.97
1H	11_20712	30.55
1H	SCRI_RS_124926	30.55
1H	SCRI_RS_138043	30.55
1H	SCRI_RS_150226	30.55
1H	SCRI_RS_171221	30.55
1H	SCRI_RS_182603	30.55
1H	SCRI_RS_88375	30.55
1H	SCRI_RS_117492	31.99
1H	11_10757	32.45
1H	SCRI_RS_155382	32.45
1H	SCRI_RS_239784	32.66
1H	12_11498	34.27
1H	11_21095	35.59
1H	SCRI_RS_123187	36.44
1H	12_11266	36.46
1H	12_30268	36.46
1H	12_31276	36.69
1H	12_10314	38.18
1H	SCRI_RS_165811	39.16
1H	11_10760	40
1H	12_31177	40.16
1H	11_10294	40.22
1H	12_30817	40.24
1H	11_21072	40.66
1H	11_10438	42.24
1H	11_11336	42.24
1H	11_20047	42.24
1H	12_11036	42.24
1H	12_21408	42.24
1H	12_30043	42.24
1H	12_30498	42.24
1H	12_30336	42.29
1H	11_11484	43.99
1H	SCRI_RS_116548	44.31
1H	SCRI_RS_126734	44.31
1H	SCRI_RS_161702	44.31
1H	12_10938	44.41
1H	12_11209	44.41
1H	12_31381	44.41
1H	12_31124	44.49
1H	11_10764	44.56
1H	11_11162	45.02
1H	12_30059	45.02
1H	12_30462	45.02
1H	12_30592	45.02
1H	12_31272	45.02
1H	SCRI_RS_189197	45.39
1H	SCRI_RS_1948	45.39
1H	11_21000	45.67
1H	SCRI_RS_206772	46.1
1H	11_21134	46.26
1H	11_20912	46.32
1H	SCRI_RS_189637	46.51
1H	SCRI_RS_114047	46.67
1H	SCRI_RS_11615	46.67
1H	SCRI_RS_160545	46.67
1H	SCRI_RS_193392	46.67
1H	SCRI_RS_205816	46.67
1H	12_11107	47.03
1H	12_30522	47.03
1H	SCRI_RS_140837	47.6
1H	SCRI_RS_153785	47.6
1H	SCRI_RS_170869	47.6
1H	SCRI_RS_170878	47.6
1H	SCRI_RS_184784	47.6
1H	SCRI_RS_189483	47.6
1H	SCRI_RS_230987	47.6
1H	11_20514	47.82
1H	SCRI_RS_141140	47.82
1H	SCRI_RS_183171	47.82
1H	SCRI_RS_187264	47.82
1H	11_11359	48.11
1H	11_10275	48.13
1H	11_10597	48.13
1H	SCRI_RS_147042	48.13
1H	12_30243	48.81
1H	SCRI_RS_132461	48.92
1H	SCRI_RS_199178	48.92
1H	SCRI_RS_199179	48.92
1H	SCRI_RS_9480	48.92
1H	11_10259	49.36
1H	SCRI_RS_124727	49.36
1H	SCRI_RS_221759	49.36
1H	12_20641	49.42
1H	11_11256	49.47
1H	11_10526	49.56
1H	12_31467	49.56
1H	SCRI_RS_152144	49.56
1H	12_30683	49.66
1H	12_10491	49.68
1H	SCRI_RS_198854	49.68
1H	SCRI_RS_222748	49.68
1H	SCRI_RS_188937	49.86
1H	SCRI_RS_141598	50.28
1H	11_10324	50.36
1H	SCRI_RS_214559	50.56
1H	12_10159	50.56
1H	12_30436	50.56
1H	12_10235	50.56
1H	12_30762	50.56
1H	SCRI_RS_116685	50.56
1H	12_30306	50.56
1H	SCRI_RS_1914	50.56
1H	SCRI_RS_200294	50.56
1H	SCRI_RS_230133	50.56
1H	11_11478	50.56
1H	12_30438	50.56
1H	11_20698	50.56
1H	11_20757	50.56
1H	12_11169	50.56
1H	12_11301	50.56
1H	12_30562	50.56
1H	12_31208	50.56
1H	SCRI_RS_123138	50.56
1H	11_10075	50.56
1H	11_10470	50.56
1H	11_10957	50.56
1H	11_11064	50.56
1H	11_11287	50.56
1H	11_20260	50.56
1H	11_20660	50.56
1H	11_20798	50.56
1H	11_20810	50.56
1H	11_20855	50.56
1H	11_21193	50.56
1H	11_21312	50.56
1H	11_21357	50.56
1H	12_10300	50.56
1H	12_10489	50.56
1H	12_30350	50.56
1H	12_30404	50.56
1H	12_30406	50.56
1H	12_30694	50.56
1H	12_30796	50.56
1H	12_31134	50.56
1H	SCRI_RS_118168	50.56
1H	SCRI_RS_129941	50.56
1H	SCRI_RS_142702	50.56
1H	SCRI_RS_149726	50.56
1H	SCRI_RS_149971	50.56
1H	SCRI_RS_158003	50.56
1H	SCRI_RS_163127	50.56
1H	SCRI_RS_170542	50.56
1H	SCRI_RS_171543	50.56
1H	SCRI_RS_160916	50.56
1H	12_30110	50.56
1H	11_20427	50.63
1H	12_10506	50.63
1H	SCRI_RS_216058	50.63
1H	11_10985	50.66
1H	SCRI_RS_138691	50.66
1H	SCRI_RS_229698	50.7
1H	SCRI_RS_151874	50.79
1H	11_10520	50.86
1H	12_30348	51.02
1H	SCRI_RS_236235	51.07
1H	11_21217	51.92
1H	11_11326	52.07
1H	12_30750	52.87
1H	11_21361	52.87
1H	12_30478	52.91
1H	12_30499	52.91
1H	11_10933	53.11
1H	12_30786	53.11
1H	11_20997	53.22
1H	SCRI_RS_151764	53.81
1H	SCRI_RS_182631	53.81
1H	12_30710	53.82
1H	11_21053	54.11
1H	12_10198	54.15
1H	SCRI_RS_85917	54.22
1H	SCRI_RS_85918	54.22
1H	11_10552	54.36
1H	12_11444	54.36
1H	12_30343	54.72
1H	SCRI_RS_141511	54.91
1H	SCRI_RS_143927	54.91
1H	SCRI_RS_155439	54.91
1H	SCRI_RS_172170	54.91
1H	SCRI_RS_186830	54.91
1H	SCRI_RS_189248	54.91
1H	SCRI_RS_189586	54.91
1H	SCRI_RS_213103	54.91
1H	SCRI_RS_7197	54.91
1H	SCRI_RS_124377	55.01
1H	SCRI_RS_193640	55.01
1H	SCRI_RS_17924	55.11
1H	SCRI_RS_82327	55.11
1H	SCRI_RS_132599	55.45
1H	SCRI_RS_132604	55.45
1H	SCRI_RS_139785	55.45
1H	12_10105	55.78
1H	SCRI_RS_217272	55.78
1H	SCRI_RS_188820	55.81
1H	11_10768	55.85
1H	11_21333	55.97
1H	SCRI_RS_122889	56.25
1H	SCRI_RS_125407	56.25
1H	SCRI_RS_160466	56.25
1H	SCRI_RS_189898	56.25
1H	SCRI_RS_189920	56.25
1H	SCRI_RS_219043	56.25
1H	SCRI_RS_1445	56.25
1H	SCRI_RS_207335	56.25
1H	SCRI_RS_122216	56.56
1H	SCRI_RS_148600	56.89
1H	SCRI_RS_225107	56.89
1H	SCRI_RS_145305	57.05
1H	SCRI_RS_213455	57.05
1H	SCRI_RS_229636	57.05
1H	SCRI_RS_907	57.05
1H	SCRI_RS_100503	57.05
1H	SCRI_RS_109060	57.05
1H	SCRI_RS_14834	57.05
1H	SCRI_RS_153733	57.05
1H	SCRI_RS_161643	57.05
1H	SCRI_RS_165072	57.05
1H	SCRI_RS_167877	57.05
1H	SCRI_RS_17256	57.05
1H	SCRI_RS_193401	57.05
1H	SCRI_RS_198546	57.05
1H	SCRI_RS_152642	57.05
1H	SCRI_RS_153434	57.05
1H	SCRI_RS_204810	57.05
1H	SCRI_RS_221609	57.05
1H	SCRI_RS_130590	57.05
1H	SCRI_RS_229932	57.05
1H	SCRI_RS_7813	57.05
1H	12_30672	57.05
1H	SCRI_RS_119743	57.05
1H	12_10201	57.05
1H	12_30753	57.05
1H	SCRI_RS_152795	57.05
1H	SCRI_RS_138112	57.05
1H	SCRI_RS_138118	57.05
1H	SCRI_RS_201072	57.05
1H	SCRI_RS_207081	57.05
1H	12_30821	57.05
1H	SCRI_RS_132246	57.41
1H	SCRI_RS_121978	57.94
1H	SCRI_RS_211274	57.94
1H	SCRI_RS_156506	57.94
1H	SCRI_RS_56976	57.94
1H	SCRI_RS_175480	58.19
1H	SCRI_RS_179793	58.41
1H	11_20095	58.41
1H	11_10176	58.41
1H	12_30744	58.65
1H	SCRI_RS_130666	58.85
1H	SCRI_RS_182431	58.85
1H	SCRI_RS_192779	58.85
1H	SCRI_RS_21483	58.85
1H	SCRI_RS_236104	58.85
1H	SCRI_RS_237999	58.85
1H	12_21131	59.35
1H	11_10798	59.65
1H	11_10617	59.81
1H	11_11049	59.81
1H	SCRI_RS_118785	59.81
1H	SCRI_RS_12873	59.81
1H	SCRI_RS_2945	59.81
1H	12_30304	59.81
1H	SCRI_RS_231735	59.85
1H	11_10302	60.01
1H	12_31179	60.75
1H	SCRI_RS_132028	61.35
1H	SCRI_RS_236551	61.35
1H	11_20432	61.41
1H	12_30477	61.41
1H	SCRI_RS_239686	61.41
1H	11_21431	61.96
1H	12_10313	62.1
1H	SCRI_RS_147134	62.1
1H	SCRI_RS_193657	62.1
1H	11_10516	62.5
1H	SCRI_RS_194015	62.86
1H	11_10043	63.1
1H	SCRI_RS_151894	63.1
1H	SCRI_RS_219685	63.77
1H	SCRI_RS_160500	63.77
1H	SCRI_RS_170810	63.77
1H	SCRI_RS_204611	63.77
1H	12_31464	63.8
1H	11_21219	63.8
1H	11_10002	63.8
1H	12_11062	63.8
1H	SCRI_RS_206795	63.9
1H	11_11367	63.9
1H	SCRI_RS_116661	64.56
1H	SCRI_RS_151228	64.56
1H	SCRI_RS_180349	64.56
1H	SCRI_RS_85391	64.56
1H	SCRI_RS_120605	64.75
1H	SCRI_RS_133886	65.62
1H	SCRI_RS_175487	65.62
1H	SCRI_RS_120518	65.62
1H	SCRI_RS_1473	65.62
1H	SCRI_RS_169123	65.62
1H	SCRI_RS_182406	65.62
1H	SCRI_RS_183588	65.62
1H	12_31099	65.75
1H	12_30295	66.85
1H	SCRI_RS_10836	66.99
1H	SCRI_RS_196439	66.99
1H	12_10166	68.67
1H	SCRI_RS_153727	68.74
1H	SCRI_RS_181135	68.74
1H	12_20696	69.57
1H	11_20157	69.57
1H	12_30298	69.58
1H	11_20290	69.58
1H	SCRI_RS_155723	69.58
1H	12_30505	69.62
1H	SCRI_RS_157039	69.62
1H	SCRI_RS_195238	69.62
1H	12_10206	70.15
1H	12_30147	70.15
1H	12_30742	71.82
1H	11_20229	71.82
1H	SCRI_RS_145336	71.82
1H	SCRI_RS_159201	71.82
1H	11_21057	71.82
1H	12_21176	72.49
1H	SCRI_RS_181239	73.23
1H	12_11267	73.23
1H	11_10686	73.42
1H	SCRI_RS_151244	73.58
1H	SCRI_RS_823	73.58
1H	SCRI_RS_138527	73.62
1H	SCRI_RS_188360	73.62
1H	SCRI_RS_160234	73.72
1H	11_21126	73.93
1H	11_10006	73.93
1H	11_10890	73.93
1H	SCRI_RS_21080	74.33
1H	SCRI_RS_156208	75.46
1H	SCRI_RS_219726	75.46
1H	SCRI_RS_4893	75.46
1H	11_10279	75.46
1H	11_20121	75.46
1H	11_20990	75.46
1H	12_11078	76.6
1H	11_20657	77.16
1H	SCRI_RS_181353	79.76
1H	SCRI_RS_125339	83.16
1H	12_11463	84.38
1H	SCRI_RS_9158	84.57
1H	SCRI_RS_225431	85.27
1H	11_20434	85.27
1H	12_11144	85.27
1H	11_20550	85.46
1H	12_31160	85.46
1H	11_10830	85.46
1H	SCRI_RS_135092	85.52
1H	SCRI_RS_154140	85.52
1H	SCRI_RS_157246	85.52
1H	SCRI_RS_236576	85.52
1H	11_10471	85.52
1H	SCRI_RS_114255	85.59
1H	SCRI_RS_209524	87.03
1H	11_20792	87.39
1H	SCRI_RS_121048	87.39
1H	SCRI_RS_144315	87.39
1H	SCRI_RS_170389	87.39
1H	SCRI_RS_17486	87.39
1H	SCRI_RS_139690	87.39
1H	12_10535	87.39
1H	SCRI_RS_21187	87.59
1H	SCRI_RS_213675	88.25
1H	SCRI_RS_188909	88.29
1H	SCRI_RS_197263	88.29
1H	SCRI_RS_189168	88.42
1H	11_20769	88.42
1H	SCRI_RS_179987	88.42
1H	SCRI_RS_195200	88.42
1H	SCRI_RS_201866	88.42
1H	SCRI_RS_236623	88.42
1H	SCRI_RS_103372	88.42
1H	SCRI_RS_133132	88.42
1H	SCRI_RS_166168	88.42
1H	SCRI_RS_181300	88.42
1H	11_20475	88.49
1H	11_21446	88.49
1H	12_30546	89.11
1H	11_20971	89.75
1H	12_20323	89.75
1H	12_20632	89.75
1H	12_31229	89.75
1H	SCRI_RS_114308	89.75
1H	SCRI_RS_168562	89.75
1H	SCRI_RS_177788	89.75
1H	SCRI_RS_182326	89.75
1H	SCRI_RS_197910	89.75
1H	SCRI_RS_231869	89.75
1H	SCRI_RS_7407	89.75
1H	11_10433	90.1
1H	SCRI_RS_130139	90.15
1H	SCRI_RS_142282	90.15
1H	SCRI_RS_194371	90.15
1H	SCRI_RS_199689	90.15
1H	11_21213	91.91
1H	SCRI_RS_172734	91.91
1H	SCRI_RS_149772	91.91
1H	12_31163	91.91
1H	11_21373	91.91
1H	12_11008	91.91
1H	SCRI_RS_188218	92.32
1H	11_20149	93.64
1H	11_10396	93.64
1H	SCRI_RS_176664	93.64
1H	SCRI_RS_231267	93.64
1H	SCRI_RS_138010	95.22
1H	12_31319	95.36
1H	SCRI_RS_139242	95.36
1H	12_31192	95.36
1H	11_11277	95.36
1H	SCRI_RS_145026	96.21
1H	SCRI_RS_136856	96.26
1H	SCRI_RS_235724	96.26
1H	SCRI_RS_235968	96.26
1H	SCRI_RS_78578	96.26
1H	11_10522	96.57
1H	11_20754	97.22
1H	11_20169	97.22
1H	SCRI_RS_139733	98.03
1H	SCRI_RS_154528	98.03
1H	SCRI_RS_11137	98.97
1H	12_11173	99.04
1H	11_10357	99.04
1H	SCRI_RS_143810	99.81
1H	SCRI_RS_6913	100.01
1H	SCRI_RS_199972	100.04
1H	SCRI_RS_213539	100.04
1H	SCRI_RS_787	100.18
1H	12_20187	100.82
1H	12_20613	100.82
1H	12_21020	100.82
1H	12_31152	100.82
1H	SCRI_RS_146959	100.82
1H	SCRI_RS_154646	100.82
1H	SCRI_RS_155112	100.82
1H	SCRI_RS_236160	100.82
1H	11_20125	100.82
1H	11_20153	100.82
1H	12_30532	100.82
1H	SCRI_RS_171501	100.97
1H	11_10111	101
1H	11_20267	101
1H	11_20909	101
1H	12_31181	101.25
1H	SCRI_RS_165086	101.25
1H	SCRI_RS_201086	101.25
1H	SCRI_RS_6450	102.31
1H	12_30191	102.82
1H	SCRI_RS_215740	102.82
1H	SCRI_RS_232650	102.82
1H	12_30509	102.82
1H	SCRI_RS_141421	102.95
1H	SCRI_RS_162	102.95
1H	11_20780	103.37
1H	SCRI_RS_106752	103.37
1H	SCRI_RS_106754	103.37
1H	SCRI_RS_206759	103.37
1H	SCRI_RS_175646	103.92
1H	11_20921	104.23
1H	SCRI_RS_197337	104.83
1H	SCRI_RS_156823	104.83
1H	SCRI_RS_207276	104.83
1H	11_20625	104.9
1H	SCRI_RS_176063	105.01
1H	11_20220	105.23
1H	SCRI_RS_161388	105.23
1H	11_20844	105.23
1H	12_10149	105.23
1H	SCRI_RS_120418	105.23
1H	SCRI_RS_173813	105.23
1H	SCRI_RS_192730	105.37
1H	SCRI_RS_151414	105.37
1H	SCRI_RS_206896	105.37
1H	11_20021	106.37
1H	SCRI_RS_147329	107.63
1H	SCRI_RS_155427	107.63
1H	SCRI_RS_174052	108.08
1H	SCRI_RS_238125	108.5
1H	SCRI_RS_127968	108.5
1H	11_10789	108.62
1H	SCRI_RS_218473	108.62
1H	SCRI_RS_120481	108.93
1H	SCRI_RS_219889	108.93
1H	12_31029	108.93
1H	SCRI_RS_155997	108.97
1H	BK_01	108.97
1H	12_30014	109.28
1H	11_21392	109.28
1H	SCRI_RS_91596	109.61
1H	12_31526	110.31
1H	SCRI_RS_157953	110.47
1H	12_10905	110.5
1H	SCRI_RS_168172	111.8
1H	12_11409	112.46
1H	11_10338	114.95
1H	11_10854	115.75
1H	SCRI_RS_127646	115.75
1H	11_20959	115.75
1H	12_30257	115.75
1H	SCRI_RS_224392	116.8
1H	12_21172	117.53
1H	11_10911	117.77
1H	11_11528	118.31
1H	11_20908	118.31
1H	12_31105	119.07
1H	12_11457	119.78
1H	SCRI_RS_48925	121.15
1H	11_21038	121.23
1H	SCRI_RS_151464	121.29
1H	SCRI_RS_201865	121.3
1H	12_21522	122
1H	11_10586	122.36
1H	SCRI_RS_4522	122.36
1H	11_20940	122.36
1H	SCRI_RS_150607	122.36
1H	SCRI_RS_196910	122.36
1H	SCRI_RS_137116	122.43
1H	SCRI_RS_147611	122.43
1H	SCRI_RS_155758	122.43
1H	SCRI_RS_170110	122.43
1H	12_11443	122.43
1H	SCRI_RS_159031	122.64
1H	11_10722	123.14
1H	SCRI_RS_162765	123.56
1H	SCRI_RS_165588	123.56
1H	12_30403	123.66
1H	12_31377	123.66
1H	SCRI_RS_189967	123.66
1H	11_21140	123.66
1H	11_21068	123.85
1H	11_11481	123.96
1H	12_10207	123.96
1H	SCRI_RS_207076	123.99
1H	11_10644	124.26
1H	SCRI_RS_160475	124.28
1H	12_10808	125.33
1H	12_31387	125.33
1H	12_10693	126.53
1H	SCRI_RS_219802	126.53
1H	11_11038	127.29
1H	11_20133	127.76
1H	12_30649	127.76
1H	11_10903	129.07
1H	SCRI_RS_217245	129.66
1H	SCRI_RS_138629	130.04
1H	SCRI_RS_196448	130.04
1H	12_30444	130.52
1H	11_20383	130.56
1H	SCRI_RS_165338	130.56
1H	SCRI_RS_165476	130.56
1H	SCRI_RS_169881	130.56
1H	SCRI_RS_206532	130.56
1H	SCRI_RS_207416	130.56
1H	SCRI_RS_216088	130.56
1H	SCRI_RS_161239	130.56
1H	SCRI_RS_196025	130.66
1H	SCRI_RS_213666	130.94
1H	11_10782	131.21
1H	SCRI_RS_150786	132.86
1H	SCRI_RS_155407	132.86
1H	SCRI_RS_130891	133
1H	SCRI_RS_149262	133.05
1H	SCRI_RS_10956	133.05
1H	SCRI_RS_196512	133.37
1H	12_21105	133.37
1H	SCRI_RS_165600	133.37
1H	11_11509	134.45
1H	SCRI_RS_106140	134.45
1H	SCRI_RS_106142	134.45
1H	SCRI_RS_163142	134.45
1H	SCRI_RS_187051	134.45
1H	SCRI_RS_206337	134.45
1H	SCRI_RS_236175	134.45
1H	12_11496	134.45
1H	12_20599	134.45
1H	12_30517	134.45
1H	11_10041	134.45
1H	11_20594	134.45
1H	SCRI_RS_13869	134.45
1H	SCRI_RS_149094	134.45
1H	SCRI_RS_162628	134.45
1H	SCRI_RS_165434	134.45
1H	SCRI_RS_170857	134.45
1H	SCRI_RS_182206	134.45
1H	SCRI_RS_184058	134.45
1H	SCRI_RS_214888	134.45
1H	11_11105	134.45
1H	SCRI_RS_130577	134.45
1H	SCRI_RS_141322	134.45
1H	SCRI_RS_150563	134.45
1H	SCRI_RS_166469	134.45
1H	SCRI_RS_180081	134.45
1H	11_21384	134.45
1H	11_20603	134.45
1H	12_30277	134.45
1H	SCRI_RS_147318	134.97
1H	SCRI_RS_156009	134.97
1H	SCRI_RS_175774	134.97
1H	11_20138	135.65
1H	SCRI_RS_143790	135.65
1H	SCRI_RS_4928	135.65
1H	SCRI_RS_176006	135.85
1H	SCRI_RS_167999	135.99
1H	11_20840	136.1
1H	SCRI_RS_147896	136.1
1H	SCRI_RS_152330	136.1
1H	12_10746	136.15
1H	12_30231	136.15
1H	SCRI_RS_153896	136.15
1H	11_20915	136.15
1H	SCRI_RS_158298	136.15
1H	11_10590	136.24
1H	SCRI_RS_235776	136.24
1H	11_20772	136.25
1H	SCRI_RS_175218	136.25
1H	SCRI_RS_195067	136.25
1H	SCRI_RS_132472	136.25
1H	SCRI_RS_150935	136.25
1H	SCRI_RS_117283	136.36
1H	11_20509	136.36
1H	12_30935	136.36
1H	12_31081	136.36
1H	SCRI_RS_154621	136.36
1H	SCRI_RS_52789	136.36
1H	12_20429	136.36
1H	11_10443	136.36
1H	SCRI_RS_150984	136.36
1H	SCRI_RS_199705	136.36
1H	12_30934	136.43
1H	SCRI_RS_199945	136.56
2H	11_20609	0
2H	SCRI_RS_11169	0
2H	SCRI_RS_148734	0
2H	SCRI_RS_58904	0
2H	12_31446	0
2H	SCRI_RS_126952	1.18
2H	SCRI_RS_142851	1.18
2H	SCRI_RS_166806	1.18
2H	SCRI_RS_187638	1.18
2H	SCRI_RS_219333	1.18
2H	SCRI_RS_225720	1.18
2H	SCRI_RS_166816	1.93
2H	SCRI_RS_135585	2.03
2H	SCRI_RS_139708	2.13
2H	SCRI_RS_211923	2.13
2H	12_10718	2.44
2H	SCRI_RS_174552	2.8
2H	SCRI_RS_186847	2.8
2H	12_21415	4.06
2H	12_30132	4.06
2H	SCRI_RS_122	4.06
2H	11_10326	4.06
2H	SCRI_RS_146936	5.19
2H	SCRI_RS_156845	5.19
2H	SCRI_RS_184899	5.19
2H	SCRI_RS_232673	5.19
2H	SCRI_RS_192440	5.33
2H	SCRI_RS_184395	5.33
2H	SCRI_RS_1426	5.35
2H	SCRI_RS_238263	5.35
2H	12_11030	5.43
2H	SCRI_RS_168604	5.43
2H	SCRI_RS_169758	7.23
2H	SCRI_RS_187012	7.23
2H	12_10230	7.26
2H	SCRI_RS_158054	7.26
2H	SCRI_RS_192463	7.26
2H	SCRI_RS_227507	7.26
2H	11_11059	7.26
2H	SCRI_RS_88391	7.36
2H	SCRI_RS_165540	7.36
2H	12_30424	7.75
2H	SCRI_RS_177330	8.24
2H	SCRI_RS_224641	8.24
2H	11_21377	8.46
2H	12_10592	8.46
2H	12_30781	8.46
2H	SCRI_RS_103515	9.06
2H	SCRI_RS_135391	9.06
2H	SCRI_RS_162694	9.06
2H	SCRI_RS_164876	9.06
2H	SCRI_RS_196276	9.06
2H	SCRI_RS_237918	9.06
2H	SCRI_RS_141753	9.06
2H	11_20563	9.41
2H	SCRI_RS_206761	9.41
2H	SCRI_RS_10642	10
2H	SCRI_RS_128813	10
2H	SCRI_RS_136200	10
2H	SCRI_RS_163314	10
2H	SCRI_RS_165171	10
2H	SCRI_RS_167823	10
2H	SCRI_RS_173486	10
2H	SCRI_RS_175550	10
2H	12_10430	10.19
2H	12_31497	10.19
2H	SCRI_RS_173604	10.19
2H	SCRI_RS_193452	10.19
2H	11_20562	10.2
2H	12_10502	10.2
2H	SCRI_RS_197395	10.2
2H	12_30775	10.42
2H	SCRI_RS_149407	10.49
2H	SCRI_RS_659	10.49
2H	12_30155	10.5
2H	SCRI_RS_605	10.63
2H	SCRI_RS_144545	11.69
2H	SCRI_RS_209516	11.69
2H	SCRI_RS_160747	11.71
2H	SCRI_RS_166237	11.71
2H	SCRI_RS_81464	11.71
2H	11_21416	11.71
2H	12_30631	11.71
2H	SCRI_RS_152744	13.49
2H	11_20107	13.98
2H	SCRI_RS_108647	13.98
2H	SCRI_RS_139979	13.98
2H	SCRI_RS_909	13.98
2H	SCRI_RS_133377	14.22
2H	SCRI_RS_13476	14.22
2H	SCRI_RS_146997	14.22
2H	SCRI_RS_182210	14.22
2H	SCRI_RS_190048	14.22
2H	SCRI_RS_106759	14.58
2H	SCRI_RS_168461	14.58
2H	SCRI_RS_173947	14.62
2H	11_20724	14.62
2H	SCRI_RS_154030	14.76
2H	SCRI_RS_136287	15.14
2H	SCRI_RS_150792	15.14
2H	SCRI_RS_159158	15.14
2H	SCRI_RS_159503	15.14
2H	SCRI_RS_182947	15.14
2H	SCRI_RS_188511	15.14
2H	SCRI_RS_192552	15.14
2H	SCRI_RS_204158	15.14
2H	SCRI_RS_141771	15.59
2H	SCRI_RS_151082	15.59
2H	SCRI_RS_167465	15.59
2H	SCRI_RS_194134	15.59
2H	SCRI_RS_213799	15.59
2H	12_31284	15.62
2H	SCRI_RS_149462	15.62
2H	SCRI_RS_171185	15.62
2H	SCRI_RS_179003	15.62
2H	SCRI_RS_188987	15.62
2H	SCRI_RS_196864	15.62
2H	SCRI_RS_202814	15.62
2H	11_11040	15.78
2H	11_10943	16.88
2H	SCRI_RS_157480	17.02
2H	SCRI_RS_231057	17.04
2H	SCRI_RS_141564	18.57
2H	SCRI_RS_231806	18.57
2H	11_20112	19.06
2H	SCRI_RS_226348	19.06
2H	SCRI_RS_153672	19.29
2H	SCRI_RS_196316	19.37
2H	SCRI_RS_211637	19.37
2H	SCRI_RS_210172	20.09
2H	12_10777	20.09
2H	SCRI_RS_153798	20.09
2H	11_10180	20.09
2H	SCRI_RS_145190	20.15
2H	SCRI_RS_196192	20.15
2H	SCRI_RS_205712	20.15
2H	12_30145	20.22
2H	SCRI_RS_195810	21.09
2H	SCRI_RS_155957	21.25
2H	12_30870	21.34
2H	BK_12	21.34
2H	BK_13	21.34
2H	BK_14	21.34
2H	BK_15	21.34
2H	BK_16	21.34
2H	SCRI_RS_128162	21.78
2H	SCRI_RS_153226	21.78
2H	SCRI_RS_154683	21.78
2H	SCRI_RS_159228	21.78
2H	SCRI_RS_185319	21.78
2H	SCRI_RS_192386	21.78
2H	SCRI_RS_194812	21.78
2H	SCRI_RS_198643	21.78
2H	SCRI_RS_231889	21.78
2H	SCRI_RS_66401	21.78
2H	SCRI_RS_188893	24.6
2H	SCRI_RS_196202	24.89
2H	12_30871	24.97
2H	12_30872	24.97
2H	11_21015	25.6
2H	12_10880	25.6
2H	11_20394	26.23
2H	SCRI_RS_173164	26.23
2H	SCRI_RS_182270	26.27
2H	11_10216	26.27
2H	SCRI_RS_115905	27.14
2H	11_21366	27.14
2H	11_21261	27.14
2H	11_21265	27.14
2H	11_21187	27.79
2H	12_20593	28.69
2H	11_10307	30.06
2H	12_20368	30.06
2H	SCRI_RS_150590	30.29
2H	SCRI_RS_190423	30.29
2H	SCRI_RS_233272	30.8
2H	11_10787	31.55
2H	11_10891	31.78
2H	11_20864	31.99
2H	12_11452	33.46
2H	12_10847	33.88
2H	SCRI_RS_110647	35.97
2H	11_21304	36.58
2H	SCRI_RS_173697	36.58
2H	SCRI_RS_190819	36.58
2H	SCRI_RS_131218	36.93
2H	SCRI_RS_115892	38.02
2H	SCRI_RS_120529	38.02
2H	SCRI_RS_12516	38.02
2H	SCRI_RS_143250	38.02
2H	SCRI_RS_170337	38.02
2H	SCRI_RS_183655	38.02
2H	SCRI_RS_186387	38.02
2H	SCRI_RS_186448	38.02
2H	SCRI_RS_192783	38.02
2H	SCRI_RS_239882	38.02
2H	SCRI_RS_153693	38.16
2H	SCRI_RS_155612	38.16
2H	SCRI_RS_7026	39.07
2H	SCRI_RS_140819	39.37
2H	SCRI_RS_147371	39.37
2H	11_10178	39.67
2H	12_21461	39.67
2H	12_30420	40.33
2H	SCRI_RS_167605	41
2H	SCRI_RS_182408	41
2H	SCRI_RS_197357	41
2H	SCRI_RS_215080	41
2H	11_20173	41.18
2H	12_21049	41.36
2H	12_20326	41.65
2H	11_10987	41.76
2H	SCRI_RS_10302	41.76
2H	SCRI_RS_182371	41.76
2H	SCRI_RS_192657	41.76
2H	SCRI_RS_207162	41.76
2H	11_10525	41.88
2H	SCRI_RS_134823	42.16
2H	11_11073	42.5
2H	SCRI_RS_165427	42.5
2H	11_10399	42.78
2H	12_30657	42.8
2H	SCRI_RS_158687	42.8
2H	11_21153	43.02
2H	12_10296	43.2
2H	SCRI_RS_183558	43.2
2H	11_10837	43.21
2H	11_10919	43.21
2H	11_10648	43.51
2H	SCRI_RS_135590	43.78
2H	SCRI_RS_186954	43.78
2H	12_30029	43.91
2H	12_30432	43.91
2H	SCRI_RS_125793	43.91
2H	11_21338	43.91
2H	12_11493	43.91
2H	12_10715	43.91
2H	12_30748	44.57
2H	SCRI_RS_174935	44.57
2H	SCRI_RS_5552	44.57
2H	SCRI_RS_41263	44.98
2H	11_10342	45.01
2H	12_30379	45.97
2H	12_10486	46.67
2H	12_30491	46.67
2H	SCRI_RS_207244	46.89
2H	SCRI_RS_78277	46.89
2H	SCRI_RS_89851	46.89
2H	SCRI_RS_181166	46.89
2H	SCRI_RS_171029	46.91
2H	SCRI_RS_206586	47.17
2H	SCRI_RS_81287	47.47
2H	SCRI_RS_194318	47.51
2H	SCRI_RS_212932	47.53
2H	SCRI_RS_137093	47.65
2H	12_11340	47.71
2H	12_20281	47.71
2H	SCRI_RS_120182	47.82
2H	SCRI_RS_135619	47.93
2H	SCRI_RS_215094	47.93
2H	11_11061	48.16
2H	12_30703	48.16
2H	SCRI_RS_141655	48.82
2H	SCRI_RS_154981	48.82
2H	SCRI_RS_177405	48.82
2H	SCRI_RS_206245	48.82
2H	SCRI_RS_229103	48.82
2H	11_11505	49.04
2H	SCRI_RS_152206	49.51
2H	11_10084	49.51
2H	12_10643	49.51
2H	SCRI_RS_221843	50.2
2H	SCRI_RS_8366	50.2
2H	11_11054	50.35
2H	11_20131	51.44
2H	12_10156	51.44
2H	12_11428	51.44
2H	SCRI_RS_166431	51.68
2H	SCRI_RS_163305	52.57
2H	12_20688	52.63
2H	12_10219	53.22
2H	SCRI_RS_174444	53.22
2H	SCRI_RS_140673	53.24
2H	11_20929	53.6
2H	11_10422	53.81
2H	11_11302	53.81
2H	12_31169	53.95
2H	SCRI_RS_158928	54.35
2H	SCRI_RS_173531	54.35
2H	SCRI_RS_198460	54.35
2H	11_10638	54.46
2H	12_11131	54.46
2H	SCRI_RS_14448	54.49
2H	11_10234	54.75
2H	11_10297	54.75
2H	11_10498	54.75
2H	11_21005	54.75
2H	12_31127	54.88
2H	12_30691	55.35
2H	SCRI_RS_167564	55.35
2H	11_20674	55.65
2H	11_10996	55.67
2H	11_11400	55.67
2H	SCRI_RS_86605	55.77
2H	11_21096	56.05
2H	SCRI_RS_126877	56.62
2H	SCRI_RS_198148	56.62
2H	SCRI_RS_212484	56.62
2H	SCRI_RS_238424	56.75
2H	11_20387	56.75
2H	SCRI_RS_14801	57.39
2H	SCRI_RS_201800	57.39
2H	SCRI_RS_237094	57.39
2H	SCRI_RS_239231	57.39
2H	12_30338	57.66
2H	11_21388	57.66
2H	12_30259	57.66
2H	SCRI_RS_10398	57.66
2H	SCRI_RS_154617	57.66
2H	SCRI_RS_6792	57.66
2H	12_30487	57.66
2H	12_31408	57.66
2H	SCRI_RS_34836	57.66
2H	11_10733	57.66
2H	12_31474	57.66
2H	11_10325	57.76
2H	SCRI_RS_12752	57.99
2H	11_11015	58.06
2H	11_20602	58.06
2H	11_10147	58.06
2H	12_20235	58.17
2H	SCRI_RS_155546	58.17
2H	SCRI_RS_186443	58.17
2H	12_11272	58.17
2H	SCRI_RS_144592	58.17
2H	SCRI_RS_151576	58.2
2H	SCRI_RS_158649	58.2
2H	SCRI_RS_231782	58.2
2H	SCRI_RS_182050	58.2
2H	SCRI_RS_125325	58.6
2H	SCRI_RS_206569	58.6
2H	12_30195	58.66
2H	11_20891	58.66
2H	12_10927	58.66
2H	12_20917	58.66
2H	12_30251	58.66
2H	SCRI_RS_187110	58.67
2H	SCRI_RS_206492	58.67
2H	SCRI_RS_6099	58.67
2H	SCRI_RS_122681	59.35
2H	SCRI_RS_147210	59.35
2H	SCRI_RS_151535	59.35
2H	SCRI_RS_175065	59.35
2H	SCRI_RS_224846	59.35
2H	12_30209	59.36
2H	SCRI_RS_199897	59.64
2H	SCRI_RS_199898	59.64
2H	SCRI_RS_218303	59.64
2H	SCRI_RS_106444	59.87
2H	12_11163	60.02
2H	11_10997	60.02
2H	12_10558	60.02
2H	12_30557	60.02
2H	11_11101	60.65
2H	SCRI_RS_177142	60.65
2H	SCRI_RS_184121	60.7
2H	SCRI_RS_169913	60.72
2H	SCRI_RS_195938	60.72
2H	SCRI_RS_151921	60.72
2H	SCRI_RS_30973	60.72
2H	SCRI_RS_41905	60.72
2H	SCRI_RS_142400	60.72
2H	SCRI_RS_158123	60.72
2H	SCRI_RS_238404	60.72
2H	12_31288	60.72
2H	SCRI_RS_194216	60.72
2H	SCRI_RS_198865	60.72
2H	11_21286	60.72
2H	12_10330	60.72
2H	12_30853	60.72
2H	11_10602	60.72
2H	SCRI_RS_131325	60.72
2H	SCRI_RS_220718	60.72
2H	SCRI_RS_164343	60.72
2H	12_10948	60.72
2H	SCRI_RS_192676	60.72
2H	SCRI_RS_138888	60.72
2H	SCRI_RS_204254	60.72
2H	11_10679	60.72
2H	11_11046	60.72
2H	11_10070	60.72
2H	11_20500	60.72
2H	12_20181	60.72
2H	12_30582	60.72
2H	SCRI_RS_132200	60.72
2H	SCRI_RS_98229	60.72
2H	11_10796	60.72
2H	12_30772	60.72
2H	SCRI_RS_122395	60.72
2H	SCRI_RS_171237	60.72
2H	11_10750	60.72
2H	12_20861	60.72
2H	12_30224	60.72
2H	SCRI_RS_100469	60.72
2H	SCRI_RS_100476	60.72
2H	SCRI_RS_155808	60.72
2H	SCRI_RS_173857	60.72
2H	SCRI_RS_185595	60.72
2H	SCRI_RS_224512	60.72
2H	12_30634	60.72
2H	12_10485	60.72
2H	11_11133	60.72
2H	11_20631	60.91
2H	SCRI_RS_13952	60.91
2H	SCRI_RS_167713	60.91
2H	SCRI_RS_189408	60.91
2H	SCRI_RS_34413	60.91
2H	SCRI_RS_193756	60.91
2H	SCRI_RS_196323	60.91
2H	SCRI_RS_219740	60.91
2H	SCRI_RS_170235	60.91
2H	SCRI_RS_55840	60.91
2H	SCRI_RS_235694	60.91
2H	SCRI_RS_159	60.94
2H	SCRI_RS_214410	60.94
2H	SCRI_RS_219675	60.94
2H	SCRI_RS_137018	61.01
2H	SCRI_RS_160277	61.01
2H	11_10012	61.01
2H	11_10624	61.01
2H	11_20039	61.01
2H	11_20417	61.01
2H	12_10474	61.01
2H	12_30828	61.01
2H	SCRI_RS_206358	61.01
2H	SCRI_RS_228442	61.01
2H	SCRI_RS_87934	61.01
2H	12_10883	61.01
2H	12_11288	61.01
2H	12_11369	61.01
2H	12_20196	61.01
2H	12_20878	61.01
2H	12_21337	61.01
2H	12_30068	61.01
2H	12_30206	61.01
2H	12_31189	61.01
2H	12_31218	61.01
2H	SCRI_RS_130072	61.01
2H	SCRI_RS_135468	61.01
2H	SCRI_RS_175300	61.01
2H	SCRI_RS_194640	61.01
2H	11_10947	61.01
2H	11_11211	61.01
2H	11_10358	61.01
2H	11_20251	61.01
2H	11_20476	61.01
2H	11_20905	61.01
2H	12_31194	61.01
2H	SCRI_RS_103572	61.01
2H	SCRI_RS_109192	61.01
2H	SCRI_RS_122871	61.01
2H	SCRI_RS_131936	61.01
2H	SCRI_RS_135633	61.01
2H	SCRI_RS_136233	61.01
2H	SCRI_RS_141874	61.01
2H	SCRI_RS_146010	61.01
2H	SCRI_RS_151430	61.01
2H	SCRI_RS_153174	61.01
2H	SCRI_RS_162700	61.01
2H	SCRI_RS_167882	61.01
2H	SCRI_RS_180032	61.01
2H	SCRI_RS_222933	61.01
2H	SCRI_RS_231746	61.01
2H	12_10035	61.01
2H	11_10194	61.01
2H	11_10243	61.01
2H	11_11384	61.01
2H	12_10640	61.01
2H	12_20335	61.01
2H	11_10632	61.01
2H	11_20442	61.01
2H	12_31256	61.01
2H	SCRI_RS_171045	61.01
2H	11_20585	61.01
2H	12_11278	61.01
2H	12_30108	61.01
2H	12_30275	61.01
2H	12_30724	61.01
2H	SCRI_RS_137710	61.01
2H	SCRI_RS_141789	61.01
2H	SCRI_RS_206529	61.01
2H	SCRI_RS_216129	61.01
2H	11_10909	61.01
2H	SCRI_RS_144776	61.01
2H	SCRI_RS_213826	61.01
2H	11_20374	61.01
2H	11_10692	61.01
2H	11_20438	61.01
2H	SCRI_RS_116920	61.01
2H	SCRI_RS_127347	61.01
2H	SCRI_RS_132839	61.01
2H	SCRI_RS_177375	61.01
2H	SCRI_RS_191136	61.01
2H	SCRI_RS_222769	61.01
2H	SCRI_RS_4969	61.01
2H	11_21166	61.01
2H	SCRI_RS_107754	61.01
2H	SCRI_RS_143213	61.01
2H	11_21110	61.01
2H	SCRI_RS_4802	61.01
2H	12_30179	61.01
2H	11_11178	61.01
2H	SCRI_RS_196026	61.01
2H	SCRI_RS_134925	61.01
2H	SCRI_RS_155067	61.01
2H	SCRI_RS_157207	61.01
2H	11_10191	61.01
2H	11_20532	61.01
2H	SCRI_RS_145117	61.01
2H	SCRI_RS_151459	61.01
2H	SCRI_RS_208320	61.01
2H	SCRI_RS_213718	61.01
2H	SCRI_RS_222778	61.01
2H	SCRI_RS_194604	61.01
2H	SCRI_RS_208799	61.01
2H	11_10685	61.01
2H	11_11354	61.01
2H	11_20032	61.01
2H	11_20390	61.01
2H	11_20458	61.01
2H	11_20887	61.01
2H	12_11504	61.01
2H	12_30265	61.01
2H	12_30561	61.01
2H	SCRI_RS_130961	61.01
2H	SCRI_RS_144891	61.01
2H	SCRI_RS_145617	61.01
2H	SCRI_RS_147644	61.01
2H	SCRI_RS_154657	61.01
2H	SCRI_RS_162917	61.01
2H	SCRI_RS_174318	61.01
2H	SCRI_RS_175307	61.01
2H	SCRI_RS_185513	61.01
2H	SCRI_RS_186840	61.01
2H	SCRI_RS_188255	61.01
2H	SCRI_RS_206881	61.01
2H	SCRI_RS_206891	61.01
2H	SCRI_RS_220670	61.01
2H	SCRI_RS_233449	61.01
2H	SCRI_RS_235063	61.01
2H	SCRI_RS_236995	61.01
2H	SCRI_RS_240011	61.01
2H	SCRI_RS_139883	61.01
2H	SCRI_RS_142418	61.01
2H	11_20160	61.01
2H	11_20669	61.01
2H	11_20862	61.01
2H	11_21399	61.01
2H	12_10099	61.01
2H	12_30042	61.01
2H	SCRI_RS_117951	61.01
2H	SCRI_RS_136740	61.01
2H	SCRI_RS_138697	61.01
2H	SCRI_RS_156028	61.01
2H	SCRI_RS_165669	61.01
2H	SCRI_RS_171203	61.01
2H	SCRI_RS_176173	61.01
2H	SCRI_RS_184218	61.01
2H	SCRI_RS_230984	61.01
2H	SCRI_RS_231725	61.01
2H	SCRI_RS_232669	61.01
2H	11_10317	61.01
2H	SCRI_RS_100054	61.01
2H	SCRI_RS_119479	61.01
2H	SCRI_RS_12448	61.01
2H	SCRI_RS_133246	61.01
2H	SCRI_RS_140618	61.01
2H	SCRI_RS_165574	61.01
2H	SCRI_RS_185710	61.01
2H	SCRI_RS_186769	61.01
2H	SCRI_RS_195208	61.01
2H	SCRI_RS_197997	61.01
2H	12_21288	61.01
2H	12_31110	61.01
2H	12_31175	61.01
2H	SCRI_RS_11204	61.01
2H	SCRI_RS_125516	61.01
2H	SCRI_RS_134606	61.01
2H	SCRI_RS_1502	61.01
2H	SCRI_RS_153880	61.01
2H	SCRI_RS_167594	61.01
2H	SCRI_RS_173145	61.01
2H	SCRI_RS_182959	61.01
2H	SCRI_RS_196078	61.01
2H	SCRI_RS_200879	61.01
2H	SCRI_RS_231897	61.01
2H	SCRI_RS_202786	61.01
2H	SCRI_RS_219152	61.01
2H	SCRI_RS_12492	61.01
2H	SCRI_RS_161169	61.01
2H	SCRI_RS_236164	61.01
2H	SCRI_RS_155367	61.01
2H	SCRI_RS_172109	61.01
2H	SCRI_RS_83731	61.01
2H	SCRI_RS_237688	61.01
2H	SCRI_RS_168400	61.01
2H	SCRI_RS_13639	61.04
2H	SCRI_RS_186664	61.06
2H	11_11206	61.06
2H	SCRI_RS_144583	61.06
2H	SCRI_RS_173525	61.2
2H	11_20922	61.44
2H	12_20424	61.44
2H	SCRI_RS_162413	61.44
2H	SCRI_RS_212940	61.44
2H	11_10436	61.97
2H	12_11324	61.97
2H	12_30323	61.97
2H	SCRI_RS_173757	62
2H	11_11430	62.87
2H	12_11316	62.87
2H	SCRI_RS_145381	62.99
2H	SCRI_RS_146785	62.99
2H	12_10545	63.11
2H	SCRI_RS_153531	63.21
2H	11_21144	63.21
2H	12_21476	63.53
2H	12_31252	63.53
2H	SCRI_RS_156063	63.98
2H	SCRI_RS_179253	63.98
2H	SCRI_RS_147810	64.01
2H	11_21094	64.28
2H	12_11121	65.59
2H	SCRI_RS_163975	65.97
2H	SCRI_RS_59851	66.6
2H	11_11072	66.74
2H	SCRI_RS_198848	66.8
2H	SCRI_RS_120672	66.97
2H	SCRI_RS_155470	66.97
2H	SCRI_RS_180983	66.97
2H	SCRI_RS_201814	66.97
2H	SCRI_RS_209969	66.97
2H	SCRI_RS_236716	66.97
2H	11_20690	67.52
2H	SCRI_RS_165145	68.5
2H	11_10651	68.8
2H	12_11096	68.9
2H	SCRI_RS_110043	68.9
2H	11_20528	68.92
2H	11_10265	69.12
2H	11_21258	69.34
2H	SCRI_RS_150266	69.49
2H	SCRI_RS_6727	69.69
2H	SCRI_RS_173752	70.1
2H	SCRI_RS_16024	70.29
2H	SCRI_RS_16995	70.29
2H	11_20947	70.29
2H	12_10650	70.29
2H	11_21078	70.29
2H	12_10719	70.29
2H	SCRI_RS_206677	70.29
2H	SCRI_RS_221992	70.29
2H	SCRI_RS_73	70.29
2H	SCRI_RS_9469	70.29
2H	SCRI_RS_156871	70.29
2H	12_31394	70.29
2H	11_20960	70.29
2H	11_21156	70.29
2H	12_30674	70.29
2H	11_10952	70.29
2H	SCRI_RS_150	70.29
2H	12_20985	70.29
2H	SCRI_RS_207224	70.29
2H	11_10017	70.29
2H	11_20734	70.99
2H	11_10407	71.42
2H	11_20833	71.42
2H	11_21251	71.42
2H	12_30205	71.42
2H	12_31020	71.42
2H	12_31021	71.42
2H	11_21205	71.96
2H	12_10717	71.96
2H	11_20667	72.68
2H	SCRI_RS_172667	73.09
2H	SCRI_RS_207237	73.09
2H	SCRI_RS_182079	73.41
2H	11_10196	73.47
2H	SCRI_RS_50957	73.47
2H	11_10818	73.47
2H	12_30696	73.47
2H	SCRI_RS_7517	73.47
2H	BK_10	73.47
2H	SCRI_RS_145193	73.47
2H	SCRI_RS_157647	73.47
2H	SCRI_RS_219568	73.61
2H	SCRI_RS_220533	73.61
2H	SCRI_RS_214114	73.71
2H	11_11402	73.94
2H	12_31383	73.94
2H	11_20419	74.5
2H	12_20489	75.11
2H	12_31445	75.21
2H	12_11449	75.56
2H	SCRI_RS_32156	75.74
2H	SCRI_RS_17898	76.11
2H	12_31293	76.31
2H	SCRI_RS_158444	76.68
2H	SCRI_RS_2273	76.68
2H	SCRI_RS_133539	76.69
2H	SCRI_RS_235874	76.71
2H	12_10859	76.89
2H	SCRI_RS_158285	76.89
2H	SCRI_RS_156323	77.01
2H	12_11274	77.01
2H	11_21242	77.81
2H	SCRI_RS_129857	77.81
2H	SCRI_RS_221795	77.81
2H	SCRI_RS_222844	77.81
2H	SCRI_RS_239742	77.81
2H	SCRI_RS_139193	77.81
2H	SCRI_RS_2961	78.24
2H	SCRI_RS_166540	78.31
2H	SCRI_RS_200291	78.31
2H	SCRI_RS_154398	78.34
2H	SCRI_RS_157347	78.34
2H	SCRI_RS_151898	78.34
2H	SCRI_RS_152855	78.34
2H	SCRI_RS_158295	78.34
2H	SCRI_RS_209461	78.34
2H	SCRI_RS_134812	78.43
2H	11_11435	78.66
2H	12_11388	78.66
2H	12_31398	78.66
2H	11_11214	78.94
2H	11_10823	79.01
2H	SCRI_RS_235860	79.11
2H	SCRI_RS_171032	79.97
2H	11_20340	79.97
2H	SCRI_RS_4930	79.97
2H	SCRI_RS_184441	79.97
2H	SCRI_RS_133327	79.97
2H	SCRI_RS_150663	79.97
2H	11_20699	80.09
2H	SCRI_RS_164330	80.15
2H	11_10287	82.17
2H	SCRI_RS_91810	82.17
2H	SCRI_RS_137263	82.27
2H	SCRI_RS_165473	82.27
2H	SCRI_RS_196853	82.27
2H	SCRI_RS_88704	82.27
2H	12_30896	82.27
2H	SCRI_RS_154203	82.27
2H	SCRI_RS_171038	82.87
2H	SCRI_RS_221886	83.13
2H	11_11533	83.17
2H	SCRI_RS_13565	83.17
2H	SCRI_RS_192398	83.17
2H	SCRI_RS_3376	83.17
2H	12_31205	83.17
2H	11_10786	83.23
2H	11_11100	85.21
2H	11_20781	85.21
2H	SCRI_RS_180028	85.73
2H	SCRI_RS_10670	86.02
2H	SCRI_RS_128449	86.02
2H	SCRI_RS_156090	86.02
2H	SCRI_RS_160616	86.02
2H	SCRI_RS_160833	86.02
2H	SCRI_RS_172648	86.02
2H	SCRI_RS_198601	86.02
2H	SCRI_RS_198603	86.02
2H	SCRI_RS_211281	86.02
2H	SCRI_RS_235221	86.02
2H	SCRI_RS_126439	86.02
2H	SCRI_RS_165463	86.02
2H	11_10475	86.26
2H	SCRI_RS_159462	86.26
2H	SCRI_RS_179213	86.26
2H	SCRI_RS_185505	86.26
2H	SCRI_RS_188339	86.26
2H	SCRI_RS_208760	86.26
2H	SCRI_RS_182039	86.26
2H	SCRI_RS_189489	86.26
2H	12_10936	86.3
2H	12_31424	86.3
2H	SCRI_RS_119261	86.47
2H	SCRI_RS_157236	86.47
2H	SCRI_RS_174800	86.47
2H	SCRI_RS_176114	86.47
2H	SCRI_RS_211894	86.47
2H	SCRI_RS_138463	86.54
2H	SCRI_RS_183984	86.54
2H	11_21037	86.54
2H	SCRI_RS_219074	86.54
2H	11_21351	86.54
2H	12_10969	86.54
2H	SCRI_RS_155456	86.54
2H	SCRI_RS_162798	86.54
2H	SCRI_RS_237481	86.54
2H	SCRI_RS_118068	86.54
2H	12_30897	88.37
2H	12_30900	88.37
2H	12_30901	88.37
2H	11_21136	90.28
2H	11_21245	90.7
2H	11_11058	91.36
2H	11_10214	91.54
2H	SCRI_RS_2911	91.54
2H	SCRI_RS_116694	91.54
2H	SCRI_RS_177251	91.63
2H	SCRI_RS_157140	91.76
2H	SCRI_RS_165795	91.76
2H	SCRI_RS_203799	91.76
2H	SCRI_RS_237763	91.76
2H	12_20793	92.11
2H	11_20080	93.4
2H	SCRI_RS_129223	93.4
2H	SCRI_RS_144778	93.4
2H	SCRI_RS_155722	93.4
2H	SCRI_RS_165509	93.4
2H	SCRI_RS_218338	93.4
2H	SCRI_RS_237921	93.4
2H	SCRI_RS_7211	93.4
2H	SCRI_RS_222876	93.44
2H	12_10649	94.8
2H	SCRI_RS_115690	94.8
2H	SCRI_RS_135112	94.8
2H	SCRI_RS_142621	94.8
2H	SCRI_RS_160618	94.8
2H	11_10138	94.8
2H	SCRI_RS_196270	94.99
2H	SCRI_RS_171287	95.03
2H	12_30216	95.15
2H	SCRI_RS_195164	95.17
2H	11_21175	95.64
2H	12_11285	95.88
2H	SCRI_RS_154713	95.88
2H	SCRI_RS_162641	95.88
2H	SCRI_RS_196862	95.88
2H	11_11307	96
2H	11_20086	96.02
2H	SCRI_RS_170162	96.02
2H	11_21007	96.02
2H	12_10735	96.08
2H	SCRI_RS_140972	96.08
2H	SCRI_RS_148298	96.08
2H	SCRI_RS_229078	96.08
2H	SCRI_RS_239916	96.08
2H	SCRI_RS_4931	96.08
2H	SCRI_RS_149601	97.08
2H	SCRI_RS_209354	97.18
2H	SCRI_RS_172220	99.27
2H	SCRI_RS_147985	99.28
2H	SCRI_RS_157097	99.28
2H	SCRI_RS_142188	100.47
2H	SCRI_RS_221763	100.52
2H	SCRI_RS_196842	100.57
2H	11_11250	100.57
2H	SCRI_RS_209622	100.57
2H	11_10876	100.58
2H	SCRI_RS_159484	100.58
2H	SCRI_RS_160958	100.58
2H	SCRI_RS_173374	100.58
2H	SCRI_RS_135355	100.58
2H	SCRI_RS_194603	100.58
2H	SCRI_RS_150494	100.66
2H	SCRI_RS_135248	100.66
2H	11_20923	100.96
2H	11_10398	102.21
2H	12_30200	102.21
2H	SCRI_RS_152485	102.33
2H	SCRI_RS_157053	102.33
2H	11_10900	103.02
2H	11_11346	103.02
2H	12_21527	103.02
2H	SCRI_RS_153793	104.69
2H	12_31270	104.81
2H	SCRI_RS_187700	104.81
2H	11_20498	105.18
2H	11_21340	105.18
2H	12_30095	105.85
2H	12_30480	105.85
2H	11_11480	107.07
2H	11_11323	107.07
2H	12_30555	107.07
2H	SCRI_RS_116575	111.49
2H	SCRI_RS_173807	111.49
2H	SCRI_RS_178149	111.49
2H	SCRI_RS_179560	111.49
2H	SCRI_RS_204145	111.49
2H	SCRI_RS_205499	112.24
2H	SCRI_RS_238760	112.24
2H	SCRI_RS_139831	112.45
2H	SCRI_RS_88466	112.53
2H	11_20099	112.97
2H	12_30965	112.97
2H	SCRI_RS_123654	112.97
2H	SCRI_RS_200285	112.97
2H	SCRI_RS_20168	112.97
2H	SCRI_RS_206700	112.97
2H	SCRI_RS_227965	112.97
2H	SCRI_RS_4952	112.97
2H	SCRI_RS_620	112.97
2H	11_20955	113.37
2H	11_11236	113.48
2H	SCRI_RS_162690	113.48
2H	11_10977	113.48
2H	11_21184	113.48
2H	12_20436	113.48
2H	SCRI_RS_126890	113.48
2H	SCRI_RS_12996	113.48
2H	SCRI_RS_142313	113.48
2H	SCRI_RS_190073	113.48
2H	SCRI_RS_224624	113.48
2H	SCRI_RS_225624	113.48
2H	11_10429	113.58
2H	SCRI_RS_229405	113.58
2H	11_10731	113.58
2H	11_20064	113.58
2H	12_21396	113.58
2H	SCRI_RS_156045	113.58
2H	SCRI_RS_192384	113.58
2H	SCRI_RS_223885	113.58
2H	SCRI_RS_48964	113.58
2H	SCRI_RS_222093	113.66
2H	SCRI_RS_143727	113.66
2H	11_10988	113.66
2H	11_10128	113.66
2H	11_10263	113.66
2H	11_11043	113.68
2H	12_31402	113.68
2H	11_10352	113.68
2H	11_10989	113.68
2H	11_10990	113.68
2H	11_21238	113.68
2H	11_21507	113.68
2H	SCRI_RS_114164	113.68
2H	SCRI_RS_16799	113.68
2H	SCRI_RS_184090	113.68
2H	SCRI_RS_185363	113.68
2H	12_30459	113.69
2H	SCRI_RS_136379	113.69
2H	SCRI_RS_15050	113.69
2H	SCRI_RS_918	113.75
2H	SCRI_RS_15711	113.79
2H	SCRI_RS_151617	113.79
2H	SCRI_RS_219799	113.79
2H	SCRI_RS_128484	113.79
2H	12_20649	113.84
2H	SCRI_RS_149618	113.84
2H	SCRI_RS_171852	113.84
2H	SCRI_RS_15537	113.89
2H	SCRI_RS_153420	113.91
2H	SCRI_RS_158091	114.18
2H	SCRI_RS_183974	114.18
2H	SCRI_RS_610	114.62
2H	SCRI_RS_223905	114.62
2H	SCRI_RS_132586	114.97
2H	SCRI_RS_154176	114.97
2H	SCRI_RS_156220	115.08
2H	11_20182	115.45
2H	11_10538	115.68
2H	11_10707	115.68
2H	SCRI_RS_151349	115.68
2H	SCRI_RS_177125	115.68
2H	SCRI_RS_238606	115.68
2H	12_31095	115.82
2H	SCRI_RS_173131	115.82
2H	SCRI_RS_164608	115.98
2H	SCRI_RS_160911	116.17
2H	11_11118	116.58
2H	11_10404	116.6
2H	11_10916	116.92
2H	SCRI_RS_206070	116.92
2H	12_20989	118.09
2H	SCRI_RS_167694	118.1
2H	SCRI_RS_116193	118.18
2H	SCRI_RS_147052	118.18
2H	SCRI_RS_12444	118.18
2H	SCRI_RS_144379	118.18
2H	SCRI_RS_207327	118.18
2H	SCRI_RS_149429	118.88
2H	SCRI_RS_151556	119.08
2H	SCRI_RS_189936	119.3
2H	SCRI_RS_196100	119.3
2H	12_31264	119.39
2H	SCRI_RS_142593	119.39
2H	SCRI_RS_183064	119.39
2H	SCRI_RS_205571	119.39
2H	12_30598	119.48
2H	12_20295	119.59
2H	SCRI_RS_155161	120.38
2H	SCRI_RS_174214	120.38
2H	SCRI_RS_235261	120.38
2H	11_10092	120.38
2H	11_20511	120.38
2H	11_21220	120.38
2H	SCRI_RS_155689	120.38
2H	SCRI_RS_218275	121.84
2H	12_10739	122
2H	12_30636	122.16
2H	SCRI_RS_166987	122.16
2H	12_30152	122.26
2H	SCRI_RS_129177	122.26
2H	SCRI_RS_159243	122.26
2H	SCRI_RS_205807	122.26
2H	SCRI_RS_129178	122.26
2H	SCRI_RS_130279	122.3
2H	SCRI_RS_173490	122.3
2H	SCRI_RS_200033	122.3
2H	SCRI_RS_227502	122.3
2H	SCRI_RS_209218	122.4
2H	SCRI_RS_162820	122.4
2H	SCRI_RS_218206	122.4
2H	SCRI_RS_130285	122.4
2H	SCRI_RS_166443	122.4
2H	SCRI_RS_210959	122.4
2H	12_31327	122.4
2H	SCRI_RS_154954	122.4
2H	SCRI_RS_168629	122.4
2H	SCRI_RS_206945	122.4
2H	SCRI_RS_224454	122.4
2H	SCRI_RS_7392	122.4
2H	SCRI_RS_143501	122.45
2H	SCRI_RS_708	122.45
2H	SCRI_RS_179555	122.48
2H	SCRI_RS_185665	122.48
2H	12_10151	122.56
2H	SCRI_RS_10930	122.56
2H	SCRI_RS_118062	122.56
2H	SCRI_RS_124313	122.56
2H	SCRI_RS_147230	122.56
2H	SCRI_RS_192711	122.56
2H	SCRI_RS_193100	122.56
2H	SCRI_RS_204022	122.56
2H	SCRI_RS_116197	122.9
2H	SCRI_RS_151129	123.58
2H	11_21440	123.7
2H	12_31406	124.04
2H	11_10780	124.74
2H	11_11365	125.75
2H	11_21315	125.75
2H	SCRI_RS_12934	125.84
2H	SCRI_RS_153811	126.15
2H	SCRI_RS_161030	126.15
2H	SCRI_RS_168451	126.15
2H	SCRI_RS_201870	126.15
2H	SCRI_RS_208901	126.15
2H	SCRI_RS_230672	126.15
2H	11_20711	126.2
2H	SCRI_RS_116226	126.63
2H	SCRI_RS_137825	126.63
2H	SCRI_RS_152659	126.63
2H	SCRI_RS_211291	126.98
2H	11_20141	127
2H	12_30097	127
2H	SCRI_RS_149273	127.89
2H	11_10446	128.4
2H	11_20480	128.4
2H	12_30310	128.4
2H	11_10656	128.95
2H	11_21088	128.95
2H	SCRI_RS_124541	128.95
2H	SCRI_RS_165373	128.95
2H	SCRI_RS_200949	128.95
2H	SCRI_RS_209551	128.95
2H	SCRI_RS_239674	128.95
2H	11_20366	128.95
2H	12_30690	128.95
2H	SCRI_RS_119426	128.95
2H	SCRI_RS_139840	128.95
2H	SCRI_RS_157504	128.95
2H	SCRI_RS_223119	128.95
2H	SCRI_RS_231021	128.95
2H	SCRI_RS_2552	128.95
2H	11_21125	128.95
2H	SCRI_RS_144102	128.95
2H	SCRI_RS_200388	128.95
2H	SCRI_RS_200406	128.95
2H	SCRI_RS_175783	128.95
2H	SCRI_RS_55841	128.95
2H	SCRI_RS_119513	128.95
2H	SCRI_RS_194202	129.15
2H	12_30678	129.66
2H	12_30695	129.72
2H	11_21406	130.38
2H	12_31100	130.38
2H	SCRI_RS_157929	130.6
2H	11_11486	130.89
2H	11_21370	130.89
2H	SCRI_RS_136969	131.19
2H	SCRI_RS_198883	131.19
2H	11_10109	131.42
2H	12_30041	131.42
2H	SCRI_RS_185506	131.97
2H	SCRI_RS_142314	132.16
2H	SCRI_RS_134241	132.56
2H	SCRI_RS_146812	132.56
2H	SCRI_RS_238605	132.56
2H	12_20183	132.74
2H	11_10376	132.96
2H	SCRI_RS_133224	132.96
2H	SCRI_RS_181112	133.26
2H	SCRI_RS_182627	133.26
2H	SCRI_RS_124301	133.26
2H	SCRI_RS_139719	133.26
2H	SCRI_RS_171523	133.26
2H	SCRI_RS_194089	133.26
2H	SCRI_RS_202469	133.26
2H	11_20895	133.56
2H	12_10579	133.74
2H	11_10065	135.08
2H	11_10383	135.08
2H	11_20215	135.08
2H	12_10164	135.08
2H	12_10472	135.08
2H	12_30942	135.08
2H	12_31268	135.08
2H	11_11227	135.14
2H	SCRI_RS_11645	136.06
2H	SCRI_RS_121952	136.06
2H	SCRI_RS_155734	136.06
2H	SCRI_RS_138045	136.8
2H	SCRI_RS_201191	136.8
2H	SCRI_RS_221939	136.98
2H	11_21142	136.98
2H	SCRI_RS_157555	138.91
2H	11_20715	139.63
2H	12_30106	139.63
2H	12_30396	139.63
2H	SCRI_RS_194195	140.14
2H	SCRI_RS_206266	140.14
2H	12_30341	140.17
2H	11_20590	140.57
2H	SCRI_RS_106163	140.57
2H	11_21274	140.57
2H	SCRI_RS_194198	140.57
2H	11_21181	140.67
2H	SCRI_RS_215471	140.67
2H	SCRI_RS_148699	141.17
2H	SCRI_RS_194441	141.55
2H	12_30248	141.74
2H	SCRI_RS_158072	141.87
2H	SCRI_RS_179544	141.87
2H	SCRI_RS_7219	141.91
2H	SCRI_RS_13322	142.26
2H	12_10915	142.3
2H	SCRI_RS_208720	143.11
2H	SCRI_RS_227994	143.11
2H	12_20027	143.39
2H	12_31461	143.39
2H	11_20494	143.39
2H	11_10826	143.39
2H	11_10625	143.42
2H	12_31209	143.44
2H	11_11262	143.47
2H	12_30914	143.47
2H	SCRI_RS_230951	143.47
2H	SCRI_RS_740	143.47
2H	12_10623	143.47
2H	SCRI_RS_163464	143.47
2H	SCRI_RS_194105	143.47
2H	SCRI_RS_604	143.47
2H	SCRI_RS_226193	144.18
2H	12_30352	144.48
2H	11_10566	144.58
2H	11_10551	144.58
2H	SCRI_RS_6458	145.2
2H	SCRI_RS_168471	145.3
2H	SCRI_RS_160474	145.3
2H	12_20518	145.94
2H	SCRI_RS_114969	145.94
2H	12_10447	145.99
2H	SCRI_RS_174071	145.99
2H	11_11023	146.38
2H	SCRI_RS_114673	146.38
2H	SCRI_RS_156155	146.48
2H	SCRI_RS_233261	146.48
2H	11_10714	146.49
2H	SCRI_RS_197269	146.49
2H	SCRI_RS_205658	146.49
2H	SCRI_RS_158242	146.49
2H	SCRI_RS_172784	146.49
2H	SCRI_RS_173446	146.49
2H	SCRI_RS_188579	146.49
2H	SCRI_RS_207309	146.49
2H	SCRI_RS_234587	146.49
2H	11_21250	146.68
2H	SCRI_RS_149592	146.68
2H	SCRI_RS_175074	146.68
2H	SCRI_RS_192591	146.68
2H	12_10950	146.68
2H	SCRI_RS_44952	146.68
2H	12_10487	146.68
2H	SCRI_RS_138320	146.68
2H	SCRI_RS_194079	146.78
2H	SCRI_RS_186925	147.03
2H	SCRI_RS_204274	147.03
2H	SCRI_RS_175216	147.03
2H	11_11380	147.13
2H	SCRI_RS_146068	147.13
2H	12_10181	147.52
2H	SCRI_RS_222342	147.52
2H	11_20994	147.52
2H	SCRI_RS_178751	147.52
2H	12_11368	147.52
2H	SCRI_RS_139073	147.63
2H	SCRI_RS_10006	147.63
2H	SCRI_RS_161636	147.63
2H	SCRI_RS_155544	148.22
2H	SCRI_RS_156819	148.36
2H	SCRI_RS_194653	148.36
2H	11_21346	148.36
2H	SCRI_RS_230497	148.36
2H	11_20293	148.36
2H	12_11383	148.36
2H	SCRI_RS_154135	148.36
2H	SCRI_RS_166430	148.36
2H	SCRI_RS_207267	148.36
2H	11_20943	149.07
2H	SCRI_RS_224822	149.07
2H	SCRI_RS_173017	149.35
2H	SCRI_RS_8420	149.35
2H	SCRI_RS_156013	149.35
2H	SCRI_RS_174037	149.35
2H	SCRI_RS_208292	149.35
2H	SCRI_RS_182277	149.35
2H	SCRI_RS_4691	149.35
2H	SCRI_RS_125484	149.63
2H	12_30823	151.41
2H	11_21436	151.41
2H	SCRI_RS_134343	151.41
2H	SCRI_RS_152664	151.71
2H	SCRI_RS_153293	151.71
2H	SCRI_RS_174077	151.71
2H	11_10791	151.71
2H	12_30822	151.71
2H	SCRI_RS_116590	151.71
2H	SCRI_RS_141500	151.78
2H	SCRI_RS_200267	151.78
2H	12_31128	151.84
2H	SCRI_RS_15119	152.21
2H	11_10072	152.34
2H	12_31527	152.34
2H	SCRI_RS_147278	152.34
2H	SCRI_RS_8347	152.34
2H	SCRI_RS_122297	152.34
2H	SCRI_RS_152274	152.34
2H	SCRI_RS_188722	152.34
2H	SCRI_RS_171198	152.34
2H	12_31506	153.33
2H	SCRI_RS_172136	153.33
2H	SCRI_RS_225633	153.33
2H	SCRI_RS_73620	153.33
2H	SCRI_RS_13450	153.33
2H	SCRI_RS_149233	153.33
2H	SCRI_RS_187318	153.33
2H	SCRI_RS_223188	153.33
2H	12_10937	153.66
2H	12_31300	153.66
2H	SCRI_RS_119718	154.12
2H	SCRI_RS_159526	154.19
2H	11_10770	154.72
2H	SCRI_RS_227482	154.83
2H	11_21099	155.32
2H	11_21453	155.32
2H	12_31180	155.32
2H	SCRI_RS_119848	155.32
2H	11_10329	155.32
2H	SCRI_RS_170209	155.42
2H	SCRI_RS_109266	156.62
2H	SCRI_RS_13386	156.62
2H	SCRI_RS_161281	156.62
2H	SCRI_RS_176159	156.62
2H	SCRI_RS_195051	156.62
2H	SCRI_RS_228319	156.62
2H	SCRI_RS_139737	156.65
2H	SCRI_RS_151056	156.65
2H	SCRI_RS_156075	156.65
2H	SCRI_RS_196748	156.65
2H	SCRI_RS_174051	156.65
2H	SCRI_RS_206020	156.65
2H	11_10085	156.72
2H	SCRI_RS_119279	156.72
2H	SCRI_RS_139106	156.72
2H	SCRI_RS_139115	156.72
2H	SCRI_RS_142982	156.72
2H	SCRI_RS_158043	156.72
2H	SCRI_RS_161224	156.72
2H	SCRI_RS_169956	156.72
2H	SCRI_RS_169970	156.72
2H	SCRI_RS_170753	156.72
2H	SCRI_RS_186760	156.72
2H	SCRI_RS_227525	156.72
2H	SCRI_RS_236521	156.72
2H	12_11050	156.72
2H	SCRI_RS_159171	156.72
2H	SCRI_RS_237016	156.72
2H	11_20561	156.72
2H	SCRI_RS_140931	156.72
2H	SCRI_RS_7940	156.72
2H	12_30240	156.72
2H	SCRI_RS_8418	156.72
2H	SCRI_RS_134252	156.86
2H	SCRI_RS_204579	156.86
2H	11_20681	156.86
2H	SCRI_RS_205992	156.86
2H	SCRI_RS_223897	156.86
2H	12_30378	156.86
2H	SCRI_RS_123364	156.86
2H	SCRI_RS_138848	156.86
2H	SCRI_RS_154153	156.86
2H	SCRI_RS_159536	156.86
2H	SCRI_RS_160512	156.86
2H	SCRI_RS_196604	156.86
2H	SCRI_RS_8637	156.86
2H	SCRI_RS_231015	156.86
2H	12_21496	156.86
2H	SCRI_RS_161538	156.86
2H	12_20775	156.86
2H	SCRI_RS_207084	156.86
2H	SCRI_RS_8671	156.86
2H	SCRI_RS_163112	156.86
2H	11_21414	156.94
2H	SCRI_RS_129821	156.94
2H	SCRI_RS_150519	156.94
2H	SCRI_RS_165239	156.94
2H	11_10101	157.79
3H	11_20858	0
3H	SCRI_RS_148646	0.92
3H	12_31428	0.92
3H	SCRI_RS_174383	0.92
3H	SCRI_RS_190002	0.92
3H	SCRI_RS_216164	0.92
3H	SCRI_RS_1804	1.02
3H	12_10103	2.52
3H	SCRI_RS_235332	3.46
3H	SCRI_RS_129935	3.5
3H	11_20797	3.91
3H	12_31448	4.51
3H	SCRI_RS_110189	4.51
3H	SCRI_RS_237545	4.51
3H	12_11434	4.86
3H	11_20159	4.86
3H	SCRI_RS_14819	4.86
3H	12_31230	5.06
3H	12_31487	6.98
3H	11_11453	7.08
3H	SCRI_RS_207228	7.08
3H	12_10173	7.11
3H	11_20252	7.11
3H	SCRI_RS_168529	7.64
3H	SCRI_RS_119379	7.64
3H	SCRI_RS_180343	7.64
3H	12_31409	7.76
3H	11_20953	7.76
3H	SCRI_RS_172351	7.99
3H	11_20529	9.21
3H	11_21398	9.21
3H	12_11005	9.21
3H	SCRI_RS_154670	9.97
3H	11_21190	9.97
3H	11_21027	10.09
3H	12_30297	10.09
3H	SCRI_RS_173491	10.13
3H	SCRI_RS_67208	10.13
3H	SCRI_RS_127822	10.23
3H	SCRI_RS_127903	10.23
3H	SCRI_RS_155475	10.26
3H	12_30818	10.26
3H	SCRI_RS_6682	10.38
3H	SCRI_RS_193258	10.38
3H	SCRI_RS_230638	10.38
3H	SCRI_RS_132388	10.41
3H	11_10112	10.66
3H	11_10886	10.66
3H	11_20976	10.66
3H	SCRI_RS_130264	10.66
3H	SCRI_RS_239142	10.66
3H	12_11310	10.66
3H	SCRI_RS_122224	10.66
3H	12_10792	11.44
3H	SCRI_RS_210101	11.44
3H	12_30915	11.81
3H	11_20595	11.84
3H	SCRI_RS_108971	12.43
3H	12_11095	13.99
3H	SCRI_RS_65533	15.37
3H	11_20172	15.68
3H	SCRI_RS_154700	15.68
3H	SCRI_RS_184857	15.68
3H	SCRI_RS_97417	15.68
3H	SCRI_RS_97418	15.68
3H	SCRI_RS_179462	16.1
3H	SCRI_RS_203454	16.1
3H	SCRI_RS_173917	17.04
3H	SCRI_RS_4564	17.04
3H	SCRI_RS_8644	17.04
3H	SCRI_RS_10191	17.18
3H	SCRI_RS_197216	17.18
3H	SCRI_RS_190015	18.5
3H	11_10565	19.18
3H	SCRI_RS_214280	19.18
3H	11_20742	19.18
3H	SCRI_RS_209312	19.24
3H	SCRI_RS_9614	19.33
3H	SCRI_RS_153718	19.33
3H	SCRI_RS_221814	19.33
3H	SCRI_RS_192352	19.36
3H	SCRI_RS_151808	19.36
3H	SCRI_RS_161041	19.36
3H	11_20982	22.24
3H	12_30192	23.17
3H	11_20552	23.87
3H	SCRI_RS_222975	24.35
3H	11_10559	24.35
3H	SCRI_RS_229894	24.35
3H	12_30920	24.35
3H	11_20794	25.66
3H	SCRI_RS_159598	26.05
3H	11_20968	26.46
3H	SCRI_RS_177084	26.46
3H	SCRI_RS_189757	26.46
3H	12_11237	26.46
3H	SCRI_RS_102971	26.7
3H	SCRI_RS_121895	26.7
3H	SCRI_RS_144410	26.7
3H	SCRI_RS_230052	26.7
3H	SCRI_RS_3748	26.7
3H	SCRI_RS_7929	26.7
3H	11_20455	27.12
3H	11_20607	30.31
3H	12_30571	30.31
3H	SCRI_RS_162639	30.58
3H	SCRI_RS_186444	30.58
3H	11_10026	31.54
3H	12_11527	31.54
3H	12_11414	31.68
3H	SCRI_RS_11012	32.17
3H	12_21268	33.34
3H	SCRI_RS_153732	33.34
3H	SCRI_RS_192875	33.34
3H	12_30431	33.48
3H	11_10672	35.15
3H	SCRI_RS_230486	36.5
3H	SCRI_RS_115423	36.7
3H	SCRI_RS_180821	36.7
3H	SCRI_RS_231261	36.7
3H	SCRI_RS_4528	36.7
3H	SCRI_RS_6922	36.7
3H	SCRI_RS_119697	36.85
3H	SCRI_RS_136959	36.85
3H	12_10968	36.85
3H	SCRI_RS_192449	36.85
3H	SCRI_RS_7983	36.85
3H	SCRI_RS_194531	36.85
3H	SCRI_RS_1627	37.43
3H	SCRI_RS_181164	37.43
3H	SCRI_RS_198852	37.43
3H	SCRI_RS_200512	37.43
3H	SCRI_RS_206030	37.43
3H	SCRI_RS_230475	37.43
3H	11_10710	37.45
3H	11_10825	37.45
3H	11_20410	37.45
3H	SCRI_RS_115045	37.45
3H	SCRI_RS_115969	37.45
3H	SCRI_RS_151299	37.45
3H	SCRI_RS_173721	37.45
3H	SCRI_RS_211943	37.45
3H	SCRI_RS_103614	37.64
3H	SCRI_RS_177984	37.64
3H	SCRI_RS_175314	37.65
3H	SCRI_RS_9439	37.65
3H	SCRI_RS_102837	37.71
3H	SCRI_RS_133685	37.71
3H	SCRI_RS_135333	37.71
3H	SCRI_RS_152682	37.71
3H	SCRI_RS_170626	37.71
3H	SCRI_RS_174979	37.71
3H	SCRI_RS_136252	37.71
3H	SCRI_RS_174984	37.75
3H	12_30953	37.85
3H	12_31298	38.03
3H	12_31159	38.3
3H	11_10863	39.35
3H	SCRI_RS_154747	39.35
3H	11_20193	39.35
3H	12_10114	39.35
3H	SCRI_RS_237761	39.35
3H	SCRI_RS_159002	39.57
3H	SCRI_RS_202424	39.57
3H	11_21145	40
3H	SCRI_RS_213686	40.34
3H	SCRI_RS_235084	40.34
3H	SCRI_RS_154769	40.48
3H	SCRI_RS_227903	40.51
3H	11_20647	41
3H	SCRI_RS_154973	41.04
3H	SCRI_RS_231185	41.04
3H	SCRI_RS_141171	43.99
3H	BK_24	44.05
3H	11_11002	44.87
3H	SCRI_RS_229693	45.28
3H	12_21506	45.5
3H	SCRI_RS_9584	45.63
3H	11_21259	45.92
3H	12_11117	45.92
3H	12_30785	46.07
3H	SCRI_RS_127994	46.07
3H	12_31475	46.57
3H	SCRI_RS_151254	46.57
3H	SCRI_RS_238649	46.57
3H	BK_06	46.57
3H	SCRI_RS_168541	46.57
3H	SCRI_RS_211268	46.57
3H	12_30064	47.17
3H	SCRI_RS_146425	47.17
3H	SCRI_RS_199987	47.17
3H	SCRI_RS_227793	47.17
3H	11_10601	47.23
3H	11_20356	47.39
3H	11_21101	47.39
3H	12_31009	47.39
3H	SCRI_RS_189054	47.71
3H	11_21189	47.77
3H	SCRI_RS_126627	47.82
3H	SCRI_RS_235603	47.82
3H	12_30991	47.92
3H	SCRI_RS_200297	47.92
3H	SCRI_RS_205999	47.92
3H	SCRI_RS_205711	48.07
3H	11_20719	48.71
3H	12_30609	48.71
3H	12_11069	48.8
3H	12_20264	48.8
3H	12_30467	48.8
3H	12_30721	48.8
3H	SCRI_RS_219247	49.07
3H	SCRI_RS_110693	49.09
3H	SCRI_RS_149207	49.09
3H	SCRI_RS_153735	49.09
3H	SCRI_RS_166122	49.09
3H	SCRI_RS_185596	49.09
3H	SCRI_RS_218235	49.09
3H	SCRI_RS_239787	49.09
3H	BK_08	49.32
3H	SCRI_RS_11338	49.32
3H	SCRI_RS_13433	49.32
3H	SCRI_RS_141081	49.32
3H	SCRI_RS_178915	49.32
3H	SCRI_RS_182479	49.32
3H	SCRI_RS_189045	49.32
3H	SCRI_RS_230096	49.32
3H	SCRI_RS_886	49.32
3H	11_21402	50.12
3H	11_21093	51.27
3H	12_30583	51.27
3H	12_20037	51.61
3H	12_11102	52.28
3H	11_20328	52.59
3H	12_10532	52.59
3H	12_30474	52.59
3H	12_30737	52.59
3H	12_31122	52.59
3H	SCRI_RS_226826	52.6
3H	SCRI_RS_170111	52.81
3H	SCRI_RS_153723	53.09
3H	SCRI_RS_157048	53.09
3H	SCRI_RS_239403	53.09
3H	SCRI_RS_165605	53.24
3H	SCRI_RS_193439	53.24
3H	11_10380	53.34
3H	11_20866	53.34
3H	11_21109	53.34
3H	11_21197	53.34
3H	12_11387	53.34
3H	12_11511	53.34
3H	12_30680	53.34
3H	12_30922	53.34
3H	12_30923	53.34
3H	12_31015	53.34
3H	SCRI_RS_175073	53.34
3H	SCRI_RS_204057	53.34
3H	SCRI_RS_11126	53.34
3H	SCRI_RS_142442	53.34
3H	SCRI_RS_169606	53.34
3H	SCRI_RS_173916	53.34
3H	SCRI_RS_222735	53.34
3H	SCRI_RS_65878	53.34
3H	SCRI_RS_196383	53.34
3H	SCRI_RS_121826	53.34
3H	SCRI_RS_159312	53.34
3H	SCRI_RS_164254	53.34
3H	SCRI_RS_186768	53.34
3H	SCRI_RS_219551	53.34
3H	SCRI_RS_109716	53.34
3H	SCRI_RS_128491	53.34
3H	SCRI_RS_17525	53.34
3H	SCRI_RS_187032	53.34
3H	SCRI_RS_191131	53.34
3H	SCRI_RS_213587	53.34
3H	SCRI_RS_126246	53.34
3H	SCRI_RS_188912	53.34
3H	SCRI_RS_84470	53.34
3H	SCRI_RS_141166	53.34
3H	11_11313	53.34
3H	12_30002	53.48
3H	SCRI_RS_103211	53.48
3H	SCRI_RS_114566	53.48
3H	SCRI_RS_132097	53.48
3H	SCRI_RS_136150	53.48
3H	SCRI_RS_150883	53.48
3H	SCRI_RS_152198	53.48
3H	SCRI_RS_155784	53.48
3H	SCRI_RS_156426	53.48
3H	SCRI_RS_166308	53.48
3H	SCRI_RS_174254	53.48
3H	SCRI_RS_182609	53.48
3H	SCRI_RS_184523	53.48
3H	SCRI_RS_204628	53.48
3H	SCRI_RS_214259	53.48
3H	SCRI_RS_220070	53.48
3H	SCRI_RS_7804	53.48
3H	SCRI_RS_9327	53.48
3H	12_10233	53.5
3H	11_11086	53.5
3H	11_11258	53.5
3H	11_21129	53.5
3H	12_30618	53.5
3H	12_30799	53.5
3H	SCRI_RS_128706	53.5
3H	SCRI_RS_186341	53.5
3H	SCRI_RS_207180	53.5
3H	SCRI_RS_223894	53.5
3H	SCRI_RS_138918	53.5
3H	SCRI_RS_181360	53.5
3H	SCRI_RS_6877	53.5
3H	SCRI_RS_137552	53.5
3H	SCRI_RS_157988	53.5
3H	SCRI_RS_165264	53.5
3H	SCRI_RS_196189	53.5
3H	SCRI_RS_212974	53.5
3H	SCRI_RS_213411	53.5
3H	SCRI_RS_213902	53.5
3H	SCRI_RS_221896	53.5
3H	SCRI_RS_239964	53.5
3H	SCRI_RS_2523	53.5
3H	SCRI_RS_906	53.5
3H	SCRI_RS_161083	53.5
3H	SCRI_RS_207066	53.5
3H	SCRI_RS_186504	53.5
3H	12_30299	53.5
3H	12_30622	53.5
3H	SCRI_RS_107178	53.5
3H	SCRI_RS_111312	53.5
3H	SCRI_RS_120315	53.5
3H	SCRI_RS_137201	53.5
3H	SCRI_RS_150370	53.5
3H	SCRI_RS_151545	53.5
3H	SCRI_RS_158967	53.5
3H	SCRI_RS_160464	53.5
3H	SCRI_RS_166244	53.5
3H	SCRI_RS_167084	53.5
3H	SCRI_RS_170765	53.5
3H	SCRI_RS_171453	53.5
3H	SCRI_RS_176264	53.5
3H	SCRI_RS_181347	53.5
3H	SCRI_RS_187465	53.5
3H	SCRI_RS_189204	53.5
3H	SCRI_RS_194233	53.5
3H	SCRI_RS_214796	53.5
3H	SCRI_RS_221644	53.5
3H	SCRI_RS_224702	53.5
3H	SCRI_RS_229167	53.5
3H	SCRI_RS_4728	53.5
3H	SCRI_RS_6959	53.5
3H	11_10365	53.54
3H	11_10437	53.54
3H	11_10966	53.54
3H	11_20890	53.54
3H	12_31012	53.54
3H	12_31372	53.54
3H	SCRI_RS_136921	53.54
3H	SCRI_RS_144038	53.54
3H	SCRI_RS_181847	53.54
3H	SCRI_RS_190876	53.54
3H	SCRI_RS_212864	53.54
3H	SCRI_RS_238625	53.54
3H	SCRI_RS_150800	53.54
3H	SCRI_RS_205949	53.54
3H	12_10391	53.54
3H	12_11265	53.54
3H	12_30262	53.54
3H	12_31008	53.54
3H	11_10620	53.64
3H	11_20002	53.64
3H	11_20288	53.64
3H	12_31214	53.64
3H	SCRI_RS_115769	53.64
3H	SCRI_RS_151799	53.64
3H	SCRI_RS_157545	53.64
3H	11_20102	53.64
3H	SCRI_RS_209249	53.64
3H	SCRI_RS_108473	53.64
3H	SCRI_RS_12851	53.64
3H	SCRI_RS_132190	53.64
3H	SCRI_RS_176023	53.64
3H	SCRI_RS_178900	53.64
3H	SCRI_RS_182282	53.64
3H	SCRI_RS_186923	53.64
3H	SCRI_RS_189167	53.64
3H	SCRI_RS_215085	53.64
3H	SCRI_RS_219354	53.64
3H	SCRI_RS_225537	53.64
3H	SCRI_RS_237716	53.64
3H	SCRI_RS_90637	53.64
3H	SCRI_RS_153477	53.71
3H	SCRI_RS_210160	53.78
3H	SCRI_RS_6793	53.78
3H	SCRI_RS_137787	53.79
3H	12_30153	53.79
3H	SCRI_RS_160256	53.79
3H	SCRI_RS_173348	53.79
3H	SCRI_RS_238114	53.79
3H	SCRI_RS_129291	53.96
3H	SCRI_RS_149481	53.96
3H	SCRI_RS_156525	53.96
3H	SCRI_RS_144369	54.02
3H	SCRI_RS_8664	54.02
3H	SCRI_RS_6729	54.58
3H	SCRI_RS_152212	54.83
3H	SCRI_RS_204401	54.83
3H	11_10925	54.91
3H	11_11125	54.91
3H	11_11283	54.91
3H	11_11530	54.91
3H	11_20486	54.91
3H	11_20583	54.91
3H	11_20801	54.91
3H	11_21435	54.91
3H	11_21472	54.91
3H	11_10224	55.56
3H	11_10349	55.56
3H	11_11099	55.56
3H	11_11337	55.56
3H	11_20439	55.56
3H	11_21062	55.56
3H	11_21147	55.56
3H	12_10784	55.56
3H	12_11295	55.56
3H	12_11482	55.56
3H	12_20591	55.56
3H	12_20721	55.56
3H	12_21411	55.56
3H	12_30039	55.56
3H	12_30130	55.56
3H	12_31502	55.56
3H	SCRI_RS_140648	56.1
3H	11_10008	56.15
3H	11_10011	56.15
3H	11_10137	56.15
3H	11_10328	56.15
3H	11_10456	56.15
3H	11_10926	56.15
3H	11_11124	56.15
3H	11_11501	56.15
3H	11_11502	56.15
3H	11_20333	56.15
3H	11_20428	56.15
3H	11_20796	56.15
3H	11_20856	56.15
3H	11_20970	56.15
3H	12_11518	56.15
3H	12_20574	56.15
3H	12_30126	56.15
3H	12_30318	56.15
3H	12_30809	56.15
3H	12_31010	56.15
3H	12_31014	56.15
3H	12_31017	56.15
3H	12_31281	56.15
3H	12_31368	56.15
3H	12_31484	56.24
3H	11_20444	56.8
3H	SCRI_RS_152172	57.22
3H	SCRI_RS_155168	57.22
3H	SCRI_RS_173186	57.22
3H	SCRI_RS_186102	57.22
3H	SCRI_RS_222102	57.22
3H	11_11401	57.34
3H	11_10225	57.69
3H	11_10653	57.69
3H	12_20108	57.69
3H	11_20995	58.24
3H	12_30009	58.24
3H	SCRI_RS_162838	58.3
3H	SCRI_RS_235588	58.48
3H	SCRI_RS_236448	58.6
3H	SCRI_RS_174324	58.99
3H	SCRI_RS_152371	59.1
3H	SCRI_RS_179400	59.13
3H	SCRI_RS_158616	59.28
3H	SCRI_RS_182328	59.33
3H	SCRI_RS_200042	59.35
3H	11_10005	59.55
3H	11_11016	59.55
3H	11_20276	59.55
3H	11_20325	59.55
3H	11_20639	59.55
3H	11_21511	59.55
3H	12_10678	59.55
3H	12_10850	59.55
3H	12_11429	59.55
3H	12_21064	59.55
3H	12_31011	59.55
3H	12_31016	59.55
3H	12_31393	59.55
3H	SCRI_RS_10387	59.71
3H	SCRI_RS_124201	59.71
3H	SCRI_RS_124607	59.71
3H	SCRI_RS_141109	59.71
3H	SCRI_RS_151680	59.71
3H	SCRI_RS_159661	59.71
3H	SCRI_RS_162862	59.71
3H	SCRI_RS_16934	59.71
3H	SCRI_RS_175771	59.71
3H	SCRI_RS_179275	59.71
3H	SCRI_RS_214807	59.71
3H	SCRI_RS_7385	59.71
3H	SCRI_RS_231801	60.33
3H	11_10728	60.5
3H	SCRI_RS_8249	60.9
3H	SCRI_RS_12836	61.1
3H	SCRI_RS_168665	61.1
3H	SCRI_RS_195934	61.33
3H	SCRI_RS_166310	61.38
3H	11_10158	61.59
3H	11_10281	61.59
3H	12_20863	61.59
3H	12_30829	61.59
3H	SCRI_RS_142939	61.96
3H	SCRI_RS_157479	61.96
3H	SCRI_RS_197825	61.96
3H	SCRI_RS_135581	62.15
3H	SCRI_RS_143331	62.15
3H	SCRI_RS_1799	62.15
3H	11_10373	62.26
3H	12_30594	62.34
3H	SCRI_RS_132345	62.34
3H	SCRI_RS_224335	62.34
3H	12_30008	62.75
3H	SCRI_RS_133415	62.75
3H	SCRI_RS_170182	62.75
3H	SCRI_RS_201987	62.75
3H	11_11191	63.56
3H	11_21120	63.56
3H	12_11084	63.56
3H	12_11150	63.56
3H	12_30088	63.56
3H	SCRI_RS_6231	63.81
3H	SCRI_RS_234006	63.87
3H	SCRI_RS_200508	64.02
3H	SCRI_RS_154396	64.05
3H	SCRI_RS_178907	64.05
3H	SCRI_RS_231031	64.05
3H	SCRI_RS_232895	64.05
3H	SCRI_RS_168538	64.16
3H	SCRI_RS_204130	64.16
3H	SCRI_RS_209237	64.16
3H	SCRI_RS_67	64.16
3H	SCRI_RS_127140	64.27
3H	SCRI_RS_157150	64.27
3H	SCRI_RS_164731	64.27
3H	SCRI_RS_171837	64.27
3H	SCRI_RS_230975	64.27
3H	SCRI_RS_174419	64.3
3H	SCRI_RS_179206	64.3
3H	SCRI_RS_220894	64.3
3H	SCRI_RS_226190	64.3
3H	SCRI_RS_152472	64.45
3H	SCRI_RS_210118	64.45
3H	SCRI_RS_214023	64.45
3H	SCRI_RS_220115	64.45
3H	SCRI_RS_220129	64.45
3H	SCRI_RS_107261	64.46
3H	SCRI_RS_130616	64.46
3H	SCRI_RS_137934	64.46
3H	SCRI_RS_182209	64.46
3H	SCRI_RS_211224	64.46
3H	SCRI_RS_238981	64.46
3H	11_20704	64.72
3H	11_21502	64.72
3H	SCRI_RS_130849	65.45
3H	SCRI_RS_175710	65.45
3H	11_10335	65.66
3H	11_10839	65.66
3H	12_11152	65.66
3H	12_30005	65.66
3H	SCRI_RS_145300	65.72
3H	SCRI_RS_158555	65.72
3H	SCRI_RS_69605	65.72
3H	SCRI_RS_9350	65.72
3H	11_20124	66.07
3H	SCRI_RS_177314	66.07
3H	SCRI_RS_144535	66.28
3H	SCRI_RS_235065	66.28
3H	12_11284	66.54
3H	12_20608	66.54
3H	12_30616	66.54
3H	12_30788	66.54
3H	SCRI_RS_223097	66.57
3H	SCRI_RS_10288	66.67
3H	SCRI_RS_13376	66.67
3H	SCRI_RS_1398	66.67
3H	SCRI_RS_161744	66.67
3H	SCRI_RS_167463	66.67
3H	SCRI_RS_182061	66.67
3H	SCRI_RS_187102	66.67
3H	SCRI_RS_204491	66.67
3H	SCRI_RS_220192	66.67
3H	SCRI_RS_225540	66.67
3H	SCRI_RS_4575	66.67
3H	SCRI_RS_139294	66.91
3H	SCRI_RS_187887	66.91
3H	SCRI_RS_224360	66.91
3H	SCRI_RS_138291	67.02
3H	SCRI_RS_139449	67.02
3H	SCRI_RS_144534	67.02
3H	SCRI_RS_157904	67.02
3H	SCRI_RS_159431	67.02
3H	SCRI_RS_161066	67.02
3H	SCRI_RS_183659	67.02
3H	SCRI_RS_225522	67.02
3H	SCRI_RS_76971	67.3
3H	SCRI_RS_190529	67.46
3H	SCRI_RS_149160	67.94
3H	SCRI_RS_162931	68.13
3H	SCRI_RS_237939	68.13
3H	SCRI_RS_108543	68.42
3H	SCRI_RS_14456	68.42
3H	11_21305	68.78
3H	SCRI_RS_155609	68.93
3H	SCRI_RS_221981	68.93
3H	SCRI_RS_104564	69.03
3H	SCRI_RS_194602	69.03
3H	12_31275	69.47
3H	11_11394	69.57
3H	11_20931	69.57
3H	SCRI_RS_164675	69.7
3H	11_11276	69.71
3H	SCRI_RS_161186	69.71
3H	SCRI_RS_189039	69.83
3H	SCRI_RS_117006	70.28
3H	SCRI_RS_122796	70.28
3H	SCRI_RS_13778	70.28
3H	SCRI_RS_156056	70.28
3H	SCRI_RS_160216	70.28
3H	SCRI_RS_191130	70.28
3H	SCRI_RS_200011	70.28
3H	SCRI_RS_211003	70.28
3H	SCRI_RS_219322	70.28
3H	SCRI_RS_224377	70.28
3H	SCRI_RS_235869	70.28
3H	SCRI_RS_153222	70.35
3H	SCRI_RS_165390	70.35
3H	SCRI_RS_227565	70.35
3H	SCRI_RS_175886	70.49
3H	11_20017	70.91
3H	11_20351	70.91
3H	12_31242	70.91
3H	11_11314	71.45
3H	11_10813	71.87
3H	12_31323	71.87
3H	11_20877	72.09
3H	SCRI_RS_149945	72.47
3H	SCRI_RS_205216	72.47
3H	11_11241	72.51
3H	12_30754	72.51
3H	SCRI_RS_202772	72.53
3H	11_20694	73.27
3H	11_10350	73.69
3H	12_10452	73.69
3H	12_31356	73.69
3H	12_31529	73.69
3H	SCRI_RS_148020	73.83
3H	SCRI_RS_171062	73.83
3H	SCRI_RS_219894	73.83
3H	SCRI_RS_238157	73.83
3H	SCRI_RS_27417	73.83
3H	11_20521	74.32
3H	11_10276	74.86
3H	12_30399	74.86
3H	SCRI_RS_136721	75.26
3H	SCRI_RS_137852	75.26
3H	SCRI_RS_141432	75.26
3H	SCRI_RS_171415	75.26
3H	SCRI_RS_208373	75.26
3H	SCRI_RS_227898	75.26
3H	SCRI_RS_27538	75.26
3H	11_20566	75.44
3H	SCRI_RS_13617	75.45
3H	SCRI_RS_153148	75.79
3H	SCRI_RS_140190	76.08
3H	11_10683	76.09
3H	11_20695	76.09
3H	11_20778	76.09
3H	12_10609	76.09
3H	12_30743	76.09
3H	SCRI_RS_159249	76.31
3H	SCRI_RS_190676	76.31
3H	SCRI_RS_207408	76.31
3H	SCRI_RS_223611	76.31
3H	12_31346	76.79
3H	SCRI_RS_155763	77.5
3H	SCRI_RS_157648	77.5
3H	SCRI_RS_233246	77.5
3H	SCRI_RS_189790	78
3H	SCRI_RS_198659	78.35
3H	11_10047	78.39
3H	11_21163	78.39
3H	12_11454	78.39
3H	12_21194	78.39
3H	SCRI_RS_199922	78.4
3H	SCRI_RS_177399	78.64
3H	11_20362	78.81
3H	11_20597	79.48
3H	SCRI_RS_156111	79.5
3H	11_20222	80.08
3H	SCRI_RS_13780	80.3
3H	SCRI_RS_162539	80.3
3H	SCRI_RS_167410	80.3
3H	SCRI_RS_178618	80.3
3H	SCRI_RS_208662	80.3
3H	SCRI_RS_136937	80.4
3H	11_20115	81.02
3H	12_11524	81.02
3H	12_20849	81.02
3H	12_30170	81.02
3H	11_20093	82.1
3H	11_21358	82.1
3H	12_11517	82.1
3H	12_30677	82.1
3H	12_31262	82.1
3H	12_30278	83.43
3H	SCRI_RS_146347	84.08
3H	SCRI_RS_206471	84.08
3H	11_20063	85.43
3H	SCRI_RS_147950	85.92
3H	SCRI_RS_199887	85.92
3H	SCRI_RS_234564	85.92
3H	SCRI_RS_239399	85.92
3H	SCRI_RS_240090	85.92
3H	SCRI_RS_3193	85.92
3H	SCRI_RS_146429	86.08
3H	SCRI_RS_206202	86.08
3H	SCRI_RS_1509	86.08
3H	12_11322	86.08
3H	SCRI_RS_172730	86.08
3H	11_10444	86.34
3H	11_10628	86.34
3H	11_21505	86.34
3H	12_11138	86.34
3H	12_31299	86.34
3H	SCRI_RS_192761	86.78
3H	SCRI_RS_198154	86.78
3H	12_21019	86.82
3H	SCRI_RS_235849	86.88
3H	11_21348	86.95
3H	SCRI_RS_227472	86.98
3H	SCRI_RS_185339	86.98
3H	SCRI_RS_159340	87.38
3H	12_11468	88.55
3H	SCRI_RS_146480	88.55
3H	12_30663	88.58
3H	11_21294	88.58
3H	12_31018	88.58
3H	SCRI_RS_95272	88.58
3H	11_20136	88.58
3H	SCRI_RS_134017	88.58
3H	SCRI_RS_159006	88.58
3H	SCRI_RS_182014	88.58
3H	SCRI_RS_231362	88.58
3H	SCRI_RS_116760	88.74
3H	SCRI_RS_143933	88.74
3H	12_10134	89
3H	SCRI_RS_198609	89.12
3H	SCRI_RS_219733	89.12
3H	SCRI_RS_826	89.12
3H	12_30325	89.66
3H	11_20659	90.41
3H	11_10253	90.95
3H	12_10583	90.95
3H	SCRI_RS_137450	91.79
3H	SCRI_RS_159046	91.79
3H	SCRI_RS_165444	91.79
3H	11_10747	92.44
3H	11_11021	92.44
3H	SCRI_RS_158070	93.78
3H	SCRI_RS_198519	93.78
3H	SCRI_RS_80331	93.78
3H	SCRI_RS_161385	93.88
3H	12_11255	93.88
3H	SCRI_RS_150866	93.88
3H	SCRI_RS_130795	94.27
3H	12_31367	94.29
3H	12_10210	94.54
3H	12_20285	94.54
3H	SCRI_RS_172266	94.54
3H	SCRI_RS_8667	94.54
3H	12_30250	94.96
3H	12_30090	96.29
3H	11_20628	96.92
3H	11_20999	96.92
3H	11_21438	96.92
3H	SCRI_RS_166119	96.92
3H	12_30640	96.92
3H	SCRI_RS_138193	96.92
3H	11_20130	96.99
3H	12_10074	96.99
3H	12_30909	96.99
3H	SCRI_RS_140043	97.37
3H	11_10515	97.37
3H	11_20626	97.37
3H	SCRI_RS_174067	97.37
3H	SCRI_RS_231007	97.37
3H	11_21083	97.42
3H	SCRI_RS_120503	97.42
3H	SCRI_RS_162929	97.42
3H	SCRI_RS_229593	97.42
3H	SCRI_RS_3125	97.42
3H	11_21381	97.86
3H	12_10344	99.96
3H	11_21495	100.07
3H	SCRI_RS_115925	100.07
3H	SCRI_RS_228486	100.07
3H	SCRI_RS_235238	100.07
3H	12_30342	100.07
3H	12_10662	100.07
3H	SCRI_RS_170206	100.5
3H	SCRI_RS_238430	100.5
3H	SCRI_RS_3352	100.5
3H	11_21493	100.81
3H	SCRI_RS_236065	101.92
3H	SCRI_RS_116542	102
3H	SCRI_RS_168981	102
3H	SCRI_RS_237734	102
3H	SCRI_RS_142438	102.2
3H	SCRI_RS_147984	102.2
3H	SCRI_RS_230069	102.2
3H	SCRI_RS_153519	102.3
3H	SCRI_RS_13434	102.3
3H	SCRI_RS_155	102.44
3H	SCRI_RS_167825	103.83
3H	SCRI_RS_234342	103.85
3H	SCRI_RS_156032	103.99
3H	SCRI_RS_174963	104.02
3H	11_21513	104.75
3H	SCRI_RS_166189	105.9
3H	12_11392	105.95
3H	12_21081	105.95
3H	11_21212	106.38
3H	12_30423	106.38
3H	SCRI_RS_187928	106.38
3H	SCRI_RS_211929	106.38
3H	SCRI_RS_221787	106.38
3H	SCRI_RS_235791	106.38
3H	SCRI_RS_133339	106.55
3H	SCRI_RS_164704	106.55
3H	SCRI_RS_225641	106.55
3H	SCRI_RS_7396	106.55
3H	SCRI_RS_230075	106.84
3H	SCRI_RS_142964	107.31
3H	SCRI_RS_154011	107.89
3H	SCRI_RS_159125	107.89
3H	SCRI_RS_198594	107.89
3H	11_20023	107.92
3H	11_21161	107.92
3H	12_30926	108.03
3H	SCRI_RS_192360	108.03
3H	SCRI_RS_14857	108.5
3H	11_10753	108.59
3H	SCRI_RS_114333	108.59
3H	SCRI_RS_138983	108.59
3H	SCRI_RS_149566	108.59
3H	11_20168	109.26
3H	11_21277	109.26
3H	SCRI_RS_150944	109.66
3H	SCRI_RS_200365	109.66
3H	SCRI_RS_131897	109.89
3H	SCRI_RS_163092	109.89
3H	11_10312	110.33
3H	11_11503	110.33
3H	SCRI_RS_206510	110.33
3H	SCRI_RS_5194	110.33
3H	12_30375	110.33
3H	12_31329	110.33
3H	SCRI_RS_136593	110.33
3H	SCRI_RS_141195	110.33
3H	SCRI_RS_143059	110.33
3H	SCRI_RS_148653	110.33
3H	SCRI_RS_154397	110.33
3H	SCRI_RS_171144	110.33
3H	SCRI_RS_184462	110.33
3H	SCRI_RS_196677	110.33
3H	SCRI_RS_231027	110.33
3H	SCRI_RS_236528	110.33
3H	12_30276	110.33
3H	11_20523	110.33
3H	12_10100	110.33
3H	SCRI_RS_151711	111.55
3H	SCRI_RS_153915	111.55
3H	SCRI_RS_164290	111.55
3H	SCRI_RS_193132	111.55
3H	SCRI_RS_150063	111.64
3H	SCRI_RS_153414	111.64
3H	SCRI_RS_183638	111.64
3H	SCRI_RS_203905	111.64
3H	SCRI_RS_170370	111.64
3H	SCRI_RS_202425	111.64
3H	11_10584	111.73
3H	12_30927	111.73
3H	SCRI_RS_138723	112.24
3H	SCRI_RS_120973	112.44
3H	SCRI_RS_181071	112.44
3H	12_10680	112.48
3H	SCRI_RS_121052	112.74
3H	SCRI_RS_103215	113.04
3H	SCRI_RS_165334	113.04
3H	SCRI_RS_138317	113.04
3H	12_31420	113.04
3H	11_11330	114.3
3H	12_30223	114.3
3H	12_31220	114.3
3H	12_31269	115.05
3H	11_20944	115.71
3H	12_30732	115.71
3H	12_31499	115.71
3H	11_10918	117.09
3H	11_21405	117.09
3H	12_30274	117.83
3H	12_31525	118.07
3H	11_10044	118.73
3H	11_10754	118.73
3H	11_10821	118.73
3H	11_11172	118.73
3H	12_11338	118.73
3H	12_30924	118.73
3H	11_10867	119.45
3H	11_20650	119.45
3H	12_20413	119.45
3H	12_30096	119.45
3H	12_31238	119.45
3H	12_30972	120.55
3H	SCRI_RS_146012	120.55
3H	11_10280	120.94
3H	11_20343	120.94
3H	11_20662	120.94
3H	12_30081	120.94
3H	SCRI_RS_146197	121.19
3H	SCRI_RS_237846	121.19
3H	11_11141	121.76
3H	12_10188	121.76
3H	12_10505	121.76
3H	12_30963	121.76
3H	SCRI_RS_180027	122.25
3H	SCRI_RS_173004	122.31
3H	SCRI_RS_202406	122.31
3H	11_11196	122.41
3H	12_20369	122.41
3H	SCRI_RS_167755	122.85
3H	11_10842	123.02
3H	11_20612	123.02
3H	12_10205	124.58
3H	11_20527	124.62
3H	12_30973	124.62
3H	12_30092	125.38
3H	12_10122	125.55
3H	SCRI_RS_13871	125.55
3H	SCRI_RS_189161	125.55
3H	SCRI_RS_200201	125.55
3H	11_10381	126.69
3H	11_20409	126.69
3H	11_21428	126.69
3H	11_20146	126.69
3H	SCRI_RS_154027	126.69
3H	SCRI_RS_219896	127.25
3H	SCRI_RS_202428	127.29
3H	SCRI_RS_208297	127.89
3H	11_20085	127.89
3H	SCRI_RS_151733	127.89
3H	11_11127	127.89
3H	SCRI_RS_209963	129.2
3H	11_11328	129.46
3H	12_11154	129.46
3H	12_31251	129.62
3H	SCRI_RS_162863	130.08
3H	11_20920	130.49
3H	SCRI_RS_208688	130.72
3H	SCRI_RS_69837	131.15
3H	11_20851	131.33
3H	SCRI_RS_10016	131.33
3H	SCRI_RS_213950	131.33
3H	SCRI_RS_175038	131.34
3H	SCRI_RS_24545	131.34
3H	12_30367	131.34
3H	12_11047	131.36
3H	SCRI_RS_177313	131.36
3H	12_31496	131.36
3H	SCRI_RS_194148	132.17
3H	12_30137	132.5
3H	11_10570	132.77
3H	SCRI_RS_106728	132.77
3H	SCRI_RS_113112	132.77
3H	SCRI_RS_14107	132.77
3H	SCRI_RS_168977	132.77
3H	SCRI_RS_204462	132.77
3H	SCRI_RS_208633	132.77
3H	SCRI_RS_221869	132.77
3H	SCRI_RS_231382	132.97
3H	12_10542	135.55
3H	12_11208	135.64
3H	12_21386	135.64
3H	SCRI_RS_209285	135.64
3H	SCRI_RS_183550	135.88
3H	SCRI_RS_169325	135.98
3H	SCRI_RS_175220	135.98
3H	12_31473	136.12
3H	SCRI_RS_167698	136.28
3H	SCRI_RS_144559	136.37
3H	SCRI_RS_175682	136.37
3H	SCRI_RS_168360	136.37
3H	SCRI_RS_205592	136.68
3H	SCRI_RS_115755	136.72
3H	SCRI_RS_1793	137.02
3H	11_20952	137.21
3H	11_21272	139.02
3H	SCRI_RS_184593	139.02
3H	SCRI_RS_135155	139.22
3H	SCRI_RS_135156	139.22
3H	SCRI_RS_144313	139.22
3H	SCRI_RS_182610	139.22
3H	SCRI_RS_187742	139.22
3H	SCRI_RS_189186	139.22
3H	SCRI_RS_165044	139.22
3H	12_31161	139.32
3H	SCRI_RS_129737	139.32
3H	SCRI_RS_189710	139.32
3H	SCRI_RS_90299	139.32
3H	SCRI_RS_204422	139.39
3H	12_21376	141.02
3H	SCRI_RS_205957	141.22
3H	SCRI_RS_113294	141.28
3H	SCRI_RS_172357	141.32
3H	SCRI_RS_179447	141.32
3H	SCRI_RS_208722	141.32
3H	SCRI_RS_238255	141.32
3H	12_31500	141.62
3H	SCRI_RS_205663	141.92
3H	12_20421	142.96
3H	12_21345	143.28
3H	12_30370	143.28
3H	12_20505	143.28
3H	11_20155	143.41
3H	SCRI_RS_151868	143.41
3H	SCRI_RS_154449	143.41
3H	SCRI_RS_158679	143.41
3H	SCRI_RS_157113	143.46
3H	11_11436	143.56
3H	SCRI_RS_201075	143.56
3H	12_30921	143.56
3H	12_10564	143.56
3H	SCRI_RS_110995	143.56
3H	SCRI_RS_164726	146.06
3H	SCRI_RS_224468	146.06
3H	11_10646	146.46
3H	12_20198	146.46
3H	11_10935	146.46
3H	12_30767	146.46
3H	SCRI_RS_161416	146.46
3H	SCRI_RS_206483	146.46
3H	SCRI_RS_216174	146.77
3H	11_21523	147.06
3H	SCRI_RS_118833	147.06
3H	12_30960	147.06
3H	SCRI_RS_130177	147.06
3H	SCRI_RS_141898	147.06
3H	SCRI_RS_230023	147.06
3H	SCRI_RS_126351	147.06
3H	SCRI_RS_168042	147.18
3H	SCRI_RS_173623	147.42
3H	SCRI_RS_65010	147.42
3H	11_21008	147.44
3H	12_30271	147.6
3H	SCRI_RS_188420	147.76
3H	SCRI_RS_126369	147.86
3H	SCRI_RS_232945	147.96
3H	SCRI_RS_229623	147.96
3H	SCRI_RS_144189	147.96
3H	SCRI_RS_237864	148.1
3H	11_10702	148.1
3H	SCRI_RS_176108	148.1
3H	SCRI_RS_238412	148.1
3H	SCRI_RS_232649	148.1
3H	SCRI_RS_194256	148.76
3H	11_20605	148.76
3H	SCRI_RS_189322	148.76
3H	SCRI_RS_216141	148.76
3H	SCRI_RS_180847	149.16
3H	12_10629	149.32
3H	12_31388	149.32
3H	11_21267	149.93
3H	12_30736	149.93
3H	SCRI_RS_128254	149.93
3H	SCRI_RS_128336	149.93
3H	SCRI_RS_142818	149.93
3H	SCRI_RS_143594	149.93
3H	SCRI_RS_154549	149.93
3H	SCRI_RS_156315	149.93
3H	SCRI_RS_159344	149.93
3H	SCRI_RS_194527	149.93
3H	SCRI_RS_204353	149.93
3H	SCRI_RS_206475	149.93
3H	SCRI_RS_216865	149.93
3H	SCRI_RS_230717	149.93
3H	SCRI_RS_237894	149.93
3H	SCRI_RS_739	149.93
3H	11_10694	149.93
3H	11_11410	149.93
3H	SCRI_RS_127719	149.93
3H	11_10681	149.93
3H	11_10893	149.93
3H	11_11411	149.93
3H	SCRI_RS_1435	149.93
3H	SCRI_RS_49693	149.93
3H	12_10014	149.93
3H	11_11516	149.93
3H	SCRI_RS_192891	149.93
3H	SCRI_RS_203164	149.93
3H	SCRI_RS_1172	149.93
3H	11_20339	149.93
3H	11_20057	149.93
3H	SCRI_RS_146798	150.59
3H	11_11320	150.59
3H	SCRI_RS_128375	150.59
3H	12_20176	151.18
3H	SCRI_RS_139876	151.6
3H	SCRI_RS_184998	151.6
3H	12_30229	152.49
3H	SCRI_RS_112067	152.62
3H	11_10423	152.81
3H	12_30055	154.48
3H	11_10767	154.64
3H	11_10343	155.65
3H	12_10215	155.74
3H	12_30135	155.74
3H	12_10981	155.74
3H	SCRI_RS_220221	155.74
3H	SCRI_RS_143505	155.82
3H	SCRI_RS_160338	155.82
3H	SCRI_RS_178836	155.82
3H	SCRI_RS_184261	155.82
3H	SCRI_RS_236603	155.82
3H	SCRI_RS_237738	155.82
3H	11_21362	155.92
3H	SCRI_RS_13426	156.48
3H	SCRI_RS_153022	156.48
3H	SCRI_RS_159189	156.48
3H	SCRI_RS_162720	156.48
3H	SCRI_RS_238050	156.48
4H	marker.2	consensus.2
4H	12_21117	0
4H	12_30764	0
4H	SCRI_RS_150051	0.1
4H	SCRI_RS_164381	0.1
4H	SCRI_RS_185058	0.1
4H	SCRI_RS_32890	0.2
4H	SCRI_RS_170785	0.4
4H	12_31324	0.7
4H	SCRI_RS_126417	0.7
4H	SCRI_RS_149873	0.7
4H	SCRI_RS_176074	0.7
4H	SCRI_RS_116187	0.7
4H	SCRI_RS_153221	0.7
4H	SCRI_RS_169770	0.7
4H	SCRI_RS_173223	0.7
4H	SCRI_RS_236542	0.7
4H	SCRI_RS_236981	0.7
4H	SCRI_RS_13428	0.9
4H	12_20954	0.9
4H	12_31167	0.9
4H	SCRI_RS_113609	0.9
4H	SCRI_RS_206744	0.9
4H	SCRI_RS_220188	0.9
4H	SCRI_RS_222283	0.9
4H	SCRI_RS_80297	0.94
4H	11_20145	1.15
4H	SCRI_RS_110334	1.15
4H	SCRI_RS_235822	1.15
4H	11_10208	2.55
4H	11_21056	2.55
4H	11_10409	3.15
4H	12_11020	3.35
4H	11_21228	3.75
4H	12_30140	3.75
4H	SCRI_RS_100278	4.14
4H	12_20274	5.07
4H	12_31486	5.07
4H	12_11485	6.14
4H	11_10319	6.41
4H	SCRI_RS_231066	6.66
4H	12_31511	6.66
4H	SCRI_RS_154517	6.66
4H	SCRI_RS_173731	6.66
4H	SCRI_RS_235733	6.66
4H	11_10490	7.71
4H	12_11300	7.71
4H	12_31458	7.71
4H	12_30540	10.25
4H	12_30150	12.33
4H	SCRI_RS_10014	14.31
4H	SCRI_RS_145228	14.31
4H	SCRI_RS_150573	14.31
4H	SCRI_RS_152153	14.31
4H	SCRI_RS_154075	14.31
4H	SCRI_RS_176647	14.31
4H	SCRI_RS_194090	14.31
4H	SCRI_RS_197394	14.31
4H	SCRI_RS_219136	14.31
4H	SCRI_RS_220110	14.31
4H	11_10738	14.82
4H	12_31086	14.82
4H	SCRI_RS_146989	14.82
4H	SCRI_RS_190426	14.82
4H	SCRI_RS_195137	14.82
4H	11_10113	14.82
4H	11_10223	14.82
4H	11_21359	15.3
4H	11_20557	15.73
4H	SCRI_RS_175862	16.61
4H	SCRI_RS_189331	16.61
4H	SCRI_RS_203148	16.61
4H	12_31291	17.13
4H	11_10221	17.79
4H	SCRI_RS_222099	18.36
4H	11_11199	18.82
4H	SCRI_RS_105960	18.97
4H	SCRI_RS_180891	18.97
4H	SCRI_RS_134182	19.5
4H	11_11136	19.57
4H	12_21458	20.31
4H	12_11060	21.06
4H	11_21385	21.06
4H	12_10562	21.06
4H	12_10626	21.06
4H	12_30394	21.06
4H	11_20210	21.57
4H	12_11175	21.57
4H	12_30793	22.12
4H	11_20302	22.86
4H	11_20422	22.86
4H	11_20606	22.86
4H	11_21070	22.86
4H	11_20680	23.61
4H	11_21418	23.61
4H	11_20109	24.2
4H	11_20777	24.62
4H	SCRI_RS_119628	24.7
4H	SCRI_RS_150585	24.7
4H	SCRI_RS_154178	24.7
4H	11_20411	25.23
4H	11_21374	25.23
4H	12_30907	25.23
4H	11_20001	25.23
4H	SCRI_RS_121241	25.8
4H	SCRI_RS_7704	26.23
4H	SCRI_RS_162784	26.44
4H	12_31313	26.46
4H	SCRI_RS_188190	26.83
4H	12_31164	26.89
4H	11_21389	27.92
4H	11_21122	28.78
4H	11_21397	28.78
4H	12_30863	28.78
4H	12_30864	28.78
4H	12_30865	28.78
4H	SCRI_RS_139079	29.16
4H	SCRI_RS_125487	29.4
4H	SCRI_RS_12719	29.4
4H	SCRI_RS_127657	29.4
4H	SCRI_RS_162743	29.4
4H	SCRI_RS_58772	29.4
4H	SCRI_RS_98443	29.4
4H	SCRI_RS_228232	29.4
4H	SCRI_RS_157832	29.6
4H	SCRI_RS_143144	31.24
4H	SCRI_RS_207768	31.24
4H	SCRI_RS_225074	31.24
4H	SCRI_RS_238822	31.24
4H	SCRI_RS_39834	31.24
4H	12_10810	31.42
4H	12_10860	31.42
4H	SCRI_RS_209362	35.5
4H	12_11382	35.76
4H	12_30992	36.6
4H	11_20012	37.26
4H	12_10171	37.26
4H	12_10347	37.26
4H	12_10371	37.26
4H	SCRI_RS_179489	37.4
4H	11_20114	37.92
4H	11_20180	37.92
4H	12_10063	37.92
4H	12_20011	37.92
4H	12_20240	37.92
4H	12_30328	38.58
4H	12_21003	39.49
4H	SCRI_RS_161528	39.6
4H	SCRI_RS_6956	39.6
4H	SCRI_RS_116338	39.6
4H	SCRI_RS_183399	39.6
4H	SCRI_RS_75805	39.6
4H	SCRI_RS_145412	39.6
4H	11_10048	39.9
4H	12_30187	39.9
4H	SCRI_RS_220122	41.53
4H	11_11180	41.7
4H	SCRI_RS_190764	41.87
4H	12_30993	42.36
4H	11_10668	43.15
4H	11_10793	43.15
4H	SCRI_RS_9296	43.7
4H	11_20939	44.46
4H	12_11112	44.46
4H	SCRI_RS_154081	45.49
4H	SCRI_RS_167844	45.74
4H	11_11405	46.21
4H	12_30564	46.21
4H	SCRI_RS_159159	46.77
4H	11_10261	46.87
4H	12_30331	46.87
4H	12_30777	46.87
4H	12_31382	46.87
4H	11_10432	47.66
4H	11_10577	47.66
4H	11_10667	47.66
4H	11_10756	47.66
4H	11_20269	47.66
4H	11_21073	47.66
4H	11_20782	47.87
4H	11_21071	47.87
4H	11_10942	48.08
4H	11_20853	48.08
4H	SCRI_RS_144321	48.34
4H	SCRI_RS_144322	48.34
4H	11_10028	48.72
4H	11_10093	48.72
4H	11_10411	48.72
4H	11_10424	48.72
4H	11_10509	48.72
4H	11_11042	48.72
4H	11_11332	48.72
4H	11_20496	48.72
4H	11_21254	48.72
4H	11_21303	48.72
4H	12_10492	48.72
4H	12_11063	48.72
4H	12_11190	48.72
4H	12_20156	48.72
4H	12_20525	48.72
4H	12_20831	48.72
4H	12_21029	48.72
4H	12_21442	48.72
4H	12_30488	48.72
4H	12_30525	48.72
4H	12_30605	48.72
4H	12_30684	48.72
4H	12_30878	48.72
4H	12_31026	48.72
4H	12_31156	48.72
4H	12_31360	48.72
4H	SCRI_RS_222133	49.2
4H	12_30450	49.38
4H	12_30866	49.38
4H	12_21137	49.38
4H	SCRI_RS_176091	49.41
4H	SCRI_RS_207030	49.57
4H	SCRI_RS_209409	49.57
4H	11_10379	50.04
4H	11_10480	50.04
4H	11_20289	50.04
4H	12_10607	50.04
4H	SCRI_RS_157290	50.09
4H	SCRI_RS_157666	50.09
4H	SCRI_RS_169389	50.09
4H	SCRI_RS_188580	50.09
4H	SCRI_RS_217322	50.09
4H	SCRI_RS_176092	50.13
4H	SCRI_RS_160373	50.14
4H	SCRI_RS_226221	50.14
4H	SCRI_RS_156596	50.17
4H	SCRI_RS_188187	50.17
4H	SCRI_RS_190401	50.17
4H	SCRI_RS_135637	50.24
4H	SCRI_RS_1396	50.24
4H	SCRI_RS_153459	50.24
4H	SCRI_RS_154048	50.24
4H	SCRI_RS_157310	50.24
4H	SCRI_RS_168580	50.24
4H	SCRI_RS_184107	50.24
4H	SCRI_RS_189400	50.24
4H	SCRI_RS_194649	50.24
4H	SCRI_RS_198888	50.24
4H	SCRI_RS_220535	50.24
4H	SCRI_RS_220697	50.24
4H	SCRI_RS_231739	50.24
4H	SCRI_RS_2937	50.24
4H	SCRI_RS_151735	50.27
4H	SCRI_RS_101389	50.29
4H	SCRI_RS_119778	50.29
4H	SCRI_RS_128974	50.29
4H	SCRI_RS_145311	50.29
4H	SCRI_RS_148289	50.29
4H	SCRI_RS_150603	50.29
4H	SCRI_RS_157072	50.29
4H	SCRI_RS_157396	50.29
4H	SCRI_RS_171142	50.29
4H	SCRI_RS_175365	50.29
4H	SCRI_RS_177312	50.29
4H	SCRI_RS_194316	50.29
4H	SCRI_RS_200010	50.29
4H	SCRI_RS_205863	50.29
4H	SCRI_RS_213798	50.29
4H	SCRI_RS_219128	50.29
4H	SCRI_RS_224697	50.29
4H	SCRI_RS_229704	50.29
4H	SCRI_RS_239818	50.29
4H	SCRI_RS_92128	50.29
4H	SCRI_RS_16811	50.32
4H	SCRI_RS_176630	50.47
4H	11_10881	50.58
4H	11_20610	50.58
4H	SCRI_RS_133147	50.58
4H	SCRI_RS_170494	50.58
4H	SCRI_RS_181886	50.58
4H	11_10946	50.58
4H	11_11114	50.58
4H	12_30427	50.58
4H	12_11232	50.67
4H	12_10836	50.68
4H	SCRI_RS_110052	50.68
4H	SCRI_RS_130686	50.68
4H	SCRI_RS_145379	50.68
4H	SCRI_RS_164612	50.68
4H	SCRI_RS_187708	50.68
4H	SCRI_RS_188484	50.68
4H	SCRI_RS_188527	50.68
4H	SCRI_RS_203147	50.68
4H	SCRI_RS_206918	50.68
4H	SCRI_RS_233908	50.68
4H	12_11077	50.82
4H	12_11269	50.82
4H	SCRI_RS_130433	50.82
4H	SCRI_RS_143088	50.82
4H	SCRI_RS_143091	50.82
4H	SCRI_RS_188822	50.82
4H	SCRI_RS_6833	50.82
4H	SCRI_RS_128984	50.9
4H	11_20135	50.96
4H	11_20450	50.96
4H	11_20472	50.96
4H	11_21017	50.96
4H	12_10426	50.96
4H	12_10637	50.96
4H	12_30839	50.96
4H	11_10527	50.96
4H	11_20363	50.96
4H	12_10088	50.96
4H	12_30060	50.96
4H	12_31297	50.96
4H	SCRI_RS_168496	50.96
4H	SCRI_RS_185430	50.96
4H	SCRI_RS_206232	50.96
4H	SCRI_RS_228477	50.96
4H	11_10568	50.96
4H	SCRI_RS_221172	50.96
4H	12_30995	50.96
4H	12_31462	50.96
4H	11_10262	50.96
4H	11_20412	50.96
4H	SCRI_RS_147636	50.96
4H	SCRI_RS_208732	50.96
4H	11_10046	50.96
4H	11_20020	50.96
4H	SCRI_RS_128723	50.96
4H	SCRI_RS_141730	50.96
4H	SCRI_RS_155554	50.96
4H	SCRI_RS_208828	50.96
4H	SCRI_RS_10216	50.96
4H	SCRI_RS_109271	50.96
4H	SCRI_RS_121003	50.96
4H	SCRI_RS_141848	50.96
4H	SCRI_RS_187354	50.96
4H	SCRI_RS_195892	50.96
4H	SCRI_RS_197371	50.96
4H	SCRI_RS_204804	50.96
4H	SCRI_RS_237142	50.96
4H	SCRI_RS_239237	50.96
4H	SCRI_RS_239781	50.96
4H	11_10914	50.96
4H	12_11315	50.96
4H	12_20144	50.96
4H	12_30503	50.96
4H	12_30597	50.96
4H	12_30625	50.96
4H	12_31310	50.96
4H	12_31414	50.96
4H	SCRI_RS_104697	50.96
4H	SCRI_RS_123814	50.96
4H	SCRI_RS_133407	50.96
4H	SCRI_RS_134953	50.96
4H	SCRI_RS_137360	50.96
4H	SCRI_RS_140586	50.96
4H	SCRI_RS_143191	50.96
4H	SCRI_RS_144983	50.96
4H	SCRI_RS_165861	50.96
4H	SCRI_RS_175331	50.96
4H	SCRI_RS_186944	50.96
4H	SCRI_RS_189638	50.96
4H	SCRI_RS_192458	50.96
4H	SCRI_RS_193122	50.96
4H	SCRI_RS_193415	50.96
4H	SCRI_RS_199711	50.96
4H	SCRI_RS_207936	50.96
4H	SCRI_RS_230472	50.96
4H	SCRI_RS_62855	50.96
4H	SCRI_RS_7401	50.96
4H	SCRI_RS_74014	50.96
4H	SCRI_RS_81718	50.96
4H	SCRI_RS_9618	50.96
4H	11_21481	50.96
4H	12_20898	50.96
4H	11_10807	50.96
4H	12_11108	50.96
4H	SCRI_RS_13552	50.97
4H	SCRI_RS_148130	50.97
4H	SCRI_RS_150615	50.97
4H	SCRI_RS_164698	50.97
4H	SCRI_RS_182555	50.97
4H	SCRI_RS_173516	51.12
4H	SCRI_RS_225722	51.3
4H	SCRI_RS_204773	51.61
4H	SCRI_RS_10607	51.9
4H	SCRI_RS_237345	51.9
4H	SCRI_RS_151142	52.04
4H	SCRI_RS_94957	52.04
4H	12_20926	52.25
4H	SCRI_RS_168603	52.84
4H	SCRI_RS_182937	52.84
4H	SCRI_RS_195935	52.84
4H	SCRI_RS_224629	52.84
4H	11_20361	53.23
4H	11_21010	53.23
4H	SCRI_RS_206025	53.23
4H	SCRI_RS_234596	53.23
4H	SCRI_RS_66562	53.23
4H	SCRI_RS_188340	53.23
4H	11_21400	53.88
4H	12_10500	54.18
4H	SCRI_RS_14612	54.2
4H	SCRI_RS_123275	54.2
4H	11_20482	54.28
4H	SCRI_RS_136645	54.35
4H	12_11427	55
4H	11_21191	55
4H	SCRI_RS_170560	55.08
4H	SCRI_RS_204282	55.08
4H	SCRI_RS_98343	55.08
4H	SCRI_RS_136648	55.14
4H	11_20453	55.34
4H	11_21087	55.34
4H	11_21273	55.34
4H	11_21296	55.34
4H	SCRI_RS_109600	55.38
4H	SCRI_RS_189180	55.38
4H	SCRI_RS_202326	55.38
4H	SCRI_RS_207394	55.38
4H	SCRI_RS_235738	55.38
4H	SCRI_RS_167567	55.8
4H	11_11207	55.99
4H	12_31186	55.99
4H	11_20820	55.99
4H	SCRI_RS_113574	56.16
4H	SCRI_RS_135365	56.38
4H	SCRI_RS_138684	56.38
4H	SCRI_RS_163440	56.38
4H	SCRI_RS_166159	56.38
4H	SCRI_RS_172285	56.38
4H	SCRI_RS_188555	56.38
4H	SCRI_RS_192900	56.38
4H	SCRI_RS_196044	56.38
4H	SCRI_RS_234574	56.38
4H	SCRI_RS_96016	56.38
4H	11_20062	56.58
4H	11_20723	56.58
4H	SCRI_RS_134620	56.79
4H	SCRI_RS_14498	56.79
4H	SCRI_RS_146174	56.79
4H	SCRI_RS_146941	56.79
4H	SCRI_RS_151213	56.79
4H	SCRI_RS_153392	56.79
4H	SCRI_RS_166817	56.79
4H	SCRI_RS_175805	56.79
4H	SCRI_RS_206179	56.79
4H	SCRI_RS_20701	56.79
4H	SCRI_RS_218193	56.79
4H	SCRI_RS_8100	56.79
4H	SCRI_RS_149252	56.86
4H	SCRI_RS_195216	56.86
4H	SCRI_RS_206537	56.86
4H	SCRI_RS_110333	56.88
4H	SCRI_RS_147712	56.88
4H	SCRI_RS_163033	56.88
4H	SCRI_RS_165031	56.88
4H	SCRI_RS_165912	56.88
4H	SCRI_RS_168478	56.88
4H	SCRI_RS_225248	56.88
4H	SCRI_RS_229658	56.88
4H	SCRI_RS_239145	56.88
4H	SCRI_RS_157650	57.51
4H	SCRI_RS_151068	57.73
4H	SCRI_RS_144194	57.76
4H	SCRI_RS_185497	57.76
4H	SCRI_RS_189363	57.76
4H	SCRI_RS_206361	57.76
4H	SCRI_RS_123385	57.84
4H	SCRI_RS_13921	58.15
4H	11_20924	58.4
4H	SCRI_RS_166457	58.69
4H	11_10639	59.05
4H	SCRI_RS_137903	59.24
4H	SCRI_RS_139806	59.24
4H	SCRI_RS_140349	59.24
4H	SCRI_RS_147772	59.24
4H	SCRI_RS_215737	59.24
4H	11_10846	59.27
4H	11_11431	59.27
4H	12_10053	59.27
4H	12_30620	59.27
4H	12_31515	59.27
4H	SCRI_RS_13644	59.49
4H	SCRI_RS_189861	59.82
4H	SCRI_RS_194525	59.82
4H	SCRI_RS_212903	59.82
4H	11_11070	59.96
4H	SCRI_RS_186106	59.96
4H	SCRI_RS_206928	59.96
4H	SCRI_RS_213542	59.96
4H	12_30455	59.96
4H	SCRI_RS_197889	59.97
4H	SCRI_RS_10205	60.1
4H	SCRI_RS_132067	60.1
4H	11_10010	60.62
4H	12_30755	60.62
4H	12_30904	60.62
4H	12_30905	60.62
4H	12_30906	60.62
4H	12_31385	60.62
4H	SCRI_RS_219816	60.82
4H	11_20906	60.83
4H	11_10606	61.04
4H	11_20072	61.04
4H	SCRI_RS_143825	61.27
4H	SCRI_RS_154704	61.27
4H	11_11224	61.46
4H	SCRI_RS_179701	61.73
4H	SCRI_RS_141230	61.8
4H	11_10627	62.43
4H	11_20451	62.43
4H	11_20580	62.43
4H	12_30693	62.43
4H	12_31536	62.43
4H	SCRI_RS_108495	62.87
4H	SCRI_RS_108509	62.87
4H	SCRI_RS_108502	62.87
4H	SCRI_RS_142257	63.57
4H	11_11513	63.69
4H	11_21504	63.69
4H	SCRI_RS_197405	63.72
4H	SCRI_RS_8092	64
4H	SCRI_RS_89959	64
4H	SCRI_RS_119486	64.5
4H	11_20740	64.79
4H	SCRI_RS_148392	64.79
4H	SCRI_RS_231208	64.79
4H	SCRI_RS_4583	64.79
4H	11_10467	64.79
4H	11_10829	65.73
4H	12_30994	65.73
4H	12_31258	65.73
4H	12_31362	65.73
4H	SCRI_RS_175327	66.46
4H	11_10309	67.05
4H	11_20815	67.05
4H	11_10090	67.27
4H	SCRI_RS_154658	67.27
4H	SCRI_RS_179444	67.27
4H	SCRI_RS_159331	67.33
4H	SCRI_RS_195017	67.33
4H	SCRI_RS_218491	67.33
4H	SCRI_RS_239956	67.33
4H	SCRI_RS_106799	67.4
4H	11_21353	67.41
4H	12_30136	67.41
4H	12_31231	67.41
4H	11_11319	67.41
4H	12_20143	67.41
4H	11_11004	67.56
4H	SCRI_RS_160624	67.75
4H	11_10247	68.41
4H	11_21332	68.41
4H	SCRI_RS_155536	68.41
4H	SCRI_RS_179438	68.41
4H	SCRI_RS_184798	68.53
4H	SCRI_RS_106435	68.7
4H	SCRI_RS_237913	68.71
4H	SCRI_RS_144091	69.06
4H	SCRI_RS_166212	69.06
4H	12_31148	69.7
4H	12_10170	70.1
4H	12_30787	70.1
4H	11_11500	70.92
4H	SCRI_RS_200957	70.92
4H	SCRI_RS_200964	70.92
4H	12_30226	70.93
4H	12_11526	71.5
4H	11_20670	71.51
4H	11_20197	72.17
4H	11_20718	72.17
4H	11_10724	72.82
4H	SCRI_RS_221945	73.31
4H	SCRI_RS_125524	73.4
4H	SCRI_RS_13460	73.4
4H	SCRI_RS_144204	73.4
4H	SCRI_RS_152388	73.4
4H	SCRI_RS_163062	73.4
4H	SCRI_RS_168074	73.4
4H	SCRI_RS_181725	73.4
4H	SCRI_RS_223734	73.4
4H	SCRI_RS_238618	73.4
4H	SCRI_RS_7523	73.4
4H	12_31246	73.81
4H	11_10723	74.18
4H	12_30390	74.18
4H	11_20689	74.26
4H	12_10670	74.67
4H	11_11213	74.78
4H	11_21151	74.78
4H	SCRI_RS_134956	75.15
4H	11_20178	75.38
4H	12_10022	75.38
4H	11_11398	75.68
4H	11_20765	75.88
4H	SCRI_RS_203859	75.88
4H	SCRI_RS_117233	75.92
4H	SCRI_RS_164399	75.92
4H	11_20358	75.99
4H	11_10588	76.88
4H	SCRI_RS_210971	76.88
4H	11_10751	76.88
4H	SCRI_RS_171874	78.2
4H	SCRI_RS_132562	78.41
4H	SCRI_RS_165623	78.41
4H	SCRI_RS_166474	78.41
4H	SCRI_RS_233242	78.41
4H	SCRI_RS_237756	78.41
4H	12_30138	78.72
4H	11_20384	78.72
4H	11_20732	79.59
4H	SCRI_RS_107010	80.14
4H	SCRI_RS_129218	80.14
4H	SCRI_RS_168399	80.14
4H	SCRI_RS_172072	80.14
4H	SCRI_RS_184126	80.4
4H	12_30232	80.58
4H	12_30655	80.68
4H	SCRI_RS_176669	80.75
4H	11_20513	81.3
4H	12_20648	81.3
4H	12_31523	81.3
4H	SCRI_RS_128147	81.3
4H	SCRI_RS_149449	81.3
4H	SCRI_RS_152166	81.3
4H	SCRI_RS_165833	81.3
4H	SCRI_RS_169134	81.3
4H	SCRI_RS_170178	81.3
4H	SCRI_RS_207108	81.3
4H	SCRI_RS_230980	81.3
4H	11_10785	81.36
4H	12_30718	81.36
4H	12_10271	81.77
4H	12_10824	82.16
4H	12_30554	82.16
4H	SCRI_RS_11114	82.16
4H	SCRI_RS_139508	82.16
4H	SCRI_RS_181163	82.16
4H	SCRI_RS_182626	82.16
4H	SCRI_RS_189881	82.16
4H	SCRI_RS_205975	82.16
4H	SCRI_RS_226787	82.55
4H	12_20825	82.84
4H	SCRI_RS_141214	82.84
4H	SCRI_RS_214059	82.84
4H	11_20838	82.92
4H	12_30114	82.92
4H	SCRI_RS_138835	83.31
4H	11_21243	83.46
4H	12_30117	83.63
4H	12_30584	84.01
4H	11_20762	84.36
4H	SCRI_RS_14487	84.36
4H	SCRI_RS_168581	84.36
4H	SCRI_RS_168591	84.36
4H	SCRI_RS_188827	84.36
4H	SCRI_RS_107762	84.46
4H	SCRI_RS_162410	84.8
4H	12_30158	84.94
4H	SCRI_RS_203870	84.94
4H	11_11292	84.94
4H	11_20119	84.94
4H	12_10666	84.94
4H	SCRI_RS_229409	85.22
4H	SCRI_RS_233444	85.51
4H	SCRI_RS_141803	85.66
4H	SCRI_RS_148330	85.66
4H	SCRI_RS_153700	85.66
4H	SCRI_RS_157611	85.66
4H	SCRI_RS_16715	85.66
4H	SCRI_RS_167808	85.66
4H	SCRI_RS_179398	85.66
4H	SCRI_RS_192689	85.66
4H	SCRI_RS_6448	85.66
4H	SCRI_RS_201871	85.8
4H	SCRI_RS_7914	85.8
4H	11_20454	85.81
4H	SCRI_RS_131671	85.81
4H	12_11139	86.37
4H	12_30990	86.37
4H	SCRI_RS_148773	86.37
4H	SCRI_RS_182584	86.37
4H	SCRI_RS_207853	86.37
4H	SCRI_RS_209508	86.37
4H	SCRI_RS_235688	86.37
4H	11_11470	86.37
4H	SCRI_RS_156130	86.56
4H	SCRI_RS_197256	86.56
4H	12_11183	86.56
4H	11_10614	86.56
4H	12_30539	86.56
4H	12_30987	86.56
4H	12_30988	86.56
4H	11_20515	86.78
4H	11_10510	87.44
4H	11_10334	88.51
4H	11_21111	88.51
4H	12_31139	89.17
4H	11_20044	89.93
4H	SCRI_RS_157760	90.6
4H	11_20974	90.63
4H	SCRI_RS_165236	90.8
4H	SCRI_RS_188944	90.87
4H	SCRI_RS_25685	91.36
4H	12_11194	91.56
4H	SCRI_RS_121084	92.5
4H	SCRI_RS_142683	92.5
4H	12_30385	92.9
4H	12_31138	92.9
4H	11_11299	93.57
4H	SCRI_RS_108369	94.24
4H	SCRI_RS_172684	97.24
4H	SCRI_RS_122057	97.31
4H	SCRI_RS_153184	97.31
4H	SCRI_RS_169580	97.31
4H	SCRI_RS_193252	97.31
4H	SCRI_RS_3281	97.31
4H	SCRI_RS_228030	98.5
4H	SCRI_RS_97755	98.5
4H	SCRI_RS_144761	98.73
4H	SCRI_RS_130863	99.08
4H	SCRI_RS_62678	99.08
4H	12_30989	99.65
4H	SCRI_RS_237744	101.21
4H	SCRI_RS_10818	101.8
4H	SCRI_RS_224674	101.8
4H	SCRI_RS_200445	102.3
4H	SCRI_RS_170388	102.3
4H	SCRI_RS_9549	102.5
4H	SCRI_RS_160461	102.51
4H	SCRI_RS_161327	102.51
4H	SCRI_RS_220391	102.51
4H	SCRI_RS_148709	103.61
4H	SCRI_RS_188177	103.61
4H	SCRI_RS_157866	103.94
4H	11_10123	104.76
4H	11_10387	104.76
4H	12_11233	104.76
4H	12_20237	104.76
4H	12_30006	104.76
4H	11_10712	106.11
4H	11_20701	106.85
4H	12_11235	106.85
4H	11_10150	107.08
4H	SCRI_RS_229116	107.85
4H	11_21035	107.88
4H	11_10611	108.91
4H	11_10697	108.91
4H	12_30146	108.91
4H	SCRI_RS_196076	109.9
4H	SCRI_RS_170170	110.4
4H	11_21130	110.54
4H	12_10337	110.72
4H	SCRI_RS_216855	110.83
4H	SCRI_RS_76975	110.83
4H	SCRI_RS_113536	110.94
4H	SCRI_RS_192456	111
4H	12_31200	111.04
4H	SCRI_RS_149172	111.04
4H	SCRI_RS_179695	111.2
4H	SCRI_RS_198507	111.2
4H	SCRI_RS_148782	111.65
4H	SCRI_RS_171481	111.65
4H	SCRI_RS_2513	111.65
4H	SCRI_RS_66181	111.65
4H	11_10269	112.03
4H	11_20224	112.03
4H	11_21210	112.03
4H	11_21534	112.03
4H	11_10610	112.65
4H	11_20272	112.65
4H	12_21450	112.65
4H	12_30476	112.65
4H	12_30873	112.65
4H	12_31151	112.65
4H	12_31219	112.65
4H	SCRI_RS_13454	112.65
4H	SCRI_RS_157974	112.65
4H	SCRI_RS_189457	112.65
4H	SCRI_RS_214071	112.65
4H	SCRI_RS_216897	112.65
4H	SCRI_RS_221876	112.65
4H	SCRI_RS_222098	112.65
4H	SCRI_RS_238901	112.65
4H	SCRI_RS_8250	112.65
4H	SCRI_RS_99798	112.65
4H	12_10032	112.65
4H	SCRI_RS_124791	112.65
4H	SCRI_RS_140963	112.65
4H	SCRI_RS_142792	112.65
4H	SCRI_RS_145343	112.65
4H	SCRI_RS_152271	112.65
4H	SCRI_RS_188943	112.65
4H	SCRI_RS_218891	112.65
4H	SCRI_RS_7809	112.65
4H	11_20007	112.65
4H	12_30889	112.65
4H	12_30892	112.65
4H	12_30884	112.65
4H	12_30891	112.65
4H	12_30239	113.4
4H	12_31422	113.4
4H	SCRI_RS_110912	113.4
4H	SCRI_RS_119390	113.4
4H	SCRI_RS_13970	113.4
4H	SCRI_RS_142177	113.4
4H	SCRI_RS_142924	113.4
4H	SCRI_RS_149609	113.4
4H	SCRI_RS_157125	113.4
4H	SCRI_RS_157277	113.4
4H	SCRI_RS_181566	113.4
4H	SCRI_RS_189599	113.4
4H	SCRI_RS_235762	113.4
4H	SCRI_RS_222936	113.4
4H	SCRI_RS_9164	113.4
4H	12_30886	113.4
4H	SCRI_RS_99965	114.4
4H	11_11186	114.45
4H	SCRI_RS_151357	114.6
4H	SCRI_RS_188829	114.9
4H	11_20089	115.75
4H	11_20668	119.83
4H	11_20013	120.61
4H	11_11019	120.61
4H	SCRI_RS_111	121.06
4H	SCRI_RS_133334	121.06
4H	SCRI_RS_136981	121.06
4H	SCRI_RS_188329	121.06
4H	12_30825	122.08
4H	12_30824	122.08
5H	11_20894	0
5H	11_20553	0.65
5H	SCRI_RS_131198	0.65
5H	12_30543	0.65
5H	SCRI_RS_137053	0.65
5H	SCRI_RS_171486	0.65
5H	12_30981	0.79
5H	12_30982	0.79
5H	12_31022	1.07
5H	12_30979	1.17
5H	11_20226	1.17
5H	12_30976	1.17
5H	12_30978	1.17
5H	12_30980	1.17
5H	SCRI_RS_141591	1.17
5H	SCRI_RS_147811	1.17
5H	12_20257	1.17
5H	12_30977	1.28
5H	12_31326	1.28
5H	SCRI_RS_209607	1.28
5H	12_30975	1.28
5H	12_31023	1.28
5H	SCRI_RS_10929	1.28
5H	SCRI_RS_109375	1.28
5H	SCRI_RS_133909	1.28
5H	SCRI_RS_179411	1.28
5H	12_30983	1.28
5H	12_30984	1.28
5H	12_30985	1.28
5H	SCRI_RS_236068	1.28
5H	SCRI_RS_206456	1.28
5H	SCRI_RS_31797	1.38
5H	12_30591	2.28
5H	SCRI_RS_168359	2.48
5H	SCRI_RS_236640	2.98
5H	11_20206	3.38
5H	11_21221	3.38
5H	11_21202	4.48
5H	SCRI_RS_114102	7.78
5H	SCRI_RS_155555	7.78
5H	SCRI_RS_169138	8.17
5H	SCRI_RS_179365	8.17
5H	SCRI_RS_225187	8.17
5H	SCRI_RS_228061	8.17
5H	SCRI_RS_181570	8.48
5H	SCRI_RS_214760	8.48
5H	SCRI_RS_225420	8.48
5H	12_31094	8.5
5H	SCRI_RS_204607	8.5
5H	11_20010	9.44
5H	SCRI_RS_143952	9.98
5H	12_30714	11.18
5H	SCRI_RS_108541	11.18
5H	SCRI_RS_133600	11.18
5H	SCRI_RS_150014	11.18
5H	SCRI_RS_179260	11.18
5H	SCRI_RS_182131	11.18
5H	SCRI_RS_184564	11.18
5H	SCRI_RS_192396	11.18
5H	SCRI_RS_232930	11.18
5H	SCRI_RS_98293	11.18
5H	11_21365	12.79
5H	SCRI_RS_149877	13.13
5H	12_30167	16.46
5H	SCRI_RS_157444	16.46
5H	SCRI_RS_228431	16.46
5H	11_10695	16.46
5H	11_20873	16.76
5H	11_21065	17.87
5H	11_21426	18.41
5H	12_30531	19.44
5H	11_11048	21.49
5H	SCRI_RS_189323	21.49
5H	SCRI_RS_194819	21.49
5H	SCRI_RS_85089	21.49
5H	11_20386	21.49
5H	11_21324	22.15
5H	12_20576	22.22
5H	SCRI_RS_136706	22.5
5H	SCRI_RS_192515	22.5
5H	SCRI_RS_203585	22.5
5H	SCRI_RS_7720	22.5
5H	SCRI_RS_189874	22.5
5H	SCRI_RS_221631	22.5
5H	SCRI_RS_187759	22.56
5H	11_20761	24.12
5H	12_10530	24.64
5H	SCRI_RS_108416	25.67
5H	SCRI_RS_190770	25.67
5H	SCRI_RS_207889	25.67
5H	SCRI_RS_4717	25.67
5H	SCRI_RS_92500	25.67
5H	11_10688	25.71
5H	12_10499	25.71
5H	SCRI_RS_166209	25.71
5H	SCRI_RS_179512	25.71
5H	11_10580	26.63
5H	SCRI_RS_175087	27.08
5H	SCRI_RS_156295	27.85
5H	SCRI_RS_179526	27.85
5H	SCRI_RS_220164	27.85
5H	11_10621	27.93
5H	12_30410	27.93
5H	SCRI_RS_205100	27.96
5H	SCRI_RS_227892	27.99
5H	SCRI_RS_27067	29.31
5H	SCRI_RS_203117	29.31
5H	SCRI_RS_104699	29.45
5H	SCRI_RS_144042	29.45
5H	SCRI_RS_168039	29.45
5H	SCRI_RS_156016	29.46
5H	SCRI_RS_205508	29.77
5H	11_20845	30.28
5H	11_20980	30.28
5H	11_21391	30.28
5H	11_20730	30.44
5H	SCRI_RS_220165	30.83
5H	SCRI_RS_3114	31.37
5H	12_11318	31.42
5H	SCRI_RS_126187	31.67
5H	12_10864	32.12
5H	12_30838	32.12
5H	12_20770	32.61
5H	11_21207	32.95
5H	12_30707	33.45
5H	11_20729	33.96
5H	11_10260	35.51
5H	11_10955	35.51
5H	11_11432	35.51
5H	11_20571	35.51
5H	11_20766	35.51
5H	11_20903	35.51
5H	11_20987	35.51
5H	12_30105	35.51
5H	12_30654	35.51
5H	12_31312	35.51
5H	12_31492	35.51
5H	12_21372	36.21
5H	12_31257	36.21
5H	11_11198	36.87
5H	11_21401	36.87
5H	12_10923	36.87
5H	12_31155	36.87
5H	SCRI_RS_171189	37.97
5H	SCRI_RS_175090	37.97
5H	11_10116	38.18
5H	11_20101	38.18
5H	11_20841	38.18
5H	11_21447	38.18
5H	12_30354	38.18
5H	12_30454	38.18
5H	12_30729	38.18
5H	12_31390	38.18
5H	12_31423	38.18
5H	12_31512	38.18
5H	11_20697	38.6
5H	SCRI_RS_157728	39.04
5H	SCRI_RS_163973	39.04
5H	SCRI_RS_175557	39.04
5H	SCRI_RS_204593	39.04
5H	11_10058	39.14
5H	11_10157	39.14
5H	11_10177	39.14
5H	11_10240	39.14
5H	11_10252	39.14
5H	11_10318	39.14
5H	11_10481	39.14
5H	11_10661	39.14
5H	11_10856	39.14
5H	11_10913	39.14
5H	11_10995	39.14
5H	11_11469	39.14
5H	11_20129	39.14
5H	11_20461	39.14
5H	11_20524	39.14
5H	11_20708	39.14
5H	11_20958	39.14
5H	11_21011	39.14
5H	11_21040	39.14
5H	11_21116	39.14
5H	11_21215	39.14
5H	11_21350	39.14
5H	12_11399	39.14
5H	12_11462	39.14
5H	12_30109	39.14
5H	12_30193	39.14
5H	12_30337	39.14
5H	12_30408	39.14
5H	12_30575	39.14
5H	12_30709	39.14
5H	12_30717	39.14
5H	12_30728	39.14
5H	12_30768	39.14
5H	12_30792	39.14
5H	12_31259	39.14
5H	12_31317	39.14
5H	12_31520	39.14
5H	11_11260	39.88
5H	SCRI_RS_12495	39.91
5H	SCRI_RS_133674	39.91
5H	SCRI_RS_150377	39.91
5H	SCRI_RS_157065	39.91
5H	SCRI_RS_159069	39.91
5H	SCRI_RS_179958	39.91
5H	SCRI_RS_182015	39.91
5H	SCRI_RS_197817	39.91
5H	SCRI_RS_197942	39.91
5H	SCRI_RS_216814	39.91
5H	SCRI_RS_229712	39.91
5H	SCRI_RS_89108	39.91
5H	SCRI_RS_98248	39.91
5H	SCRI_RS_220645	39.91
5H	SCRI_RS_101263	40.22
5H	SCRI_RS_114729	40.22
5H	SCRI_RS_160471	40.71
5H	SCRI_RS_222345	40.71
5H	11_21318	40.96
5H	SCRI_RS_145348	41.03
5H	SCRI_RS_151191	41.03
5H	SCRI_RS_160999	41.03
5H	SCRI_RS_176142	41.03
5H	SCRI_RS_185617	41.03
5H	SCRI_RS_216810	41.03
5H	SCRI_RS_187735	41.17
5H	12_31521	41.17
5H	SCRI_RS_133662	41.17
5H	SCRI_RS_216162	41.17
5H	SCRI_RS_187842	41.17
5H	SCRI_RS_207769	41.17
5H	SCRI_RS_171426	41.23
5H	SCRI_RS_220205	41.23
5H	SCRI_RS_103840	41.37
5H	SCRI_RS_171812	41.37
5H	SCRI_RS_189402	41.37
5H	SCRI_RS_68142	41.37
5H	SCRI_RS_138811	41.37
5H	SCRI_RS_159315	41.37
5H	12_31410	41.37
5H	SCRI_RS_138727	41.37
5H	SCRI_RS_101469	42.37
5H	SCRI_RS_10219	42.37
5H	SCRI_RS_130708	42.37
5H	SCRI_RS_133538	42.37
5H	SCRI_RS_137866	42.37
5H	SCRI_RS_141949	42.37
5H	SCRI_RS_142259	42.37
5H	SCRI_RS_142549	42.37
5H	SCRI_RS_145129	42.37
5H	SCRI_RS_148312	42.37
5H	SCRI_RS_153553	42.37
5H	SCRI_RS_165927	42.37
5H	SCRI_RS_166369	42.37
5H	SCRI_RS_175284	42.37
5H	SCRI_RS_187764	42.37
5H	SCRI_RS_188359	42.37
5H	SCRI_RS_192520	42.37
5H	SCRI_RS_219574	42.37
5H	SCRI_RS_223100	42.37
5H	SCRI_RS_238458	42.37
5H	SCRI_RS_240108	42.37
5H	SCRI_RS_102075	42.37
5H	SCRI_RS_13902	42.37
5H	SCRI_RS_151755	42.37
5H	SCRI_RS_153139	42.37
5H	SCRI_RS_153463	42.37
5H	SCRI_RS_156505	42.37
5H	SCRI_RS_161386	42.37
5H	SCRI_RS_170957	42.37
5H	SCRI_RS_218181	42.37
5H	SCRI_RS_231784	42.37
5H	SCRI_RS_238123	42.37
5H	SCRI_RS_3544	42.37
5H	SCRI_RS_8076	42.37
5H	SCRI_RS_109468	42.37
5H	SCRI_RS_129414	42.37
5H	SCRI_RS_168105	42.37
5H	SCRI_RS_175366	42.37
5H	SCRI_RS_175637	42.37
5H	SCRI_RS_181514	42.37
5H	SCRI_RS_209980	42.37
5H	SCRI_RS_103377	42.37
5H	SCRI_RS_134225	42.37
5H	SCRI_RS_137940	42.37
5H	SCRI_RS_139961	42.37
5H	SCRI_RS_150023	42.37
5H	SCRI_RS_154965	42.37
5H	SCRI_RS_156276	42.37
5H	SCRI_RS_157294	42.37
5H	SCRI_RS_162380	42.37
5H	SCRI_RS_166343	42.37
5H	SCRI_RS_169826	42.37
5H	SCRI_RS_173527	42.37
5H	SCRI_RS_1928	42.37
5H	SCRI_RS_203996	42.37
5H	SCRI_RS_209553	42.37
5H	SCRI_RS_219788	42.37
5H	SCRI_RS_221695	42.37
5H	SCRI_RS_223318	42.37
5H	SCRI_RS_236446	42.37
5H	SCRI_RS_8464	42.37
5H	11_11128	42.37
5H	11_11506	42.37
5H	11_20332	42.37
5H	11_20396	42.37
5H	11_20737	42.37
5H	11_21260	42.37
5H	SCRI_RS_135425	42.37
5H	SCRI_RS_151061	42.37
5H	SCRI_RS_161107	42.37
5H	SCRI_RS_162688	42.37
5H	SCRI_RS_182407	42.37
5H	SCRI_RS_204494	42.37
5H	SCRI_RS_211940	42.37
5H	SCRI_RS_224213	42.37
5H	SCRI_RS_232881	42.37
5H	SCRI_RS_236595	42.37
5H	SCRI_RS_41519	42.37
5H	SCRI_RS_88410	42.37
5H	SCRI_RS_132280	42.37
5H	SCRI_RS_134444	42.37
5H	SCRI_RS_136857	42.37
5H	SCRI_RS_137196	42.37
5H	SCRI_RS_137838	42.37
5H	SCRI_RS_144850	42.37
5H	SCRI_RS_145116	42.37
5H	SCRI_RS_145275	42.37
5H	SCRI_RS_147081	42.37
5H	SCRI_RS_148798	42.37
5H	SCRI_RS_149232	42.37
5H	SCRI_RS_149440	42.37
5H	SCRI_RS_151815	42.37
5H	SCRI_RS_156086	42.37
5H	SCRI_RS_156499	42.37
5H	SCRI_RS_157301	42.37
5H	SCRI_RS_159021	42.37
5H	SCRI_RS_16164	42.37
5H	SCRI_RS_161655	42.37
5H	SCRI_RS_167575	42.37
5H	SCRI_RS_172679	42.37
5H	SCRI_RS_186690	42.37
5H	SCRI_RS_197270	42.37
5H	SCRI_RS_200057	42.37
5H	SCRI_RS_208177	42.37
5H	SCRI_RS_218911	42.37
5H	SCRI_RS_236744	42.37
5H	SCRI_RS_236777	42.37
5H	SCRI_RS_237910	42.37
5H	SCRI_RS_238720	42.37
5H	SCRI_RS_239393	42.37
5H	SCRI_RS_4724	42.37
5H	SCRI_RS_57982	42.37
5H	SCRI_RS_63610	42.37
5H	SCRI_RS_6422	42.37
5H	SCRI_RS_91468	42.37
5H	11_21536	42.37
5H	12_10508	42.37
5H	12_20059	42.37
5H	12_30214	42.37
5H	SCRI_RS_119692	42.37
5H	SCRI_RS_144101	42.37
5H	SCRI_RS_148870	42.37
5H	SCRI_RS_152211	42.37
5H	SCRI_RS_160321	42.37
5H	SCRI_RS_161221	42.37
5H	SCRI_RS_174946	42.37
5H	SCRI_RS_178739	42.37
5H	SCRI_RS_186342	42.37
5H	SCRI_RS_189	42.37
5H	SCRI_RS_189103	42.37
5H	SCRI_RS_195073	42.37
5H	SCRI_RS_203128	42.37
5H	SCRI_RS_205661	42.37
5H	SCRI_RS_206910	42.37
5H	SCRI_RS_220101	42.37
5H	SCRI_RS_229980	42.37
5H	SCRI_RS_232700	42.37
5H	SCRI_RS_234579	42.37
5H	SCRI_RS_236984	42.37
5H	SCRI_RS_237877	42.37
5H	SCRI_RS_153273	42.37
5H	SCRI_RS_194453	42.37
5H	SCRI_RS_13571	42.37
5H	SCRI_RS_204786	42.37
5H	SCRI_RS_238498	42.37
5H	SCRI_RS_134286	42.37
5H	SCRI_RS_166374	42.37
5H	SCRI_RS_167468	42.37
5H	SCRI_RS_62692	42.37
5H	12_31517	42.37
5H	SCRI_RS_141915	42.37
5H	SCRI_RS_142253	42.37
5H	SCRI_RS_206932	42.37
5H	SCRI_RS_213606	42.37
5H	SCRI_RS_226185	42.37
5H	12_30409	42.37
5H	SCRI_RS_206879	42.37
5H	SCRI_RS_98043	42.37
5H	SCRI_RS_204805	42.37
5H	12_31431	42.37
5H	SCRI_RS_136637	42.37
5H	SCRI_RS_13395	42.37
5H	SCRI_RS_153634	42.37
5H	SCRI_RS_193051	42.37
5H	SCRI_RS_225263	42.37
5H	SCRI_RS_235721	42.37
5H	SCRI_RS_234339	42.37
5H	12_31117	42.37
5H	SCRI_RS_206061	42.44
5H	11_20239	42.47
5H	12_10264	42.47
5H	11_20105	43.17
5H	SCRI_RS_156396	43.17
5H	SCRI_RS_180342	43.17
5H	SCRI_RS_13353	43.66
5H	12_10079	43.73
5H	11_10671	43.73
5H	11_10840	43.73
5H	11_20372	43.73
5H	12_30644	43.73
5H	12_30700	43.73
5H	12_30747	43.73
5H	SCRI_RS_119781	43.73
5H	SCRI_RS_122965	43.73
5H	SCRI_RS_133042	43.73
5H	SCRI_RS_13964	43.73
5H	SCRI_RS_144696	43.73
5H	SCRI_RS_153476	43.73
5H	SCRI_RS_164250	43.73
5H	SCRI_RS_165878	43.73
5H	SCRI_RS_182435	43.73
5H	SCRI_RS_199682	43.73
5H	SCRI_RS_215787	43.73
5H	SCRI_RS_220389	43.73
5H	SCRI_RS_234598	43.73
5H	SCRI_RS_235908	43.73
5H	SCRI_RS_75826	43.73
5H	11_20306	43.73
5H	SCRI_RS_134798	43.73
5H	SCRI_RS_147429	43.73
5H	SCRI_RS_147462	43.73
5H	SCRI_RS_150803	43.73
5H	SCRI_RS_157305	43.73
5H	SCRI_RS_159056	43.73
5H	SCRI_RS_161222	43.73
5H	SCRI_RS_171243	43.73
5H	SCRI_RS_182177	43.73
5H	SCRI_RS_204616	43.73
5H	SCRI_RS_209398	43.73
5H	SCRI_RS_223712	43.73
5H	SCRI_RS_231244	43.73
5H	SCRI_RS_235638	43.73
5H	12_30538	43.73
5H	12_31183	43.73
5H	SCRI_RS_114549	43.73
5H	SCRI_RS_120914	43.73
5H	SCRI_RS_162444	43.73
5H	SCRI_RS_182320	43.73
5H	SCRI_RS_225149	43.73
5H	SCRI_RS_179363	43.73
5H	12_20416	43.73
5H	SCRI_RS_166456	43.73
5H	12_10306	43.73
5H	BK_11	43.73
5H	12_31503	43.73
5H	12_10899	43.87
5H	12_31340	43.87
5H	12_10034	43.87
5H	11_20501	43.87
5H	11_20283	43.87
5H	11_20961	43.87
5H	11_11221	43.87
5H	11_21200	43.87
5H	SCRI_RS_186111	43.87
5H	SCRI_RS_188367	43.87
5H	SCRI_RS_236759	43.87
5H	12_30515	43.87
5H	SCRI_RS_160506	43.87
5H	SCRI_RS_114487	43.87
5H	SCRI_RS_129810	43.87
5H	SCRI_RS_141756	43.87
5H	SCRI_RS_143321	43.87
5H	SCRI_RS_145591	43.87
5H	SCRI_RS_148280	43.87
5H	SCRI_RS_158981	43.87
5H	SCRI_RS_165313	43.87
5H	SCRI_RS_170761	43.87
5H	SCRI_RS_197571	43.87
5H	SCRI_RS_201097	43.87
5H	SCRI_RS_205842	43.87
5H	SCRI_RS_212428	43.87
5H	SCRI_RS_212505	43.87
5H	SCRI_RS_217769	43.87
5H	SCRI_RS_239809	43.87
5H	SCRI_RS_7189	43.87
5H	SCRI_RS_90293	43.87
5H	SCRI_RS_8405	43.87
5H	SCRI_RS_161711	43.87
5H	11_11240	43.87
5H	11_21244	43.87
5H	12_11151	43.87
5H	12_11363	43.87
5H	12_11512	43.87
5H	12_20981	43.87
5H	12_30111	43.87
5H	12_31062	43.87
5H	12_31064	43.87
5H	12_31451	43.87
5H	SCRI_RS_165468	44.14
5H	12_10725	44.6
5H	SCRI_RS_144202	44.8
5H	12_11089	44.83
5H	12_31034	44.83
5H	11_11159	44.83
5H	11_21508	44.83
5H	12_11385	44.83
5H	12_30011	44.83
5H	12_31033	44.83
5H	12_31280	44.83
5H	SCRI_RS_157037	44.87
5H	SCRI_RS_134928	44.94
5H	SCRI_RS_127712	45.22
5H	12_31240	45.62
5H	SCRI_RS_137893	45.62
5H	SCRI_RS_144841	45.62
5H	SCRI_RS_168185	45.62
5H	SCRI_RS_181722	45.62
5H	SCRI_RS_221282	45.62
5H	11_20265	45.81
5H	11_21344	45.81
5H	SCRI_RS_215485	45.98
5H	SCRI_RS_215710	46.01
5H	SCRI_RS_224461	46.01
5H	11_20441	46.1
5H	SCRI_RS_1386	46.1
5H	SCRI_RS_161414	46.1
5H	SCRI_RS_167829	46.1
5H	SCRI_RS_171407	46.1
5H	SCRI_RS_174091	46.1
5H	SCRI_RS_174326	46.1
5H	SCRI_RS_213753	46.1
5H	SCRI_RS_223699	46.1
5H	SCRI_RS_7368	46.1
5H	SCRI_RS_119287	46.1
5H	SCRI_RS_125930	46.1
5H	SCRI_RS_162773	46.1
5H	SCRI_RS_171155	46.1
5H	SCRI_RS_173717	46.1
5H	SCRI_RS_182321	46.1
5H	SCRI_RS_196041	46.1
5H	SCRI_RS_198009	46.1
5H	SCRI_RS_222785	46.1
5H	SCRI_RS_231238	46.1
5H	11_11281	46.74
5H	12_30745	47.26
5H	SCRI_RS_169088	47.37
5H	SCRI_RS_211904	47.37
5H	SCRI_RS_137249	47.41
5H	SCRI_RS_147940	47.61
5H	SCRI_RS_153533	47.61
5H	SCRI_RS_1362	47.72
5H	SCRI_RS_232876	47.72
5H	11_20370	48.16
5H	SCRI_RS_128287	48.16
5H	SCRI_RS_135464	48.16
5H	SCRI_RS_148395	48.16
5H	SCRI_RS_157941	48.16
5H	11_20713	48.59
5H	SCRI_RS_8666	49.57
5H	11_21121	49.71
5H	SCRI_RS_145361	49.71
5H	SCRI_RS_49958	49.71
5H	12_21036	49.71
5H	12_31084	49.71
5H	11_21275	49.71
5H	SCRI_RS_7112	49.88
5H	SCRI_RS_161503	50.37
5H	12_10203	51.54
5H	12_11438	51.54
5H	12_20278	51.54
5H	11_10641	51.54
5H	11_21239	51.86
5H	12_30080	52.03
5H	11_20392	52.03
5H	11_11249	52.03
5H	12_30007	52.03
5H	12_30966	52.03
5H	SCRI_RS_221999	53.17
5H	SCRI_RS_205235	53.17
5H	12_20350	53.7
5H	12_10548	54.44
5H	12_20297	55.15
5H	12_30719	55.15
5H	12_10953	55.75
5H	11_20367	55.75
5H	11_21309	56.59
5H	11_21001	56.59
5H	12_20818	56.61
5H	12_21157	58.03
5H	SCRI_RS_159611	59.21
5H	11_10183	59.48
5H	11_21133	59.48
5H	SCRI_RS_166296	59.81
5H	11_20236	60.03
5H	12_21128	60.03
5H	11_10127	60.03
5H	12_10634	60.03
5H	SCRI_RS_72965	60.03
5H	12_10633	61.11
5H	SCRI_RS_140294	61.61
5H	11_20096	61.61
5H	11_10167	61.61
5H	SCRI_RS_194572	61.61
5H	SCRI_RS_156310	61.61
5H	12_10140	62.64
5H	11_20246	63.21
5H	SCRI_RS_227918	63.21
5H	11_20736	63.39
5H	12_11515	63.39
5H	SCRI_RS_207423	64.76
5H	SCRI_RS_128664	65.43
5H	11_20645	65.54
5H	11_21445	65.54
5H	12_30314	65.54
5H	SCRI_RS_173942	66.6
5H	SCRI_RS_111826	67.61
5H	SCRI_RS_152347	67.61
5H	11_11355	68.46
5H	SCRI_RS_12887	68.7
5H	11_21480	68.9
5H	SCRI_RS_1474	68.9
5H	SCRI_RS_160332	68.9
5H	12_10674	70.23
5H	12_31427	70.23
5H	SCRI_RS_165919	70.23
5H	SCRI_RS_170734	70.23
5H	SCRI_RS_203938	70.23
5H	SCRI_RS_233239	70.23
5H	12_31361	70.89
5H	SCRI_RS_11206	71.58
5H	SCRI_RS_219608	71.58
5H	SCRI_RS_140487	71.78
5H	SCRI_RS_240114	72.97
5H	11_10524	73.13
5H	SCRI_RS_212784	73.88
5H	SCRI_RS_91994	73.88
5H	12_10930	73.88
5H	11_21150	73.88
5H	SCRI_RS_196566	73.88
5H	SCRI_RS_224839	73.88
5H	12_21497	73.98
5H	SCRI_RS_235416	74.16
5H	12_20359	74.16
5H	SCRI_RS_170151	74.86
5H	11_10578	75.06
5H	11_20497	75.38
5H	SCRI_RS_157991	75.5
5H	SCRI_RS_177017	75.5
5H	SCRI_RS_205853	75.5
5H	12_11134	76.89
5H	11_20449	78.26
5H	11_10518	78.26
5H	11_20097	78.26
5H	12_30533	78.26
5H	SCRI_RS_147819	78.26
5H	SCRI_RS_153937	78.26
5H	SCRI_RS_179268	78.26
5H	SCRI_RS_204275	78.26
5H	SCRI_RS_206848	78.26
5H	SCRI_RS_206867	78.26
5H	11_10771	78.26
5H	11_20526	78.26
5H	12_31271	78.26
5H	SCRI_RS_150410	78.26
5H	SCRI_RS_220136	78.26
5H	SCRI_RS_239097	78.26
5H	SCRI_RS_7201	78.26
5H	SCRI_RS_192717	78.26
5H	SCRI_RS_232871	78.26
5H	SCRI_RS_155585	78.4
5H	12_30182	79.02
5H	11_11473	79.07
5H	SCRI_RS_143814	79.07
5H	12_10408	79.77
5H	SCRI_RS_10386	80.95
5H	SCRI_RS_240059	80.95
5H	11_10622	81.03
5H	SCRI_RS_146093	81.36
5H	SCRI_RS_157881	81.36
5H	SCRI_RS_172547	81.46
5H	SCRI_RS_4923	81.46
5H	11_20850	81.46
5H	11_21421	81.68
5H	SCRI_RS_169005	82.12
5H	SCRI_RS_156633	82.25
5H	11_10414	82.92
5H	11_20327	82.92
5H	SCRI_RS_224899	83.24
5H	11_11350	83.36
5H	11_20018	83.36
5H	SCRI_RS_140054	83.36
5H	12_20403	84.1
5H	12_30098	84.1
5H	11_20134	84.81
5H	11_20549	84.81
5H	12_10077	84.81
5H	11_20795	85.22
5H	SCRI_RS_158235	85.59
5H	12_30962	85.59
5H	12_31279	85.59
5H	SCRI_RS_168467	85.59
5H	SCRI_RS_238417	85.73
5H	11_10024	85.82
5H	11_21314	85.82
5H	12_30855	85.82
5H	12_31417	85.82
5H	11_21321	87.19
5H	12_10844	87.19
5H	SCRI_RS_218201	87.49
5H	11_10834	87.9
5H	11_21168	87.9
5H	12_30846	87.9
5H	12_30850	87.9
5H	12_30852	87.9
5H	12_30854	87.9
5H	SCRI_RS_161381	88.31
5H	11_20320	88.55
5H	SCRI_RS_219728	89.02
5H	11_21061	89.09
5H	SCRI_RS_169596	89.4
5H	SCRI_RS_230566	89.4
5H	SCRI_RS_11368	91.64
5H	SCRI_RS_143508	91.64
5H	SCRI_RS_159430	91.64
5H	SCRI_RS_147762	91.64
5H	SCRI_RS_1619	91.64
5H	SCRI_RS_143514	91.7
5H	SCRI_RS_3280	91.7
5H	SCRI_RS_169223	91.76
5H	SCRI_RS_127785	92.59
5H	11_20805	92.71
5H	12_10507	92.71
5H	12_30705	92.71
5H	12_30848	92.83
5H	BK_22	92.83
5H	SCRI_RS_138826	92.83
5H	SCRI_RS_149088	92.83
5H	SCRI_RS_165331	92.83
5H	SCRI_RS_224501	92.83
5H	SCRI_RS_236569	92.83
5H	11_11361	92.83
5H	12_30849	92.83
5H	SCRI_RS_189093	92.83
5H	SCRI_RS_236939	92.83
5H	BK_23	92.97
5H	BK_21	92.97
5H	SCRI_RS_112985	92.97
5H	12_10752	93.44
5H	SCRI_RS_237352	93.63
5H	SCRI_RS_13960	94.09
5H	SCRI_RS_165215	94.09
5H	SCRI_RS_224346	94.09
5H	SCRI_RS_157181	94.57
5H	SCRI_RS_157334	94.74
5H	SCRI_RS_192377	94.74
5H	SCRI_RS_140846	94.74
5H	SCRI_RS_157430	94.74
5H	SCRI_RS_174177	94.74
5H	SCRI_RS_2303	94.74
5H	SCRI_RS_206565	94.79
5H	11_11273	95.39
5H	SCRI_RS_137242	95.43
5H	SCRI_RS_123677	95.49
5H	SCRI_RS_125580	95.49
5H	SCRI_RS_125581	95.49
5H	SCRI_RS_142217	95.49
5H	SCRI_RS_152849	95.49
5H	SCRI_RS_171047	95.49
5H	SCRI_RS_196858	95.49
5H	SCRI_RS_206460	95.49
5H	11_21390	96.06
5H	SCRI_RS_138221	96.29
5H	SCRI_RS_157897	96.29
5H	SCRI_RS_225268	96.69
5H	SCRI_RS_214241	96.69
5H	11_10477	96.77
5H	12_30056	96.77
5H	SCRI_RS_119308	96.77
5H	SCRI_RS_44800	96.77
5H	SCRI_RS_120829	96.89
5H	SCRI_RS_235443	96.89
5H	SCRI_RS_45011	96.89
5H	12_30456	96.89
5H	SCRI_RS_136777	96.89
5H	SCRI_RS_172024	97.69
5H	12_30619	97.86
5H	SCRI_RS_206793	97.86
5H	11_11341	97.86
5H	SCRI_RS_119301	99.01
5H	SCRI_RS_129893	99.01
5H	SCRI_RS_139944	99.01
5H	SCRI_RS_149479	99.01
5H	SCRI_RS_178985	99.01
5H	SCRI_RS_185563	99.01
5H	SCRI_RS_198617	99.01
5H	SCRI_RS_208647	99.01
5H	11_11200	99.06
5H	12_11245	103.09
5H	SCRI_RS_13766	103.29
5H	SCRI_RS_106306	103.3
5H	SCRI_RS_150470	103.3
5H	SCRI_RS_175290	103.3
5H	SCRI_RS_176680	103.3
5H	SCRI_RS_214153	103.3
5H	SCRI_RS_88710	103.3
5H	11_10094	103.72
5H	11_20629	104.4
5H	12_11298	105.03
5H	11_20637	105.34
5H	SCRI_RS_12723	106.16
5H	SCRI_RS_160191	106.16
5H	SCRI_RS_177133	106.16
5H	SCRI_RS_222377	106.16
5H	12_30524	106.24
5H	12_31278	106.9
5H	11_11507	107.74
5H	SCRI_RS_14097	107.89
5H	SCRI_RS_9991	108.46
5H	12_20158	108.46
5H	SCRI_RS_142618	108.46
5H	SCRI_RS_237950	108.46
5H	11_20883	108.46
5H	11_21422	108.48
5H	12_20045	109.23
5H	SCRI_RS_145435	109.7
5H	SCRI_RS_16177	109.7
5H	12_30377	110.59
5H	SCRI_RS_234720	110.61
5H	SCRI_RS_240005	110.61
5H	SCRI_RS_231239	110.75
5H	SCRI_RS_196437	110.96
5H	11_10360	111.25
5H	11_11024	111.25
5H	11_20003	111.25
5H	11_20347	111.25
5H	11_20653	111.25
5H	11_21203	111.25
5H	12_10228	111.25
5H	12_11535	111.25
5H	12_30169	111.25
5H	12_30590	111.25
5H	12_30611	111.25
5H	SCRI_RS_136812	111.8
5H	SCRI_RS_38003	111.8
5H	11_10805	111.91
5H	11_20300	111.91
5H	11_11375	112.7
5H	11_21325	112.7
5H	12_30067	113.36
5H	12_11472	113.56
5H	SCRI_RS_127692	113.6
5H	SCRI_RS_156454	113.81
5H	SCRI_RS_161728	113.81
5H	SCRI_RS_8256	113.81
5H	SCRI_RS_2831	113.81
5H	SCRI_RS_123912	113.81
5H	SCRI_RS_189923	113.81
5H	SCRI_RS_195813	113.81
5H	11_10146	114.01
5H	SCRI_RS_162696	114.09
5H	SCRI_RS_165569	114.09
5H	SCRI_RS_195217	114.09
5H	SCRI_RS_154148	114.09
5H	SCRI_RS_198525	114.19
5H	SCRI_RS_206982	114.19
5H	SCRI_RS_126419	114.36
5H	SCRI_RS_197945	114.36
5H	SCRI_RS_236583	114.36
5H	SCRI_RS_239128	114.36
5H	SCRI_RS_165273	114.36
5H	SCRI_RS_182540	114.36
5H	SCRI_RS_212515	114.36
5H	SCRI_RS_236782	114.36
5H	SCRI_RS_212523	114.36
5H	11_10705	114.67
5H	11_11090	114.67
5H	11_20259	114.67
5H	11_20298	114.67
5H	11_21177	114.67
5H	11_21247	114.67
5H	12_21471	114.67
5H	SCRI_RS_186538	115.1
5H	SCRI_RS_196673	115.1
5H	SCRI_RS_212890	115.1
5H	SCRI_RS_222698	115.1
5H	SCRI_RS_232705	115.1
5H	SCRI_RS_128712	115.1
5H	SCRI_RS_134711	115.1
5H	SCRI_RS_135254	115.1
5H	SCRI_RS_153247	115.1
5H	SCRI_RS_179388	115.1
5H	SCRI_RS_199964	115.1
5H	SCRI_RS_216743	115.1
5H	SCRI_RS_221130	115.1
5H	SCRI_RS_230675	115.1
5H	SCRI_RS_4611	115.1
5H	SCRI_RS_148876	117.24
5H	SCRI_RS_209280	118.15
5H	SCRI_RS_157896	118.19
5H	SCRI_RS_220076	118.19
5H	SCRI_RS_7191	118.19
5H	SCRI_RS_15011	118.19
5H	SCRI_RS_162590	118.19
5H	SCRI_RS_189371	118.19
5H	SCRI_RS_189046	118.19
5H	SCRI_RS_189094	118.19
5H	SCRI_RS_196147	118.19
5H	SCRI_RS_51000	118.19
5H	SCRI_RS_90041	118.19
5H	SCRI_RS_90070	118.19
5H	SCRI_RS_142564	118.19
5H	SCRI_RS_138448	118.23
5H	SCRI_RS_160288	118.33
5H	SCRI_RS_122171	118.33
5H	SCRI_RS_167563	118.55
5H	SCRI_RS_24549	118.55
5H	SCRI_RS_237805	118.61
5H	SCRI_RS_150691	118.85
5H	12_31041	119.02
5H	11_10521	119.19
5H	SCRI_RS_124472	119.2
5H	SCRI_RS_133323	119.2
5H	SCRI_RS_214550	119.2
5H	SCRI_RS_234330	119.2
5H	SCRI_RS_162933	119.2
5H	SCRI_RS_1501	119.2
5H	SCRI_RS_170159	119.2
5H	SCRI_RS_238070	119.2
5H	SCRI_RS_150232	119.3
5H	SCRI_RS_158259	119.3
5H	SCRI_RS_159474	119.3
5H	SCRI_RS_159490	119.3
5H	12_31286	119.3
5H	SCRI_RS_14626	119.3
5H	SCRI_RS_148712	119.3
5H	SCRI_RS_171993	119.8
5H	SCRI_RS_166218	119.8
5H	SCRI_RS_188785	119.8
5H	11_10783	119.9
5H	12_30883	119.9
5H	SCRI_RS_202774	120.3
5H	11_11080	120.56
5H	11_20188	120.56
5H	12_30668	120.56
5H	12_30869	120.56
5H	SCRI_RS_138146	121.2
5H	11_21241	121.22
5H	12_31202	121.3
5H	SCRI_RS_201248	121.3
5H	SCRI_RS_174710	121.46
5H	SCRI_RS_80654	121.63
5H	11_10095	121.9
5H	11_10855	121.9
5H	11_20884	121.9
5H	12_30929	121.9
5H	12_30930	121.9
5H	12_31237	121.9
5H	SCRI_RS_154144	122.06
5H	SCRI_RS_147315	122.16
5H	SCRI_RS_168141	122.16
5H	12_10179	122.16
5H	SCRI_RS_130992	122.3
5H	SCRI_RS_122458	122.3
5H	SCRI_RS_133602	122.3
5H	SCRI_RS_138933	122.3
5H	SCRI_RS_141778	122.3
5H	12_30129	122.3
5H	SCRI_RS_200080	122.3
5H	SCRI_RS_8015	122.3
5H	SCRI_RS_193529	123.52
5H	SCRI_RS_142212	123.72
5H	12_30635	125.2
5H	SCRI_RS_148402	125.86
5H	SCRI_RS_171042	125.86
5H	SCRI_RS_237494	125.86
5H	11_10845	126.52
5H	11_11071	126.52
5H	11_11532	126.52
5H	12_31366	126.52
5H	BK_17	126.84
5H	SCRI_RS_186901	126.84
5H	SCRI_RS_217212	126.84
5H	SCRI_RS_235055	126.84
5H	12_30861	126.84
5H	12_30867	126.84
5H	12_30868	126.84
5H	SCRI_RS_170843	126.84
5H	SCRI_RS_202396	126.84
5H	SCRI_RS_161471	127.04
5H	SCRI_RS_172665	127.04
5H	SCRI_RS_229673	127.04
5H	SCRI_RS_184066	127.16
5H	SCRI_RS_13320	127.72
5H	11_20551	127.83
5H	SCRI_RS_149432	128.51
5H	SCRI_RS_158089	128.51
5H	SCRI_RS_197881	128.51
5H	11_10658	128.58
5H	11_10819	128.58
5H	11_20375	128.58
5H	12_30556	128.58
5H	12_31217	129.21
5H	SCRI_RS_138532	129.4
5H	SCRI_RS_215063	129.47
5H	12_11164	129.47
5H	12_11252	129.47
5H	11_11092	129.72
5H	12_30833	129.72
5H	SCRI_RS_166857	129.77
5H	SCRI_RS_222984	129.77
5H	SCRI_RS_228463	129.87
5H	SCRI_RS_214130	130.17
5H	SCRI_RS_197258	130.27
5H	11_20008	131.06
5H	SCRI_RS_102414	131.07
5H	SCRI_RS_165578	131.07
5H	SCRI_RS_175672	131.07
5H	SCRI_RS_128407	131.07
5H	SCRI_RS_133453	131.07
5H	SCRI_RS_170205	131.07
5H	SCRI_RS_129727	131.07
5H	SCRI_RS_148120	131.07
5H	12_30222	131.21
5H	SCRI_RS_140356	131.37
5H	SCRI_RS_196175	131.37
5H	SCRI_RS_197532	131.37
5H	SCRI_RS_206956	131.37
5H	11_20676	131.46
5H	11_20731	131.46
5H	12_31182	131.46
5H	12_31234	131.46
5H	SCRI_RS_157238	131.47
5H	SCRI_RS_188141	132.87
5H	11_10080	132.87
5H	11_11441	132.87
5H	11_20568	132.87
5H	11_21077	132.87
5H	12_31050	132.87
5H	SCRI_RS_161534	132.97
5H	SCRI_RS_210928	132.97
5H	SCRI_RS_168534	133.04
5H	SCRI_RS_78648	133.04
5H	SCRI_RS_157935	133.43
5H	12_30400	133.52
5H	12_30580	133.52
5H	SCRI_RS_213086	133.77
5H	SCRI_RS_185613	133.95
5H	SCRI_RS_189650	133.95
5H	SCRI_RS_168544	133.95
5H	SCRI_RS_185612	133.95
5H	SCRI_RS_213078	133.95
5H	SCRI_RS_175848	133.95
5H	11_10557	134.18
5H	11_20100	134.18
5H	12_31206	134.18
5H	11_20791	135.48
5H	11_21297	135.48
5H	11_20388	136.13
5H	SCRI_RS_4658	136.59
5H	SCRI_RS_123457	136.59
5H	SCRI_RS_124040	136.59
5H	SCRI_RS_149936	136.95
5H	SCRI_RS_167592	136.95
5H	11_10363	137.07
5H	11_21360	137.07
5H	12_10333	137.07
5H	12_10904	137.07
5H	12_30062	137.07
5H	12_30183	137.07
5H	12_31165	137.07
5H	SCRI_RS_14089	137.35
5H	SCRI_RS_184603	137.35
5H	SCRI_RS_216751	137.35
5H	SCRI_RS_223157	137.35
5H	SCRI_RS_9182	137.35
5H	SCRI_RS_235550	137.75
5H	SCRI_RS_25684	137.75
5H	SCRI_RS_13262	137.85
5H	SCRI_RS_138735	137.85
5H	SCRI_RS_146573	137.85
5H	SCRI_RS_161550	137.85
5H	SCRI_RS_180920	137.85
5H	SCRI_RS_188572	137.85
5H	SCRI_RS_6203	137.85
5H	SCRI_RS_78626	137.85
5H	12_31096	137.98
5H	SCRI_RS_173780	137.98
5H	SCRI_RS_155999	138.04
5H	12_30795	138.16
5H	12_31221	138.16
5H	12_31109	138.42
5H	SCRI_RS_105701	138.42
5H	SCRI_RS_105705	138.45
5H	SCRI_RS_234223	138.45
5H	SCRI_RS_201831	138.45
5H	SCRI_RS_198008	138.61
5H	SCRI_RS_208686	138.61
5H	SCRI_RS_225632	139.05
5H	SCRI_RS_157026	139.05
5H	SCRI_RS_236610	139.05
5H	SCRI_RS_125258	139.19
5H	SCRI_RS_138029	139.19
5H	SCRI_RS_166491	139.19
5H	SCRI_RS_200927	139.19
5H	SCRI_RS_204797	139.19
5H	BK_04	139.29
5H	11_10217	139.52
5H	11_10589	139.52
5H	11_20104	139.52
5H	11_21355	139.52
5H	12_10016	139.52
5H	SCRI_RS_157176	139.54
5H	SCRI_RS_172379	139.54
5H	SCRI_RS_196061	139.54
5H	SCRI_RS_230112	139.54
5H	11_11490	139.87
5H	12_20186	140.23
5H	11_11497	140.93
5H	SCRI_RS_208818	141.27
5H	SCRI_RS_125263	141.65
5H	SCRI_RS_152484	141.65
5H	SCRI_RS_207000	141.65
5H	SCRI_RS_143179	141.79
5H	SCRI_RS_148696	141.79
5H	SCRI_RS_152365	141.79
5H	SCRI_RS_153322	141.79
5H	SCRI_RS_184270	141.79
5H	SCRI_RS_187056	141.79
5H	SCRI_RS_193063	141.79
5H	SCRI_RS_197361	141.79
5H	SCRI_RS_161614	141.79
5H	SCRI_RS_12707	141.79
5H	SCRI_RS_153238	141.79
5H	SCRI_RS_182353	141.79
5H	SCRI_RS_204809	141.79
5H	SCRI_RS_222839	141.79
5H	SCRI_RS_177919	141.79
5H	12_30602	141.79
5H	SCRI_RS_120876	141.79
5H	SCRI_RS_199904	141.79
5H	SCRI_RS_220146	141.79
5H	SCRI_RS_227878	141.79
5H	SCRI_RS_9455	141.79
5H	SCRI_RS_12382	141.79
5H	SCRI_RS_193078	141.79
5H	SCRI_RS_3287	141.79
5H	SCRI_RS_138608	141.99
5H	SCRI_RS_167426	141.99
5H	SCRI_RS_173583	141.99
5H	SCRI_RS_205647	141.99
5H	SCRI_RS_44795	141.99
5H	11_20573	142.51
5H	11_10901	142.51
5H	SCRI_RS_3429	142.85
5H	11_10820	143.66
5H	11_11185	143.66
5H	11_21041	143.66
5H	12_30238	143.66
5H	12_30759	143.66
5H	11_20545	144.09
5H	SCRI_RS_154163	144.62
5H	SCRI_RS_189369	144.62
5H	11_10385	144.81
5H	11_10536	144.81
5H	11_21452	144.81
5H	11_10161	145.03
5H	11_10741	145.03
5H	11_10902	145.03
5H	11_20324	145.03
5H	12_20317	145.03
5H	12_30165	145.03
5H	11_10582	145.24
5H	11_20078	145.24
5H	11_21024	145.24
5H	SCRI_RS_236933	145.32
5H	SCRI_RS_7678	145.74
5H	11_20560	145.85
5H	11_20876	145.85
5H	12_30685	146.49
5H	SCRI_RS_143385	146.49
5H	11_10251	146.54
5H	SCRI_RS_139374	146.54
5H	SCRI_RS_196483	146.54
5H	SCRI_RS_147359	146.54
5H	SCRI_RS_230034	146.6
5H	SCRI_RS_181379	146.65
5H	SCRI_RS_222314	146.65
5H	SCRI_RS_238426	146.65
5H	SCRI_RS_32778	146.65
5H	SCRI_RS_143947	146.7
5H	SCRI_RS_180871	146.7
5H	SCRI_RS_219601	146.7
5H	SCRI_RS_181376	146.7
5H	SCRI_RS_226862	146.7
5H	SCRI_RS_239368	146.7
5H	SCRI_RS_140456	146.82
5H	11_10336	147.19
5H	11_20646	147.19
5H	11_21018	147.19
5H	12_10273	147.19
5H	12_11325	147.19
5H	12_30162	147.19
5H	12_30642	147.19
5H	12_31375	147.19
5H	SCRI_RS_107772	147.27
5H	SCRI_RS_14455	147.27
5H	SCRI_RS_240105	147.27
5H	SCRI_RS_189174	147.32
5H	SCRI_RS_199888	147.32
5H	SCRI_RS_205722	147.55
5H	SCRI_RS_146309	147.57
5H	SCRI_RS_157356	147.57
5H	SCRI_RS_216071	147.66
5H	SCRI_RS_131479	147.66
5H	SCRI_RS_155688	147.76
5H	SCRI_RS_168654	147.8
5H	SCRI_RS_153575	147.8
5H	SCRI_RS_194155	147.8
5H	11_20988	147.86
5H	SCRI_RS_213163	147.9
5H	SCRI_RS_224438	147.9
5H	SCRI_RS_235092	147.9
5H	SCRI_RS_14527	147.9
5H	SCRI_RS_6917	147.9
5H	SCRI_RS_19741	148.66
5H	SCRI_RS_237478	148.8
5H	SCRI_RS_720	148.8
5H	SCRI_RS_173233	148.94
5H	11_20934	149.13
5H	11_20334	149.55
5H	SCRI_RS_171	149.87
5H	SCRI_RS_214021	149.87
5H	11_20829	150.48
5H	SCRI_RS_173935	151.04
5H	SCRI_RS_181521	151.04
5H	11_20826	151.61
5H	12_30666	151.61
5H	SCRI_RS_168668	151.74
5H	12_30566	152.26
5H	SCRI_RS_118326	152.31
5H	SCRI_RS_130982	152.36
5H	SCRI_RS_151716	152.36
5H	SCRI_RS_168091	152.36
5H	SCRI_RS_165400	152.54
5H	SCRI_RS_192640	152.54
5H	SCRI_RS_134358	152.62
5H	SCRI_RS_1458	152.62
5H	SCRI_RS_122949	152.62
5H	SCRI_RS_181059	152.62
5H	SCRI_RS_204570	152.62
5H	SCRI_RS_214011	152.62
5H	SCRI_RS_221599	152.62
5H	SCRI_RS_175560	152.75
5H	SCRI_RS_155322	152.92
5H	11_20546	153.3
5H	11_20686	153.3
5H	12_10769	153.3
5H	12_30087	153.3
5H	SCRI_RS_168083	153.65
5H	11_20644	153.73
5H	11_10593	153.94
5H	SCRI_RS_102066	153.97
5H	SCRI_RS_123668	153.97
5H	SCRI_RS_12491	153.97
5H	SCRI_RS_150540	153.97
5H	SCRI_RS_157318	153.97
5H	SCRI_RS_160975	153.97
5H	SCRI_RS_175338	153.97
5H	SCRI_RS_175743	153.97
5H	SCRI_RS_192445	153.97
5H	SCRI_RS_194030	153.97
5H	SCRI_RS_206883	153.97
5H	SCRI_RS_220002	153.97
5H	SCRI_RS_224671	153.97
5H	SCRI_RS_236958	153.97
5H	SCRI_RS_354	153.97
5H	SCRI_RS_65371	153.97
5H	SCRI_RS_726	153.97
5H	SCRI_RS_12878	154.01
5H	SCRI_RS_10924	154.05
5H	SCRI_RS_6902	154.05
5H	SCRI_RS_165835	154.05
5H	SCRI_RS_143418	154.72
5H	SCRI_RS_143426	154.72
5H	SCRI_RS_128397	154.85
5H	11_10869	154.86
5H	SCRI_RS_159482	155.14
5H	12_30716	155.35
5H	SCRI_RS_127288	155.35
5H	SCRI_RS_141271	155.35
5H	SCRI_RS_186008	155.52
5H	12_30003	155.83
5H	SCRI_RS_152085	155.83
5H	SCRI_RS_1918	155.83
5H	SCRI_RS_2824	155.83
5H	SCRI_RS_50995	155.83
5H	11_10778	156.23
5H	SCRI_RS_155101	156.59
5H	SCRI_RS_236114	156.59
5H	SCRI_RS_220005	156.59
5H	SCRI_RS_218503	156.79
5H	SCRI_RS_145388	156.79
5H	11_10600	156.98
5H	11_21012	156.98
5H	12_20867	156.98
5H	12_21290	156.98
5H	11_21141	157.48
5H	11_20536	158.02
5H	12_21009	158.02
5H	11_10870	158.25
5H	12_11010	158.72
5H	12_11450	158.72
5H	12_20816	158.72
5H	11_10254	159.3
5H	12_30656	159.3
5H	11_21138	159.83
5H	11_11489	159.99
5H	SCRI_RS_108855	159.99
5H	SCRI_RS_149420	159.99
5H	SCRI_RS_156273	159.99
5H	SCRI_RS_159467	159.99
5H	SCRI_RS_160183	159.99
5H	SCRI_RS_163091	159.99
5H	SCRI_RS_182201	159.99
5H	SCRI_RS_207260	159.99
5H	SCRI_RS_25330	159.99
5H	SCRI_RS_140499	161.14
5H	11_10736	161.16
5H	12_30494	161.16
5H	SCRI_RS_165290	161.24
5H	SCRI_RS_179841	161.24
5H	SCRI_RS_13318	161.24
5H	SCRI_RS_199694	161.24
5H	SCRI_RS_179453	161.31
5H	SCRI_RS_12757	161.31
5H	SCRI_RS_152162	161.43
5H	11_20309	161.5
5H	11_10236	162.41
5H	11_10405	162.41
5H	SCRI_RS_169845	162.89
5H	SCRI_RS_234527	162.89
5H	SCRI_RS_184542	162.89
5H	SCRI_RS_168188	162.98
5H	12_30504	163.08
5H	12_30769	163.75
5H	SCRI_RS_4965	163.88
5H	12_21135	164.3
5H	SCRI_RS_173687	164.38
5H	SCRI_RS_138797	164.5
5H	SCRI_RS_199722	164.82
5H	12_31404	164.82
5H	SCRI_RS_199298	164.82
5H	SCRI_RS_203575	164.82
5H	SCRI_RS_142656	164.82
5H	SCRI_RS_158428	164.82
5H	SCRI_RS_230046	164.82
5H	SCRI_RS_148903	165.1
5H	12_31239	165.29
5H	SCRI_RS_150686	165.29
5H	SCRI_RS_132308	165.29
5H	SCRI_RS_210006	165.29
5H	SCRI_RS_141634	165.29
5H	SCRI_RS_160831	165.29
5H	SCRI_RS_186984	165.29
5H	SCRI_RS_235652	165.29
5H	SCRI_RS_8410	165.29
5H	SCRI_RS_229416	165.63
5H	SCRI_RS_131163	166.06
5H	SCRI_RS_154756	166.06
5H	SCRI_RS_13882	166.86
5H	SCRI_RS_167103	166.86
5H	SCRI_RS_195241	166.86
5H	SCRI_RS_204598	166.86
5H	SCRI_RS_240083	166.92
5H	SCRI_RS_133010	167.06
5H	11_20022	167.48
5H	SCRI_RS_202800	167.48
5H	SCRI_RS_13914	167.48
5H	SCRI_RS_179582	167.48
5H	11_20897	167.48
5H	12_21393	167.48
5H	12_30577	167.48
5H	12_11018	167.51
5H	SCRI_RS_203880	167.58
5H	SCRI_RS_207299	167.58
5H	SCRI_RS_194125	169.43
5H	SCRI_RS_216726	169.43
5H	SCRI_RS_145394	169.62
5H	SCRI_RS_153933	169.72
5H	SCRI_RS_190416	170.72
5H	SCRI_RS_190418	170.72
5H	12_31352	170.79
5H	SCRI_RS_194337	171.82
5H	SCRI_RS_194566	172.11
5H	11_21155	172.11
5H	SCRI_RS_157762	172.11
5H	SCRI_RS_178615	172.32
5H	11_10310	172.41
5H	SCRI_RS_198007	172.41
5H	SCRI_RS_237948	172.58
5H	11_20786	172.72
5H	11_21162	172.72
5H	SCRI_RS_206768	172.72
5H	SCRI_RS_223728	172.87
5H	12_31292	172.91
5H	11_11364	173.23
5H	SCRI_RS_141787	173.27
5H	12_31267	173.41
5H	11_21108	173.77
5H	SCRI_RS_174123	173.97
5H	SCRI_RS_203132	173.97
5H	SCRI_RS_4753	173.97
5H	SCRI_RS_151407	173.97
5H	SCRI_RS_167850	173.97
5H	SCRI_RS_130320	173.97
5H	SCRI_RS_14608	173.97
5H	SCRI_RS_146241	173.97
5H	SCRI_RS_225568	173.97
5H	SCRI_RS_99344	174.17
5H	SCRI_RS_136415	174.17
5H	SCRI_RS_198142	174.17
5H	11_21052	174.25
5H	12_11192	174.25
5H	12_31210	174.25
5H	SCRI_RS_1971	174.25
5H	12_11413	174.25
5H	11_10401	174.25
5H	12_30360	174.25
5H	SCRI_RS_105868	174.25
5H	SCRI_RS_239653	174.25
5H	SCRI_RS_11024	174.39
5H	SCRI_RS_120501	174.39
5H	SCRI_RS_121526	174.39
5H	SCRI_RS_12519	174.39
5H	SCRI_RS_219619	174.39
5H	12_10732	174.39
5H	12_31481	174.39
5H	SCRI_RS_130207	174.39
5H	SCRI_RS_130212	174.39
5H	SCRI_RS_176020	174.39
5H	SCRI_RS_232575	174.49
5H	11_20402	174.99
5H	12_10857	174.99
5H	12_30382	174.99
5H	SCRI_RS_239569	174.99
5H	12_21356	174.99
5H	12_10322	174.99
5H	12_30958	174.99
5H	12_31123	174.99
5H	SCRI_RS_165349	174.99
5H	11_20132	174.99
5H	SCRI_RS_112000	174.99
5H	SCRI_RS_127921	174.99
5H	SCRI_RS_141314	174.99
5H	SCRI_RS_156598	174.99
5H	SCRI_RS_193456	174.99
5H	SCRI_RS_217776	174.99
5H	SCRI_RS_10702	174.99
5H	SCRI_RS_207382	174.99
5H	12_30502	174.99
5H	SCRI_RS_105688	174.99
6H	11_20232	0
6H	SCRI_RS_168487	0.3
6H	11_20493	0.5
6H	SCRI_RS_123065	0.5
6H	SCRI_RS_169941	0.5
6H	11_20212	1.03
6H	11_20886	1.4
6H	SCRI_RS_10770	1.4
6H	SCRI_RS_167505	1.4
6H	SCRI_RS_237565	1.4
6H	SCRI_RS_237782	1.4
6H	11_21521	2.18
6H	SCRI_RS_9560	2.2
6H	SCRI_RS_199940	2.2
6H	11_20881	2.5
6H	12_31233	2.64
6H	SCRI_RS_194023	2.64
6H	SCRI_RS_153928	2.8
6H	SCRI_RS_194048	2.99
6H	11_10669	3.33
6H	SCRI_RS_8388	3.33
6H	11_20882	4.06
6H	SCRI_RS_159612	4.06
6H	SCRI_RS_56	4.2
6H	SCRI_RS_123172	4.34
6H	SCRI_RS_138977	4.34
6H	SCRI_RS_141842	4.34
6H	SCRI_RS_146246	4.34
6H	SCRI_RS_203036	4.34
6H	SCRI_RS_207933	4.34
6H	SCRI_RS_211856	4.34
6H	SCRI_RS_211920	4.34
6H	SCRI_RS_153023	4.4
6H	11_10120	5.72
6H	11_11406	5.72
6H	SCRI_RS_1429	6.25
6H	SCRI_RS_194036	6.25
6H	SCRI_RS_202485	6.25
6H	SCRI_RS_206183	6.25
6H	11_20294	6.4
6H	SCRI_RS_89111	6.4
6H	12_30651	7
6H	11_21204	7.8
6H	11_20262	9
6H	11_21032	9.96
6H	SCRI_RS_231003	10.3
6H	SCRI_RS_159124	10.5
6H	SCRI_RS_146663	13.28
6H	SCRI_RS_164308	13.28
6H	SCRI_RS_229916	13.28
6H	11_11479	13.44
6H	11_20415	14.94
6H	SCRI_RS_139713	15.38
6H	SCRI_RS_139715	15.38
6H	SCRI_RS_202723	15.78
6H	SCRI_RS_20187	15.88
6H	12_30010	16.14
6H	SCRI_RS_190075	16.9
6H	SCRI_RS_154574	16.97
6H	11_10165	17.87
6H	12_30842	17.87
6H	SCRI_RS_106920	17.87
6H	SCRI_RS_62666	17.87
6H	11_10064	20.87
6H	12_10554	20.87
6H	SCRI_RS_139519	21.61
6H	SCRI_RS_186897	21.61
6H	SCRI_RS_190870	21.61
6H	SCRI_RS_207376	21.61
6H	12_30843	21.81
6H	11_10023	21.81
6H	SCRI_RS_129888	21.81
6H	11_21246	22.78
6H	SCRI_RS_183580	24.21
6H	11_20315	24.23
6H	11_10868	24.23
6H	11_10136	24.28
6H	SCRI_RS_139117	24.66
6H	SCRI_RS_189122	24.66
6H	SCRI_RS_154426	25.5
6H	SCRI_RS_231372	25.5
6H	SCRI_RS_138556	25.65
6H	SCRI_RS_201251	25.65
6H	SCRI_RS_189878	28.28
6H	11_10676	28.95
6H	SCRI_RS_222092	29.54
6H	12_30697	29.78
6H	12_30603	29.87
6H	12_30673	29.88
6H	12_31485	29.88
6H	12_31308	29.98
6H	11_20745	30.88
6H	11_10994	31.98
6H	SCRI_RS_21695	32.38
6H	11_10799	32.98
6H	11_10939	33.55
6H	11_10427	33.55
6H	12_30358	34.22
6H	SCRI_RS_147090	35.38
6H	SCRI_RS_186520	35.78
6H	SCRI_RS_219710	36.62
6H	SCRI_RS_157552	36.78
6H	12_30521	37.11
6H	SCRI_RS_235711	37.78
6H	SCRI_RS_154121	37.88
6H	12_30361	38.94
6H	11_10061	40.52
6H	11_10129	40.52
6H	11_10494	40.52
6H	11_10882	40.52
6H	11_20052	40.52
6H	11_21030	40.52
6H	12_11455	40.52
6H	12_30665	40.52
6H	12_30783	40.52
6H	11_20936	42
6H	12_30516	42
6H	11_21281	42.66
6H	11_10244	43.98
6H	11_20743	43.98
6H	11_10355	44.73
6H	11_10462	44.73
6H	SCRI_RS_172746	44.77
6H	SCRI_RS_125560	45.05
6H	SCRI_RS_14820	45.05
6H	11_10013	45.23
6H	11_10817	45.23
6H	11_11097	45.23
6H	11_20707	45.23
6H	12_10199	45.23
6H	12_10575	45.23
6H	12_30317	45.23
6H	11_10539	45.89
6H	SCRI_RS_139451	46.96
6H	11_10461	47.75
6H	12_10910	47.75
6H	12_30133	47.75
6H	12_30316	47.75
6H	12_30658	47.75
6H	11_20291	48.41
6H	12_30510	48.41
6H	12_30751	48.41
6H	12_31092	48.41
6H	12_31274	48.41
6H	12_31433	48.41
6H	SCRI_RS_161627	48.49
6H	SCRI_RS_147692	48.54
6H	SCRI_RS_157378	48.54
6H	SCRI_RS_162771	48.54
6H	SCRI_RS_165352	48.54
6H	SCRI_RS_196285	48.54
6H	SCRI_RS_7397	48.54
6H	SCRI_RS_102426	48.64
6H	11_11205	49.1
6H	11_20675	49.1
6H	12_11171	49.1
6H	12_30782	49.1
6H	SCRI_RS_114351	49.29
6H	SCRI_RS_141429	49.29
6H	SCRI_RS_219061	49.29
6H	SCRI_RS_115036	49.47
6H	SCRI_RS_121709	49.47
6H	SCRI_RS_150028	49.47
6H	SCRI_RS_213547	49.96
6H	SCRI_RS_168118	49.96
6H	SCRI_RS_170814	49.96
6H	SCRI_RS_2534	49.96
6H	12_21449	50.07
6H	12_30569	50.81
6H	SCRI_RS_156616	51.33
6H	SCRI_RS_143259	51.33
6H	SCRI_RS_146865	51.33
6H	SCRI_RS_162581	51.4
6H	SCRI_RS_238639	51.4
6H	12_31528	51.4
6H	SCRI_RS_155654	51.4
6H	SCRI_RS_171247	51.4
6H	SCRI_RS_228181	51.47
6H	SCRI_RS_142506	51.47
6H	SCRI_RS_151282	51.47
6H	SCRI_RS_182004	51.47
6H	SCRI_RS_210025	51.47
6H	SCRI_RS_128460	51.47
6H	SCRI_RS_147342	51.47
6H	SCRI_RS_120783	51.47
6H	SCRI_RS_133544	51.47
6H	SCRI_RS_146235	51.47
6H	SCRI_RS_176	51.47
6H	SCRI_RS_188949	51.47
6H	SCRI_RS_192720	51.47
6H	SCRI_RS_196459	51.47
6H	SCRI_RS_236452	51.47
6H	SCRI_RS_169829	51.47
6H	SCRI_RS_119674	51.54
6H	SCRI_RS_121633	51.54
6H	SCRI_RS_182367	51.54
6H	SCRI_RS_196458	51.54
6H	SCRI_RS_200882	51.54
6H	SCRI_RS_238855	51.54
6H	SCRI_RS_239962	51.54
6H	12_30186	51.54
6H	12_30353	51.54
6H	SCRI_RS_121489	51.54
6H	SCRI_RS_176663	51.54
6H	SCRI_RS_211830	51.54
6H	SCRI_RS_219962	51.54
6H	11_10003	51.56
6H	12_10497	51.56
6H	12_11104	51.56
6H	12_11353	51.56
6H	12_20381	51.56
6H	12_30032	51.56
6H	12_30120	51.56
6H	12_30430	51.56
6H	12_30473	51.56
6H	SCRI_RS_205857	51.93
6H	SCRI_RS_209824	51.93
6H	SCRI_RS_168111	51.99
6H	SCRI_RS_157299	52.1
6H	SCRI_RS_168455	52.1
6H	SCRI_RS_234724	52.28
6H	SCRI_RS_142352	52.28
6H	11_21473	52.68
6H	11_20720	52.68
6H	12_30441	52.73
6H	12_31007	52.73
6H	12_31005	52.73
6H	11_21158	52.73
6H	SCRI_RS_140158	52.74
6H	SCRI_RS_152174	52.74
6H	SCRI_RS_187497	52.74
6H	SCRI_RS_211299	52.74
6H	SCRI_RS_146096	53.06
6H	SCRI_RS_200067	53.06
6H	SCRI_RS_239889	53.06
6H	12_30802	53.08
6H	11_10962	53.38
6H	11_21014	53.38
6H	12_30021	53.38
6H	11_20656	53.38
6H	SCRI_RS_232893	53.48
6H	SCRI_RS_9980	53.48
6H	SCRI_RS_167853	53.48
6H	11_20651	53.58
6H	11_10323	53.58
6H	11_10659	53.58
6H	11_10749	53.58
6H	11_10848	53.58
6H	11_20329	53.58
6H	11_20567	53.58
6H	11_20572	53.58
6H	11_20600	53.58
6H	11_20785	53.58
6H	11_20799	53.58
6H	11_20854	53.58
6H	11_20946	53.58
6H	11_21216	53.58
6H	12_10591	53.58
6H	12_11442	53.58
6H	12_11487	53.58
6H	12_20142	53.58
6H	12_21482	53.58
6H	12_30230	53.58
6H	12_30508	53.58
6H	12_30567	53.58
6H	12_30765	53.58
6H	12_30837	53.58
6H	12_31249	53.58
6H	12_31509	53.58
6H	11_10954	54.16
6H	11_11312	54.16
6H	11_21124	54.16
6H	11_11153	54.18
6H	12_11140	54.18
6H	12_30305	54.18
6H	SCRI_RS_204495	54.54
6H	12_30311	54.68
6H	12_30465	54.68
6H	12_30511	54.68
6H	12_30596	54.68
6H	12_30749	54.68
6H	12_30856	54.68
6H	12_31006	54.68
6H	SCRI_RS_189254	54.79
6H	11_10227	54.92
6H	11_20835	54.92
6H	11_10377	54.92
6H	11_10513	54.92
6H	12_10278	54.92
6H	12_10345	54.92
6H	12_21114	54.92
6H	12_30681	54.92
6H	12_31174	54.92
6H	12_20463	54.96
6H	12_30395	54.96
6H	12_31178	54.96
6H	SCRI_RS_116842	54.97
6H	SCRI_RS_125353	55.18
6H	SCRI_RS_156115	55.18
6H	SCRI_RS_164602	55.18
6H	SCRI_RS_176150	55.18
6H	SCRI_RS_189606	55.18
6H	SCRI_RS_206986	55.18
6H	SCRI_RS_210918	55.18
6H	SCRI_RS_135049	55.18
6H	SCRI_RS_166120	55.18
6H	SCRI_RS_205103	55.18
6H	SCRI_RS_236999	55.18
6H	SCRI_RS_131647	55.25
6H	SCRI_RS_148432	55.25
6H	SCRI_RS_124850	55.25
6H	SCRI_RS_229687	55.25
6H	SCRI_RS_153619	55.25
6H	SCRI_RS_158873	55.25
6H	SCRI_RS_141134	55.25
6H	SCRI_RS_146801	55.25
6H	SCRI_RS_237465	55.25
6H	SCRI_RS_180937	55.25
6H	SCRI_RS_114741	55.25
6H	SCRI_RS_127556	55.25
6H	SCRI_RS_130732	55.25
6H	SCRI_RS_170058	55.25
6H	SCRI_RS_188520	55.25
6H	SCRI_RS_129048	55.25
6H	SCRI_RS_130605	55.25
6H	SCRI_RS_136897	55.25
6H	SCRI_RS_150096	55.25
6H	SCRI_RS_159136	55.25
6H	SCRI_RS_161167	55.25
6H	SCRI_RS_161288	55.25
6H	SCRI_RS_169728	55.25
6H	SCRI_RS_176086	55.25
6H	SCRI_RS_182195	55.25
6H	SCRI_RS_187832	55.25
6H	SCRI_RS_188523	55.25
6H	SCRI_RS_195226	55.25
6H	SCRI_RS_196373	55.25
6H	SCRI_RS_221874	55.25
6H	SCRI_RS_208339	55.25
6H	SCRI_RS_187	55.25
6H	SCRI_RS_126925	55.28
6H	SCRI_RS_182515	55.37
6H	SCRI_RS_131341	55.43
6H	SCRI_RS_2834	55.43
6H	SCRI_RS_118381	55.43
6H	SCRI_RS_121418	55.43
6H	SCRI_RS_128181	55.43
6H	SCRI_RS_131870	55.43
6H	SCRI_RS_136658	55.43
6H	SCRI_RS_179537	55.43
6H	SCRI_RS_193776	55.43
6H	SCRI_RS_200419	55.43
6H	SCRI_RS_205256	55.43
6H	SCRI_RS_215773	55.43
6H	SCRI_RS_219810	55.43
6H	SCRI_RS_236959	55.43
6H	SCRI_RS_11588	55.43
6H	SCRI_RS_193129	55.43
6H	SCRI_RS_224430	55.43
6H	12_31479	55.43
6H	BK_09	55.43
6H	SCRI_RS_123295	55.43
6H	SCRI_RS_143104	55.43
6H	SCRI_RS_236545	55.43
6H	12_11039	55.43
6H	SCRI_RS_136678	55.43
6H	SCRI_RS_179278	55.43
6H	SCRI_RS_225532	55.43
6H	12_30857	55.46
6H	11_20184	55.46
6H	12_30144	55.46
6H	SCRI_RS_10964	56.46
6H	SCRI_RS_80327	56.46
6H	SCRI_RS_138529	56.46
6H	SCRI_RS_143415	56.46
6H	SCRI_RS_146867	56.46
6H	SCRI_RS_163053	56.46
6H	SCRI_RS_239642	56.46
6H	SCRI_RS_16796	56.46
6H	SCRI_RS_16817	56.46
6H	SCRI_RS_198506	56.46
6H	SCRI_RS_222017	56.46
6H	SCRI_RS_239917	56.46
6H	SCRI_RS_10685	56.46
6H	SCRI_RS_108698	56.46
6H	SCRI_RS_124776	56.46
6H	SCRI_RS_126229	56.46
6H	SCRI_RS_142767	56.46
6H	SCRI_RS_144344	56.46
6H	SCRI_RS_149539	56.46
6H	SCRI_RS_162961	56.46
6H	SCRI_RS_186147	56.46
6H	SCRI_RS_224389	56.46
6H	SCRI_RS_233266	56.46
6H	SCRI_RS_237750	56.46
6H	SCRI_RS_7967	56.46
6H	SCRI_RS_111556	56.46
6H	SCRI_RS_111820	56.46
6H	SCRI_RS_118255	56.46
6H	SCRI_RS_122124	56.46
6H	SCRI_RS_138001	56.46
6H	SCRI_RS_138253	56.46
6H	SCRI_RS_13935	56.46
6H	SCRI_RS_142727	56.46
6H	SCRI_RS_144098	56.46
6H	SCRI_RS_144162	56.46
6H	SCRI_RS_144579	56.46
6H	SCRI_RS_148652	56.46
6H	SCRI_RS_153502	56.46
6H	SCRI_RS_153797	56.46
6H	SCRI_RS_160798	56.46
6H	SCRI_RS_161533	56.46
6H	SCRI_RS_162504	56.46
6H	SCRI_RS_162760	56.46
6H	SCRI_RS_175593	56.46
6H	SCRI_RS_187343	56.46
6H	SCRI_RS_187725	56.46
6H	SCRI_RS_192768	56.46
6H	SCRI_RS_196518	56.46
6H	SCRI_RS_206213	56.46
6H	SCRI_RS_207083	56.46
6H	SCRI_RS_207174	56.46
6H	SCRI_RS_223224	56.46
6H	SCRI_RS_224695	56.46
6H	SCRI_RS_34440	56.46
6H	SCRI_RS_7104	56.46
6H	SCRI_RS_72672	56.46
6H	SCRI_RS_114613	56.46
6H	SCRI_RS_137706	56.46
6H	SCRI_RS_140091	56.46
6H	SCRI_RS_146992	56.46
6H	SCRI_RS_147666	56.46
6H	SCRI_RS_153707	56.46
6H	SCRI_RS_174583	56.46
6H	SCRI_RS_176650	56.46
6H	SCRI_RS_179476	56.46
6H	SCRI_RS_182180	56.46
6H	SCRI_RS_186193	56.46
6H	SCRI_RS_187697	56.46
6H	SCRI_RS_188243	56.46
6H	SCRI_RS_192599	56.46
6H	SCRI_RS_222319	56.46
6H	SCRI_RS_222944	56.46
6H	SCRI_RS_235731	56.46
6H	SCRI_RS_142541	56.46
6H	SCRI_RS_213566	56.46
6H	SCRI_RS_137464	56.46
6H	SCRI_RS_171997	56.46
6H	SCRI_RS_237307	56.46
6H	SCRI_RS_141113	56.46
6H	SCRI_RS_154198	56.46
6H	SCRI_RS_188375	56.46
6H	SCRI_RS_220726	56.46
6H	SCRI_RS_165041	56.46
6H	SCRI_RS_188305	56.46
6H	SCRI_RS_190875	56.46
6H	SCRI_RS_209827	56.46
6H	SCRI_RS_125526	56.46
6H	SCRI_RS_125529	56.46
6H	SCRI_RS_237887	56.46
6H	12_30858	56.46
6H	12_30859	56.46
6H	SCRI_RS_136604	56.46
6H	SCRI_RS_166125	56.46
6H	SCRI_RS_168096	56.46
6H	SCRI_RS_195914	56.46
6H	SCRI_RS_206796	56.46
6H	SCRI_RS_168964	56.46
6H	SCRI_RS_136136	56.46
6H	SCRI_RS_203997	56.46
6H	12_30254	56.49
6H	SCRI_RS_115369	56.49
6H	SCRI_RS_184086	56.49
6H	SCRI_RS_201811	56.53
6H	SCRI_RS_206159	56.53
6H	11_11067	56.74
6H	12_11181	56.74
6H	12_11253	56.74
6H	12_10803	57.74
6H	SCRI_RS_10810	58.66
6H	SCRI_RS_134034	58.66
6H	SCRI_RS_134469	58.66
6H	SCRI_RS_165585	58.66
6H	SCRI_RS_170846	58.66
6H	SCRI_RS_192489	58.66
6H	11_10270	58.68
6H	12_31003	58.68
6H	SCRI_RS_206704	58.76
6H	SCRI_RS_129321	59.1
6H	SCRI_RS_158011	59.1
6H	SCRI_RS_170672	59.1
6H	SCRI_RS_173641	59.1
6H	SCRI_RS_182016	59.1
6H	SCRI_RS_184246	59.1
6H	SCRI_RS_237419	59.1
6H	SCRI_RS_222315	59.1
6H	11_10964	59.32
6H	11_20266	59.32
6H	11_21339	59.32
6H	SCRI_RS_169672	59.36
6H	12_30804	59.46
6H	11_10189	59.86
6H	SCRI_RS_133367	59.86
6H	SCRI_RS_137824	59.86
6H	SCRI_RS_140640	59.86
6H	SCRI_RS_148543	59.86
6H	SCRI_RS_150504	59.86
6H	SCRI_RS_167	59.86
6H	SCRI_RS_206731	59.86
6H	SCRI_RS_209993	59.86
6H	SCRI_RS_211289	59.86
6H	SCRI_RS_217259	59.86
6H	SCRI_RS_232544	59.86
6H	SCRI_RS_237661	59.86
6H	SCRI_RS_7251	59.86
6H	11_20058	59.86
6H	11_21310	59.86
6H	11_10635	59.86
6H	12_10758	59.86
6H	12_11321	59.86
6H	12_30346	59.86
6H	12_30844	59.86
6H	SCRI_RS_197287	60.17
6H	SCRI_RS_204148	60.26
6H	12_11386	60.27
6H	SCRI_RS_226330	61.58
6H	SCRI_RS_4697	61.58
6H	SCRI_RS_106581	61.73
6H	SCRI_RS_143492	61.73
6H	SCRI_RS_154772	61.73
6H	SCRI_RS_187114	61.73
6H	SCRI_RS_6224	61.73
6H	12_31359	61.87
6H	12_31444	61.87
6H	SCRI_RS_224297	61.87
6H	SCRI_RS_226342	61.87
6H	11_11483	61.96
6H	12_11475	61.96
6H	11_21298	62
6H	SCRI_RS_175000	62.01
6H	SCRI_RS_9180	62.07
6H	SCRI_RS_221698	62.61
6H	SCRI_RS_8681	62.61
6H	11_21225	63.16
6H	11_21293	63.16
6H	12_30637	63.16
6H	11_21069	63.16
6H	12_10392	63.49
6H	11_20904	63.63
6H	11_10781	63.67
6H	11_11261	63.84
6H	SCRI_RS_152841	64.64
6H	SCRI_RS_140123	64.74
6H	SCRI_RS_149556	64.74
6H	SCRI_RS_152393	64.74
6H	SCRI_RS_165986	64.74
6H	SCRI_RS_168065	64.74
6H	SCRI_RS_182648	64.74
6H	SCRI_RS_236457	64.74
6H	12_20148	64.79
6H	11_10455	64.86
6H	SCRI_RS_154805	64.96
6H	SCRI_RS_206536	65.16
6H	SCRI_RS_209210	65.16
6H	11_20287	65.16
6H	SCRI_RS_205595	65.27
6H	11_10040	65.6
6H	11_20709	65.6
6H	SCRI_RS_115440	65.62
6H	SCRI_RS_224292	65.62
6H	SCRI_RS_204596	65.66
6H	11_10124	65.66
6H	12_10504	65.66
6H	11_20015	65.66
6H	11_20714	65.84
6H	11_21469	66.1
6H	SCRI_RS_116605	66.26
6H	SCRI_RS_152800	66.26
6H	11_20468	66.3
6H	11_20636	67.1
6H	SCRI_RS_143367	67.4
6H	SCRI_RS_225072	67.4
6H	12_10596	67.4
6H	SCRI_RS_9620	67.4
6H	11_20673	67.4
6H	12_31289	67.4
6H	SCRI_RS_139937	67.67
6H	SCRI_RS_219632	67.67
6H	SCRI_RS_136724	67.7
6H	11_20620	67.7
6H	SCRI_RS_133948	67.71
6H	SCRI_RS_231205	67.74
6H	SCRI_RS_3016	67.74
6H	SCRI_RS_5787	67.74
6H	SCRI_RS_140155	67.88
6H	12_10348	67.89
6H	SCRI_RS_236779	67.89
6H	11_11329	68.05
6H	11_20892	68.05
6H	SCRI_RS_126705	68.22
6H	SCRI_RS_124856	68.36
6H	12_31250	68.6
6H	11_11349	68.78
6H	11_20577	68.78
6H	11_20784	68.78
6H	SCRI_RS_165594	68.8
6H	SCRI_RS_175370	69.09
6H	SCRI_RS_204363	69.09
6H	SCRI_RS_6697	69.09
6H	SCRI_RS_157316	69.44
6H	11_11459	69.58
6H	11_20744	69.58
6H	11_21256	69.58
6H	12_31101	69.58
6H	SCRI_RS_225193	69.74
6H	11_20053	70.51
6H	12_10393	70.51
6H	SCRI_RS_137215	70.51
6H	SCRI_RS_153166	70.51
6H	SCRI_RS_166239	70.51
6H	SCRI_RS_205971	70.51
6H	SCRI_RS_206976	70.51
6H	SCRI_RS_235954	70.51
6H	SCRI_RS_4653	70.51
6H	11_10469	70.51
6H	SCRI_RS_231790	71.2
6H	SCRI_RS_152224	71.2
6H	SCRI_RS_167655	71.2
6H	SCRI_RS_164341	71.2
6H	SCRI_RS_172605	71.2
6H	SCRI_RS_187506	71.2
6H	SCRI_RS_221951	71.2
6H	SCRI_RS_228493	71.2
6H	12_30908	71.55
6H	12_30939	71.55
6H	12_30236	71.82
6H	12_31111	71.82
6H	SCRI_RS_139297	71.9
6H	SCRI_RS_238393	71.9
6H	SCRI_RS_145279	71.9
6H	12_31088	72.35
6H	11_20488	73.01
6H	11_20682	73.01
6H	11_20969	73.01
6H	11_20336	73.36
6H	SCRI_RS_16773	73.36
6H	SCRI_RS_8401	73.36
6H	SCRI_RS_129756	73.59
6H	SCRI_RS_137870	73.59
6H	SCRI_RS_13815	73.59
6H	SCRI_RS_143317	73.59
6H	SCRI_RS_144337	73.59
6H	SCRI_RS_144892	73.59
6H	SCRI_RS_152324	73.59
6H	SCRI_RS_207284	73.59
6H	SCRI_RS_193336	73.7
6H	SCRI_RS_220821	74.14
6H	11_20746	74.48
6H	SCRI_RS_12874	74.63
6H	SCRI_RS_144862	74.63
6H	SCRI_RS_154582	74.63
6H	SCRI_RS_217187	74.63
6H	SCRI_RS_14134	74.73
6H	SCRI_RS_100903	74.88
6H	SCRI_RS_136848	75.02
6H	SCRI_RS_153227	75.02
6H	SCRI_RS_153435	75.02
6H	11_21224	75.5
6H	SCRI_RS_80	76.24
6H	SCRI_RS_171358	76.38
6H	11_10608	76.79
6H	SCRI_RS_133	76.94
6H	SCRI_RS_150951	76.94
6H	SCRI_RS_171096	76.94
6H	SCRI_RS_173878	76.94
6H	SCRI_RS_81903	76.94
6H	SCRI_RS_142393	77.37
6H	11_10220	77.42
6H	11_11458	77.42
6H	12_30573	77.42
6H	12_30940	77.42
6H	12_31253	77.42
6H	SCRI_RS_170674	77.6
6H	SCRI_RS_126972	77.97
6H	12_30148	78.12
6H	SCRI_RS_1937	79.5
6H	SCRI_RS_131992	79.63
6H	SCRI_RS_181031	79.63
6H	SCRI_RS_184811	79.63
6H	SCRI_RS_140037	79.78
6H	SCRI_RS_165945	80.57
6H	11_10331	81.23
6H	SCRI_RS_736	81.9
6H	11_10185	81.98
6H	11_11246	81.98
6H	11_20465	81.98
6H	BK_02	82.12
6H	SCRI_RS_177093	82.4
6H	SCRI_RS_200064	82.4
6H	SCRI_RS_4707	82.4
6H	SCRI_RS_167497	82.52
6H	11_20346	82.72
6H	11_21404	82.72
6H	11_11147	83.3
6H	11_20654	83.3
6H	11_10815	84.6
6H	SCRI_RS_10932	84.99
6H	SCRI_RS_160175	84.99
6H	SCRI_RS_164037	84.99
6H	SCRI_RS_169374	84.99
6H	SCRI_RS_184453	84.99
6H	SCRI_RS_8702	84.99
6H	SCRI_RS_149165	85.18
6H	SCRI_RS_165322	85.2
6H	SCRI_RS_159441	85.2
6H	12_30698	86.28
6H	SCRI_RS_102418	86.37
6H	SCRI_RS_167845	86.37
6H	SCRI_RS_187841	87.09
6H	11_10400	88.28
6H	11_20783	88.28
6H	SCRI_RS_202478	88.81
6H	11_21025	89.64
6H	SCRI_RS_161652	89.84
6H	SCRI_RS_200907	89.84
6H	SCRI_RS_182637	90.02
6H	11_10202	90.22
6H	SCRI_RS_164156	90.26
6H	SCRI_RS_175709	90.76
6H	12_30626	90.92
6H	12_31235	90.92
6H	SCRI_RS_8252	90.97
6H	11_10496	91.32
6H	SCRI_RS_138716	91.32
6H	SCRI_RS_147599	91.32
6H	SCRI_RS_149401	91.32
6H	SCRI_RS_183055	91.32
6H	SCRI_RS_129236	91.61
6H	SCRI_RS_147062	91.61
6H	SCRI_RS_131929	91.61
6H	SCRI_RS_155564	91.61
6H	SCRI_RS_161211	91.61
6H	SCRI_RS_162589	91.61
6H	SCRI_RS_194066	91.61
6H	SCRI_RS_7640	91.61
6H	SCRI_RS_173232	91.61
6H	SCRI_RS_190647	91.61
6H	11_20996	92.1
6H	11_11294	92.65
6H	11_20728	92.65
6H	SCRI_RS_222802	93.2
6H	SCRI_RS_120442	93.28
6H	SCRI_RS_124224	93.28
6H	SCRI_RS_131977	93.28
6H	SCRI_RS_95857	93.28
6H	SCRI_RS_98225	93.28
6H	SCRI_RS_175469	93.48
6H	SCRI_RS_176230	93.48
6H	11_10978	93.75
6H	11_20118	93.75
6H	SCRI_RS_158871	93.76
6H	SCRI_RS_159133	93.95
6H	SCRI_RS_173142	93.95
6H	SCRI_RS_224910	94.4
6H	11_20972	94.42
6H	11_10595	95.09
6H	12_31432	95.09
6H	SCRI_RS_139987	96.25
6H	SCRI_RS_120404	96.31
6H	SCRI_RS_168578	96.31
6H	SCRI_RS_191173	96.31
6H	11_10734	96.65
6H	SCRI_RS_239959	96.81
6H	SCRI_RS_201988	96.83
6H	SCRI_RS_218152	96.83
6H	SCRI_RS_152675	96.88
6H	SCRI_RS_188103	96.88
6H	SCRI_RS_194345	96.88
6H	SCRI_RS_235665	96.88
6H	11_10139	97.31
6H	SCRI_RS_132023	97.31
6H	SCRI_RS_144448	97.94
6H	11_10015	98.68
6H	11_20531	98.68
6H	12_30151	98.68
6H	12_31353	98.68
6H	12_11494	98.68
6H	12_31042	99.33
6H	12_31043	99.33
6H	12_31044	99.33
6H	12_31048	99.33
6H	12_31115	99.33
6H	SCRI_RS_151574	99.36
6H	SCRI_RS_214350	99.36
6H	11_20342	100.29
6H	SCRI_RS_128218	100.8
6H	SCRI_RS_131404	101.95
6H	SCRI_RS_15771	101.95
6H	SCRI_RS_234362	101.98
6H	11_20292	102.25
6H	11_20379	102.25
6H	SCRI_RS_11212	103.75
6H	SCRI_RS_223732	103.75
6H	12_10704	104.45
6H	11_20036	105.09
6H	11_20725	105.09
6H	11_21271	105.09
6H	SCRI_RS_202438	105.24
6H	SCRI_RS_43891	105.24
6H	SCRI_RS_8034	105.24
6H	11_20467	105.24
6H	SCRI_RS_168121	105.45
6H	SCRI_RS_189619	105.45
6H	SCRI_RS_3070	105.45
6H	SCRI_RS_6720	105.45
6H	SCRI_RS_161117	105.45
6H	SCRI_RS_189611	105.45
6H	SCRI_RS_159084	105.59
6H	12_30734	106.5
6H	11_20355	107.63
6H	SCRI_RS_144541	107.64
6H	SCRI_RS_147455	107.64
6H	SCRI_RS_149487	107.64
6H	SCRI_RS_162836	107.64
6H	SCRI_RS_206207	107.64
6H	SCRI_RS_240029	107.64
6H	SCRI_RS_2530	107.64
6H	SCRI_RS_131119	107.8
6H	12_20448	108.38
6H	11_20558	109.08
6H	SCRI_RS_204188	109.08
6H	SCRI_RS_213956	109.08
6H	SCRI_RS_236953	109.08
6H	11_10239	109.08
6H	12_31495	109.08
6H	11_11534	109.08
6H	SCRI_RS_151263	109.42
6H	SCRI_RS_135063	109.7
6H	SCRI_RS_169022	109.7
6H	SCRI_RS_176076	109.7
6H	SCRI_RS_4971	109.7
6H	SCRI_RS_233106	109.77
6H	SCRI_RS_182275	109.91
6H	SCRI_RS_626	109.91
6H	SCRI_RS_149269	110.12
6H	12_21477	112.33
6H	12_30025	113.35
6H	11_10107	113.35
6H	11_21467	113.35
6H	11_10645	114.13
6H	11_10175	114.78
6H	11_21455	114.78
6H	12_10051	114.78
6H	12_10071	114.78
6H	12_30355	114.78
6H	12_31198	115.05
6H	SCRI_RS_143018	115.05
6H	11_20005	115.9
6H	11_11187	115.9
6H	12_31126	115.9
6H	SCRI_RS_157323	116.21
6H	SCRI_RS_162678	116.21
6H	SCRI_RS_234356	116.35
6H	SCRI_RS_161343	116.68
6H	SCRI_RS_234548	116.68
6H	SCRI_RS_347	116.68
6H	SCRI_RS_124549	117.15
6H	SCRI_RS_151280	117.5
6H	11_20029	117.65
6H	SCRI_RS_102455	117.81
6H	12_31053	117.84
6H	SCRI_RS_111434	117.84
6H	SCRI_RS_164877	117.84
6H	SCRI_RS_188314	117.84
6H	SCRI_RS_138887	118.23
6H	11_10748	118.3
6H	11_11488	118.3
6H	11_20211	118.3
6H	11_20817	118.3
6H	11_20868	119.1
6H	12_20907	119.98
6H	SCRI_RS_171887	120.53
6H	SCRI_RS_138295	120.58
6H	SCRI_RS_138188	120.61
6H	SCRI_RS_177168	120.61
6H	SCRI_RS_201159	120.61
6H	SCRI_RS_226875	120.61
6H	SCRI_RS_6399	120.61
6H	SCRI_RS_99836	120.61
6H	SCRI_RS_126069	120.67
6H	SCRI_RS_179580	120.67
6H	SCRI_RS_205578	120.67
6H	SCRI_RS_206827	120.67
6H	SCRI_RS_77293	120.67
6H	12_31498	121.06
6H	SCRI_RS_17542	121.62
6H	SCRI_RS_235672	121.62
6H	11_10390	121.81
6H	11_10828	121.81
6H	12_31283	121.81
6H	SCRI_RS_238352	122.15
6H	SCRI_RS_203457	122.15
6H	SCRI_RS_10655	122.59
6H	SCRI_RS_174459	122.59
6H	SCRI_RS_175610	122.59
6H	SCRI_RS_181159	122.59
6H	SCRI_RS_225737	122.59
6H	SCRI_RS_9648	122.59
6H	SCRI_RS_159529	122.59
6H	SCRI_RS_204644	122.59
6H	11_21291	122.59
6H	SCRI_RS_159236	122.59
6H	SCRI_RS_152414	122.66
6H	SCRI_RS_232698	123.01
6H	SCRI_RS_193443	123.16
6H	11_20687	123.49
6H	SCRI_RS_143994	123.55
6H	SCRI_RS_200962	123.55
6H	11_20537	126.5
6H	SCRI_RS_223104	126.5
6H	11_11111	126.5
6H	SCRI_RS_229960	126.5
6H	SCRI_RS_10811	126.5
6H	11_21112	126.5
6H	12_30956	126.6
6H	SCRI_RS_144034	126.6
6H	12_30627	128.95
7H	11_21419	0
7H	12_31075	0
7H	SCRI_RS_154356	0.26
7H	11_10949	0.26
7H	11_10971	0.26
7H	11_21395	0.26
7H	SCRI_RS_101902	0.26
7H	SCRI_RS_101976	0.26
7H	SCRI_RS_156125	0.26
7H	SCRI_RS_161101	0.26
7H	SCRI_RS_166511	0.26
7H	SCRI_RS_186177	0.26
7H	SCRI_RS_189396	0.26
7H	SCRI_RS_190844	0.26
7H	SCRI_RS_193293	0.26
7H	SCRI_RS_207095	0.26
7H	SCRI_RS_208186	0.26
7H	SCRI_RS_235318	0.26
7H	SCRI_RS_2844	0.26
7H	12_10036	0.26
7H	SCRI_RS_138496	0.26
7H	SCRI_RS_14172	0.26
7H	SCRI_RS_200389	0.26
7H	SCRI_RS_200895	0.26
7H	SCRI_RS_201258	0.26
7H	SCRI_RS_205803	0.26
7H	SCRI_RS_92998	0.26
7H	11_20303	0.26
7H	12_30296	0.26
7H	12_30959	0.26
7H	12_31076	0.26
7H	SCRI_RS_235587	0.26
7H	SCRI_RS_6865	0.26
7H	SCRI_RS_190585	0.26
7H	SCRI_RS_82982	0.26
7H	SCRI_RS_217123	0.26
7H	SCRI_RS_186199	0.26
7H	SCRI_RS_8079	0.26
7H	12_20016	0.4
7H	12_31072	0.4
7H	SCRI_RS_229445	0.4
7H	SCRI_RS_140736	0.4
7H	SCRI_RS_148107	0.4
7H	SCRI_RS_200232	0.4
7H	SCRI_RS_114207	0.44
7H	SCRI_RS_177315	0.44
7H	SCRI_RS_201028	0.44
7H	SCRI_RS_93773	0.44
7H	11_21307	0.44
7H	SCRI_RS_621	0.44
7H	SCRI_RS_237689	0.44
7H	11_21443	0.51
7H	11_10682	0.51
7H	12_30472	0.51
7H	11_10121	0.54
7H	11_10894	0.54
7H	SCRI_RS_166230	0.54
7H	12_20839	0.68
7H	SCRI_RS_121519	0.68
7H	SCRI_RS_194557	0.68
7H	SCRI_RS_226361	0.68
7H	SCRI_RS_187033	0.76
7H	12_20201	0.76
7H	SCRI_RS_172655	0.84
7H	11_20710	0.84
7H	SCRI_RS_127385	0.84
7H	12_31350	1.04
7H	SCRI_RS_160297	1.04
7H	SCRI_RS_13615	1.44
7H	11_20534	2.11
7H	SCRI_RS_178911	2.7
7H	11_20242	2.78
7H	11_11222	2.78
7H	SCRI_RS_157337	2.78
7H	SCRI_RS_230959	2.78
7H	11_11031	3.04
7H	SCRI_RS_132017	3.04
7H	SCRI_RS_137983	3.04
7H	SCRI_RS_42792	3.04
7H	12_31173	3.04
7H	12_31411	3.04
7H	SCRI_RS_175756	3.04
7H	SCRI_RS_230060	3.04
7H	SCRI_RS_230149	3.04
7H	SCRI_RS_140481	4.67
7H	SCRI_RS_140250	4.67
7H	SCRI_RS_129323	4.67
7H	SCRI_RS_146728	4.67
7H	SCRI_RS_12396	4.99
7H	SCRI_RS_131225	4.99
7H	SCRI_RS_139563	4.99
7H	SCRI_RS_156237	4.99
7H	SCRI_RS_158266	4.99
7H	SCRI_RS_159196	4.99
7H	SCRI_RS_190541	4.99
7H	SCRI_RS_235976	4.99
7H	SCRI_RS_333	4.99
7H	12_11035	4.99
7H	11_11495	5.03
7H	12_10406	5.57
7H	SCRI_RS_146349	5.65
7H	SCRI_RS_147533	5.65
7H	SCRI_RS_155813	5.65
7H	SCRI_RS_170908	5.65
7H	SCRI_RS_186187	5.65
7H	SCRI_RS_45657	5.65
7H	11_20245	6.11
7H	12_31450	6.11
7H	12_20227	6.69
7H	SCRI_RS_156158	7.96
7H	11_10841	7.97
7H	11_10851	7.97
7H	11_20225	7.97
7H	12_31285	7.97
7H	11_20755	8.81
7H	11_21437	9.89
7H	SCRI_RS_111979	9.94
7H	SCRI_RS_166323	9.94
7H	11_20014	10.11
7H	SCRI_RS_152860	10.43
7H	SCRI_RS_186943	10.43
7H	SCRI_RS_152931	10.44
7H	SCRI_RS_167386	10.74
7H	SCRI_RS_155121	11.64
7H	SCRI_RS_138238	11.71
7H	11_20722	11.94
7H	SCRI_RS_153202	12.54
7H	SCRI_RS_235422	12.54
7H	11_10025	12.63
7H	12_30723	12.63
7H	SCRI_RS_144773	12.84
7H	SCRI_RS_137069	12.84
7H	BK_19	12.84
7H	12_31071	12.84
7H	SCRI_RS_126944	12.84
7H	SCRI_RS_147695	12.84
7H	SCRI_RS_194866	12.84
7H	SCRI_RS_174720	12.91
7H	SCRI_RS_23322	12.91
7H	SCRI_RS_240014	12.91
7H	12_30902	13.43
7H	11_21050	14.13
7H	SCRI_RS_235577	15.01
7H	SCRI_RS_152228	16.54
7H	SCRI_RS_158512	16.54
7H	SCRI_RS_142893	16.88
7H	SCRI_RS_181385	16.88
7H	SCRI_RS_222330	16.88
7H	SCRI_RS_222920	16.88
7H	SCRI_RS_230691	16.88
7H	SCRI_RS_47206	16.88
7H	SCRI_RS_98829	16.88
7H	SCRI_RS_197190	16.94
7H	11_20495	16.97
7H	12_30530	18.53
7H	SCRI_RS_127224	19.75
7H	SCRI_RS_145415	19.75
7H	SCRI_RS_166520	19.75
7H	SCRI_RS_128121	20.35
7H	SCRI_RS_150768	20.67
7H	SCRI_RS_162631	20.85
7H	12_30329	21.08
7H	SCRI_RS_47235	21.98
7H	SCRI_RS_139762	22.26
7H	11_10965	22.39
7H	SCRI_RS_148318	22.51
7H	SCRI_RS_179528	22.51
7H	SCRI_RS_213333	22.51
7H	SCRI_RS_78255	22.51
7H	12_31351	22.65
7H	12_30780	23.05
7H	SCRI_RS_200391	23.05
7H	SCRI_RS_159253	23.08
7H	12_11508	23.12
7H	SCRI_RS_140118	23.12
7H	SCRI_RS_168075	23.12
7H	SCRI_RS_203776	23.12
7H	SCRI_RS_203781	23.12
7H	11_20162	23.12
7H	12_30040	23.12
7H	11_21098	23.65
7H	SCRI_RS_165631	23.65
7H	11_10451	24.07
7H	12_30702	24.07
7H	11_10956	24.67
7H	12_30083	26.03
7H	12_30141	26.03
7H	SCRI_RS_114639	26.03
7H	SCRI_RS_154007	26.03
7H	SCRI_RS_181055	26.03
7H	SCRI_RS_190665	26.03
7H	SCRI_RS_192686	26.03
7H	SCRI_RS_193769	26.03
7H	SCRI_RS_197364	26.03
7H	SCRI_RS_198173	26.03
7H	SCRI_RS_202811	26.03
7H	SCRI_RS_227921	26.03
7H	SCRI_RS_237670	26.03
7H	SCRI_RS_45018	26.03
7H	11_10232	26.03
7H	12_30219	26.03
7H	SCRI_RS_143373	26.23
7H	SCRI_RS_145156	26.23
7H	SCRI_RS_160641	26.23
7H	SCRI_RS_169269	26.23
7H	SCRI_RS_182092	26.23
7H	SCRI_RS_204453	26.23
7H	SCRI_RS_223021	26.23
7H	SCRI_RS_229727	26.23
7H	SCRI_RS_47197	26.23
7H	SCRI_RS_170038	26.66
7H	SCRI_RS_172087	26.66
7H	12_10878	26.66
7H	SCRI_RS_143967	26.66
7H	SCRI_RS_14803	26.66
7H	SCRI_RS_165928	26.66
7H	SCRI_RS_203950	26.66
7H	11_20192	26.68
7H	11_20993	26.68
7H	SCRI_RS_160723	27.01
7H	SCRI_RS_142007	27.15
7H	SCRI_RS_163976	27.15
7H	SCRI_RS_175424	27.15
7H	SCRI_RS_179005	27.15
7H	SCRI_RS_235345	27.15
7H	SCRI_RS_150053	27.15
7H	11_20126	27.68
7H	12_30893	27.68
7H	12_30894	27.68
7H	12_30895	27.68
7H	11_10838	28.34
7H	12_10218	28.46
7H	SCRI_RS_140831	28.51
7H	SCRI_RS_220780	28.51
7H	SCRI_RS_114631	28.68
7H	SCRI_RS_138142	28.68
7H	SCRI_RS_149724	28.68
7H	SCRI_RS_171103	28.68
7H	SCRI_RS_194080	29.05
7H	SCRI_RS_219709	29.05
7H	SCRI_RS_106456	29.1
7H	SCRI_RS_140096	29.1
7H	SCRI_RS_161476	29.1
7H	SCRI_RS_29353	29.1
7H	SCRI_RS_1376	29.49
7H	SCRI_RS_230633	29.49
7H	SCRI_RS_138457	29.65
7H	SCRI_RS_138461	29.65
7H	SCRI_RS_160602	29.65
7H	SCRI_RS_236580	29.65
7H	11_10056	29.82
7H	SCRI_RS_150517	29.85
7H	SCRI_RS_153154	29.85
7H	SCRI_RS_129779	29.85
7H	12_31305	30.48
7H	SCRI_RS_172761	31.02
7H	SCRI_RS_214533	31.02
7H	SCRI_RS_168638	31.02
7H	SCRI_RS_209500	31.02
7H	SCRI_RS_201868	31.41
7H	11_10576	31.69
7H	11_10327	32.59
7H	12_30065	32.59
7H	12_10979	33.25
7H	SCRI_RS_165545	33.42
7H	BK_05	33.9
7H	12_10368	34.84
7H	12_30143	34.84
7H	11_21528	35.26
7H	12_11339	36.75
7H	SCRI_RS_155061	37.45
7H	SCRI_RS_179937	37.45
7H	11_21326	38.53
7H	SCRI_RS_209176	38.65
7H	SCRI_RS_166536	40.29
7H	11_20150	40.39
7H	SCRI_RS_91662	40.55
7H	SCRI_RS_121774	41.25
7H	SCRI_RS_193044	41.25
7H	SCRI_RS_223017	41.55
7H	SCRI_RS_37395	41.74
7H	SCRI_RS_149501	41.85
7H	SCRI_RS_8200	41.85
7H	12_30545	42.71
7H	12_30752	42.71
7H	11_10772	43.37
7H	12_30371	43.37
7H	SCRI_RS_186130	43.85
7H	SCRI_RS_202061	43.85
7H	SCRI_RS_155795	43.85
7H	11_20357	44.33
7H	11_20466	44.33
7H	12_10696	44.33
7H	12_20031	44.33
7H	SCRI_RS_187827	44.35
7H	SCRI_RS_125000	44.82
7H	SCRI_RS_152122	44.82
7H	SCRI_RS_200387	44.82
7H	SCRI_RS_205573	44.82
7H	SCRI_RS_208770	44.82
7H	11_20113	45.88
7H	11_20790	46.09
7H	12_30290	46.76
7H	SCRI_RS_198754	47.5
7H	SCRI_RS_188133	47.5
7H	11_10726	47.5
7H	12_30181	47.5
7H	SCRI_RS_169904	48.4
7H	12_31490	48.58
7H	12_10959	48.79
7H	12_30576	48.79
7H	SCRI_RS_177253	48.8
7H	SCRI_RS_162708	49
7H	SCRI_RS_6969	49
7H	SCRI_RS_157020	49.09
7H	SCRI_RS_164730	49.09
7H	SCRI_RS_172335	49.09
7H	SCRI_RS_141629	49.09
7H	SCRI_RS_157816	49.09
7H	SCRI_RS_182102	49.09
7H	SCRI_RS_750	49.09
7H	SCRI_RS_7797	49.09
7H	11_20074	49.09
7H	SCRI_RS_136918	49.1
7H	SCRI_RS_146114	49.1
7H	SCRI_RS_150016	49.1
7H	SCRI_RS_150783	49.1
7H	SCRI_RS_171008	49.1
7H	SCRI_RS_202653	49.1
7H	SCRI_RS_234502	49.1
7H	SCRI_RS_7115	49.1
7H	SCRI_RS_212956	49.3
7H	SCRI_RS_230487	49.3
7H	SCRI_RS_146145	49.3
7H	SCRI_RS_164437	49.3
7H	SCRI_RS_134872	50.49
7H	SCRI_RS_168494	50.49
7H	SCRI_RS_136556	50.54
7H	SCRI_RS_146382	50.54
7H	SCRI_RS_209511	50.54
7H	SCRI_RS_122879	50.54
7H	SCRI_RS_6608	50.54
7H	SCRI_RS_219349	51.1
7H	12_30880	51.26
7H	SCRI_RS_200953	51.49
7H	11_10346	51.91
7H	SCRI_RS_187590	52.28
7H	SCRI_RS_196063	52.28
7H	SCRI_RS_186683	52.48
7H	11_10721	52.52
7H	SCRI_RS_178619	53.04
7H	11_10050	53.84
7H	12_30149	53.84
7H	SCRI_RS_137626	54.04
7H	SCRI_RS_161111	54.09
7H	SCRI_RS_15864	54.26
7H	SCRI_RS_213842	54.26
7H	SCRI_RS_229041	54.26
7H	SCRI_RS_230478	54.26
7H	12_10605	54.8
7H	SCRI_RS_138111	55.03
7H	12_31463	55.11
7H	11_21270	55.29
7H	SCRI_RS_236651	55.29
7H	11_20750	55.46
7H	12_10657	55.46
7H	11_20975	55.46
7H	SCRI_RS_132425	55.46
7H	SCRI_RS_152299	55.46
7H	SCRI_RS_159066	55.46
7H	SCRI_RS_234226	55.46
7H	SCRI_RS_180	55.88
7H	12_10267	56.18
7H	12_11242	56.22
7H	12_31441	56.22
7H	SCRI_RS_139962	56.22
7H	SCRI_RS_182	56.22
7H	12_11525	56.22
7H	12_30639	56.22
7H	11_11098	56.31
7H	11_11348	56.31
7H	11_20671	56.31
7H	11_11028	57.24
7H	11_20060	57.24
7H	BK_03	57.89
7H	SCRI_RS_108830	57.89
7H	SCRI_RS_132654	57.89
7H	SCRI_RS_132722	57.89
7H	SCRI_RS_140553	57.89
7H	SCRI_RS_152696	57.89
7H	SCRI_RS_171588	57.89
7H	SCRI_RS_198541	57.89
7H	12_11257	58.06
7H	SCRI_RS_129686	58.06
7H	11_10431	58.28
7H	SCRI_RS_165918	58.28
7H	11_20195	58.28
7H	12_30125	58.46
7H	SCRI_RS_124819	58.86
7H	SCRI_RS_127040	58.86
7H	SCRI_RS_152074	58.86
7H	SCRI_RS_195940	58.86
7H	SCRI_RS_207354	58.86
7H	SCRI_RS_214366	58.86
7H	SCRI_RS_219081	58.86
7H	SCRI_RS_235641	58.86
7H	SCRI_RS_235584	59.01
7H	SCRI_RS_115426	59.04
7H	SCRI_RS_12729	59.04
7H	SCRI_RS_131323	59.04
7H	SCRI_RS_135346	59.04
7H	SCRI_RS_146847	59.04
7H	SCRI_RS_149647	59.04
7H	SCRI_RS_151367	59.04
7H	SCRI_RS_153388	59.04
7H	SCRI_RS_161359	59.04
7H	SCRI_RS_162419	59.04
7H	SCRI_RS_164251	59.04
7H	SCRI_RS_169639	59.04
7H	SCRI_RS_189377	59.04
7H	SCRI_RS_126380	59.04
7H	SCRI_RS_127143	59.04
7H	SCRI_RS_152635	59.04
7H	SCRI_RS_235409	59.04
7H	SCRI_RS_115208	59.09
7H	SCRI_RS_129817	59.09
7H	SCRI_RS_132879	59.09
7H	SCRI_RS_13354	59.09
7H	SCRI_RS_133777	59.09
7H	SCRI_RS_134797	59.09
7H	SCRI_RS_137364	59.09
7H	SCRI_RS_13927	59.09
7H	SCRI_RS_141164	59.09
7H	SCRI_RS_147689	59.09
7H	SCRI_RS_148407	59.09
7H	SCRI_RS_149228	59.09
7H	SCRI_RS_156971	59.09
7H	SCRI_RS_158126	59.09
7H	SCRI_RS_160279	59.09
7H	SCRI_RS_162623	59.09
7H	SCRI_RS_167383	59.09
7H	SCRI_RS_174500	59.09
7H	SCRI_RS_177060	59.09
7H	SCRI_RS_185707	59.09
7H	SCRI_RS_188905	59.09
7H	SCRI_RS_189067	59.09
7H	SCRI_RS_196678	59.09
7H	SCRI_RS_200107	59.09
7H	SCRI_RS_207127	59.09
7H	SCRI_RS_212852	59.09
7H	SCRI_RS_214425	59.09
7H	SCRI_RS_228070	59.09
7H	SCRI_RS_231300	59.09
7H	SCRI_RS_232559	59.09
7H	SCRI_RS_236675	59.09
7H	SCRI_RS_237881	59.09
7H	SCRI_RS_66459	59.09
7H	SCRI_RS_102957	59.1
7H	SCRI_RS_152698	59.1
7H	SCRI_RS_173914	59.1
7H	SCRI_RS_219291	59.1
7H	SCRI_RS_229684	59.1
7H	SCRI_RS_112718	59.13
7H	SCRI_RS_116905	59.13
7H	SCRI_RS_154111	59.13
7H	SCRI_RS_184894	59.13
7H	SCRI_RS_219749	59.13
7H	SCRI_RS_239806	59.13
7H	SCRI_RS_96550	59.23
7H	SCRI_RS_9736	59.23
7H	12_30997	59.93
7H	SCRI_RS_200890	59.99
7H	SCRI_RS_175164	60.43
7H	SCRI_RS_175859	60.43
7H	12_31452	60.59
7H	12_11103	61.25
7H	12_11202	61.25
7H	11_10299	63.22
7H	12_11045	63.22
7H	SCRI_RS_13917	63.25
7H	12_31357	63.39
7H	12_20006	63.82
7H	SCRI_RS_180004	63.82
7H	SCRI_RS_136221	63.99
7H	SCRI_RS_11068	64.03
7H	SCRI_RS_121291	64.03
7H	12_10284	64.17
7H	SCRI_RS_190570	64.17
7H	11_10983	64.58
7H	SCRI_RS_207246	65.27
7H	SCRI_RS_143118	65.37
7H	SCRI_RS_181154	65.37
7H	SCRI_RS_235888	65.37
7H	SCRI_RS_103902	65.37
7H	SCRI_RS_150062	65.37
7H	SCRI_RS_230083	65.37
7H	SCRI_RS_231912	65.37
7H	12_30999	65.37
7H	SCRI_RS_133026	65.37
7H	SCRI_RS_207238	65.37
7H	SCRI_RS_219581	65.37
7H	SCRI_RS_104566	65.5
7H	SCRI_RS_185680	65.5
7H	SCRI_RS_2914	65.5
7H	SCRI_RS_204771	65.7
7H	SCRI_RS_206747	65.7
7H	SCRI_RS_149650	65.78
7H	SCRI_RS_169649	65.78
7H	SCRI_RS_171080	65.78
7H	SCRI_RS_194085	65.78
7H	11_20885	66.56
7H	12_21167	66.56
7H	12_31120	66.56
7H	SCRI_RS_109893	67.11
7H	SCRI_RS_181727	67.11
7H	SCRI_RS_225636	67.11
7H	12_30344	68.06
7H	SCRI_RS_154193	68.19
7H	12_30496	69.48
7H	12_11254	69.83
7H	12_30832	69.83
7H	SCRI_RS_170175	69.83
7H	SCRI_RS_235046	69.83
7H	12_30646	69.98
7H	SCRI_RS_194454	70.05
7H	11_20827	70.12
7H	12_11492	70.12
7H	SCRI_RS_192587	70.12
7H	SCRI_RS_5699	70.12
7H	SCRI_RS_158234	70.12
7H	11_10256	70.12
7H	11_10083	70.12
7H	11_10153	70.12
7H	12_30581	70.12
7H	11_11122	70.19
7H	SCRI_RS_171786	70.19
7H	12_31203	71.01
7H	11_10700	71.05
7H	12_30595	71.79
7H	12_10655	71.89
7H	11_10055	71.99
7H	11_10209	71.99
7H	11_10370	71.99
7H	11_10534	71.99
7H	11_11219	71.99
7H	11_11291	71.99
7H	11_20460	71.99
7H	11_20828	71.99
7H	11_20911	71.99
7H	11_21302	71.99
7H	12_10581	71.99
7H	12_11536	71.99
7H	12_21319	71.99
7H	12_30389	71.99
7H	12_30411	71.99
7H	12_30445	71.99
7H	12_30449	71.99
7H	12_30475	71.99
7H	12_30481	71.99
7H	12_30486	71.99
7H	12_30492	71.99
7H	12_30544	71.99
7H	12_30600	71.99
7H	12_30621	71.99
7H	12_31000	71.99
7H	12_31055	71.99
7H	12_31227	71.99
7H	12_31345	71.99
7H	BK_07	71.99
7H	SCRI_RS_10407	71.99
7H	SCRI_RS_120850	71.99
7H	SCRI_RS_127006	71.99
7H	SCRI_RS_129757	71.99
7H	SCRI_RS_139480	71.99
7H	SCRI_RS_14173	71.99
7H	SCRI_RS_142605	71.99
7H	SCRI_RS_150229	71.99
7H	SCRI_RS_165137	71.99
7H	SCRI_RS_168543	71.99
7H	SCRI_RS_192888	71.99
7H	SCRI_RS_206751	71.99
7H	SCRI_RS_225475	71.99
7H	SCRI_RS_227799	71.99
7H	SCRI_RS_237304	71.99
7H	SCRI_RS_34815	71.99
7H	12_30760	71.99
7H	11_10773	71.99
7H	11_10924	71.99
7H	11_11145	71.99
7H	11_11352	71.99
7H	11_20879	71.99
7H	11_20893	71.99
7H	11_21178	71.99
7H	11_21335	71.99
7H	12_10268	71.99
7H	12_10459	71.99
7H	12_10698	71.99
7H	12_10713	71.99
7H	12_30004	71.99
7H	12_30550	71.99
7H	12_30563	71.99
7H	12_30574	71.99
7H	12_30794	71.99
7H	12_30835	71.99
7H	12_31140	71.99
7H	12_31418	71.99
7H	SCRI_RS_154541	71.99
7H	SCRI_RS_222088	71.99
7H	11_10115	71.99
7H	11_10073	71.99
7H	11_10394	71.99
7H	12_11146	71.99
7H	12_30053	71.99
7H	SCRI_RS_194569	71.99
7H	12_10222	71.99
7H	12_11477	71.99
7H	SCRI_RS_204144	71.99
7H	11_20314	71.99
7H	SCRI_RS_164280	71.99
7H	11_20200	71.99
7H	SCRI_RS_157035	72.11
7H	12_21115	72.11
7H	12_31002	72.11
7H	SCRI_RS_131490	72.11
7H	SCRI_RS_190352	72.11
7H	11_11461	72.13
7H	12_30589	72.13
7H	SCRI_RS_229174	72.85
7H	12_31211	73.29
7H	12_31513	73.91
7H	11_21300	73.91
7H	12_30565	73.91
7H	SCRI_RS_231916	73.99
7H	12_10369	73.99
7H	12_30213	73.99
7H	11_10069	74.31
7H	12_30645	74.31
7H	SCRI_RS_195741	74.31
7H	11_20205	74.31
7H	12_10125	74.31
7H	12_10982	74.31
7H	12_30506	74.31
7H	12_11377	74.51
7H	11_10673	74.79
7H	11_21079	74.79
7H	11_20880	74.99
7H	12_30998	74.99
7H	12_11529	74.99
7H	12_11499	74.99
7H	11_11239	74.99
7H	11_11445	74.99
7H	12_11055	74.99
7H	12_21520	74.99
7H	11_10442	75.49
7H	12_30790	76.28
7H	11_20349	76.55
7H	12_31199	76.71
7H	11_20896	76.99
7H	12_31137	76.99
7H	12_30199	77.09
7H	SCRI_RS_122512	77.09
7H	SCRI_RS_229585	77.49
7H	SCRI_RS_109701	77.54
7H	11_21330	77.63
7H	11_20042	77.77
7H	SCRI_RS_124478	77.77
7H	SCRI_RS_194291	77.77
7H	11_20230	77.77
7H	11_20771	77.77
7H	11_21409	77.77
7H	SCRI_RS_190420	78.19
7H	11_10303	78.33
7H	11_20083	78.43
7H	11_20485	79.22
7H	12_30419	79.22
7H	11_10143	79.22
7H	SCRI_RS_134640	80.08
7H	SCRI_RS_148722	80.08
7H	SCRI_RS_188534	80.08
7H	SCRI_RS_200020	80.08
7H	SCRI_RS_200021	80.08
7H	SCRI_RS_4114	80.08
7H	12_30301	81.89
7H	12_11437	82.51
7H	12_20685	82.51
7H	12_30026	82.51
7H	12_30996	82.51
7H	SCRI_RS_127791	82.62
7H	SCRI_RS_194841	82.62
7H	SCRI_RS_112863	84.07
7H	SCRI_RS_146157	84.07
7H	SCRI_RS_157663	85.42
7H	SCRI_RS_4562	85.42
7H	11_11343	85.42
7H	12_11044	85.82
7H	11_20311	87.07
7H	11_21103	87.07
7H	SCRI_RS_146640	87.07
7H	11_20076	87.12
7H	11_21448	87.12
7H	11_21201	87.12
7H	11_20584	87.67
7H	12_30806	88.11
7H	12_31395	88.11
7H	11_10301	89.69
7H	SCRI_RS_136590	90.12
7H	SCRI_RS_14491	90.12
7H	SCRI_RS_153505	90.12
7H	SCRI_RS_174514	90.12
7H	SCRI_RS_174699	90.12
7H	SCRI_RS_181338	90.12
7H	SCRI_RS_193197	90.12
7H	SCRI_RS_195937	90.12
7H	SCRI_RS_202464	90.12
7H	SCRI_RS_204256	90.12
7H	SCRI_RS_208890	90.12
7H	SCRI_RS_222286	90.12
7H	SCRI_RS_229415	90.12
7H	SCRI_RS_4556	90.12
7H	11_21173	90.23
7H	SCRI_RS_157912	90.23
7H	12_31188	90.28
7H	SCRI_RS_168994	90.62
7H	SCRI_RS_136586	90.93
7H	SCRI_RS_139808	90.93
7H	SCRI_RS_141732	90.93
7H	SCRI_RS_149645	90.93
7H	SCRI_RS_150049	90.93
7H	SCRI_RS_222863	90.93
7H	SCRI_RS_7974	90.93
7H	11_20808	91.14
7H	12_30335	91.14
7H	SCRI_RS_143884	92.87
7H	SCRI_RS_155169	93.02
7H	SCRI_RS_196885	93.02
7H	11_20103	93.02
7H	12_20611	93.02
7H	12_30630	93.22
7H	12_31440	93.22
7H	12_31294	93.35
7H	SCRI_RS_162966	93.37
7H	SCRI_RS_173459	93.37
7H	12_11051	93.49
7H	12_21464	94.29
7H	11_10169	94.95
7H	12_31261	96.76
7H	11_20824	97.42
7H	SCRI_RS_175893	97.6
7H	SCRI_RS_182503	97.6
7H	SCRI_RS_161667	98.28
7H	SCRI_RS_208893	98.28
7H	12_20684	98.28
7H	12_10362	98.28
7H	12_20406	98.28
7H	12_10897	98.59
7H	SCRI_RS_186083	98.59
7H	SCRI_RS_197282	98.59
7H	11_10853	99.13
7H	11_20092	99.13
7H	11_20385	99.13
7H	SCRI_RS_112204	99.42
7H	SCRI_RS_124251	99.42
7H	SCRI_RS_172243	99.42
7H	SCRI_RS_174327	99.42
7H	SCRI_RS_189409	99.42
7H	SCRI_RS_219260	99.42
7H	SCRI_RS_7217	99.42
7H	11_20623	100.33
7H	SCRI_RS_109484	100.33
7H	SCRI_RS_185529	100.33
7H	SCRI_RS_231275	100.92
7H	12_10652	100.97
7H	11_10563	101.28
7H	SCRI_RS_174285	101.28
7H	SCRI_RS_126397	102.48
7H	SCRI_RS_1347	102.48
7H	SCRI_RS_135181	102.48
7H	SCRI_RS_152504	102.48
7H	SCRI_RS_152752	102.48
7H	SCRI_RS_161285	102.48
7H	SCRI_RS_166774	102.48
7H	SCRI_RS_172331	102.48
7H	SCRI_RS_173554	102.48
7H	SCRI_RS_174255	102.48
7H	SCRI_RS_239722	102.48
7H	SCRI_RS_107367	102.83
7H	12_30362	102.94
7H	11_20247	104.26
7H	12_21208	104.26
7H	12_30797	104.92
7H	SCRI_RS_16798	106.24
7H	12_11184	106.42
7H	12_30164	106.42
7H	SCRI_RS_142219	106.42
7H	12_30368	106.5
7H	SCRI_RS_204483	108.17
7H	12_20217	108.5
7H	SCRI_RS_153261	108.54
7H	SCRI_RS_13570	108.69
7H	SCRI_RS_160611	108.69
7H	SCRI_RS_205991	108.69
7H	11_11243	108.76
7H	12_31232	108.76
7H	SCRI_RS_144462	109.25
7H	12_10543	109.34
7H	SCRI_RS_152402	109.77
7H	SCRI_RS_154545	110.95
7H	SCRI_RS_156412	110.95
7H	SCRI_RS_204424	110.95
7H	SCRI_RS_224574	110.95
7H	12_31535	111.9
7H	12_31254	111.9
7H	SCRI_RS_159555	111.9
7H	SCRI_RS_163463	111.9
7H	SCRI_RS_172062	111.9
7H	SCRI_RS_175568	111.9
7H	SCRI_RS_189107	111.9
7H	11_20998	111.98
7H	SCRI_RS_128439	111.98
7H	SCRI_RS_142295	111.98
7H	SCRI_RS_151242	111.98
7H	SCRI_RS_154664	111.98
7H	SCRI_RS_176094	111.98
7H	SCRI_RS_197833	111.98
7H	SCRI_RS_206926	111.98
7H	SCRI_RS_213939	111.98
7H	SCRI_RS_93571	111.98
7H	SCRI_RS_130990	112.44
7H	SCRI_RS_138715	112.44
7H	SCRI_RS_150401	112.44
7H	SCRI_RS_150955	112.44
7H	SCRI_RS_155489	112.44
7H	SCRI_RS_163468	112.44
7H	SCRI_RS_214028	112.44
7H	11_20354	112.56
7H	12_31374	114.92
7H	SCRI_RS_1383	115.01
7H	11_10182	116.12
7H	11_21229	116.12
7H	SCRI_RS_146324	116.12
7H	SCRI_RS_196730	116.12
7H	SCRI_RS_202130	116.12
7H	12_20832	117.29
7H	11_21209	117.69
7H	SCRI_RS_206189	118.25
7H	SCRI_RS_206913	118.25
7H	11_21104	119.04
7H	SCRI_RS_181575	119.04
7H	SCRI_RS_235853	119.04
7H	11_10861	119.9
7H	SCRI_RS_237466	119.9
7H	SCRI_RS_69124	119.9
7H	SCRI_RS_2896	120.51
7H	SCRI_RS_195908	120.51
7H	SCRI_RS_161703	120.51
7H	SCRI_RS_133561	120.65
7H	SCRI_RS_145605	120.65
7H	SCRI_RS_229953	120.65
7H	SCRI_RS_125205	121.86
7H	11_21160	122
7H	SCRI_RS_126437	122
7H	SCRI_RS_149709	122
7H	SCRI_RS_154003	122
7H	SCRI_RS_155078	122
7H	SCRI_RS_162972	122
7H	SCRI_RS_178933	122
7H	SCRI_RS_179554	122
7H	SCRI_RS_180889	122
7H	SCRI_RS_187873	122
7H	SCRI_RS_207086	122
7H	SCRI_RS_220680	122
7H	SCRI_RS_229573	122
7H	11_10797	122
7H	SCRI_RS_140746	122
7H	SCRI_RS_151387	122
7H	SCRI_RS_193330	122
7H	SCRI_RS_198785	122
7H	SCRI_RS_200937	122
7H	SCRI_RS_149573	122
7H	SCRI_RS_196031	122
7H	SCRI_RS_5518	122
7H	12_30380	122.36
7H	SCRI_RS_225155	122.4
7H	SCRI_RS_154069	122.4
7H	12_30308	122.57
7H	SCRI_RS_151523	122.57
7H	SCRI_RS_155132	122.57
7H	SCRI_RS_994	122.57
7H	11_10078	123.11
7H	SCRI_RS_184488	123.4
7H	12_10973	123.45
7H	SCRI_RS_4520	123.6
7H	SCRI_RS_154327	123.98
7H	SCRI_RS_187512	123.98
7H	12_31282	124.05
7H	SCRI_RS_182575	124.23
7H	11_10454	124.49
7H	11_10885	124.63
7H	SCRI_RS_174159	124.63
7H	11_20847	124.68
7H	11_10687	125.39
7H	SCRI_RS_237742	125.39
7H	12_30761	125.74
7H	11_20139	125.85
7H	SCRI_RS_171456	125.85
7H	11_21280	126.37
7H	11_10550	127.46
7H	12_31325	127.46
7H	SCRI_RS_130821	127.46
7H	SCRI_RS_130825	127.46
7H	SCRI_RS_138598	127.46
7H	SCRI_RS_141470	127.46
7H	SCRI_RS_185445	127.46
7H	SCRI_RS_219233	127.46
7H	11_10289	127.92
7H	SCRI_RS_134057	127.92
7H	SCRI_RS_135189	128.06
7H	SCRI_RS_159164	128.06
7H	SCRI_RS_188993	128.06
7H	SCRI_RS_131173	128.06
7H	12_31241	128.35
7H	SCRI_RS_174164	128.35
7H	SCRI_RS_177876	128.35
7H	SCRI_RS_178796	128.42
7H	SCRI_RS_189592	128.49
7H	11_20691	128.53
7H	11_21363	128.83
7H	11_10843	128.83
7H	11_11440	128.83
7H	SCRI_RS_197886	128.83
7H	SCRI_RS_223076	128.83
7H	11_21223	128.83
7H	SCRI_RS_206322	128.83
7H	12_10677	128.83
7H	12_20241	128.83
7H	12_20949	128.83
7H	12_31166	128.83
7H	SCRI_RS_202141	129.08
7H	SCRI_RS_219948	130.05
7H	12_30593	130.16
7H	SCRI_RS_160259	130.33
7H	SCRI_RS_164623	130.33
7H	SCRI_RS_16316	130.33
7H	SCRI_RS_155652	130.47
7H	SCRI_RS_171853	130.47
7H	11_20414	130.81
7H	12_31491	130.9
7H	SCRI_RS_154647	130.9
7H	SCRI_RS_184967	131.18
7H	12_20640	131.51
7H	SCRI_RS_140282	131.66
7H	SCRI_RS_141495	131.66
7H	SCRI_RS_179575	131.66
7H	SCRI_RS_132118	132.37
7H	SCRI_RS_148742	132.37
7H	SCRI_RS_200086	132.37
7H	SCRI_RS_120720	132.53
7H	SCRI_RS_165689	132.53
7H	SCRI_RS_182331	132.53
7H	SCRI_RS_184902	132.53
7H	11_11012	132.54
7H	SCRI_RS_183593	132.83
7H	11_10130	133.2
7H	12_20207	133.23
7H	12_21016	133.43
7H	SCRI_RS_4604	133.43
7H	SCRI_RS_197550	133.73
7H	11_10896	133.73
7H	SCRI_RS_53474	133.8
7H	12_21328	134.03
7H	SCRI_RS_213791	134.33
7H	12_11309	134.33
7H	SCRI_RS_153546	134.43
7H	SCRI_RS_6430	134.43
7H	SCRI_RS_86194	134.43
7H	11_11275	134.43
7H	SCRI_RS_120015	135.35
7H	SCRI_RS_150302	135.35
7H	11_20962	135.64
7H	SCRI_RS_121057	136.03
7H	SCRI_RS_165668	136.03
7H	SCRI_RS_198005	136.03
7H	SCRI_RS_752	136.03
7H	SCRI_RS_204567	136.32
7H	11_20117	136.42
7H	12_20079	136.42
7H	SCRI_RS_169044	136.42
7H	SCRI_RS_224666	136.42
7H	SCRI_RS_14119	137.96
7H	11_20586	138.98
7H	SCRI_RS_126143	139.07
7H	12_30974	139.12
7H	11_10999	139.12
7H	12_10888	139.14
7H	11_20504	140.13
7H	SCRI_RS_10111	142.42
7H	SCRI_RS_200397	142.42
7H	SCRI_RS_6252	142.42
7H	11_10547	142.52
7H	SCRI_RS_219515	142.52
7H	SCRI_RS_166176	142.52
7H	SCRI_RS_123211	142.59
7H	SCRI_RS_179198	142.59
7H	SCRI_RS_206804	142.59
7H	11_20170	142.62
7H	SCRI_RS_157208	142.62
7H	SCRI_RS_157219	142.62
7H	SCRI_RS_158599	142.62
7H	SCRI_RS_193505	142.62
7H	SCRI_RS_204098	142.62
7H	SCRI_RS_168479	142.72
7H	SCRI_RS_169268	142.72
7H	11_20185	142.72
7H	SCRI_RS_214451	142.72
7H	SCRI_RS_230261	142.82
7H	12_30827	142.86
7H	SCRI_RS_131108	142.86
7H	SCRI_RS_167617	142.86
7H	11_10174	142.92
7H	12_30826	142.92
7H	11_20365	143.02
7H	12_10378	143.32
7H	12_31019	143.32
```
