# Supplementary material for: Genome‐Wide Association Analysis of Grain Yield‐Associated Traits in a Pan‐European Barley Cultivar Collection
Source: Plant Genome. 2018 Mar 1;11(1):170073. doi: 10.3835/plantgenome2017.08.0073 (PMC12962562; doi:10.3835/plantgenome2017.08.0073)
Supplement: Supplementary file 3 [file TPG2-11-170073-s003.docx]

**Supplemental Table S3: Associated data for grain trait QTLs in this study**

| **QTL number** | **Chromosome** | **Position** | | | **Trait** | **Growth Habit** | **Probability** | **QTL type** | **Cross-Over?** | **RefGeno f** | **Effect of minor allele at location** | | | | | | | | **Std error** | **QTL HOT SPOT** |
| --- | --- | --- | --- | --- | --- | --- | --- | --- | --- | --- | --- | --- | --- | --- | --- | --- | --- | --- | --- | --- |
|  |  | **Min** | **Peak** | **Max** |  |  |  |  |  |  | **CR 09** | **CR 10** | **HA09** | **HA10** | **IP 09** | **IP 10** | **SC 09** | **SC 10** |  |  |
| 1 | 1H | 0.00 | 0.00 | 0.00 | **GR** | **S** | **4.26** | Main | No | 0.18 | 0.00 | 0.00 | 0.00 | 0.00 | 0.00 | 0.00 | 0.00 | 0.00 | 0.00 |  |
| 2 | 1H | 2.13 | 2.51 | 4.75 | **TGW** | **W** | **4.20** | QTLxE | No | 0.42 | -0.94 | 0.09 | -1.67 | -1.21 | -1.54 | -0.73 | -1.25 | -0.83 | 0.40 |  |
| 3 | 1H | 8.69 | 8.78 | 10.08 | **GA** | **S** | **4.54** | Main | No | 0.33 | -0.35 | -0.35 | -0.35 | -0.35 | -0.35 | -0.35 | -0.35 | -0.35 | 0.08 |  |
| 4 | 1H | 23.85 | 24.10 | 25.26 | **GR** | **S** | **4.25** | Main | No | 0.95 | 0.01 | 0.01 | 0.01 | 0.01 | 0.01 | 0.01 | 0.01 | 0.01 | 0.00 |  |
| 5 | 1H | 32.45 | 34.27 | 35.59 | **GA** | **W** | **8.38** | QTLxE | No | 0.83 | -0.86 | -0.57 | -1.18 | -1.08 | -1.45 | -1.37 | -1.48 | -1.13 | 0.26 |  |
| 6 | 1H | 32.45 | 34.27 | 35.59 | **GL** | **W** | **11.51** | QTLxE | No | 0.83 | -0.23 | -0.21 | -0.45 | -0.50 | -0.47 | -0.49 | -0.48 | -0.41 | 0.09 |  |
| 7 | 1H | 44.31 | 44.41 | 44.56 | **G/E** | **S** | **10.18** | Main | No | 0.87 | 0.00 | 0.00 | 4.39 | 4.39 | 4.39 | 4.39 | 4.39 | 4.39 | 0.90 |  |
| 8 | 1H | 45.39 | 45.67 | 50.63 | **GA** | **S** | **5.45** | Main | No | 0.84 | 0.77 | 0.77 | 0.77 | 0.77 | 0.77 | 0.77 | 0.77 | 0.77 | 0.19 |  |
| 9 | 1H | 45.39 | 45.67 | 50.63 | **GL** | **S** | **6.85** | Main | No | 0.84 | 0.17 | 0.17 | 0.17 | 0.17 | 0.17 | 0.17 | 0.17 | 0.17 | 0.05 |  |
| 10 | 1H | 46.67 | 47.60 | 48.92 | **GA** | **W** | **6.27** | QTLxE | No | 0.36 | -0.38 | -0.26 | -0.57 | -0.51 | -0.95 | -0.89 | -0.57 | -0.53 | 0.17 |  |
| 11 | 1H | 49.36 | 49.56 | 53.82 | **GL** | **W** | **8.26** | QTLxE | No | 0.64 | 0.17 | 0.21 | 0.42 | 0.34 | 0.30 | 0.20 | 0.31 | 0.30 | 0.07 |  |
| 12 | 1H | 50.28 | 50.56 | 57.94 | **GW** | **S** | **4.85** | Main | No | 0.78 | -0.08 | -0.08 | -0.08 | -0.08 | -0.08 | -0.08 | -0.08 | -0.08 | 0.02 | **1_1** |
| 13 | 1H | 50.28 | 50.56 | 57.94 | **TGW** | **S** | **5.69** | Main | No | 0.78 | -1.76 | -1.76 | -1.76 | -1.76 | -1.76 | -1.76 | -1.76 | -1.76 | 0.46 | **1_1** |
| 14 | 1H | 50.28 | 50.56 | 57.94 | **GR** | **S** | **9.03** | Main | No | 0.76 | -0.01 | -0.01 | -0.01 | -0.01 | -0.01 | -0.01 | -0.01 | -0.01 | 0.00 | **1_1** |
| 15 | 1H | 54.36 | 54.72 | 54.91 | **G/E** | **S** | **9.05** | QTLxE | No | 0.91 | 0.00 | 0.00 | 3.92 | 1.81 | 3.67 | 0.69 | 3.42 | 2.64 | 0.74 | **1_1** |
| 16 | 1H | 63.80 | 63.90 | 64.56 | **GL** | **W** | **5.23** | QTLxE | No | 0.33 | 0.13 | -0.01 | 0.17 | 0.26 | 0.26 | 0.23 | 0.23 | 0.24 | 0.07 |  |
| 17 | 1H | 85.52 | 87.03 | 89.75 | **GY** | **W** | **5.54** | QTLxE | No | 0.86 | -0.05 | 0.00 | -0.37 | -0.22 | -0.79 | 0.00 | -0.44 | -0.39 | 0.16 |  |
| 18 | 1H | 88.25 | 88.29 | 88.42 | **GL** | **W** | **4.76** | QTLxE | No | 0.60 | 0.07 | 0.08 | 0.16 | 0.21 | 0.20 | 0.25 | 0.24 | 0.24 | 0.06 |  |
| 19 | 1H | 92.32 | 93.64 | 95.36 | **GA** | **S** | **6.28** | Main | No | 0.83 | -0.90 | -0.90 | -0.90 | -0.90 | -0.90 | -0.90 | -0.90 | -0.90 | 0.24 | **1_2** |
| 20 | 1H | 92.32 | 93.64 | 95.36 | **GW** | **S** | **8.14** | Main | No | 0.83 | -0.11 | -0.11 | -0.11 | -0.11 | -0.11 | -0.11 | -0.11 | -0.11 | 0.03 | **1_2** |
| 21 | 1H | 92.32 | 93.64 | 95.36 | **G/E** | **S** | **32.73** | Main | No | 0.83 | 0.00 | 0.00 | 8.37 | 8.37 | 8.37 | 8.37 | 8.37 | 8.37 | 0.87 | **1_2** |
| 22 | 1H | 92.32 | 93.64 | 95.36 | **TGW** | **S** | **10.57** | QTLxE | No | 0.83 | -2.66 | -1.86 | -4.24 | -3.22 | -3.80 | -3.75 | -3.35 | -3.73 | 0.67 | **1_2** |
| 23 | 1H | 96.21 | 96.26 | 96.57 | **GL** | **S** | **14.32** | QTLxE | No | 0.86 | 0.15 | 0.18 | 0.32 | 0.32 | 0.31 | 0.35 | 0.38 | 0.30 | 0.06 |  |
| 24 | 1H | 96.21 | 96.26 | 101.25 | **GR** | **S** | **8.87** | QTLxE | No | 0.86 | -0.01 | -0.01 | -0.01 | -0.01 | -0.02 | -0.02 | -0.02 | -0.02 | 0.00 |  |
| 25 | 1H | 100.04 | 100.82 | 100.97 | **TGW** | **W** | **6.55** | QTLxE | No | 0.71 | -1.36 | -0.09 | -1.35 | -1.61 | -2.05 | -1.88 | -2.22 | -1.67 | 0.46 |  |
| 26 | 1H | 100.04 | 101.00 | 101.25 | **GL** | **W** | **6.80** | QTLxE | No | 0.56 | -0.04 | 0.07 | 0.31 | 0.22 | 0.24 | 0.23 | 0.32 | 0.34 | 0.09 |  |
| 27 | 1H | 100.97 | 101.00 | 101.25 | **GR** | **W** | **10.16** | QTLxE | No | 0.56 | 0.00 | 0.00 | -0.02 | -0.01 | -0.01 | -0.01 | -0.02 | -0.02 | 0.00 |  |
| 28 | 1H | 105.01 | 105.23 | 105.37 | **GY** | **W** | **5.01** | QTLxE | No | 0.79 | 0.08 | -0.25 | -0.43 | -0.38 | -0.32 | -0.12 | -0.30 | -0.32 | 0.14 |  |
| 29 | 1H | 110.50 | 111.80 | 114.95 | **G/E** | **S** | **4.10** | QTLxE | No | 0.88 | 0.00 | 0.00 | 2.53 | 2.27 | 2.23 | 0.63 | 3.60 | 2.24 | 0.77 |  |
| 30 | 1H | 114.95 | 115.75 | 116.80 | **GA** | **W** | **4.83** | QTLxE | No | 0.91 | 0.81 | 0.40 | 0.94 | 0.87 | 1.22 | 1.13 | 0.63 | 0.53 | 0.25 |  |
| 31 | 1H | 114.95 | 115.75 | 116.80 | **TGW** | **W** | **8.12** | QTLxE | No | 0.91 | 1.95 | 0.48 | 3.29 | 2.54 | 3.53 | 2.95 | 3.34 | 1.92 | 0.68 |  |
| 32 | 1H | 130.04 | 130.52 | 130.56 | **G/E** | **W** | **4.34** | QTLxE | No | 0.69 | 0.00 | 0.00 | 1.47 | 0.95 | 0.16 | 2.83 | 0.81 | -1.00 | 0.71 |  |
| 33 | 1H | 130.52 | 130.56 | 130.66 | **GA** | **W** | **5.27** | QTLxE | No | 0.53 | 0.45 | 0.19 | 0.43 | 0.61 | 0.82 | 0.63 | 0.60 | 0.64 | 0.16 |  |
| 34 | 1H | 130.52 | 130.56 | 130.66 | **GL** | **W** | **4.57** | QTLxE | No | 0.72 | 0.07 | 0.06 | 0.10 | 0.18 | 0.16 | 0.20 | 0.19 | 0.17 | 0.05 |  |
| 35 | 1H | 130.52 | 130.56 | 130.66 | **TGW** | **W** | **4.12** | QTLxE | No | 0.53 | 1.11 | 0.54 | 0.14 | 1.26 | 1.66 | 1.17 | 2.03 | 1.20 | 0.44 |  |
| 36 | 1H | 133.37 | 134.45 | 134.97 | **G/E** | **S** | **5.96** | QTLxE | No | 0.94 | 0.00 | 0.00 | 2.47 | 1.08 | 2.24 | 1.10 | 2.54 | 1.75 | 0.54 |  |
| 37 | 1H | 136.24 | 136.25 | 136.36 | **GA** | **S** | **5.55** | Main | No | 0.65 | -0.28 | -0.28 | -0.28 | -0.28 | -0.28 | -0.28 | -0.28 | -0.28 | 0.09 |  |
| 38 | 1H | 136.24 | 136.36 | 136.43 | **EL** | **S** | **4.91** | QTLxE | No | 0.67 | -0.28 | -0.13 | -0.28 | -0.32 | -0.24 | -0.26 | -0.02 | -0.03 | 0.08 |  |
| 39 | 1H | 136.24 | 136.36 | 136.43 | **GL** | **S** | **5.10** | Main | No | 0.67 | -0.07 | -0.07 | -0.07 | -0.07 | -0.07 | -0.07 | -0.07 | -0.07 | 0.02 |  |
| 40 | 2H | 8.46 | 9.06 | 9.41 | **G/E** | **S** | **5.73** | Main | No | 0.80 | 0.00 | 0.00 | -2.52 | -2.52 | -2.52 | -2.52 | -2.52 | -2.52 | 0.47 |  |
| 41 | 2H | 8.46 | 9.06 | 9.41 | **GW** | **W** | **4.07** | QTLxE | No | 0.34 | 0.04 | -0.01 | 0.05 | 0.05 | 0.05 | 0.06 | 0.04 | 0.05 | 0.02 |  |
| 42 | 2H | 17.02 | 17.04 | 18.57 | **GA** | **W** | **6.25** | Main | No | 0.50 | 0.55 | 0.55 | 0.55 | 0.55 | 0.55 | 0.55 | 0.55 | 0.55 | 0.14 |  |
| 43 | 2H | 14.22 | 20.09 | 20.15 | **GL** | **W** | **6.87** | QTLxE | No | 0.55 | 0.09 | 0.08 | 0.28 | 0.19 | 0.25 | 0.19 | 0.23 | 0.17 | 0.05 |  |
| 44 | 2H | 19.37 | 20.09 | 20.15 | **GR** | **W** | **11.65** | QTLxE | No | 0.55 | -0.01 | 0.00 | -0.01 | -0.01 | -0.01 | -0.01 | -0.01 | -0.01 | 0.00 |  |
| 45 | 2H | 21.25 | 21.34 | 25.60 | **GY** | **W** | **6.06** | QTLxE | No | 0.31 | -0.10 | -0.12 | 0.47 | 0.17 | 0.27 | 0.03 | 0.38 | 0.36 | 0.14 |  |
| 46 | 2H | 21.25 | 30.80 | 31.55 | **GW** | **W** | **4.43** | QTLxE | No | 0.23 | 0.03 | -0.01 | 0.04 | 0.06 | 0.06 | 0.05 | 0.06 | 0.06 | 0.02 |  |
| 47 | 2H | 27.14 | 30.29 | 30.80 | **GL** | **W** | **7.29** | QTLxE | No | 0.66 | -0.07 | -0.10 | -0.28 | -0.21 | -0.16 | -0.14 | -0.22 | -0.20 | 0.05 |  |
| 48 | 2H | 30.80 | 31.55 | 31.78 | **G/E** | **S** | **4.74** | QTLxE | No | 0.65 | 0.00 | 0.00 | -1.16 | -0.16 | -1.26 | -1.03 | 0.05 | 0.28 | 0.40 |  |
| 49 | 2H | 31.78 | 31.99 | 41.88 | **GA** | **W** | **5.92** | QTLxE | No | 0.15 | -0.76 | -0.29 | -0.80 | -1.02 | -1.22 | -1.16 | -0.64 | -0.76 | 0.23 |  |
| 50 | 2H | 42.50 | 42.78 | 47.51 | **GL** | **S** | **5.40** | QTLxE | No | 0.65 | 0.06 | 0.04 | 0.10 | 0.07 | 0.14 | 0.13 | 0.10 | 0.07 | 0.03 |  |
| 51 | 2H | 42.50 | 42.78 | 47.51 | **GR** | **S** | **5.16** | Main | No | 0.65 | 0.00 | 0.00 | 0.00 | 0.00 | 0.00 | 0.00 | 0.00 | 0.00 | 0.00 |  |
| 52 | 2H | 45.01 | 46.89 | 46.91 | **EL** | **S** | **7.54** | QTLxE | No | 0.80 | 0.20 | 0.16 | 0.22 | 0.45 | 0.32 | 0.45 | 0.15 | 0.37 | 0.09 |  |
| 53 | 2H | 60.65 | 60.72 | 61.06 | **GA** | **W** | **10.01** | QTLxE | No | 0.23 | 0.45 | 0.34 | 0.69 | 0.79 | 1.11 | 1.12 | 1.11 | 0.77 | 0.19 | **2_1** |
| 54 | 2H | 60.65 | 60.72 | 61.06 | **G/E** | **W** | **5.02** | QTLxE | No | 0.24 |  |  | -3.26 | -2.05 | -0.69 | -2.47 | -1.98 | -0.32 | 0.73 | **2_1** |
| 55 | 2H | 60.91 | 61.01 | 61.04 | **GA** | **S** | **5.85** | Main | No | 0.83 | -0.72 | -0.72 | -0.72 | -0.72 | -0.72 | -0.72 | -0.72 | -0.72 | 0.16 | **2_1** |
| 56 | 2H | 54.75 | 61.01 | 61.04 | **GW** | **S** | **4.08** | QTLxE | No | 0.54 | -0.01 | -0.02 | -0.04 | -0.04 | -0.03 | -0.03 | -0.04 | -0.04 | 0.01 | **2_1** |
| 57 | 2H | 60.91 | 61.01 | 61.04 | **TGW** | **S** | **5.20** | QTLxE | No | 0.83 | -1.28 | -0.92 | -0.85 | -2.34 | -1.82 | -1.56 | -1.60 | -1.15 | 0.46 | **2_1** |
| 58 | 2H | 60.94 | 61.01 | 61.06 | **EL** | **W** | **6.07** | QTLxE | No | 0.17 | 0.43 | 0.16 | 0.70 | 0.48 | 0.57 | -4.64 | 1.08 | -11.98 | 1.68 | **2_1** |
| 59 | 2H | 59.35 | 61.01 | 61.06 | **GL** | **W** | **7.03** | QTLxE | No | 0.23 | 0.09 | 0.09 | 0.20 | 0.22 | 0.28 | 0.34 | 0.28 | 0.19 | 0.06 | **2_1** |
| 60 | 2H | 60.65 | 61.01 | 61.06 | **TGW** | **W** | **5.18** | QTLxE | No | 0.13 | -0.44 | 0.76 | 2.54 | 1.28 | 1.19 | 0.71 | 4.26 | 1.87 | 0.86 | **2_1** |
| 61 | 2H | 75.21 | 75.56 | 76.11 | **GL** | **W** | **15.31** | QTLxE | No | 0.82 | 0.15 | 0.10 | 0.40 | 0.41 | 0.30 | 0.37 | 0.35 | 0.29 | 0.06 |  |
| 62 | 2H | 75.21 | 75.56 | 76.11 | **GR** | **W** | **11.49** | QTLxE | No | 0.82 | -0.01 | -0.01 | -0.02 | -0.02 | -0.01 | -0.01 | -0.01 | -0.01 | 0.00 |  |
| 63 | 2H | 76.71 | 76.89 | 77.01 | **GR** | **S** | **8.75** | QTLxE | No | 0.94 | 0.00 | -0.01 | -0.01 | -0.01 | -0.01 | -0.01 | -0.02 | -0.01 | 0.00 |  |
| 64 | 2H | 79.01 | 79.11 | 79.97 | **G/E** | **S** | **12.03** | Main | No | 0.80 | 0.00 | 0.00 | 5.07 | 5.07 | 5.07 | 5.07 | 5.07 | 5.07 | 0.76 | **2_2** |
| 65 | 2H | 79.11 | 79.97 | 82.17 | **EL** | **S** | **6.24** | QTLxE | No | 0.86 | -0.17 | 0.00 | -0.60 | -0.80 | -0.75 | -0.61 | -0.71 | -0.33 | 0.21 | **2_2** |
| 66 | 2H | 79.11 | 79.97 | 82.17 | **GW** | **S** | **4.93** | QTLxE | No | 0.86 | -0.08 | -0.09 | -0.16 | -0.07 | -0.08 | -0.07 | -0.05 | -0.08 | 0.03 | **2_2** |
| 67 | 2H | 78.31 | 79.97 | 82.87 | **TGW** | **S** | **5.12** | Main | No | 0.86 | -1.56 | -1.56 | -1.56 | -1.56 | -1.56 | -1.56 | -1.56 | -1.56 | 0.68 | **2_2** |
| 68 | 2H | 79.97 | 82.17 | 82.87 | **TGW** | **W** | **4.37** | Main | No | 0.47 | 2.04 | 2.04 | 2.04 | 2.04 | 2.04 | 2.04 | 2.04 | 2.04 | 0.80 | **2_2** |
| 69 | 2H | 82.17 | 82.27 | 82.87 | **GA** | **S** | **10.57** | QTLxE | No | 0.87 | -0.78 | -0.64 | -1.33 | -1.01 | -1.17 | -1.53 | -0.81 | -0.62 | 0.22 |  |
| 70 | 2H | 82.17 | 82.27 | 82.87 | **GL** | **S** | **14.96** | QTLxE | No | 0.87 | -0.19 | -0.19 | -0.36 | -0.32 | -0.36 | -0.51 | -0.24 | -0.15 | 0.06 |  |
| 71 | 2H | 91.54 | 91.76 | 92.11 | **GR** | **W** | **4.91** | QTLxE | Yes | 0.61 | 0.00 | 0.01 | -0.01 | 0.00 | 0.00 | 0.00 | 0.00 | -0.01 | 0.00 |  |
| 72 | 2H | 93.44 | 94.80 | 94.99 | **GA** | **W** | **4.78** | QTLxE | No | 0.58 | 0.34 | 0.03 | 0.49 | 0.47 | 0.83 | 0.59 | 0.42 | 0.32 | 0.16 |  |
| 73 | 2H | 91.54 | 94.80 | 96.02 | **GW** | **W** | **4.56** | QTLxE | No | 0.54 | 0.04 | 0.00 | 0.04 | 0.03 | 0.06 | 0.06 | 0.03 | 0.03 | 0.01 | **2_3** |
| 74 | 2H | 86.54 | 95.88 | 97.18 | **GL** | **S** | **4.82** | Main | No | 0.14 | 0.13 | 0.13 | 0.13 | 0.13 | 0.13 | 0.13 | 0.13 | 0.13 | 0.03 | **2_3** |
| 75 | 2H | 93.44 | 96.00 | 96.02 | **GA** | **S** | **6.51** | Main | No | 0.79 | -0.52 | -0.52 | -0.52 | -0.52 | -0.52 | -0.52 | -0.52 | -0.52 | 0.16 | **2_3** |
| 76 | 2H | 95.88 | 96.00 | 96.02 | **GW** | **S** | **4.57** | Main | No | 0.79 | -0.04 | -0.04 | -0.04 | -0.04 | -0.04 | -0.04 | -0.04 | -0.04 | 0.02 | **2_3** |
| 77 | 2H | 95.88 | 96.00 | 96.02 | **TGW** | **S** | **5.07** | QTLxE | No | 0.79 | -0.89 | -0.81 | -1.53 | -1.43 | -1.96 | -2.17 | -1.74 | -1.14 | 0.44 | **2_3** |
| 78 | 2H | 104.81 | 105.18 | 105.85 | **GR** | **S** | **5.05** | Main | No | 0.64 | -0.01 | -0.01 | -0.01 | -0.01 | -0.01 | -0.01 | -0.01 | -0.01 | 0.00 |  |
| 79 | 2H | 111.49 | 113.58 | 113.69 | **G/E** | **S** | **5.35** | Main | No | 0.64 | 0.00 | 0.00 | -1.69 | -1.69 | -1.69 | -1.69 | -1.69 | -1.69 | 0.37 | **2_4** |
| 80 | 2H | 107.07 | 113.68 | 113.69 | **GA** | **S** | **5.28** | QTLxE | No | 0.64 | 0.14 | 0.25 | 0.43 | 0.32 | 0.48 | 0.48 | 0.19 | 0.16 | 0.10 | **2_4** |
| 81 | 2H | 107.07 | 113.68 | 114.18 | **GW** | **S** | **4.40** | QTLxE | No | 0.64 | 0.02 | 0.02 | 0.06 | 0.04 | 0.04 | 0.03 | 0.02 | 0.02 | 0.01 | **2_4** |
| 82 | 2H | 115.68 | 115.82 | 115.98 | **GL** | **S** | **5.63** | QTLxE | No | 0.08 | 0.10 | 0.08 | 0.22 | 0.14 | 0.21 | 0.18 | 0.25 | 0.22 | 0.06 |  |
| 83 | 2H | 105.85 | 115.82 | 115.98 | **TGW** | **S** | **4.49** | Main | No | 0.08 | 1.88 | 1.88 | 1.88 | 1.88 | 1.88 | 1.88 | 1.88 | 1.88 | 0.56 | **2_4** |
| 84 | 2H | 122.48 | 122.56 | 122.90 | **GR** | **S** | **4.56** | QTLxE | No | 0.09 | 0.01 | 0.01 | 0.01 | 0.01 | 0.02 | 0.01 | 0.01 | 0.01 | 0.00 |  |
| 85 | 2H | 122.16 | 126.15 | 126.20 | **G/E** | **S** | **4.62** | QTLxE | No | 0.36 | 0.00 | 0.00 | -1.33 | -0.46 | -0.94 | -1.10 | 0.21 | -0.20 | 0.35 |  |
| 86 | 2H | 132.96 | 133.26 | 133.56 | **GR** | **S** | **4.45** | QTLxE | No | 0.12 | 0.00 | 0.00 | 0.01 | 0.01 | 0.01 | 0.01 | 0.01 | 0.01 | 0.00 |  |
| 87 | 2H | 133.56 | 133.74 | 135.08 | **EL** | **S** | **6.11** | QTLxE | No | 0.80 | -0.35 | -0.07 | -0.13 | -0.46 | -0.37 | -0.45 | -0.44 | -0.48 | 0.12 |  |
| 88 | 2H | 138.91 | 139.63 | 146.38 | **G/E** | **S** | **4.22** | QTLxE | No | 0.95 | 0.00 | 0.00 | -0.84 | 1.21 | -0.52 | -0.95 | 1.63 | -0.34 | 0.61 |  |
| 89 | 2H | 145.94 | 145.99 | 146.38 | **GY** | **S** | **4.09** | QTLxE | No | 0.82 | 0.19 | 0.20 | 0.10 | 0.13 | -0.14 | 0.24 | 0.00 | -0.13 | 0.08 |  |
| 90 | 2H | 148.22 | 148.36 | 149.07 | **GL** | **W** | **4.19** | QTLxE | No | 0.75 | -0.11 | -0.06 | -0.23 | -0.18 | -0.24 | -0.23 | -0.22 | -0.16 | 0.06 | **2_5** |
| 91 | 2H | 149.35 | 151.41 | 155.42 | **GA** | **S** | **5.82** | QTLxE | No | 0.91 | 0.13 | 0.09 | 0.47 | 0.27 | 0.58 | 0.59 | 0.58 | 0.45 | 0.14 | **2_5** |
| 92 | 2H | 149.35 | 151.41 | 151.71 | **GL** | **S** | **11.00** | QTLxE | No | 0.91 | 0.06 | 0.05 | 0.17 | 0.14 | 0.19 | 0.28 | 0.20 | 0.16 | 0.04 | **2_5** |
| 93 | 2H | 149.35 | 151.41 | 156.86 | **GR** | **S** | **4.92** | QTLxE | No | 0.91 | 0.00 | -0.01 | 0.00 | -0.01 | -0.01 | -0.01 | -0.01 | -0.01 | 0.00 | **2_5** |
| 94 | 2H | 151.78 | 156.72 | 156.94 | **GR** | **W** | **4.28** | Main | No | 0.62 | -0.01 | -0.01 | -0.01 | -0.01 | -0.01 | -0.01 | -0.01 | -0.01 | 0.00 |  |
| 95 | 3H | 7.64 | 7.76 | 9.21 | **TGW** | **W** | **4.54** | QTLxE | No | 0.55 | -0.69 | 0.59 | -0.50 | -0.75 | -1.01 | -1.16 | -1.82 | -0.94 | 0.40 |  |
| 96 | 3H | 7.76 | 9.21 | 9.97 | **G/E** | **S** | **4.70** | QTLxE | No | 0.90 | 0.00 | 0.00 | 0.23 | 0.53 | 1.18 | 0.05 | 2.24 | 0.18 | 0.47 |  |
| 97 | 3H | 17.18 | 19.18 | 19.24 | **GW** | **S** | **4.14** | QTLxE | No | 0.16 | -0.05 | -0.03 | -0.04 | -0.01 | -0.03 | -0.01 | -0.04 | -0.03 | 0.01 |  |
| 98 | 3H | 23.87 | 24.35 | 35.15 | **GL** | **S** | **4.92** | QTLxE | No | 0.69 | -0.04 | -0.04 | -0.07 | -0.07 | -0.10 | -0.09 | -0.06 | -0.08 | 0.02 |  |
| 99 | 3H | 23.87 | 24.35 | 35.15 | **GR** | **S** | **4.48** | QTLxE | No | 0.69 | 0.00 | 0.00 | 0.00 | 0.00 | 0.01 | 0.00 | 0.00 | 0.00 | 0.00 |  |
| 100 | 3H | 25.66 | 26.46 | 26.70 | **EL** | **W** | **5.87** | QTLxE | No | 0.83 | -0.13 | -0.39 | -0.58 | -0.64 | -0.48 | 10.37 | -0.56 | -2.75 | 1.77 |  |
| 101 | 3H | 36.50 | 36.70 | 36.85 | **GA** | **S** | **4.66** | Main | No | 0.73 | -0.43 | -0.43 | -0.43 | -0.43 | -0.43 | -0.43 | -0.43 | -0.43 | 0.09 |  |
| 102 | 3H | 36.50 | 36.70 | 36.85 | **GL** | **S** | **6.79** | Main | No | 0.72 | -0.10 | -0.10 | -0.10 | -0.10 | -0.10 | -0.10 | -0.10 | -0.10 | 0.02 |  |
| 103 | 3H | 41.04 | 43.99 | 45.28 | **GL** | **W** | **4.75** | QTLxE | No | 0.80 | -0.07 | -0.10 | -0.30 | -0.26 | -0.23 | -0.23 | -0.21 | -0.17 | 0.07 |  |
| 104 | 3H | 53.48 | 53.50 | 53.54 | **EL** | **S** | **9.30** | QTLxE | No | 0.89 | 0.52 | 0.30 | 0.36 | 0.65 | 0.51 | 0.64 | 0.65 | 0.56 | 0.12 | **3_1** |
| 105 | 3H | 53.09 | 53.50 | 63.56 | **GA** | **S** | **4.17** | Main | No | 0.76 | 0.42 | 0.42 | 0.42 | 0.42 | 0.42 | 0.42 | 0.42 | 0.42 | 0.14 | **3_1** |
| 106 | 3H | 53.48 | 53.50 | 53.54 | **GL** | **S** | **9.87** | Main | No | 0.76 | 0.15 | 0.15 | 0.15 | 0.15 | 0.15 | 0.15 | 0.15 | 0.15 | 0.04 | **3_1** |
| 107 | 3H | 53.48 | 53.50 | 56.24 | **TGW** | **S** | **4.48** | Main | No | 0.80 | 1.67 | 1.67 | 1.67 | 1.67 | 1.67 | 1.67 | 1.67 | 1.67 | 0.39 | **3_1** |
| 108 | 3H | 52.59 | 53.50 | 53.54 | **GR** | **S** | **7.44** | Main | No | 0.76 | -0.01 | -0.01 | -0.01 | -0.01 | -0.01 | -0.01 | -0.01 | -0.01 | 0.00 | **3_1** |
| 109 | 3H | 53.48 | 53.50 | 56.24 | **GY** | **S** | **6.13** | QTLxE | Yes | 0.91 | -0.29 | 0.22 | 0.06 | -0.14 | -0.31 | 0.41 | -0.17 | -0.21 | 0.11 | **3_1** |
| 110 | 3H | 53.48 | 58.24 | 58.30 | **G/E** | **S** | **7.71** | QTLxE | Yes | 0.94 | 0.00 | 0.00 | -0.93 | 0.18 | 0.25 | -2.16 | 2.47 | 0.91 | 0.66 | **3_1** |
| 111 | 3H | 61.10 | 61.59 | 62.34 | **GR** | **W** | **6.90** | QTLxE | No | 0.61 | -0.01 | 0.00 | -0.01 | -0.01 | -0.01 | -0.01 | -0.01 | -0.01 | 0.00 |  |
| 112 | 3H | 61.10 | 64.27 | 64.30 | **GY** | **W** | **7.55** | QTLxE | No | 0.81 | 0.17 | 0.60 | 0.50 | 0.18 | 0.57 | 0.06 | 0.50 | 0.50 | 0.14 |  |
| 113 | 3H | 61.10 | 64.46 | 69.71 | **GL** | **W** | **4.78** | QTLxE | No | 0.43 | -0.07 | -0.03 | -0.18 | -0.19 | -0.20 | -0.21 | -0.19 | -0.17 | 0.05 | **3_2** |
| 114 | 3H | 65.72 | 66.28 | 74.86 | **GA** | **W** | **5.92** | Main | No | 0.31 | -0.67 | -0.67 | -0.67 | -0.67 | -0.67 | -0.67 | -0.67 | -0.67 | 0.18 | **3_2** |
| 115 | 3H | 65.72 | 66.28 | 72.53 | **TGW** | **W** | **5.49** | Main | No | 0.31 | -1.61 | -1.61 | -1.61 | -1.61 | -1.61 | -1.61 | -1.61 | -1.61 | 0.46 | **3_2** |
| 116 | 3H | 66.57 | 66.67 | 66.91 | **GA** | **S** | **5.41** | Main | No | 0.36 | -0.64 | -0.64 | -0.64 | -0.64 | -0.64 | -0.64 | -0.64 | -0.64 | 0.14 | **3_2** |
| 117 | 3H | 66.57 | 66.67 | 66.91 | **GL** | **S** | **7.99** | Main | No | 0.36 | -0.19 | -0.19 | -0.19 | -0.19 | -0.19 | -0.19 | -0.19 | -0.19 | 0.04 | **3_2** |
| 118 | 3H | 74.86 | 75.26 | 75.79 | **EL** | **S** | **4.83** | Main | No | 0.87 | 0.54 | 0.54 | 0.54 | 0.54 | 0.54 | 0.54 | 0.54 | 0.54 | 0.16 |  |
| 119 | 3H | 78.64 | 78.81 | 80.08 | **GL** | **S** | **5.56** | Main | No | 0.86 | 0.12 | 0.12 | 0.12 | 0.12 | 0.12 | 0.12 | 0.12 | 0.12 | 0.04 |  |
| 120 | 3H | 94.54 | 96.92 | 97.37 | **GW** | **S** | **4.97** | QTLxE | No | 0.88 | 0.01 | 0.00 | 0.06 | 0.02 | 0.03 | -0.02 | 0.02 | 0.01 | 0.01 | **3_3** |
| 121 | 3H | 90.41 | 97.37 | 97.42 | **GA** | **W** | **4.21** | QTLxE | No | 0.59 | 0.35 | 0.24 | 0.46 | 0.35 | 0.98 | 0.73 | 0.50 | 0.45 | 0.18 | **3_3** |
| 122 | 3H | 97.37 | 97.42 | 97.86 | **GL** | **W** | **9.27** | QTLxE | No | 0.50 | -0.05 | -0.04 | -0.14 | -0.16 | -0.23 | -0.32 | -0.18 | -0.14 | 0.05 | **3_3** |
| 123 | 3H | 97.37 | 97.42 | 97.86 | **GR** | **W** | **7.66** | QTLxE | No | 0.50 | 0.00 | 0.00 | 0.01 | 0.01 | 0.01 | 0.01 | 0.01 | 0.01 | 0.00 | **3_3** |
| 124 | 3H | 97.42 | 97.86 | 99.96 | **GR** | **S** | **4.62** | QTLxE | No | 0.19 | -0.01 | -0.01 | 0.00 | -0.01 | -0.01 | -0.01 | 0.00 | 0.00 | 0.00 | **3_3** |
| 125 | 3H | 99.96 | 100.07 | 100.50 | **G/E** | **S** | **6.79** | QTLxE | No | 0.87 | 0.00 | 0.00 | -2.13 | -1.42 | -0.83 | -0.27 | -1.85 | -2.36 | 0.48 |  |
| 126 | 3H | 119.45 | 120.55 | 120.94 | **GR** | **S** | **4.79** | Main | No | 0.69 | 0.00 | 0.00 | 0.00 | 0.00 | 0.00 | 0.00 | 0.00 | 0.00 | 0.00 |  |
| 127 | 3H | 121.19 | 124.58 | 124.62 | **G/E** | **S** | **4.81** | QTLxE | Yes | 0.94 | 0.00 | 0.00 | 0.57 | 0.12 | 0.33 | 1.49 | -2.58 | -0.93 | 0.71 | **3_4** |
| 128 | 3H | 121.76 | 124.62 | 126.69 | **EL** | **S** | **4.19** | Main | No | 0.71 | -0.32 | -0.32 | -0.32 | -0.32 | -0.32 | -0.32 | -0.32 | -0.32 | 0.07 | **3_4** |
| 129 | 3H | 124.58 | 124.62 | 126.69 | **GA** | **S** | **7.89** | QTLxE | No | 0.71 | -0.45 | -0.30 | -0.23 | -0.37 | -0.42 | -0.64 | -0.40 | -0.45 | 0.09 | **3_4** |
| 130 | 3H | 124.58 | 124.62 | 126.69 | **TGW** | **S** | **5.51** | QTLxE | No | 0.71 | -0.77 | -0.26 | 0.05 | -0.40 | -0.55 | -1.06 | -0.72 | -1.03 | 0.25 | **3_4** |
| 131 | 3H | 130.49 | 131.33 | 131.34 | **GL** | **S** | **6.92** | QTLxE | No | 0.93 | -0.04 | 0.01 | 0.09 | 0.05 | 0.18 | 0.24 | 0.13 | 0.08 | 0.05 |  |
| 132 | 3H | 136.28 | 136.37 | 137.02 | **EL** | **S** | **4.55** | QTLxE | No | 0.55 | 0.19 | 0.12 | 0.09 | 0.28 | 0.18 | 0.25 | 0.16 | 0.09 | 0.07 |  |
| 133 | 3H | 141.32 | 141.62 | 141.92 | **GL** | **W** | **4.38** | Main | No | 0.79 | -0.12 | -0.12 | -0.12 | -0.12 | -0.12 | -0.12 | -0.12 | -0.12 | 0.06 |  |
| 134 | 3H | 149.16 | 149.93 | 151.60 | **GW** | **S** | **5.18** | QTLxE | No | 0.81 | 0.03 | 0.00 | 0.06 | 0.03 | 0.03 | 0.02 | 0.04 | 0.04 | 0.01 |  |
| 135 | 3H | 149.16 | 149.93 | 150.59 | **GY** | **W** | **9.78** | QTLxE | No | 0.65 | 0.43 | 0.69 | 0.42 | 0.32 | 0.49 | -0.02 | 0.38 | 0.29 | 0.12 |  |
| 136 | 4H | 0.40 | 0.70 | 7.71 | **GA** | **S** | **4.55** | Main | No | 0.78 | 0.43 | 0.43 | 0.43 | 0.43 | 0.43 | 0.43 | 0.43 | 0.43 | 0.11 | **4_1** |
| 137 | 4H | 0.40 | 0.70 | 7.71 | **GL** | **S** | **5.43** | Main | No | 0.78 | 0.10 | 0.10 | 0.10 | 0.10 | 0.10 | 0.10 | 0.10 | 0.10 | 0.03 | **4_1** |
| 138 | 4H | 6.41 | 6.66 | 7.71 | **GL** | **W** | **4.37** | QTLxE | No | 0.32 | 0.11 | 0.09 | 0.26 | 0.21 | 0.13 | 0.15 | 0.23 | 0.16 | 0.06 | **4_1** |
| 139 | 4H | 6.41 | 6.66 | 7.71 | **GR** | **W** | **5.44** | QTLxE | No | 0.32 | 0.00 | 0.00 | -0.01 | -0.01 | -0.01 | 0.00 | -0.01 | -0.01 | 0.00 | **4_1** |
| 140 | 4H | 22.12 | 22.86 | 23.61 | **GA** | **S** | **11.40** | QTLxE | No | 0.83 | -1.19 | -1.51 | -1.76 | -1.63 | -1.97 | -2.46 | -1.19 | -1.44 | 0.31 | **4_2** |
| 141 | 4H | 22.12 | 22.86 | 23.61 | **GW** | **S** | **16.13** | Main | No | 0.83 | -0.16 | -0.16 | -0.16 | -0.16 | -0.16 | -0.16 | -0.16 | -0.16 | 0.03 | **4_2** |
| 142 | 4H | 22.12 | 22.86 | 23.61 | **G/E** | **S** | **51.27** | Main | No | 0.83 | 0.00 | 0.00 | 12.32 | 12.32 | 12.32 | 12.32 | 12.32 | 12.32 | 1.03 | **4_2** |
| 143 | 4H | 22.12 | 22.86 | 23.61 | **TGW** | **S** | **15.89** | QTLxE | No | 0.83 | -3.93 | -3.82 | -6.47 | -4.64 | -5.24 | -6.18 | -5.30 | -5.01 | 0.81 | **4_2** |
| 144 | 4H | 22.12 | 22.86 | 23.61 | **GR** | **S** | **7.15** | QTLxE | No | 0.83 | -0.01 | -0.02 | -0.03 | -0.02 | -0.01 | -0.01 | -0.02 | -0.02 | 0.00 | **4_2** |
| 145 | 4H | 21.57 | 22.86 | 24.70 | **GW** | **W** | **14.83** | QTLxE | No | 0.50 | 0.17 | 0.08 | 0.24 | 0.15 | 0.27 | 0.25 | 0.22 | 0.16 | 0.04 | **4_2** |
| 146 | 4H | 21.57 | 22.86 | 23.61 | **G/E** | **W** | **15.06** | QTLxE | No | 0.50 |  |  | -10.61 | -9.51 | -6.89 | -11.53 | -7.29 | -4.57 | 1.66 | **4_2** |
| 147 | 4H | 23.61 | 24.20 | 24.70 | **GA** | **W** | **18.13** | QTLxE | No | 0.53 | -1.88 | -1.03 | -2.66 | -2.73 | -2.98 | -2.52 | -2.71 | -2.07 | 0.40 | **4_2** |
| 148 | 4H | 23.61 | 24.20 | 24.70 | **TGW** | **W** | **17.16** | QTLxE | No | 0.53 | -4.39 | -3.66 | -7.64 | -6.55 | -7.22 | -6.65 | -7.50 | -5.83 | 1.11 | **4_2** |
| 149 | 4H | 26.89 | 28.78 | 29.16 | **GL** | **S** | **9.80** | QTLxE | No | 0.95 | 0.01 | 0.02 | 0.25 | 0.15 | 0.16 | 0.18 | 0.18 | 0.14 | 0.05 |  |
| 150 | 4H | 35.50 | 36.60 | 37.26 | **G/E** | **S** | **10.13** | QTLxE | No | 0.93 | 0.00 | 0.00 | 2.60 | 0.67 | 2.62 | 0.03 | 3.74 | 3.42 | 0.69 |  |
| 151 | 4H | 37.26 | 37.92 | 38.58 | **GR** | **W** | **5.42** | Main | No | 0.82 | 0.01 | 0.01 | 0.01 | 0.01 | 0.01 | 0.01 | 0.01 | 0.01 | 0.00 |  |
| 152 | 4H | 38.58 | 39.60 | 39.90 | **GL** | **S** | **8.11** | QTLxE | No | 0.86 | -0.13 | -0.18 | -0.25 | -0.26 | -0.25 | -0.26 | -0.22 | -0.17 | 0.05 |  |
| 153 | 4H | 38.58 | 39.60 | 39.90 | **GY** | **S** | **4.28** | QTLxE | No | 0.82 | -0.12 | -0.15 | -0.19 | -0.36 | -0.10 | -0.01 | 0.12 | 0.14 | 0.10 |  |
| 154 | 4H | 48.34 | 48.72 | 50.27 | **GR** | **S** | **7.00** | QTLxE | No | 0.93 | 0.00 | 0.01 | 0.00 | 0.01 | 0.01 | 0.01 | 0.01 | 0.01 | 0.00 | **4_3** |
| 155 | 4H | 48.34 | 48.72 | 50.58 | **GL** | **W** | **5.57** | QTLxE | No | 0.85 | 0.05 | 0.16 | 0.45 | 0.32 | 0.23 | 0.28 | 0.34 | 0.35 | 0.10 | **4_3** |
| 156 | 4H | 49.38 | 49.41 | 49.57 | **TGW** | **S** | **8.74** | QTLxE | No | 0.77 | -0.15 | -0.66 | -0.75 | -1.25 | -1.39 | -1.48 | -1.17 | -1.00 | 0.28 | **4_3** |
| 157 | 4H | 49.20 | 49.41 | 50.96 | **GW** | **W** | **6.46** | Main | No | 0.83 | -0.13 | -0.13 | -0.13 | -0.13 | -0.13 | -0.13 | -0.13 | -0.13 | 0.03 | **4_3** |
| 158 | 4H | 49.57 | 50.58 | 50.68 | **GA** | **W** | **5.54** | Main | No | 0.37 | 0.45 | 0.45 | 0.45 | 0.45 | 0.45 | 0.45 | 0.45 | 0.45 | 0.15 | **4_3** |
| 159 | 4H | 49.57 | 50.68 | 50.96 | **TGW** | **W** | **4.73** | Main | No | 0.63 | 1.72 | 1.72 | 1.72 | 1.72 | 1.72 | 1.72 | 1.72 | 1.72 | 0.36 | **4_3** |
| 160 | 4H | 50.82 | 50.96 | 50.97 | **GA** | **S** | **8.38** | Main | No | 0.76 | -0.55 | -0.55 | -0.55 | -0.55 | -0.55 | -0.55 | -0.55 | -0.55 | 0.13 | **4_4** |
| 161 | 4H | 50.82 | 50.96 | 50.97 | **GL** | **S** | **9.28** | Main | No | 0.76 | -0.17 | -0.17 | -0.17 | -0.17 | -0.17 | -0.17 | -0.17 | -0.17 | 0.04 | **4_4** |
| 162 | 4H | 50.82 | 50.96 | 50.97 | **G/E** | **S** | **8.08** | QTLxE | No | 0.92 | 0.00 | 0.00 | 1.78 | -0.56 | 2.72 | -0.68 | 3.12 | 1.72 | 0.76 | **4_4** |
| 163 | 4H | 50.82 | 50.96 | 54.28 | **GR** | **W** | **5.16** | QTLxE | No | 0.77 | -0.01 | 0.00 | -0.02 | -0.01 | -0.01 | -0.01 | -0.01 | -0.01 | 0.00 | **4_4** |
| 164 | 4H | 51.12 | 51.30 | 51.61 | **EL** | **S** | **5.42** | QTLxE | No | 0.87 | 0.22 | 0.27 | -0.43 | -0.63 | -0.45 | -0.25 | -0.61 | -0.28 | 0.23 |  |
| 165 | 4H | 51.61 | 51.90 | 56.58 | **GW** | **S** | **5.55** | QTLxE | No | 0.86 | -0.01 | -0.02 | -0.04 | -0.04 | -0.06 | -0.06 | -0.03 | -0.03 | 0.01 |  |
| 166 | 4H | 51.61 | 61.04 | 62.43 | **GR** | **S** | **4.65** | Main | No | 0.32 | -0.01 | -0.01 | -0.01 | -0.01 | -0.01 | -0.01 | -0.01 | -0.01 | 0.00 |  |
| 167 | 4H | 55.00 | 61.46 | 62.43 | **GA** | **S** | **4.88** | Main | No | 0.38 | 0.42 | 0.42 | 0.42 | 0.42 | 0.42 | 0.42 | 0.42 | 0.42 | 0.15 |  |
| 168 | 4H | 61.27 | 61.46 | 62.43 | **GL** | **S** | **8.68** | QTLxE | No | 0.38 | 0.12 | 0.15 | 0.24 | 0.18 | 0.28 | 0.21 | 0.20 | 0.20 | 0.04 |  |
| 169 | 4H | 67.75 | 68.41 | 75.99 | **EL** | **S** | **4.21** | QTLxE | No | 0.83 | -0.04 | 0.05 | -0.30 | -0.27 | -0.16 | -0.24 | -0.22 | 0.04 | 0.10 | **4_5** |
| 170 | 4H | 70.92 | 72.17 | 72.82 | **GA** | **S** | **5.65** | Main | No | 0.81 | -0.41 | -0.41 | -0.41 | -0.41 | -0.41 | -0.41 | -0.41 | -0.41 | 0.09 | **4_5** |
| 171 | 4H | 70.92 | 72.17 | 72.82 | **TGW** | **S** | **8.08** | QTLxE | No | 0.81 | -0.23 | -0.55 | -0.82 | -0.88 | -1.28 | -1.43 | -1.06 | -1.15 | 0.26 | **4_5** |
| 172 | 4H | 72.17 | 72.82 | 73.31 | **GA** | **W** | **4.13** | QTLxE | No | 0.55 | -0.14 | 0.06 | -0.47 | -0.45 | -0.54 | -0.45 | -0.37 | -0.39 | 0.15 | **4_5** |
| 173 | 4H | 72.17 | 72.82 | 73.31 | **GW** | **W** | **5.77** | QTLxE | Yes | 0.55 | 0.01 | 0.04 | -0.03 | -0.03 | -0.02 | -0.01 | -0.03 | -0.03 | 0.01 | **4_5** |
| 174 | 4H | 72.17 | 72.82 | 73.31 | **TGW** | **W** | **7.91** | QTLxE | Yes | 0.55 | 0.09 | 0.99 | -1.47 | -1.33 | -1.04 | -0.99 | -1.15 | -1.08 | 0.41 | **4_5** |
| 175 | 4H | 75.15 | 75.38 | 75.68 | **GW** | **S** | **5.25** | QTLxE | No | 0.92 | -0.02 | -0.06 | -0.08 | -0.06 | -0.04 | -0.04 | -0.05 | -0.07 | 0.02 |  |
| 176 | 4H | 75.15 | 75.38 | 75.68 | **G/E** | **S** | **7.59** | QTLxE | No | 0.92 | 0.00 | 0.00 | 1.14 | 0.70 | 2.13 | 0.44 | 2.36 | 2.91 | 0.55 |  |
| 177 | 4H | 75.15 | 82.55 | 85.22 | **GA** | **S** | **5.18** | QTLxE | No | 0.69 | 0.11 | 0.19 | 0.29 | 0.29 | 0.29 | 0.51 | 0.36 | 0.21 | 0.09 |  |
| 178 | 4H | 81.77 | 86.37 | 86.56 | **GL** | **S** | **6.41** | Main | No | 0.37 | -0.06 | -0.06 | -0.06 | -0.06 | -0.06 | -0.06 | -0.06 | -0.06 | 0.02 |  |
| 179 | 4H | 85.81 | 86.37 | 86.56 | **GR** | **S** | **5.81** | Main | No | 0.37 | 0.00 | 0.00 | 0.00 | 0.00 | 0.00 | 0.00 | 0.00 | 0.00 | 0.00 |  |
| 180 | 4H | 94.24 | 97.31 | 98.50 | **EL** | **S** | **4.33** | Main | No | 0.57 | -0.19 | -0.19 | -0.19 | -0.19 | -0.19 | -0.19 | -0.19 | -0.19 | 0.07 |  |
| 181 | 4H | 112.03 | 112.65 | 113.40 | **GA** | **S** | **8.30** | Main | No | 0.52 | 0.36 | 0.36 | 0.36 | 0.36 | 0.36 | 0.36 | 0.36 | 0.36 | 0.08 |  |
| 182 | 4H | 112.03 | 112.65 | 113.40 | **GL** | **S** | **8.81** | Main | No | 0.52 | 0.10 | 0.10 | 0.10 | 0.10 | 0.10 | 0.10 | 0.10 | 0.10 | 0.02 |  |
| 183 | 5H | 1.07 | 1.17 | 1.28 | **G/E** | **S** | **12.64** | QTLxE | No | 0.90 | 0.00 | 0.00 | 1.44 | 1.77 | 1.76 | 1.79 | 4.65 | 2.28 | 0.56 |  |
| 184 | 5H | 2.48 | 3.38 | 8.17 | **GW** | **W** | **4.22** | Main | No | 0.80 | -0.06 | -0.06 | -0.06 | -0.06 | -0.06 | -0.06 | -0.06 | -0.06 | 0.02 |  |
| 185 | 5H | 2.48 | 3.38 | 8.17 | **TGW** | **W** | **4.84** | Main | No | 0.80 | -1.33 | -1.33 | -1.33 | -1.33 | -1.33 | -1.33 | -1.33 | -1.33 | 0.38 |  |
| 186 | 5H | 26.63 | 27.08 | 27.85 | **GA** | **W** | **7.88** | QTLxE | No | 0.87 | 0.88 | 0.16 | 0.64 | 0.91 | 1.16 | 1.27 | 0.68 | 0.83 | 0.22 |  |
| 187 | 5H | 26.63 | 27.08 | 30.28 | **GW** | **W** | **4.56** | Main | No | 0.87 | 0.08 | 0.08 | 0.08 | 0.08 | 0.08 | 0.08 | 0.08 | 0.08 | 0.02 |  |
| 188 | 5H | 26.63 | 27.08 | 27.85 | **TGW** | **W** | **9.66** | QTLxE | No | 0.87 | 2.64 | 0.48 | 2.31 | 2.76 | 3.19 | 2.95 | 3.10 | 2.49 | 0.61 |  |
| 189 | 5H | 27.08 | 27.85 | 27.93 | **G/E** | **S** | **5.77** | QTLxE | No | 0.62 | 0.00 | 0.00 | -1.63 | -1.01 | -1.90 | -0.57 | -1.46 | -0.94 | 0.39 |  |
| 190 | 5H | 21.49 | 29.45 | 29.46 | **GL** | **W** | **4.71** | QTLxE | No | 0.82 | 0.11 | 0.08 | 0.24 | 0.23 | 0.29 | 0.27 | 0.24 | 0.17 | 0.07 |  |
| 191 | 5H | 33.45 | 33.96 | 35.51 | **GA** | **S** | **8.55** | QTLxE | No | 0.88 | -0.60 | -0.40 | -0.79 | -0.68 | -0.81 | -0.80 | -0.87 | -0.73 | 0.15 | **5_1** |
| 192 | 5H | 33.45 | 33.96 | 35.51 | **GL** | **S** | **13.25** | QTLxE | No | 0.88 | -0.17 | -0.13 | -0.28 | -0.22 | -0.24 | -0.24 | -0.26 | -0.23 | 0.04 | **5_1** |
| 193 | 5H | 33.45 | 33.96 | 35.51 | **TGW** | **S** | **5.03** | Main | No | 0.88 | -1.28 | -1.28 | -1.28 | -1.28 | -1.28 | -1.28 | -1.28 | -1.28 | 0.43 | **5_1** |
| 194 | 5H | 33.45 | 33.96 | 42.44 | **GR** | **S** | **4.23** | Main | No | 0.88 | 0.01 | 0.01 | 0.01 | 0.01 | 0.01 | 0.01 | 0.01 | 0.01 | 0.00 | **5_1; 5_2** |
| 195 | 5H | 39.88 | 39.91 | 43.87 | **GA** | **W** | **5.12** | QTLxE | No | 0.86 | 0.75 | 0.36 | 1.15 | 0.92 | 1.17 | 0.84 | 1.43 | 1.04 | 0.30 | **5_2** |
| 196 | 5H | 39.88 | 39.91 | 41.17 | **GL** | **W** | **7.25** | Main | No | 0.86 | 0.30 | 0.30 | 0.30 | 0.30 | 0.30 | 0.30 | 0.30 | 0.30 | 0.09 |  |
| 197 | 5H | 42.44 | 43.73 | 43.87 | **GW** | **W** | **8.11** | Main | No | 0.86 | -0.12 | -0.12 | -0.12 | -0.12 | -0.12 | -0.12 | -0.12 | -0.12 | 0.02 | **5_2** |
| 198 | 5H | 42.44 | 43.73 | 43.87 | **G/E** | **W** | **4.52** | Main | No | 0.86 |  |  | 4.21 | 4.21 | 4.21 | 4.21 | 4.21 | 4.21 | 0.89 | **5_2** |
| 199 | 5H | 42.44 | 43.73 | 43.87 | **TGW** | **W** | **9.53** | Main | No | 0.86 | -3.14 | -3.14 | -3.14 | -3.14 | -3.14 | -3.14 | -3.14 | -3.14 | 0.54 | **5_2** |
| 200 | 5H | 44.83 | 45.22 | 46.74 | **GA** | **S** | **9.95** | QTLxE | No | 0.09 | -0.57 | -0.73 | -1.18 | -0.91 | -1.41 | -1.08 | -1.15 | -0.98 | 0.22 |  |
| 201 | 5H | 45.81 | 46.01 | 47.26 | **GR** | **W** | **5.66** | QTLxE | No | 0.34 | 0.00 | 0.00 | 0.01 | 0.01 | 0.01 | 0.01 | 0.01 | 0.01 | 0.00 |  |
| 202 | 5H | 44.83 | 48.16 | 48.59 | **TGW** | **S** | **4.79** | Main | No | 0.12 | -1.62 | -1.62 | -1.62 | -1.62 | -1.62 | -1.62 | -1.62 | -1.62 | 0.44 |  |
| 203 | 5H | 47.72 | 48.16 | 48.59 | **GL** | **S** | **11.94** | QTLxE | No | 0.12 | -0.11 | -0.11 | -0.25 | -0.19 | -0.31 | -0.22 | -0.21 | -0.19 | 0.04 |  |
| 204 | 5H | 49.88 | 50.37 | 51.54 | **G/E** | **S** | **8.84** | QTLxE | No | 0.23 | 0.00 | 0.00 | -2.05 | -0.75 | -2.14 | -1.36 | -1.24 | -1.01 | 0.38 |  |
| 205 | 5H | 51.86 | 61.61 | 63.21 | **G/E** | **S** | **4.06** | QTLxE | No | 0.95 | 0.00 | 0.00 | 1.17 | 1.74 | 0.41 | -0.03 | 2.01 | 2.04 | 0.58 |  |
| 206 | 5H | 73.88 | 73.98 | 74.16 | **GA** | **S** | **6.17** | QTLxE | No | 0.87 | 0.01 | -0.45 | -0.50 | -0.55 | -0.56 | -1.02 | -0.53 | -0.61 | 0.18 |  |
| 207 | 5H | 75.06 | 75.38 | 75.50 | **GL** | **S** | **7.89** | QTLxE | No | 0.73 | 0.09 | 0.06 | 0.14 | 0.10 | 0.18 | 0.11 | 0.09 | 0.08 | 0.03 |  |
| 208 | 5H | 75.50 | 78.26 | 79.07 | **EL** | **S** | **6.74** | QTLxE | No | 0.88 | -0.41 | 0.04 | -0.51 | -0.69 | -0.67 | -0.68 | -0.52 | -0.23 | 0.19 |  |
| 209 | 5H | 81.46 | 81.68 | 89.02 | **TGW** | **S** | **6.37** | QTLxE | No | 0.44 | 0.31 | 0.22 | 0.84 | 0.87 | 0.96 | 0.95 | 0.92 | 1.04 | 0.24 |  |
| 210 | 5H | 95.39 | 95.49 | 99.06 | **GL** | **S** | **5.83** | QTLxE | No | 0.82 | -0.03 | -0.02 | -0.13 | -0.08 | -0.15 | -0.12 | -0.12 | -0.09 | 0.03 | **5_3** |
| 211 | 5H | 97.69 | 97.86 | 99.01 | **GW** | **S** | **6.16** | Main | No | 0.89 | -0.05 | -0.05 | -0.05 | -0.05 | -0.05 | -0.05 | -0.05 | -0.05 | 0.02 | **5_3** |
| 212 | 5H | 97.69 | 97.86 | 99.01 | **G/E** | **S** | **6.63** | QTLxE | No | 0.89 | 0.00 | 0.00 | 0.81 | 3.03 | 0.82 | 1.76 | 2.28 | 2.57 | 0.63 | **5_3** |
| 213 | 5H | 92.59 | 99.01 | 99.06 | **GR** | **S** | **5.23** | QTLxE | No | 0.83 | 0.00 | 0.00 | 0.01 | 0.01 | 0.01 | 0.01 | 0.01 | 0.01 | 0.00 | **5_3** |
| 214 | 5H | 97.69 | 106.16 | 114.67 | **EL** | **S** | **4.25** | Main | No | 0.83 | -0.51 | -0.51 | -0.51 | -0.51 | -0.51 | -0.51 | -0.51 | -0.51 | 0.11 | **5_3;5_4** |
| 215 | 5H | 110.96 | 111.25 | 114.09 | **GA** | **S** | **4.73** | Main | No | 0.75 | 0.57 | 0.57 | 0.57 | 0.57 | 0.57 | 0.57 | 0.57 | 0.57 | 0.12 | **5_4** |
| 216 | 5H | 110.96 | 111.25 | 118.33 | **TGW** | **S** | **4.29** | Main | No | 0.80 | 1.66 | 1.66 | 1.66 | 1.66 | 1.66 | 1.66 | 1.66 | 1.66 | 0.38 | **5_4** |
| 217 | 5H | 110.61 | 111.25 | 111.91 | **GY** | **W** | **4.72** | QTLxE | No | 0.74 | -0.01 | -0.25 | -0.51 | -0.27 | -0.46 | -0.09 | -0.32 | -0.33 | 0.13 |  |
| 218 | 5H | 113.36 | 113.81 | 114.67 | **GW** | **S** | **4.97** | Main | No | 0.67 | -0.04 | -0.04 | -0.04 | -0.04 | -0.04 | -0.04 | -0.04 | -0.04 | 0.01 | **5_4** |
| 219 | 5H | 118.61 | 118.85 | 119.02 | **GR** | **S** | **5.34** | QTLxE | No | 0.58 | 0.01 | 0.01 | 0.01 | 0.01 | 0.00 | 0.00 | 0.00 | 0.00 | 0.00 |  |
| 220 | 5H | 119.20 | 119.30 | 119.80 | **GR** | **W** | **4.55** | QTLxE | No | 0.54 | -0.01 | 0.00 | -0.01 | -0.01 | -0.01 | -0.01 | 0.00 | 0.00 | 0.00 |  |
| 221 | 5H | 129.21 | 129.47 | 129.72 | **GA** | **S** | **6.43** | QTLxE | No | 0.60 | -0.43 | -0.25 | -0.45 | -0.43 | -0.39 | -0.15 | -0.58 | -0.54 | 0.12 |  |
| 222 | 5H | 129.21 | 129.47 | 129.72 | **GL** | **S** | **7.03** | Main | No | 0.60 | -0.13 | -0.13 | -0.13 | -0.13 | -0.13 | -0.13 | -0.13 | -0.13 | 0.03 |  |
| 223 | 5H | 130.17 | 133.95 | 134.18 | **GR** | **S** | **6.84** | QTLxE | No | 0.57 | 0.00 | 0.01 | 0.01 | 0.01 | 0.01 | 0.01 | 0.00 | 0.00 | 0.00 |  |
| 224 | 5H | 135.48 | 137.07 | 141.79 | **GA** | **W** | **6.01** | QTLxE | No | 0.75 | 0.63 | 0.05 | 0.34 | 0.50 | 1.00 | 0.80 | 0.50 | 0.47 | 0.18 | **5_5** |
| 225 | 5H | 135.48 | 137.07 | 141.79 | **TGW** | **W** | **4.48** | QTLxE | No | 0.75 | 1.94 | 0.32 | 0.75 | 1.05 | 2.07 | 1.65 | 1.02 | 1.23 | 0.50 | **5_5** |
| 226 | 5H | 139.54 | 139.87 | 140.93 | **GY** | **S** | **5.45** | QTLxE | No | 0.27 | 0.14 | 0.44 | -0.07 | 0.20 | 0.10 | 0.04 | 0.01 | 0.11 | 0.09 | **5_5** |
| 227 | 5H | 135.48 | 141.65 | 141.79 | **G/E** | **W** | **4.42** | Main | No | 0.75 |  |  | -3.14 | -3.14 | -3.14 | -3.14 | -3.14 | -3.14 | 0.71 | **5_5** |
| 228 | 5H | 138.61 | 141.79 | 141.99 | **EL** | **S** | **4.94** | QTLxE | No | 0.50 | 0.17 | 0.14 | 0.41 | 0.32 | 0.28 | 0.22 | 0.31 | 0.17 | 0.08 | **5_5** |
| 229 | 5H | 133.77 | 141.79 | 141.99 | **GL** | **S** | **5.09** | QTLxE | No | 0.50 | 0.05 | 0.06 | 0.11 | 0.08 | 0.12 | 0.12 | 0.11 | 0.09 | 0.03 | **5_5** |
| 230 | 5H | 144.09 | 144.62 | 144.81 | **GY** | **W** | **5.69** | QTLxE | No | 0.89 | -0.06 | -0.05 | -0.67 | -0.41 | -0.29 | 0.31 | -0.44 | -0.33 | 0.17 |  |
| 231 | 5H | 145.24 | 145.74 | 145.85 | **G/E** | **S** | **4.20** | Main | No | 0.45 | 0.00 | 0.00 | -1.63 | -1.63 | -1.63 | -1.63 | -1.63 | -1.63 | 0.36 |  |
| 232 | 5H | 155.14 | 155.35 | 163.75 | **GA** | **S** | **5.23** | QTLxE | No | 0.80 | 0.24 | 0.33 | 0.42 | 0.49 | 0.69 | 0.59 | 0.49 | 0.49 | 0.13 | **5_6** |
| 233 | 5H | 155.14 | 155.35 | 155.52 | **GL** | **S** | **10.54** | QTLxE | No | 0.80 | 0.11 | 0.10 | 0.17 | 0.17 | 0.22 | 0.23 | 0.19 | 0.18 | 0.04 | **5_6** |
| 234 | 5H | 155.14 | 155.35 | 155.52 | **GR** | **S** | **6.33** | QTLxE | No | 0.80 | -0.01 | -0.01 | -0.01 | -0.01 | -0.01 | -0.01 | -0.01 | -0.01 | 0.00 | **5_6** |
| 235 | 5H | 155.14 | 155.35 | 155.52 | **GA** | **W** | **5.92** | QTLxE | No | 0.89 | 0.86 | 0.33 | 0.95 | 1.07 | 1.42 | 1.03 | 0.89 | 0.66 | 0.25 | **5_6** |
| 236 | 5H | 155.14 | 155.35 | 156.98 | **GW** | **W** | **6.97** | Main | No | 0.89 | 0.11 | 0.11 | 0.11 | 0.11 | 0.11 | 0.11 | 0.11 | 0.11 | 0.02 | **5_6** |
| 237 | 5H | 155.14 | 155.35 | 155.52 | **TGW** | **W** | **5.64** | Main | No | 0.89 | 2.49 | 2.49 | 2.49 | 2.49 | 2.49 | 2.49 | 2.49 | 2.49 | 0.58 | **5_6** |
| 238 | 5H | 158.25 | 158.72 | 159.30 | **G/E** | **S** | **9.76** | QTLxE | No | 0.94 | 0.00 | 0.00 | -2.25 | -1.21 | -0.88 | -0.31 | -0.38 | -4.72 | 0.75 |  |
| 239 | 5H | 162.41 | 162.89 | 169.62 | **GW** | **S** | **4.46** | Main | No | 0.38 | 0.04 | 0.04 | 0.04 | 0.04 | 0.04 | 0.04 | 0.04 | 0.04 | 0.01 | **5_7** |
| 240 | 5H | 161.16 | 166.06 | 166.86 | **GR** | **S** | **4.73** | Main | No | 0.48 | 0.00 | 0.00 | 0.00 | 0.00 | 0.00 | 0.00 | 0.00 | 0.00 | 0.00 |  |
| 241 | 5H | 167.06 | 167.48 | 169.43 | **GL** | **S** | **7.04** | QTLxE | No | 0.59 | 0.05 | 0.05 | 0.10 | 0.06 | 0.13 | 0.11 | 0.13 | 0.08 | 0.03 | **5_7** |
| 242 | 5H | 163.08 | 167.48 | 169.43 | **GW** | **W** | **5.89** | Main | No | 0.58 | -0.07 | -0.07 | -0.07 | -0.07 | -0.07 | -0.07 | -0.07 | -0.07 | 0.02 | **5_7** |
| 243 | 5H | 167.58 | 169.43 | 172.32 | **GA** | **S** | **8.38** | QTLxE | No | 0.59 | -0.45 | -0.22 | -0.47 | -0.41 | -0.51 | -0.48 | -0.34 | -0.27 | 0.09 | **5_7** |
| 244 | 5H | 167.58 | 169.43 | 172.32 | **TGW** | **S** | **5.79** | QTLxE | No | 0.59 | -1.08 | -0.43 | -1.16 | -1.07 | -1.01 | -0.84 | -0.75 | -0.56 | 0.24 | **5_7** |
| 245 | 5H | 172.58 | 172.72 | 174.99 | **G/E** | **S** | **6.53** | QTLxE | No | 0.93 | 0.00 | 0.00 | -1.00 | -1.05 | -0.30 | -3.29 | -1.12 | -3.32 | 0.71 | **5_7** |
| 246 | 5H | 174.25 | 174.39 | 174.49 | **GW** | **S** | **5.06** | QTLxE | No | 0.82 | 0.06 | 0.05 | 0.09 | 0.07 | 0.06 | 0.05 | 0.04 | 0.03 | 0.02 |  |
| 247 | 6H | 2.18 | 10.30 | 10.50 | **GR** | **S** | **4.31** | QTLxE | No | 0.86 | 0.00 | -0.01 | -0.01 | -0.01 | 0.00 | -0.01 | -0.01 | -0.01 | 0.00 |  |
| 248 | 6H | 24.23 | 25.65 | 28.28 | **GY** | **W** | **4.03** | QTLxE | No | 0.80 | -0.05 | -0.10 | -0.44 | -0.44 | -0.31 | -0.11 | -0.62 | -0.45 | 0.19 |  |
| 249 | 6H | 29.54 | 29.78 | 29.88 | **GL** | **S** | **7.56** | QTLxE | No | 0.93 | 0.10 | 0.06 | 0.21 | 0.14 | 0.21 | 0.22 | 0.23 | 0.18 | 0.05 |  |
| 250 | 6H | 29.54 | 29.78 | 29.88 | **GR** | **S** | **5.16** | QTLxE | No | 0.93 | -0.01 | 0.00 | -0.01 | -0.01 | -0.01 | -0.01 | -0.01 | -0.01 | 0.00 |  |
| 251 | 6H | 29.98 | 31.98 | 32.38 | **G/E** | **S** | **6.65** | QTLxE | No | 0.91 | 0.00 | 0.00 | -2.10 | -0.89 | -2.89 | -0.62 | -1.70 | -2.23 | 0.58 |  |
| 252 | 6H | 32.38 | 33.55 | 35.38 | **G/E** | **W** | **4.77** | QTLxE | Yes | 0.90 | 0.00 | 0.00 | -0.41 | -1.92 | -1.68 | -0.86 | 1.94 | 1.61 | 0.91 |  |
| 253 | 6H | 38.94 | 40.52 | 42.00 | **GA** | **W** | **4.31** | QTLxE | No | 0.78 | 0.29 | 0.13 | 0.43 | 0.49 | 0.90 | 0.54 | 0.56 | 0.66 | 0.18 |  |
| 254 | 6H | 38.94 | 40.52 | 49.96 | **GL** | **W** | **4.21** | QTLxE | No | 0.78 | 0.10 | 0.05 | 0.15 | 0.17 | 0.27 | 0.16 | 0.19 | 0.24 | 0.06 |  |
| 255 | 6H | 53.08 | 53.38 | 55.28 | **GY** | **W** | **7.43** | QTLxE | No | 0.63 | 0.20 | 0.25 | 0.34 | 0.68 | 0.48 | -0.35 | 0.59 | 0.47 | 0.19 | **6_1** |
| 256 | 6H | 51.40 | 54.92 | 61.87 | **GR** | **W** | **4.31** | QTLxE | No | 0.83 | 0.02 | 0.00 | 0.01 | 0.02 | 0.01 | 0.01 | 0.01 | 0.01 | 0.00 | **6_1** |
| 257 | 6H | 55.18 | 55.25 | 55.28 | **TGW** | **S** | **4.78** | QTLxE | No | 0.61 | 0.03 | 0.84 | 0.19 | 0.68 | 0.61 | 0.89 | 0.44 | 0.74 | 0.22 | **6_1** |
| 258 | 6H | 55.46 | 56.46 | 56.49 | **GL** | **S** | **10.03** | Main | No | 0.72 | 0.16 | 0.16 | 0.16 | 0.16 | 0.16 | 0.16 | 0.16 | 0.16 | 0.04 | **6_1** |
| 259 | 6H | 55.46 | 57.74 | 64.86 | **GA** | **S** | **4.88** | Main | No | 0.93 | -0.65 | -0.65 | -0.65 | -0.65 | -0.65 | -0.65 | -0.65 | -0.65 | 0.17 | **6_1** |
| 260 | 6H | 51.40 | 57.74 | 58.66 | **G/E** | **S** | **5.31** | QTLxE | No | 0.93 | 0.00 | 0.00 | 0.73 | -1.13 | 0.05 | -1.47 | -2.72 | -1.48 | 0.66 | **6_1** |
| 261 | 6H | 55.18 | 59.86 | 60.17 | **GW** | **S** | **4.22** | Main | No | 0.75 | 0.05 | 0.05 | 0.05 | 0.05 | 0.05 | 0.05 | 0.05 | 0.05 | 0.01 | **6_1** |
| 262 | 6H | 59.46 | 59.86 | 60.17 | **GR** | **S** | **11.04** | Main | No | 0.75 | 0.01 | 0.01 | 0.01 | 0.01 | 0.01 | 0.01 | 0.01 | 0.01 | 0.00 |  |
| 263 | 6H | 69.74 | 70.51 | 71.20 | **GW** | **S** | **5.62** | QTLxE | No | 0.72 | 0.00 | 0.00 | 0.05 | 0.01 | 0.02 | 0.00 | 0.04 | 0.04 | 0.01 |  |
| 264 | 6H | 72.35 | 73.01 | 77.42 | **TGW** | **W** | **4.37** | QTLxE | No | 0.58 | -1.37 | 0.21 | -1.23 | -1.08 | -1.70 | -1.27 | -0.84 | -1.23 | 0.42 |  |
| 265 | 6H | 73.36 | 73.59 | 80.57 | **GA** | **W** | **4.19** | QTLxE | No | 0.23 | -0.30 | 0.01 | -0.63 | -0.52 | -0.56 | -0.67 | -0.41 | -0.28 | 0.17 |  |
| 266 | 6H | 74.88 | 75.02 | 83.30 | **GA** | **S** | **6.62** | Main | No | 0.47 | -0.26 | -0.26 | -0.26 | -0.26 | -0.26 | -0.26 | -0.26 | -0.26 | 0.09 |  |
| 267 | 6H | 74.88 | 75.02 | 75.50 | **GL** | **S** | **8.15** | QTLxE | No | 0.41 | -0.03 | -0.05 | -0.12 | -0.11 | -0.10 | -0.12 | -0.12 | -0.08 | 0.03 |  |
| 268 | 6H | 77.97 | 79.50 | 79.63 | **GY** | **W** | **4.16** | QTLxE | No | 0.25 | 0.08 | 0.31 | 0.17 | 0.14 | 0.24 | -0.03 | 0.41 | 0.43 | 0.14 |  |
| 269 | 6H | 82.52 | 82.72 | 83.30 | **TGW** | **S** | **6.85** | Main | No | 0.33 | -0.53 | -0.53 | -0.53 | -0.53 | -0.53 | -0.53 | -0.53 | -0.53 | 0.24 |  |
| 270 | 6H | 84.99 | 87.09 | 91.61 | **G/E** | **S** | **4.06** | Main | No | 0.51 | 0.00 | 0.00 | 1.53 | 1.53 | 1.53 | 1.53 | 1.53 | 1.53 | 0.33 |  |
| 271 | 6H | 91.32 | 91.61 | 92.10 | **GY** | **W** | **4.37** | QTLxE | No | 0.69 | -0.05 | -0.18 | -0.42 | -0.32 | -0.57 | 0.00 | -0.12 | -0.28 | 0.12 |  |
| 272 | 6H | 97.94 | 98.68 | 99.33 | **GA** | **S** | **5.87** | QTLxE | No | 0.31 | 0.29 | 0.10 | 0.44 | 0.26 | 0.38 | 0.34 | 0.32 | 0.24 | 0.09 |  |
| 273 | 6H | 97.94 | 98.68 | 99.33 | **GL** | **S** | **5.35** | QTLxE | No | 0.31 | 0.07 | 0.03 | 0.09 | 0.07 | 0.13 | 0.10 | 0.09 | 0.06 | 0.02 |  |
| 274 | 6H | 98.68 | 99.33 | 99.36 | **GL** | **W** | **7.27** | QTLxE | No | 0.84 | 0.16 | 0.10 | 0.25 | 0.29 | 0.27 | 0.31 | 0.32 | 0.27 | 0.06 |  |
| 275 | 6H | 105.24 | 105.45 | 106.50 | **EL** | **W** | **7.84** | QTLxE | Yes | 0.90 | 0.00 | -0.08 | -0.19 | -0.55 | 0.07 | 14.56 | -0.13 | -33.8 | 1.97 |  |
| 276 | 6H | 107.63 | 107.64 | 109.91 | **GY** | **W** | **4.03** | QTLxE | No | 0.90 | 0.07 | 0.31 | 0.23 | 0.00 | 0.42 | -0.08 | 0.43 | 0.59 | 0.18 |  |
| 277 | 6H | 114.13 | 116.68 | 117.15 | **G/E** | **W** | **5.22** | QTLxE | No | 0.87 | 0.00 | 0.00 | 1.77 | 0.71 | -0.44 | 0.70 | 3.30 | 3.13 | 0.86 |  |
| 278 | 6H | 122.15 | 122.59 | 126.60 | **G/E** | **S** | **6.51** | QTLxE | No | 0.87 | 0.00 | 0.00 | -1.35 | -1.00 | -0.96 | -0.73 | -2.28 | -2.30 | 0.44 | **6_2** |
| 279 | 6H | 118.23 | 126.50 | 126.60 | **GL** | **S** | **5.99** | QTLxE | No | 0.92 | -0.08 | -0.03 | 0.09 | 0.05 | 0.07 | 0.19 | 0.11 | 0.08 | 0.05 | **6_2** |
| 280 | 6H | 123.55 | 126.50 | 126.60 | **GR** | **S** | **6.21** | QTLxE | No | 0.92 | 0.00 | 0.00 | 0.00 | -0.01 | -0.01 | -0.01 | -0.01 | -0.01 | 0.00 | **6_2** |
| 281 | 6H | 123.55 | 126.50 | 126.60 | **GY** | **S** | **5.57** | QTLxE | No | 0.92 | 0.27 | 0.39 | 0.26 | 0.35 | -0.04 | 0.19 | 0.07 | -0.18 | 0.12 | **6_2** |
| 282 | 7H | 0.00 | 0.00 | 0.26 | **GR** | **W** | **5.13** | QTLxE | Yes | 0.70 | -0.01 | 0.01 | -0.01 | -0.01 | -0.01 | 0.00 | 0.00 | -0.01 | 0.00 | **7_1** |
| 283 | 7H | 0.00 | 0.26 | 0.40 | **GA** | **W** | **5.32** | Main | No | 0.64 | 0.56 | 0.56 | 0.56 | 0.56 | 0.56 | 0.56 | 0.56 | 0.56 | 0.14 | **7_1** |
| 284 | 7H | 0.00 | 0.26 | 0.40 | **GW** | **W** | **7.39** | QTLxE | No | 0.68 | -0.06 | 0.00 | -0.05 | -0.05 | -0.08 | -0.06 | -0.05 | -0.05 | 0.01 | **7_1** |
| 285 | 7H | 0.00 | 0.26 | 0.40 | **TGW** | **W** | **6.02** | QTLxE | No | 0.64 | 1.12 | -0.26 | 1.94 | 1.40 | 2.12 | 1.34 | 1.08 | 1.17 | 0.44 | **7_1** |

**RefGeno**	Frequency of the reference genotype allele

**CR09**	Effect of the minor allele at location CRA in 2009

**CR10**	Effect of the minor allele at location CRA in 2010

**HA09**	Effect of the minor allele at location UHA in 2009

**HA10**	Effect of the minor allele at location UHA in 2010

**IP09**	Effect of the minor allele at location IPK in 2009

**IP10**	Effect of the minor allele at location IPK in 2009

**SC09**	Effect of the minor allele at location JHI in 2009

**SC10**	Effect of the minor allele at location JHI in 2010
