# Supplementary material for: Genome‐Wide Association Analysis of Grain Yield‐Associated Traits in a Pan‐European Barley Cultivar Collection
Source: Plant Genome. 2018 Mar 1;11(1):170073. doi: 10.3835/plantgenome2017.08.0073 (PMC12962562; doi:10.3835/plantgenome2017.08.0073)
Supplement: Supplementary file 4 [file TPG2-11-170073-s004.docx]

**Supplemental Table S4: Cereal genes involved in seed size control and their barley orthologues**

| **Cereal Gene** | | | | | | | **Barley Orthologue** | | | | |  |
| --- | --- | --- | --- | --- | --- | --- | --- | --- | --- | --- | --- | --- |
| **Gene Name** | **Protein name** | **Species** | **Accession number** | **Protein category** | **Mutant phenotype** | **Reference(s)** | **Barley new gene designation**^∞^ | **Barley old gene designation^¶^** | **Chromosome** | **Flanking marker left (cM)** | **Flanking marker right (cM)** | **Same barley orthologue?**^ |
| *UBIQUITIN-PROTEASOME PATHWAY* | | | |  |  |  |  |  |  |  |  |  |
| GW2 | GRAIN WIDTH 2 (GW2) | Rice | Os02g0244100 | E3 ubiquitin ligase | Wide grains | [Song et al. 2007](http://link.springer.com/article/10.1007%2Fs00299-013-1524-0#CR24) | [HORVU6Hr1G044080.1](http://apex.ipk-gatersleben.de/apex/f?p=284:45:::NO::P45_GENE_NAME:HORVU6Hr1G044080.1) | [AK250398.1](http://apex.ipk-gatersleben.de/apex/f?p=284:20:::NO::P20_GENE_NAME:AK250398.1) | 6H | 56.46 | 58.66 | same orthologue^ |
| TaGW2 | TaGW2 | Wheat | JN896622,JN896623 | E3 ubiquitin ligase | Large seeds | Su et al. (2011); Bednarek et al. (2012) | [HORVU6Hr1G044080.1](http://apex.ipk-gatersleben.de/apex/f?p=284:45:::NO::P45_GENE_NAME:HORVU6Hr1G044080.1) | [AK250398.1](http://apex.ipk-gatersleben.de/apex/f?p=284:20:::NO::P20_GENE_NAME:AK250398.1) | 6H | 56.46 | 58.66 |  |
| ZmGW2 | ZmGW2 | Maize | EU968771,FJ573211,EU962093 | E3 ubiquitin ligase | Large seeds | Li et al. (2010) | [HORVU6Hr1G044080.1](http://apex.ipk-gatersleben.de/apex/f?p=284:45:::NO::P45_GENE_NAME:HORVU6Hr1G044080.1) | [AK250398.1](http://apex.ipk-gatersleben.de/apex/f?p=284:20:::NO::P20_GENE_NAME:AK250398.1) | 6H | 56.46 | 58.66 |  |
| qSW5/GW5 | SEED WIDTH ON CHROMOSOME 5 (qSW5/GW5) | Rice | DQ991205 | Unknown protein | Wide grains | [Weng et al. 2008](http://link.springer.com/article/10.1007%2Fs00299-013-1524-0#CR29) | HORVU7Hr1G119820.1 |  | 7H | 139.12* | 139.12* |  |
|  |  |  |  |  |  |  |  |  |  |  |  |  |
| *G-PROTEIN SIGNALLLING* | | | |  |  |  |  |  |  |  |  |  |
| RGA1 | RICE G-PROTEIN ALPHA SUBUNIT (RGA1)/DWARF1 (D1) | Rice | Os05g0333200 | G-protein α subunit | Small grains | Ashikari, M. et al. (1999) | [HORVU7Hr1G008720.11](http://apex.ipk-gatersleben.de/apex/f?p=284:45:::NO::P45_GENE_NAME:HORVU7Hr1G008720.11) | [MLOC_67224.3](http://apex.ipk-gatersleben.de/apex/f?p=284:20:::NO::P20_GENE_NAME:MLOC_67224.3) | 7H | 3.04 | 5.57 |  |
| RGB1 | RICE G-PROTEIN BETA SUBUNIT (RGB1) | Rice | Os03g0669100 | G-protein β subunit | Small grains | Utsunomiya et al., 2011 | [HORVU4Hr1G003120.1](http://apex.ipk-gatersleben.de/apex/f?p=284:45:::NO::P45_GENE_NAME:HORVU4Hr1G003120.1) | [MLOC_74118.1](http://apex.ipk-gatersleben.de/apex/f?p=284:20:::NO::P20_GENE_NAME:MLOC_74118.1) | 4H | 7.71 | 14.31 |  |
| GS3 | GRAIN SIZE3 (GS3) | Rice | Os03g0407400 | Homolog of G protein γ subunit | Long grains | [Fan et al. 2006; Mao, H. et al. (2010)](http://link.springer.com/article/10.1007%2Fs00299-013-1524-0#CR3) | HORVU7Hr1G001910.1 |  | 7H | 0.44* | 0.44* |  |
| DEP1 | DENSE AND ERECT PANICLE1 (DEP1) | Rice | Os09g0441900 | Homolog of G protein γ subunit | Short grains of gain-of-function dep1 allele | Huang et al. (2009) | HORVU5Hr1G061840.7 |  | 5H | 46.1 | 46.1 |  |
|  |  |  |  |  |  |  |  |  |  |  |  |  |
| *MAPK SIGNALLING PATHWAY* | | | |  |  |  |  |  |  |  |  |  |
| SMG1 | SMALL GRAIN1 (SMG1) | Rice | Os02g0787300 | Mitogen-activated protein kinase kinase 4 (OsMKK4) | Small grains | Duan et al.,2014 | [HORVU7Hr1G031720.3](http://apex.ipk-gatersleben.de/apex/f?p=284:45:::NO::P45_GENE_NAME:HORVU7Hr1G031720.3) | [AK370268](http://apex.ipk-gatersleben.de/apex/f?p=284:20:::NO::P20_GENE_NAME:AK370268) | 7H | 52.28 | 52.28 |  |
| OsMAPK6 | MITOGEN-ACTIVATED PROTEIN KINASE 6 (OsMAPK6) | Rice | Os06g0154500 | Mitogen-activated protein kinase 6 | Small grains | Liu et al.,2015 | [HORVU7Hr1G023760.3](http://apex.ipk-gatersleben.de/apex/f?p=284:45:::NO::P45_GENE_NAME:HORVU7Hr1G023760.3) | [AK376245](http://apex.ipk-gatersleben.de/apex/f?p=284:20:::NO::P20_GENE_NAME:AK376245) | 7H | 31.02* | 31.02* |  |
|  |  |  |  |  |  |  |  |  |  |  |  |  |
| *PHYTOHORMONE SIGNALLING* | | | | |  |  |  |  |  |  |  |  |
| OsBRI/DWARF61/D61 | BRASSINOSTEROID INSENSITIVE 1 (OsBRI1)/DWARF61 (D61) | Rice | Os01g0718300 | LRR-RLK; BR receptor | Small grains | Morinaka et al., 2006 | [HORVU3Hr1G068000.2](http://apex.ipk-gatersleben.de/apex/f?p=284:45:::NO::P45_GENE_NAME:HORVU3Hr1G068000.2) | [MLOC_5176.2](http://apex.ipk-gatersleben.de/apex/f?p=284:20:::NO::P20_GENE_NAME:MLOC_5176.2) | 3H | 62.15 | 64.27 |  |
| D11 | DWARF 11 (D11) | Rice | Os04g0469800 | CYP724B1; involved in BR biosynthesis | Small grains | Sakamoto et al., 2006 | [HORVU2Hr1G081650.10](http://apex.ipk-gatersleben.de/apex/f?p=284:45:::NO::P45_GENE_NAME:HORVU2Hr1G081650.10) | [AK371371](http://apex.ipk-gatersleben.de/apex/f?p=284:20:::NO::P20_GENE_NAME:AK371371) | 2H | 61.01 | 68.9 |  |
| GS5 | GRAIN SIZE 5 (GS5) | Rice | Os05g0158500 | Putative serine carboxypeptidase | Slender grains | [Li et al. 2011](http://link.springer.com/article/10.1007%2Fs00299-013-1524-0#CR13) | [HORVU3Hr1G033550.19](http://apex.ipk-gatersleben.de/apex/f?p=284:45:::NO::P45_GENE_NAME:HORVU3Hr1G033550.19) | [MLOC_11531.1](http://apex.ipk-gatersleben.de/apex/f?p=284:20:::NO::P20_GENE_NAME:MLOC_11531.1) | 3H | 56.46* | 56.46* |  |
| SRS3 | Small and Round Seed (SRS3/OsKINESIN-13A) | Rice | Os05g0154700 | Kinesin-like protein | small and round seed | Kitagawa et al.,2010;Wu et al.,2014 | [HORVU1Hr1G022250.2](http://apex.ipk-gatersleben.de/apex/f?p=284:45:::NO::P45_GENE_NAME:HORVU1Hr1G022250.2) | [MLOC_60993.2](http://apex.ipk-gatersleben.de/apex/f?p=284:20:::NO::P20_GENE_NAME:MLOC_60993.2) | 1H | 55.11 | 55.45 |  |
| D2 | D2/CYP90D2 | Rice | Os01g0197100 | CYP90D2 |  | Hong et al., 2003; Li et al, 2013 | [HORVU3Hr1G018870.1](http://apex.ipk-gatersleben.de/apex/f?p=284:45:::NO::P45_GENE_NAME:HORVU3Hr1G018870.1) | [MLOC_62829.1](http://apex.ipk-gatersleben.de/apex/f?p=284:20:::NO::P20_GENE_NAME:MLOC_62829.1) | 3H | 46.07 | 46.57 |  |
| OsBZR1 | BRASSINAZOLE-RESISTANT1 (OsBZR1) | Rice | Os07g0580500 | Transcription factor | Large grains by overexpression |  | [HORVU0Hr1G040070.2](http://apex.ipk-gatersleben.de/apex/f?p=284:45:::NO::P45_GENE_NAME:HORVU0Hr1G040070.2) | [AK359453](http://apex.ipk-gatersleben.de/apex/f?p=284:20:::NO::P20_GENE_NAME:AK359453) | 2H | - | - |  |
| GSK3/GSK2 | GSK3/SHAGGY-LIKE KINASE2 (GSK2) | Rice | Os05g0207500 | GSK3/SHAGGY-like; regulator of BR signaling kinase | Large grains | Tong et al, 2012 | [HORVU3Hr1G026020.5](http://apex.ipk-gatersleben.de/apex/f?p=284:45:::NO::P45_GENE_NAME:HORVU3Hr1G026020.5) | [AK364823](http://apex.ipk-gatersleben.de/apex/f?p=284:20:::NO::P20_GENE_NAME:AK364823) | 3H | 48.8 | 48.8 |  |
| qGL3/GL3.1 | GRAIN LENGTH 3 (qGL3/GL3.1) | Rice | Os03g0646900 | Kelch family-Ser/Thr phosphatase (PPKL) | Long grains | [Zhang et al. 2012](http://link.springer.com/article/10.1007%2Fs00299-013-1524-0#CR32) | [HORVU5Hr1G092820.4](http://apex.ipk-gatersleben.de/apex/f?p=284:45:::NO::P45_GENE_NAME:HORVU5Hr1G092820.4) | [MLOC_37399.1](http://apex.ipk-gatersleben.de/apex/f?p=284:20:::NO::P20_GENE_NAME:MLOC_37399.1) | 5H | 111.25 | 118.19 |  |
| TGW6 | THOUSAND-GRAIN WEIGHT 6 (TGW6) | Rice | Os06g0623700 | Indole-3-acetic acid IAA-glucose hydrolase | Large grains | Ishimaru K et al., 2013 | HORVU7Hr1G028580.1 |  | 7H | 36.75 | 44.33 |  |
| BG1 | BIG GRAIN 1 (BG1) | Rice | Os03g0175800 | Unknown protein | Small grains | Liu et al., 2015 | [HORVU4Hr1G076120.1](http://apex.ipk-gatersleben.de/apex/f?p=284:45:::NO::P45_GENE_NAME:HORVU4Hr1G076120.1) | [AK355321](http://apex.ipk-gatersleben.de/apex/f?p=284:20:::NO::P20_GENE_NAME:AK355321) | 4H | 81.3* | 81.3* |  |
| TaGS5 | TaGS5 | Wheat |  | Unknown protein |  | Ma et al., 2016 | - | - | - | - | - |  |
| OsGn1a/OsCKX2^ | Grain number 1a/Cytokinin oxidase 2（OsGn1a/OsCKX2） | Rice | [Os01g0197700](http://rapdb.dna.affrc.go.jp/viewer/gbrowse_details/irgsp1?name=Os01g0197700) | cytokinin oxidase | More tiller number and higher rice Yield | Li et al., 2013; Yeh et al., 2015 | [HORVU3Hr1G027460.1](http://apex.ipk-gatersleben.de/apex/f?p=284:45:::NO::P45_GENE_NAME:HORVU3Hr1G027460.1) | [MLOC_81291.1/ MLOC_53923.1](http://apex.ipk-gatersleben.de/apex/f?p=284:20:::NO::P20_GENE_NAME:MLOC_81291.1) | 3H | 51.27 | 56.15 | same orthologue^ |
| TaCKX6-D1^ | TaCKX6-D1 | Wheat | JQ797673 | cytokinin oxidase |  | Zhang et al., 2012 | [HORVU3Hr1G027460.1](http://apex.ipk-gatersleben.de/apex/f?p=284:45:::NO::P45_GENE_NAME:HORVU3Hr1G027460.1) | [MLOC_81291.1/ MLOC_53923.1](http://apex.ipk-gatersleben.de/apex/f?p=284:20:::NO::P20_GENE_NAME:MLOC_81291.1) | 3H | 51.27 | 56.15 |  |
|  |  |  |  |  |  |  |  |  |  |  |  |  |
| *TRANSCRIPTION CONTROL FACTORS* | | | |  |  |  |  |  |  |  |  |  |
| GW8/OsSPL16 | GRAIN WIDTH 8 (GW8/OsSPL16) | Rice | Os08g0531600 | Squamosa promoter binding protein like16 (OsSPL16) | Slender grains | [Wang et al. 2012](http://link.springer.com/article/10.1007%2Fs00299-013-1524-0#CR27) | [HORVU0Hr1G039170.6](http://apex.ipk-gatersleben.de/apex/f?p=284:45:::NO::P45_GENE_NAME:HORVU0Hr1G039170.6) | [AK355321](http://apex.ipk-gatersleben.de/apex/f?p=284:20:::NO::P20_GENE_NAME:AK355321) | 7H | - | - |  |
| GS2 | GRAIN SIZE 2 (GS2) | Rice | Os02g0701300 | Rice growth-regulating factor 4 (OsGRF4) | Large grains of gain-of-function GS2AA allele | Che et al.,2015 | [HORVU6Hr1G068370.1](http://apex.ipk-gatersleben.de/apex/f?p=284:45:::NO::P45_GENE_NAME:HORVU6Hr1G068370.1) |  | 6H | 64.64 | 65.62 |  |
| OsGIF1 | GROWTH REGULATING FACTOR-INTERACTING FACTOR 1 (OsGIF1) | Rice | Os04g0413500 | Rice GRF-interacting protein 1 | Large grains by overexpression | Wang, et al. 2008 | [HORVU4Hr1G086300.2](http://apex.ipk-gatersleben.de/apex/f?p=284:45:::NO::P45_GENE_NAME:HORVU4Hr1G086300.2) | [MLOC_64782.2](http://apex.ipk-gatersleben.de/apex/f?p=284:20:::NO::P20_GENE_NAME:MLOC_64782.2) | 4H | 39.6 | 41.53 |  |
| GLW7/OsSPL13 | GRAIN LENGTH AND WEIGHT 7 (GLW7/OsSPL13) | Rice | Os07g0505200 | Squamosa promoter binding protein like13 (OsSPL13) | Long grains by overexpression | Si et al., 2016 | [HORVU2Hr1G048280.2](http://apex.ipk-gatersleben.de/apex/f?p=284:45:::NO::P45_GENE_NAME:HORVU2Hr1G048280.2) | [MLOC_13032.1](http://apex.ipk-gatersleben.de/apex/f?p=284:20:::NO::P20_GENE_NAME:MLOC_13032.1) | 2H | 61.01 | 61.01 |  |
| GW6a | GRAIN WEIGHT 6a (GW6a) | Rice | Os06g0650300 | Histone H4 acetyltransferase | Small grains | Song et al., 2015 | [HORVU7Hr1G113480.3](http://apex.ipk-gatersleben.de/apex/f?p=284:45:::NO::P45_GENE_NAME:HORVU7Hr1G113480.3) | [AK355812](http://apex.ipk-gatersleben.de/apex/f?p=284:20:::NO::P20_GENE_NAME:AK355812) | 7H | 122 | 122 |  |
|  |  |  |  |  |  |  |  |  |  |  |  |  |
| *OTHER REGULATORS* | | |  |  |  |  |  |  |  |  |  |  |
| GL7/GW7/SLG7 | GRAIN LENGTH ON CHROMOSOME 7 (GL7)/GRAIN WIDTH 7 (GW7)/SLENDER GRAIN ON CHROMOSOME 7 (SLG7) | Rice | Os07g0603300 | TON1 RECRUITING MOTIF (TRM)-containing protein | Long grains by overexpression | Zhou et al., 2015; Wang et al., 2015 | [HORVU2Hr1G032710.3](http://apex.ipk-gatersleben.de/apex/f?p=284:45:::NO::P45_GENE_NAME:HORVU2Hr1G032710.3) | [AK364466](http://apex.ipk-gatersleben.de/apex/f?p=284:20:::NO::P20_GENE_NAME:AK364466) | 2H | 58.66* | 58.66* |  |
| GE | GIANT EMBRYO (GE) | Rice | Os07g0603700 | CYP78A13 | Large grains by overexpression | Nagasawa et al. 2013 | [HORVU2Hr1G032890.2](http://apex.ipk-gatersleben.de/apex/f?p=284:45:::NO::P45_GENE_NAME:HORVU2Hr1G032890.2) | [MLOC_68312.1](http://apex.ipk-gatersleben.de/apex/f?p=284:20:::NO::P20_GENE_NAME:MLOC_68312.1) | 2H | 61.04 | 61.01 |  |
| TaCYP78A3 | TaCYP78A3 | Wheat | KP768392,KP768393,KP768394 | CYP78A3 |  | Ma et al., 2015 | [HORVU7Hr1G057100.2](http://apex.ipk-gatersleben.de/apex/f?p=284:45:::NO::P45_GENE_NAME:HORVU7Hr1G057100.2) | [MLOC_68718.1](http://apex.ipk-gatersleben.de/apex/f?p=284:20:::NO::P20_GENE_NAME:MLOC_68718.1) | 7H |  |  |  |
| MOC1 | MONOCULM 1 (MOC1) | Rice | Os06g0610350 | putative GRAS family nuclear protein | nly a main culm without any tillers | Li et al., 2003 | [HORVU7Hr1G091000.1](http://apex.ipk-gatersleben.de/apex/f?p=284:45:::NO::P45_GENE_NAME:HORVU7Hr1G091000.1) | [AK364492](http://apex.ipk-gatersleben.de/apex/f?p=284:20:::NO::P20_GENE_NAME:AK364492) | 7H | 81.89 | 82.51 | same orthologue^ |
| TaMOC1 | TaMOC1 | Wheat | KR758748 | Unknown protein | less spikelet number per spike | Zhang et al.,2015 | [HORVU7Hr1G091000.1](http://apex.ipk-gatersleben.de/apex/f?p=284:45:::NO::P45_GENE_NAME:HORVU7Hr1G091000.1) | [AK364492](http://apex.ipk-gatersleben.de/apex/f?p=284:20:::NO::P20_GENE_NAME:AK364492) | 7H | 81.89 | 82.51 |  |
| OsMED4_2 | OsMED4_2 | Rice | LOC_Os11g05150 | hydroxyproline-rich glycoprotein family protein, |  | Malik et al 2016 | [HORVU0Hr1G040300.1](http://apex.ipk-gatersleben.de/apex/f?p=284:45:::NO::P45_GENE_NAME:HORVU0Hr1G040300.1) | [MLOC_52916.1](http://apex.ipk-gatersleben.de/apex/f?p=284:20:::NO::P20_GENE_NAME:MLOC_52916.1) |  | - | - |  |
| qSPP6 qTGW6 | qSPP6 qTGW6 | Rice | Os06g0666600 | Glyceraldehyde-3-phosphate dehydrogenase |  | Kim et al 2014 | HORVU7Hr1G108580.2 | [MLOC_66206.1](http://apex.ipk-gatersleben.de/apex/f?p=284:20:::NO::P20_GENE_NAME:MLOC_66206.1) | 7H | 111.98* | 111.98* |  |
| SRS5/TID1 | Small and round seed 5(SRS5/TID1) | Rice | Os11g0247300 | α-tubulin | small and round seed | Segami et al., 2012 | [HORVU4Hr1G009520.1](http://apex.ipk-gatersleben.de/apex/f?p=284:45:::NO::P45_GENE_NAME:HORVU4Hr1G009520.1) | [MLOC_69930.1](http://apex.ipk-gatersleben.de/apex/f?p=284:20:::NO::P20_GENE_NAME:MLOC_69930.1) | 4H | 31.42 | 37.4 |  |
| SRS1/DEP2 | Small and round seed 1(SRS1/DEP2) | Rice | [Os07g0616000](http://rapdb.dna.affrc.go.jp/viewer/gbrowse_details/irgsp1?name=Os07g0616000) | Unknown protein | small and round seed | Abe et al., 2010 | [HORVU2Hr1G031030.1](http://apex.ipk-gatersleben.de/apex/f?p=284:45:::NO::P45_GENE_NAME:HORVU2Hr1G031030.1) | [AK361285](http://apex.ipk-gatersleben.de/apex/f?p=284:20:::NO::P20_GENE_NAME:AK361285) | 2H | 58.17 | 60.72 |  |
| OsMADS87 | OsMADS87 | Rice | OS03G38610 | MADS-box transcription factor family protein | seed size reduction | Chen et al., 2016 | - |  | - | - | - |  |
| OsMADS29 | OsMADS29 | Rice | [Os02g0170300](http://rapdb.dna.affrc.go.jp/viewer/gbrowse_details/irgsp1?name=Os02g0170300) | MADS-box transcription factor family protein | shriveled seed | Yang et al., 2012 | [HORVU6Hr1G032220.8](http://apex.ipk-gatersleben.de/apex/f?p=284:45:::NO::P45_GENE_NAME:HORVU6Hr1G032220.8) | [MLOC_65966.1](http://apex.ipk-gatersleben.de/apex/f?p=284:20:::NO::P20_GENE_NAME:MLOC_65966.1) | 6H | 55.28 | 55.43 |  |
| An-1 | An-1 | Rice | [Os04g0350700](http://rapdb.dna.affrc.go.jp/viewer/gbrowse_details/irgsp1?name=Os04g0350700) | [helix-loop-helix protein](http://www.ncbi.nlm.nih.gov/pubmed?term=An-1%20encodes%20a%20basic%20helix-loop-helix%20protein%20that%20regulates%20awn%20development,%20grain%20size,%20and%20grain%20number%20in%20rice%5BTitle%5D) | shorter grains and fewer grain numbers | Luo et al., 2013 | [HORVU2Hr1G067250.4](http://apex.ipk-gatersleben.de/apex/f?p=284:45:::NO::P45_GENE_NAME:HORVU2Hr1G067250.4) | [MLOC_66841.1](http://apex.ipk-gatersleben.de/apex/f?p=284:20:::NO::P20_GENE_NAME:MLOC_66841.1) | 2H | 61.01 | 61.01 |  |

*	Marker in the gene

^	These cereal genes from different species identify the same barley orthologue and may be orthologous to each other

¶	IBSC 2012

∞	Mascher et al 2017
