# Supplementary material for: Genome‐Wide Association Analysis of Grain Yield‐Associated Traits in a Pan‐European Barley Cultivar Collection
Source: Plant Genome. 2018 Mar 1;11(1):170073. doi: 10.3835/plantgenome2017.08.0073 (PMC12962562; doi:10.3835/plantgenome2017.08.0073)
Supplement: Supplementary file 5 [file TPG2-11-170073-s005.docx]

**Supplemental References**

**These references relate to the Supplemental Materials.**

Ando, I. Kono, M. Yano, H. Kitano, and Y. Iwasaki. 2010. The *SMALL AND ROUND SEED1* (*SRS1/DEP2*) gene is involved in the regulation of seed size in rice. Genes Genet. Syst. 85(5): 327–339.

Ashikari, M., J. Wu, M. Yano, T. Sasaki, and a Yoshimura. 1999. Rice gibberellin-insensitive dwarf mutant gene *Dwarf 1* encodes the alpha-subunit of GTP-binding protein. Proc. Natl. Acad. Sci. U. S. A. 96(18): 10284–10289.

Bednarek, J., A. Boulaflous, C. Girousse, C. Ravel, C. Tassy, P. Barret, M.F. Bouzidi, and S. Mouzeyar. 2012. Down-regulation of the *TaGW2* gene by RNA interference results in decreased grain size and weight in wheat. J. Exp. Bot. 63(16): 5945–5955.

Che, R., H. Tong, B. Shi, Y. Liu, S. Fang, D. Liu, Y. Xiao, B. Hu, L. Liu, H. Wang, M. Zhao, and C. Chu. 2015. Control of grain size and rice yield by GL2-mediated brassinosteroid responses. Nat. Plants 2(1): 15195.

Chen, C., K. Begcy, K. Liu, J.J. Folsom, Z. Wang, C. Zhang, and H. Walia. 2016. Molecular characterization of rice endosperm development under heat stress identifies *OsMADS87* as a determinant of seed size and thermal sensitivity. Plant Physiol. 171(1): 606–622.

Duan, P., Y. Rao, D. Zeng, Y. Yang, R. Xu, B. Zhang, G. Dong, Q. Qian, and Y. Li. 2014. *SMALL GRAIN 1*, which encodes a mitogen-activated protein kinase kinase 4, influences grain size in rice. Plant J. 77(4): 547–557.

Fan, C., Y. Xing, H. Mao, T. Lu, B. Han, C. Xu, X. Li, and Q. Zhang. 2006. *GS3*, a major QTL for grain length and weight and minor QTL for grain width and thickness in rice, encodes a putative transmembrane protein. Theor. Appl. Genet. 112(6): 1164–1171.

Hong, Z., M. Ueguchi-Tanaka, K. Umemura, S. Uozu, S. Fujioka, S. Takatsuto, S. Yoshida, M. Ashikari, H. Kitano, and M. Matsuoka. 2003. A rice brassinosteroid-deficient mutant, *ebisu dwarf* (*d2*), is caused by a loss of function of a new member of cytochrome P450. Plant Cell 15(12): 2900–10.

Huang, X., Q. Qian, Z. Liu, H. Sun, S. He, D. Luo, G. Xia, C. Chu, J. Li, and X. Fu. 2009. Natural variation at the *DEP1* locus enhances grain yield in rice. Nat. Genet. 41(4): 494–497.

Ishimaru, K., N. Hirotsu, Y. Madoka, N. Murakami, N. Hara, H. Onodera, T. Kashiwagi, K. Ujiie, B. Shimizu, A. Onishi, H. Miyagawa, and E. Katoh. 2013. Loss of function of the IAA-glucose hydrolase gene *TGW6* enhances rice grain weight and increases yield. Nat. Genet. 45(6): 707–711.

Li, Y., C. Fan, Y. Xing, Y. Jiang, L. Luo, L. Sun, D. Shao, C. Xu, X. Li, J. Xiao, Y. He, and Q. Zhang. 2011. Natural variation in *GS5* plays an important role in regulating grain size and yield in rice. Nat. Genet. 43(12): 1266–9.

Li, H., L. Jiang, J.-H. Youn, W. Sun, Z. Cheng, T. Jin, X. Ma, X. Guo, J. Wang, X. Zhang, F. Wu, C. Wu, S.-K. Kim, and J. Wan. 2013. A comprehensive genetic study reveals a crucial role of CYP90D2/D2 in regulating plant architecture in rice (*Oryza sativa*). New Phytol. 200(4): 1076–1088.

Li, F., W. Liu, J. Tang, J. Chen, H. Tong, B. Hu, C. Li, J. Fang, M. Chen, and C. Chu. 2010. Rice *DENSE AND ERECT PANICLE 2* is essential for determining panicle outgrowth and elongation. Cell Res. 20: 838–849.

Li, X., Q. Qian, Z. Fu, Y. Wang, G. Xiong, D. Zeng, X. Wang, X. Liu, S. Teng, F. Hiroshi, M. Yuan, D. Luo, B. Han, and J. Li. 2003. Control of tillering in rice. Nature 422(6932): 618–621.

Liu, S., L. Hua, S. Dong, H. Chen, X. Zhu, J. Jiang, F. Zhang, Y. Li, X. Fang, and F. Chen. 2015. *OsMAPK6*, a mitogen-activated protein kinase, influences rice grain size and biomass production. Plant J. 84(4): 672–681.

Ma, L., T. Li, C. Hao, Y. Wang, X. Chen, and X. Zhang. 2016. TaGS5-3A, a grain size gene selected during wheat improvement for larger kernel and yield. Plant Biotechnol. J. 14(5): 1269–1280.

Ma, M., Q. Wang, Z. Li, H. Cheng, Z. Li, X. Liu, W. Song, R. Appels, and H. Zhao. 2015. Expression of *TaCYP78A3*, a gene encoding cytochrome P450 CYP78A3 protein in wheat (*Triticum aestivum* L.), affects seed size. Plant J. 83(2): 312–325.

Malik, N., N. Dwivedi, A.K. Singh, S.K. Parida, P. Agarwal, J.K. Thakur, and A.K. Tyagi. 2016. An Integrated Genomic Strategy Delineates Candidate Mediator Genes Regulating Grain Size and Weight in Rice. Sci. Rep. 6(January): 23253.

Mao, H., S. Sun, J. Yao, C. Wang, S. Yu, C. Xu, X. Li, and Q. Zhang. 2010. Linking differential domain functions of the GS3 protein to natural variation of grain size in rice. Proc Natl Acad Sci U S A 107(45): 19579–19584.

Morinaka, Y., T. Sakamoto, Y. Inukai, M. Agetsuma, H. Kitano, M. Ashikari, and M. Matsuoka. 2006. Morphological alteration caused by brassinosteroid insensitivity increases the biomass and grain production of rice. Plant Physiol. 141(3): 924–31.

Nagasawa, N., K.I. Hibara, E.P. Heppard, K.A. Vander Velden, S. Luck, M. Beatty, Y. Nagato, and H. Sakai. 2013. *GIANT EMBRYO* encodes CYP78A13, required for proper size balance between embryo and endosperm in rice. Plant J. 75(4): 592–605.

Sakamoto, T., Y. Morinaka, T. Ohnishi, H. Sunohara, S. Fujioka, M. Ueguchi-Tanaka, M. Mizutani, K. Sakata, S. Takatsuto, S. Yoshida, H. Tanaka, H. Kitano, and M. Matsuoka. 2006. Erect leaves caused by brassinosteroid deficiency increase biomass production and grain yield in rice. Nat. Biotechnol. 24(1): 105–109.

Segami, S., I. Kono, T. Ando, M. Yano, H. Kitano, K. Miura, and Y. Iwasaki. 2012. Small and round seed 5 gene encodes alpha-tubulin regulating seed cell elongation in rice. Rice 5(1): 4.

Si, L., J. Chen, X. Huang, H. Gong, J. Luo, Q. Hou, T. Zhou, T. Lu, J. Zhu, Y. Shangguan, E. Chen, C. Gong, Q. Zhao, Y. Jing, Y. Zhao, Y. Li, L. Cui, D. Fan, Y. Lu, Q. Weng, Y. Wang, Q. Zhan, K. Liu, X. Wei, K. An, G. An, and B. Han. 2016. *OsSPL13* controls grain size in cultivated rice. Nat. Genet. 48(4): 447–456.

Song, X.-J., W. Huang, M. Shi, M.-Z. Zhu, and H.-X. Lin. 2007. A QTL for rice grain width and weight encodes a previously unknown RING-type E3 ubiquitin ligase. Nat. Genet. 39(5): 623–630.

Song, X.J., T. Kuroha, M. Ayano, T. Furuta, K. Nagai, N. Komeda, S. Segami, K. Miura, D. Ogawa, T. Kamura, T. Suzuki, T. Higashiyama, M. Yamasaki, H. Mori, Y. Inukai, J. Wu, H. Kitano, H. Sakakibara, S.E. Jacobsen, and M. Ashikari. 2015. Rare allele of a previously unidentified histone H4 acetyltransferase enhances grain weight, yield, and plant biomass in rice. Proc. Natl. Acad. Sci. U. S. A. 112(1): 76–81.

Su, Z., C. Hao, L. Wang, Y. Dong, and X. Zhang. 2011. Identification and development of a functional marker of TaGW2 associated with grain weight in bread wheat (*Triticum aestivum* L.). Theor. Appl. Genet. 122(1): 211–23.

Tong, H., L. Liu, Y. Jin, L. Du, Y. Yin, Q. Qian, L. Zhu, and C. Chu. 2012. DWARF AND LOW-TILLERING acts as a direct downstream target of a GSK3/SHAGGY-like kinase to mediate brassinosteroid responses in rice. Plant Cell 24(6): 2562–77.

Utsunomiya, Y., C. Samejima, Y. Takayanagi, Y. Izawa, T. Yoshida, Y. Sawada, Y. Fujisawa, H. Kato, and Y. Iwasaki. 2011. Suppression of the rice heterotrimeric G protein β-subunit gene, RGB1, causes dwarfism and browning of internodes and lamina joint regions. Plant J. 67(5): 907–916.

Wang, E., J. Wang, X. Zhu, W. Hao, L. Wang, Q. Li, L. Zhang, W. He, B. Lu, H. Lin, H. Ma, G. Zhang, and Z. He. 2008. Control of rice grain-filling and yield by a gene with a potential signature of domestication. Nat. Genet. 40(11): 1370–1374.

Wang, S., K. Wu, Q. Yuan, X. Liu, Z. Liu, X. Lin, R. Zeng, H. Zhu, G. Dong, Q. Qian, G. Zhang, and X. Fu. 2012. Control of grain size, shape and quality by *OsSPL16* in rice. Nat. Genet. 44(8): 950–4.

Wang, S., X. Zhang, F. Chen, and D. Cui. 2015. A single-nucleotide polymorphism of *TaGS5* gene revealed its association with kernel weight in chinese bread wheat. Front. Plant Sci. 6: 1166.

Weng, J.F., S.H. Gu, X.Y. Wan, H. Gao, T. Guo, N. Su, C.L. Lei, X. Zhang, Z.J. Cheng, X.P. Guo, J.L. Wang, L. Jiang, H.Q. Zhai, and J.M. Wan. 2008. Isolation and initial characterization of *GW5*, a major QTL associated with rice grain width and weight. Cell Res. 18(12): 1199–1209.

Wu, T., Y. Shen, M. Zheng, C. Yang, Y. Chen, Z. Feng, X. Liu, S. Liu, Z. Chen, C. Lei, J. Wang, L. Jiang, and J. Wan. 2014. Gene *SGL*, encoding a kinesin-like protein with transactivation activity, is involved in grain length and plant height in rice. Plant Cell Rep. 33(2): 235–244.

Yang, X., F. Wu, X. Lin, X. Du, K. Chong, L. Gramzow, S. Schilling, A. Becker, G. Theißen, and Z. Meng. 2012. Live and Let Die - The Bsister MADS-Box gene *OsMADS29* controls the degeneration of cells in maternal tissues during seed development of rice (*Oryza sativa*). PLoS One 7(12).

Yeh, S.-Y., H.-W. Chen, C.-Y. Ng, C.-Y. Lin, T.-H. Tseng, W.-H. Li, and M.S.B. Ku. 2015. Down-regulation of *Cytokinin Oxidase 2* expression increases tiller number and improves rice yield. Rice (N. Y). 8(1): 36.

Zhang, D., J. Li, R.O. Compton, J. Robertson, V.H. Goff, E. Epps, W. Kong, C. Kim, and A.H. Paterson. 2015. Comparative genetics of seed size traits in divergent cereal lineages represented by sorghum (Panicoidae) and rice (Oryzoidae). G3 (Bethesda). 5(6): 1117–1128.

Zhang, L., Y.L. Zhao, L.F. Gao, G.Y. Zhao, R.H. Zhou, B.S. Zhang, and J.Z. Jia. 2012. *TaCKX6-D1*, the ortholog of rice *OsCKX2*, is associated with grain weight in hexaploid wheat. New Phytol. 195(3): 574–584.

Zhou, Y., J. Miao, H. Gu, X. Peng, M. Leburu, F. Yuan, H. Gu, Y. Gao, Y. Tao, J. Zhu, Z. Gong, C. Yi, M. Gu, Z. Yang, and G. Liang. 2015. Natural variations in *SLG7* regulate grain shape in rice. Genetics 201(4): 1591–1599.
