# Supplementary material for: Genome‐Wide Association Analysis of Grain Yield‐Associated Traits in a Pan‐European Barley Cultivar Collection
Source: Plant Genome. 2018 Mar 1;11(1):170073. doi: 10.3835/plantgenome2017.08.0073 (PMC12962562; doi:10.3835/plantgenome2017.08.0073)
Supplement: Supplementary file 6 [file TPG2-11-170073-s006.docx]

**Supplemental Materials**

**Supplemental Figures	2**

**Supplemental Tables	4**

**Supplemental references	1**

**Supplemental Figures**

Spring

Winter

**Supplemental Figure S1: Variance component analysis of traits studied here.** Data from spring and winter trials are shown.

3H

2H

1H

30

25

20

15

10

5

0

0

10

5

0

15

10

5

0

30

25

20

15

10

5

0

6H

5H

4H

10

5

0

10

5

0

7H

10

5

0

**Supplemental Figure S2: GWAS plots for 8 yield-related phenotypic traits in barley germplasm.** Scans are colour-coded for trait as shown. X axis, Log_10_ probabilities. Y axes, marker numbers. The approximate locations of LR-PCH regions (Baker *et al.* 2014) are indicated by grey shading.
